# Unraveling the Life History of Past Populations through Hypercementosis: Insights into Cementum Apposition Patterns and Possible Etiologies Using Micro-CT and Confocal Microscopy

**DOI:** 10.3390/biology13010043

**Published:** 2024-01-13

**Authors:** Léa Massé, Emmanuel d’Incau, Antoine Souron, Nicolas Vanderesse, Frédéric Santos, Bruno Maureille, Adeline Le Cabec

**Affiliations:** 1 Univ. Bordeaux, CNRS, Ministère de la Culture, PACEA, UMR 5199, F-33600 Pessac, France; antoine.souron@u-bordeaux.fr (A.S.); nicolas.vanderesse@u-bordeaux.fr (N.V.); frederic.santos@u-bordeaux.fr (F.S.); bruno.maureille@u-bordeaux.fr (B.M.); adeline.le-cabec@u-bordeaux.fr (A.L.C.); 2 UFR des Sciences Odontologiques de Bordeaux, Univ. Bordeaux, F-33076 Bordeaux, France; emmanuel.dincau@u-bordeaux.fr; 3 University Hospital, F-33076 Bordeaux, France; 4 Univ. Bordeaux, CNRS, SANPSY, UMR 6033, F-33000 Bordeaux, France

**Keywords:** dental root, paleopathology, teeth-as-tools, impacted teeth, super-erupted tooth, incisor

## Abstract

**Simple Summary:**

In past populations, some individuals used their front teeth as tools for purposes other than eating, such as making tools or preparing food. These activities have an impact on both the visible part of the tooth (crown) through wear, as well as the part anchored deep in the gum (tooth root) by causing excessive production of a mineralized tissue called ‘cementum’. Cementum surrounds the tooth root and forms throughout an individual’s life. It records information about the individual’s oral health, physiology, and chewing activities. Under certain conditions (e.g., significant mechanical stress or infection), cementum production becomes excessive and exceeds normal physiological limits, resulting in ‘hypercementosis’. The present study aims to identify different patterns of cementum apposition and to propose probable causes. We examined a sample of 35 teeth (Sains-en-Gohelle, France, 7th–17th century) and identified four groups of cementum apposition: (i) impacted teeth that never erupted in the mouth, (ii) infected teeth, with caries or gum disease, (iii) hyperfunctional teeth subjected to high mechanical stress, and (iv) hypofunctional teeth that were not used in chewing due to the loss of opposing teeth. We conclude that hypercementosis could provide information on the life history of archeological specimens, even based on isolated teeth.

**Abstract:**

The “teeth-as-tools” hypothesis posits that Neanderthals used their anterior teeth as a tool or a third hand for non-dietary purposes. These non- or para-masticatory activities (e.g., tool-making or food preparation prior to ingestion) have also been described in other past and extant human populations, and other Primates. Cementum is the mineralized tissue that covers the tooth root surface and anchors it to the alveolar bone. Under certain conditions (e.g., mechanical stress, infection), its production becomes excessive (i.e., beyond the physiological state) and is called ‘hypercementosis’. Several studies in dental anthropology have established a correlation between the teeth-as-tools and hypercementosis. The present work aims to characterize the different patterns of cementum apposition on archeological teeth and discuss their supposed etiology. Using microtomography and confocal microscopy, the patterns of cementum apposition (i.e., thickness, location, and surface characteristics) were analyzed in 35 hypercementotic teeth (Sains-en-Gohelle, France; 7th–17th c. A.D.). Four groups were identified with distinct hypercementosis patterns: (1) impacted, (2) infected, (3) hypofunctional, and (4) hyperfunctional teeth. Characterizing hypercementosis can contribute to documenting the oral health status (paleopathology) and/or masticatory activity of individuals, even from isolated teeth. This has implications for the study of fossil hominins, particularly Neanderthals, known for their use of anterior teeth as tools and frequent and substantial occurrence of hypercementosis.

## 1. Introduction

The use of teeth for non- or para-masticatory purposes has been frequently described in ethnological and archeological reports (e.g., [1,2,3]). These non-masticatory behaviors involve the use of anterior teeth in the production of objects or tools, including activities such as weaving, tanning, and wood softening (e.g., [4,5,6]). Concerning para-masticatory activities, the anterior teeth are involved in food preparation before ingestion (e.g., [7,8]). The “stuff-and-cut” hypothesis, for example, suggests that Neanderthals used their anterior teeth as a clamp to hold a piece of meat, while the other hand used a tool to cut a portion of the flesh [7,8]. This idea was derived from behaviors observed in Arctic populations using their anterior teeth in this manner [9,10]. Such behaviors have been observed in numerous hunter-gatherer societies (e.g., [11]). While the earliest evidence of para-masticatory activities has been documented on teeth of individuals belonging to the genus *Homo* dating from 2.1 to 1.7 million years ago [12], the most numerous examples concern the Neanderthal lineage (e.g., [13,14,15,16]) which gave rise to the “teeth-as-tools” hypothesis. According to this hypothesis, Neanderthals were using their anterior teeth as a third hand for non- or para-masticatory activities [8,17,18]. In these cases, repetitive movements also leave specific features on the teeth (i.e., macro- or micro-wear) different from those associated with “normal” tooth use [19]. 

Distinctive features, such as chipping, fractures, and grooves, have been described mostly on the enamel of the tooth crown (e.g., [20]). These markers can provide information about the activities and social roles of an individual, varying by age and sex (e.g., dental wear differences between men and women; [2,11,21]). Therefore, the analysis of these dental markers is essential for better understanding the daily life and social dynamics of past populations.

Dental cementum is a mineralized tissue that covers the tooth root. Different types of cementum exist, each with its distinct characteristics and specific roles (e.g., [22,23]). Acellular cementum forms during the tooth development period, continues throughout the life of the tooth, and plays a crucial role in anchoring periodontal ligament fibers to the root surface, ensuring the stability of the tooth in the alveolar bone. It is mainly located on the cervical half of the root and lacks cementocytes and fibrils (e.g., [22,24]). Cellular cementum forms after tooth eruption and throughout the lifespan of the tooth. It has a more complex structure, with specialized cells called cementoblasts (e.g., [22]), and is mostly located in the apical half of the root and in the furcation areas of multi-rooted teeth. It serves several functions, including root repair, protection of the underlying dentine against resorption, and maintenance of the tooth within its bony crypt [22,24,25,26]. Additionally, this cellular cementum can become a reactive cementum and its excessive apposition, exceeding its physiological limit, is called ‘hypercementosis’ (e.g., [27,28]). Its etiologies are still not fully understood, since excessive apposition of this cementum occurs in response to stimuli such as intensive masticatory efforts, dental carious lesions, periodontal disease, or impaction (e.g., review in [29]).

Several scholars also established a positive correlation between the aforementioned intense and repetitive masticatory efforts and excessive cementum apposition around the tooth root (e.g., [15,30,31]). This abnormal thickening of cementum—’hypercementosis’—leads to a modification of the natural morphology of the root (e.g., [30]). Such root shape alterations have even been described in Neanderthals as “club-shaped” or “drumstick-shaped” (e.g., La Quina 5, [32]; La Ferrassie 1, [33]; Regourdou, [34]; Shanidar 1 and 2, [35]; cited in [36]). 

Some researchers have also observed significant cementum accumulation on the roots of carious teeth (e.g., [37,38]). Other studies have highlighted factors related to periodontal disease (e.g., [36,39]) or impacted teeth (e.g., [40,41]). Others have also observed hypercementosis on deciduous teeth (Maureille, Le Cabec, pers. obs.) Clinical cases involving unworn contemporary teeth have also documented the presence of hypercementosis (e.g., [42,43]). It appears that this manifestation can take different forms: (i) in terms of location, hypercementosis can be localized in a single root third (i.e., apical, middle, or cervical) or in several; (ii) in terms of extent, it can be diffuse and involve a large portion of the tooth root or rather be localized in a restricted location; (iii) in terms of form, hypercementosis can be strongly expressed and visible, and thus drastically modify the initial morphology of the root, or it can be moderate and respect the overall root morphology while altering its apical shape. Some patterns of apposition seem to be related to a specific etiology. On the one hand, in some teeth affected by heavy wear, the apposition of hypertrophic cementum appears to be localized on the apical root third with a preferential accumulation on the lingual aspect of the root, which would favor an etiology involving intense masticatory loads (e.g., [29,30,44]). On the other hand, the roots of impacted teeth or teeth with severe carious lesions (i.e., infected) show an apposition involving the whole root surface. The features of hypercementosis will appear milder in the case of impaction, while they will generally be much more accentuated in infected teeth (e.g., [29]).

Hypercementosis is a manifestation that takes on various forms, with a variable thickness of apposition, surface condition, and location on the dental root. Faced with different patterns of apposition and within the context of multiple etiologies, two research questions arise: (1) Can we characterize these different cementum appositions (i.e., thickness, location, and surface condition) and extract specific profiles unique to each supposed etiology of hypercementosis? (2) Could the hypercementosis of an isolated tooth provide information about para-masticatory activities and/or serve as a marker of oral health in a fossil/archeological individual?

In order to characterize different profiles of hypercementosis, the present study focuses on archeological teeth. By carefully analyzing the associated osteological context and examining the intrinsic history recorded in these teeth, we intend to link supposed etiologies with these different patterns of cementum apposition. This is carried out with two complementary imaging techniques: microtomography (i.e., 3D thickness maps of cementum) and confocal microscopy (i.e., high-resolution 3D topography maps of cementum surface). By investigating the connections between hypercementosis, dental health, cultural and environmental conditions, we strive to improve our understanding of living conditions, health, adaptations and life history in past human populations.

## 2. Materials and Methods

### 2.1. Sample

**Figure 1:**
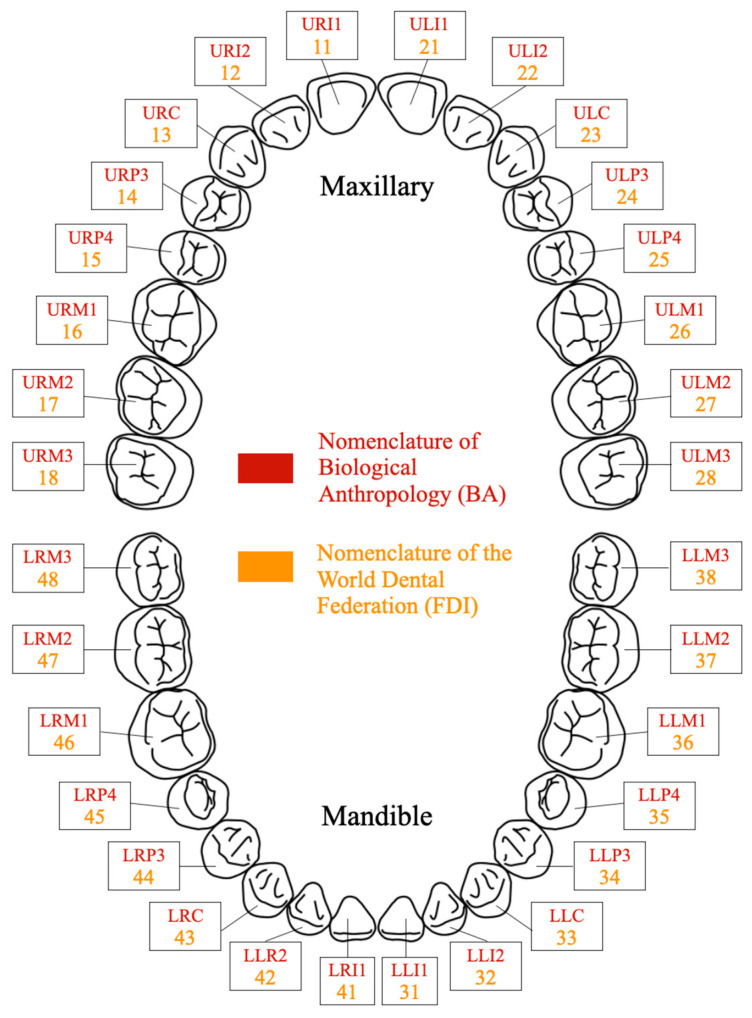
Numbering and labeling of teeth in accordance with the FDI World Dental Federation (FDI: Fédération Dentaire Internationale, in French) convention and the norms used in Biological Anthropology (BA).

Our sample comprises specimens from an archeological collection dating from the Middle Ages to the early modern period, from Sains-en-Gohelle (Pas-de-Calais, Hauts-de-France, France, 7th to 17th centuries; [45]). This population is of particular interest not only because of its high frequency of hypercementosis in comparison to other historical populations [28,46], but also because of its extreme periodontal damage and overall poor oral health. We selected 35 single-rooted human maxillary and mandibular permanent teeth coming from 23 individuals. Within the scope of this study, we have chosen to limit our analysis to single-rooted teeth due to the specific pattern of cementum apposition observed in multi-rooted teeth. In the latter, cellular cementum naturally forms at the root furcation level (i.e., at the junction of the roots), because of the biomechanical stimulations undergone by the multi-rooted teeth during mastication, and due to the location of the fulcrum of the tooth [47,48]. This would not allow for a precise characterization of the cementum deposition around each individual root due to multiple factors. By focusing on single-rooted teeth, we ensure a more representative characterization of the specific cementum features (especially thickness) for each specimen. The teeth were preserved in situ in the jawbone and, whenever possible, were gently removed from their bone sockets. These 23 individuals involve eight females, thirteen males, two indeterminates, and one individual for whom it was not possible to determine the sex due to missing data in the osteological collection [45]. All individuals in our sample were estimated to be over 20 years of age (Table 1). The age-at-death estimation and sex assessment of the Sains-en-Gohelle specimens of our sample (See Table 1) were previously determined by Beauval et al. and reported in their excavation report (see [45] for methodological details). The selected teeth comprise four central incisors (I1), two lateral incisors (I2), nine canines (C), nine first premolars (P3) and 11 second premolars (P4) (See details in Table 1). To simplify the naming of teeth, in this manuscript, we use the specimen ID followed by an underscore and the tooth ID using the FDI convention (Fédération Dentaire Internationale, in French) (Figure 1), for instance: “Sp755_13” is the maxillary right canine (URC for dental anthropologists) of specimen Sp755.

### 2.2. Multi-Method Analysis: Visual Inspection, Microtomography, and Confocal Microscopy

In this study, the first step involved a visual examination of the teeth and their surrounding osteological context, followed by the evaluation of hypercementosis according to the classification established by Massé et al. [29]. Subsequently, hypercementosis was characterized in 2D and 3D, focusing on cementum distribution and thickness, as well as surface characterization. The detailed analyses are presented in Supplementary Information S1.

#### 2.2.1. Visual Examination

A visual examination conducted by three observers of the selected teeth enabled the scoring of different features which are known to potentially influence cementum apposition—occlusal wear, carious lesions, pulp exposure, whether teeth were impacted or not—and score the presence of hypercementosis. An examination of the bone context completes the analysis.

**Figure 2:**
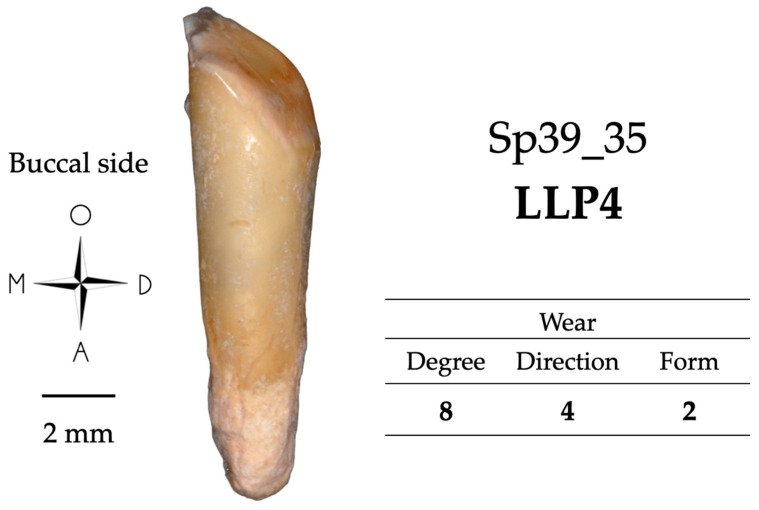
Wear score (following Molnar, 1971 [50]) of the left lower second premolar of individual Sp39. The wear pattern in this tooth may suggest para-masticatory behaviors. Abbreviations stand as follows: O = occlusal; M = mesial; D = distal; A = apical.

Tooth wear is the deterioration of the occlusal or incisal crown surface produced by the dietary and possibly non-dietary use of the teeth ([49,50]). It is an indication of the function of a tooth and of the stresses it has undergone. Molnar ’s classification [48] was used to evaluate occlusal wear using three criteria: (i) degree of wear: from 1 (no wear) to 8 (major wear, the tooth crown is totally worn away, and the chewing surface is on the root itself); (ii) direction of the worn surface: natural (1), oblique (2 to 5), horizontal (6) or rounded (7 and 8); and (iii) form of the worn surface: natural (1), flat (2), half or fully concave (3 and 4), notched (5) or rounded (6). Some types of atypical wear, with a particular form or direction, for example, may suggest some para-masticatory activities. All the teeth in the Sains-en-Gohelle sample were scored (See example in Figure 2).

**Figure 3:**
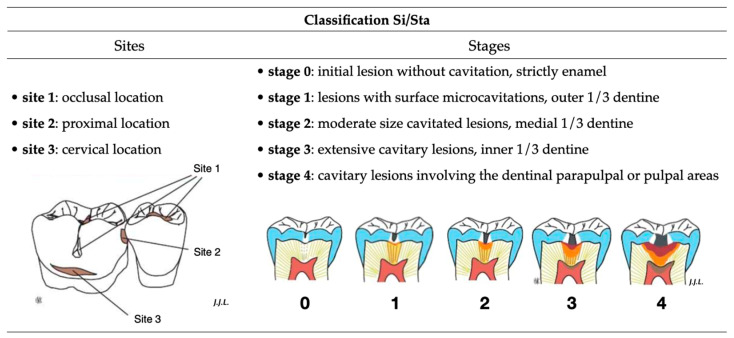
Si/Sta classification describing the different locations (site) and severity (stage) of carious lesions (adapted from [51]).

The carious lesion may be more or less severe, and the tooth may present two types of condition: (i) inflammatory, if the decay is in an early stage of development and the bacterial contamination has not reached the pulp; (ii) infectious, if the decay has reached a significant stage and has led to pulp necrosis. The transition from one stage (i) to the next (ii) can sometimes only be hypothetical on an archeological sample. However, the stage and location can be determined macroscopically and radiographically. Figure 3 shows the Si/Sta classification [51], which was used in the present study. It relies on the description of two variables to characterize the carious lesions: the site (‘Si”, location) and the stage (“Sta”, degree of severity) of the lesion.

**Figure 4:**
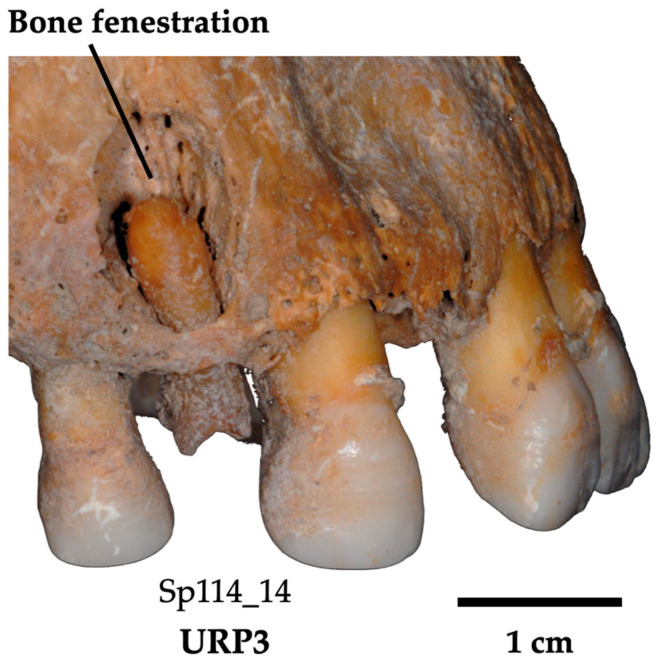
Bone fenestration in the upper left sector of individual Sp114. The destruction of the bone in the periapical area of this URP3 is caused by an infection (i.e., dental periapical abscess) due to a carious lesion with pulp exposure.

Pulp exposure will also condition cementum apposition [52]. It can be due either to a major carious process destroying the crown, or to severe wear reaching the roof of the pulp cavity. It is rated according to whether there is exposure (scored as ‘1’) or not (‘0’), and by “W” for wear, “C” for carious lesion or “M” for mixed to notify the cause of exposure. Apical periodontal disease is an infectious and inflammatory lesion of the periodontium. It is mainly found in the periapical area [53]. An advanced stage of the lesion results in a dental abscess, which often develops into pulp exposure due to severe caries. According to Pinheiro [54], this condition accounts for 15 to 25 % of hypercementosis. In fossil specimens, the presence of an *ante-mortem* abscess can be determined by resorption and fenestration (scored absent (0) or present (1)) of the peri-apical bone (Figure 4).

Impaction is the inability of a tooth to erupt fully and normally into the oral cavity. An impacted tooth remains trapped into the jawbone or retained against another tooth below the gum line [55]. To assess whether impaction and hypercementosis might be correlated, the presence of impacted teeth was systematically scored (impaction: 1; 0 otherwise).

Hypercementosis was visually scored according to type, stage, and extent (see scoring system in [29]). In addition, here, the associated osteological context was also scored as follows. The alveolar bone and neighboring teeth surrounding a tooth of interest may contain essential information to trace back the evolution of the teeth during an individual’s life. This osteological context may, as well, enable to infer the supposed etiology causing hypercementosis. It is necessary to take into consideration the fact that in archeological specimens, some pieces of information, such as calculus accumulation or the presence of antagonistic bone with teeth, can be lost over time due to the process of fossilization (i.e., taphonomic history of the specimen). Wherever possible, each maxilla and mandible was inspected, and the following three features were scored. First of all, the periodontal status may be ascertained by scoring the presence of a significant amount of dental calculus as an indicator of possible periodontal disease (0: absence; 1: presence), and of an *ante-mortem* abscess which results in the resorption or the fenestration of the peri-apical bone (0: absence; 1: presence). Second, the *ante-mortem* tooth loss of neighboring teeth may be identified when a tooth is surrounded by one or several empty bone sockets following the loss of adjacent teeth during life; the remaining tooth would thus theoretically be subjected to mechanical stress higher than in the case of an intact dentition in which mechanical loads are more evenly distributed. This excessive stress can lead to the development of hypercementosis [56]. The observation of missing neighboring teeth around an hypercementotic tooth of interest was scored so as to describe their anatomical location (0: tooth with no mesial and/or distal tooth loss; 1m: tooth with mesial tooth loss; 1d: tooth with distal tooth loss; 2: tooth loss on both sides). Third and last, the presence of antagonistic teeth was scored (1: presence; 2: *ante-mortem* loss) to discuss the possibility of super-eruption of a hypercementotic tooth and/or the potential absence of masticatory stresses associated with this condition.

#### 2.2.2. X-ray Microtomography

The teeth were scanned using the micro-CT scanner based at PLACAMAT (UAR 3626 CNRS, University of Bordeaux, France; equipment: GE phoenix^®^ V/TOME/SX X-ray microtomograph, USA). Each tooth was individually wrapped in soft tissue and several of them were then positioned side by side and rolled in soft tissue altogether. This bundle of samples was then placed in a plastic tube. The following parameters were used: voltage 100 kV, intensity 200 μA, 0.1 mm copper (Cu) filter, 2550 projections with 3 frames averaging over 360°. The final volume was reconstructed with the phoenix datos|×2 rec program in 16-bit format with an isotropic voxel size of 19.00 μm. The reconstruction software includes a ring artefact correction; no further image correction was used. The reconstructed data were opened in Avizo^®^ 7.0.1 software (Visualization Sciences Group, FEI Corp., Hillsboro, OR, USA) and saved as an .am file. Since each scan contains between five and seven teeth, each tooth was isolated using the cropping tool in the segmentation editor.

For each tooth, the dental tissues were then segmented following two steps and by a single author, adhering to a consistent protocol that was controlled and validated by the same author and another one: (i) a semi-automatic segmentation using the marker-controlled watershed algorithm available in Avizo (based on [57]) was first carried out to discriminate the dental features with the strongest contrast (i.e., enamel, dentine and pulp cavity). Yet this does not enable one to distinguish cementum from dentine due to their similar density and grey values. (ii) The distinction between cementum and dentine was achieved by manually editing this preliminary segmentation mask.

The cementum and dentine surfaces, CementumSurf and DentineSurf, were separately extracted with the GenerateSurface module in Avizo. Cementum 3D thickness maps were computed with the SurfaceDistance module, performing a pointwise distance calculation from CementumSurf to DentineSurf. The cementum thickness values were represented on a 3D map using a colormap called “temperature”, which is a linear scale. By employing a perceptually uniform sequence of colors, we ensured that the chosen colormap is both sequential and linear, thereby preserving the integrity of our results [58,59]. The color-coded cementum map was then overlaid onto the dentine surface (see Figure 5 in [30] for an example).

**Figure 5:**
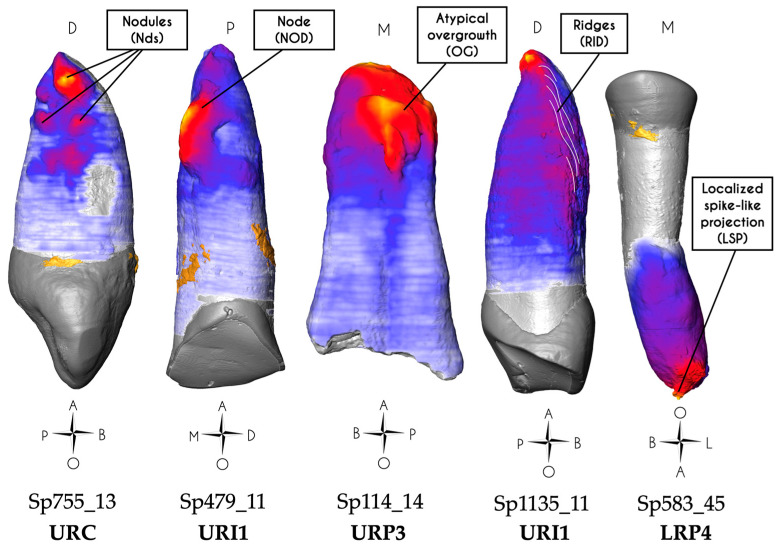
Three-dimensional thickness maps showing the five types of cementum apposition (from left to right): nodules (e.g., Sp755_13), node (e.g., Sp479_11), atypical overgrowth (e.g., Sp114_14), ridges (highlighted using thin undulating white lines; e.g., Sp1135_11) and localized spike-like projection (e.g., Sp583_45). Abbreviations stand as follows: O = occlusal; P = palatal; L = lingual; B = buccal; M = mesial; D = distal; A = apical. The orientation of the tooth Sp583_45 in the figure takes into account its rotated position within its bony socket with its actual buccal surface facing mesially (See Supplementary Information S1).

For each tooth, four parameters were then measured: (i) the maximum cementum thickness (MAX THI, in µm); (ii) the location and the side of the root where the cementum thickness was the greatest (LOC MAX; location was scored as 1 for the apical third, 2 for the middle third, and 3 for the cervical root third; and in the sagittal direction: ‘<’ stands for the buccal side while ‘>’ stands for lingual; in the frontal direction: ‘m’ stands for mesial while ‘d’ stands for distal); (iii) the side where the cementum thickness was the smallest (LOC MIN; if no face could be identified, the mention “no” was used); and (iv) the pattern of apposition: preferential or non-preferential (PREF LOC; noted ‘yes’ for preferential and ‘no’ for non-preferential), where a cementum apposition is qualified as preferential if its distribution is not homogeneous around the long axis of the tooth root. When the apposition took an atypical appearance, it could take five different forms (Figure 5): a node (NOD) is a single and well-defined protuberance of cementum, greater than 2 mm, and which may resemble a hemisphere or be elongated. A nodule (Nds) is a well-defined cementum protuberance, less than 2 mm with an approximately hemispherical form, which systematically occurs in clusters. An atypical overgrowth (OG) is a very irregular and bulky apposition covering the root on two sides or more, and is greater than 3 mm in diameter. Ridges (RID) depict an apposition covering a surface wider than 4 mm, which can be compared to a mountain range. The cementum deposition represents a succession of elongated furrows and bumps (size of one ridge: width < 1 mm; length > 2 mm; height > 0.5 mm). Ridges lie obliquely to the long axis of the tooth root. A localized spike-like projection (LSP) is defined as a single small apposition forming a sphere of cementum with spikes, smaller than 2 mm.

#### 2.2.3. Confocal Microscopy

Each tooth was scanned in scanning confocal microscopy in order to characterize the surface texture of cementum apposition at greater resolution than microtomography. The microscope (S-Neox 3D optical Profiler, Sensofar^®^ metrology, Barcelona, Spain) available at the PACEA laboratory (UMR 5199, Pessac, France) uses microdisplay scanning technology and has four objectives: ×5, ×20, ×50 and ×100. Confocal microscopy produces height maps that can be analyzed with a variety of descriptors for texture or roughness analysis. Each element of this microdisplay corresponds to a pixel on the color photographic sensor acting as a detector. The light source on this type of microscope is a monochromatic light-emitting diode in blue, green, red or white, each with a specific wavelength distribution [60]. The ×5 objective was used to visualize and edit a preview of the area of interest (overview of the tooth). The fine characterization was carried out with the ×20 objective in confocal fusion mode [61] with the following parameters: led color: blue (460 nm); gain (local variation in the sensitivity over the sensor area): 1.5; gamma (contrast factor can be described as the smoothness with which the image changes from black to white on a digital display): 1.7; factor (relates to the noise in an image; the noise is low if the factor is low, e.g., factor = 1): 2; and overlapping (limits of the registered fields of view straddling each other to avoid loss of information during the computation of the final 3D image): 10%. For each tooth and each acquisition session, all parameters were carefully recorded in an Excel sheet. The resulting surfaces had a lateral (x, y) spatial resolution of 0.65 µm/pixel. The areas of interest were chosen based on the 3D thickness maps produced from the microtomographic scans. For each tooth, and to standardize the protocol, a measurement was made in the area of the root where the cementum thickness was the greatest (LOC MAX). Other regions of interest that presented an atypical visual aspect were sometimes recorded (i.e., areas with cementum ridges, different faces of impacted teeth).

**Figure 6:**
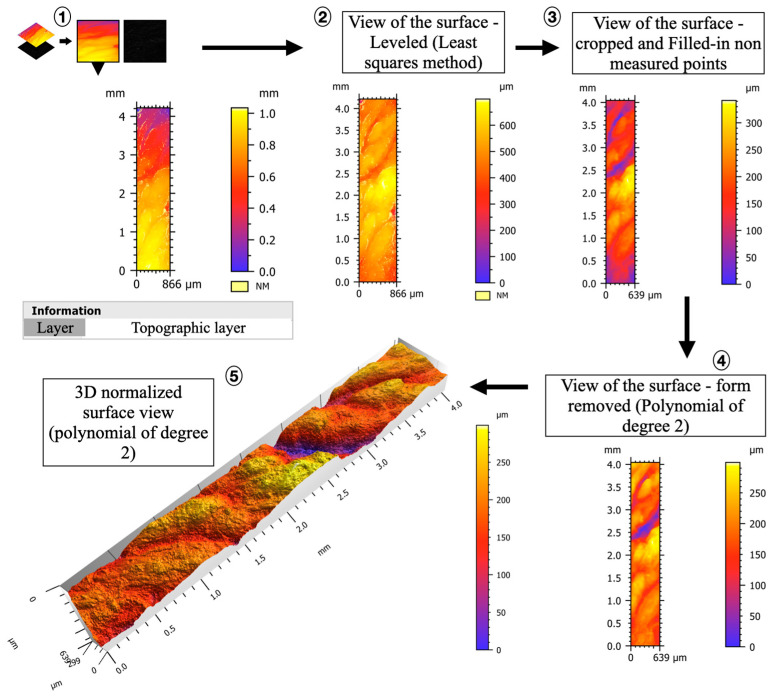
Workflow used to extract 3D surface topography (SensoMap software) from a cementum surface. Step 1: the topographic layer is extracted and processed in the SensoMap software; step 2: the surface is leveled; step 3: the surface is cropped and non-measured points (NM) are filled in; step 4: the form (curvature related to root shape) is removed by applying a polynomial of degree 2, thus straightening the 3D map; step 5: the final 3D topography can be generated.

Once the region of interest was fixed in x and y, the z-axis range was set in brightfield mode by determining the lowest and highest elevation points. In confocal mode, the light intensity is set before the beginning of the acquisition. The recorded topographic surface was considered as a raw surface that needed to be treated before extracting the 2D and 3D profiles using the SensoMap working software (version 8.2, Sensofar Group, Barcelona, Spain). SensoMap is a tool based on Digital Surf’s Mountains technology, which is a surface analysis platform. It enables advanced analysis of surface data and generates customized reports, providing detailed insights into the characteristics of cementum surfaces. In a first step, the missing or unmeasured points were estimated using a specific algorithm (interpolation from surrounding points). A series of processing steps were recorded in a saved workflow (=template), which was applied the same way for each of the processed topographies. (i) Data acquisition—the process begins by capturing a layer of data from SensoScan, providing us with the initial 3D representation of the cementum surface. (ii) Surface levelling—in this stage, the acquired data are transferred to the SensoMap software (version 8.2, Sensor Group, Barcelona, Spain). The first task is to ensure that the surface is level. This is a crucial step to correct any irregularities in the original data, ensuring accuracy in subsequent analyses. (iii) Cropping and data enhancement—to focus our analysis on the relevant area, we cropped the data as needed. Additionally, any regions with non-measured points (NM) were filled in. This enhancement is vital for a comprehensive 3D model. (iv) Curvature removal—the cementum surface may have variations caused by the curvature related to the underlying root shape. To mitigate this, we applied a polynomial of degree 2, effectively removing these curvatures. This step resulted in a straightened 3D map, allowing for a clearer and more precise analysis. (v) Final 3D topography—once the curvature of the underlying dentine root surface was accounted for, we obtained the final 3D topography of the cementum surface. These data provide a detailed representation of the surface (Figure 6).

**Figure 7:**
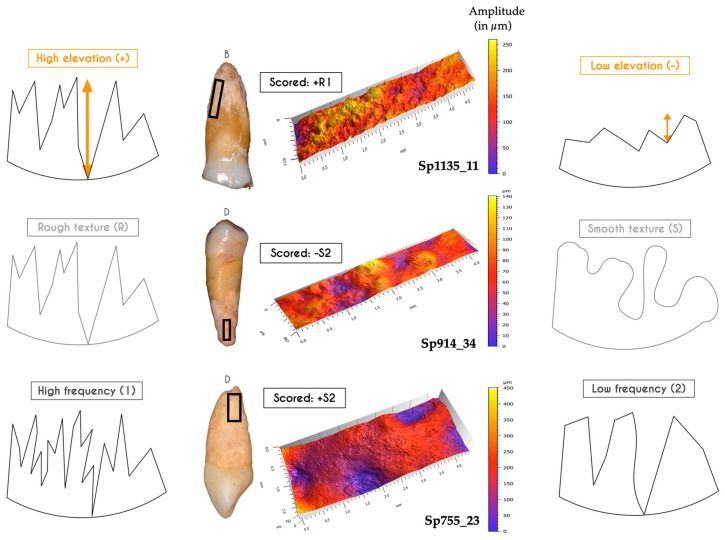
Surface aspect scoring system with examples from the study sample in the center. The sign (+ or −) represents the elevation, the letter (R or S) represents the texture, and the number (1 or 2) represents the frequency of relief. The schematized profiles of the cementum surfaces have been exaggerated for a better understanding of each of the studied variables. B = Buccal; D = Distal.

Once the 3D topography of the cementum surfaces was generated and post-processed, two parameters were collected: (i) the value of maximum vertical elevation (MAX VE, expressed in µm) and (ii) the surface aspect. The evaluation of the surface aspect took into account three variables, based on qualitative visual inspection (see Figure 7 for guidelines): the vertical elevation: scored (+) if it was high (i.e., amplitude ≥ 200 µm), or (−) if it was low (i.e., amplitude < 200 µm); the surface texture (ST): scored (S) for smooth or (R) for rough; the frequency of occurrence of the elevations (i.e., if the positive reliefs were close to each other), scored (1) if the frequency was high, or (2) if the frequency was low (Figure 7).

### 2.3. Statistical Analyses

The statistical analyses of the present study will enable (i) to identify trends, patterns, and concealed relationships among the variables we investigated; (ii) to assess the relevance of the groups we defined based on the observations and on the collection of qualitative and quantitative data; (iii) to identify the most influential factors among the measured features, thus providing insights into the underlying processes at play; and (iv) to bolster our interpretations and strengthen the reliability of our conclusions by minimizing potential biases.

The link between the etiologies and each explanatory variable was first investigated using appropriate graphical representations, depending on the type of the variable—violin plots and stripcharts for continuous variables, and barplots for ordinal and qualitative traits. Then, a Factor Analysis of Mixed Data, or FAMD [62], was performed on a subset of explanatory variables. This unsupervised (i.e., without a priori attribution of each tooth to an etiology) multivariate analysis aims to explore their relative strength and to identify and characterize their significant relationships. FAMD is a factorial method allowing for the analysis of datasets containing both continuous (i.e., quantitative) and categorical (i.e., qualitative) variables. It can thus be considered as a combination of Principal Component Analysis and Multiple Correspondence Analysis. The qualitative variables showing no variability (always absent or always present) or too little variability (only one presence or only one absence) were discarded upfront. In addition, an iterative algorithm for missing data imputation was applied to fill in the rare instances of missing values [63], for instance, due to the *ante-mortem* loss of the tooth crown in case of a carious lesion in an infected tooth. Biplots of specimens and factor levels were represented for the first three principal axes, and convex hulls were then drawn for each etiology group. In the end, FAMD enabled an efficient visualization of potential relationships among variables and associated groups, providing an overall perspective on how various dental features combine and distinguish themselves in comparison to the presumed etiologies.

Finally, a classification tree [64] was built to explain the etiology groups. A decision tree aims to create classes of specimens (“terminal leaves”) using a combination of dichotomous splits on explanatory variables (the “nodes” of the tree), in such a way that the classes are optimally homogeneous relative to the etiologies. The algorithm utilizes all the quantitative and qualitative data collected on the sample to best classify the teeth in the etiology groups, based on thresholds of key features that will discriminate them from other groups (i.e., numerical threshold for a quantitative variable or the state ‘presence’ or ‘absence’ for an ordinal variable). A terminal group is considered as “pure” when specimens from only one etiological group have clustered together. Additional technical details about the criteria retained to grow the tree are available in Supplementary Information S2.

All analyses were performed using R 4.3.2 [65]. The entire R code written for this study, along with the description of the R packages used, is available online in Supplementary Information S2.

## 3. Results

From the micro-CT and confocal microscopy data of the archeological teeth investigated, several groups of cementum apposition patterns could be distinguished based on the 2D and 3D characterization of the dental tissues. All the results are summarized in Table 2. The macroscopic data and the set of 3D maps are presented in Supplementary Information S1.

### 3.1. Impacted Teeth

Impaction is the inability of a tooth to fully and normally erupt into the oral cavity due to various factors [66]. An impacted tooth does not fully emerge at the surface of the dental arch and is instead trapped within the jawbone. This condition can occur in both the maxillary and mandibular regions [55]. Two in situ maxillary contralateral canines (Supplementary Information S1, pp. 1–8) belonging to the same individual (Sp755) were completely retained in their bone sockets and therefore not worn (occlusal wear scored DEG: 1; DIR: 1; FOR: 1 [50].

#### 3.1.1. Visual Examination

**Figure 8:**
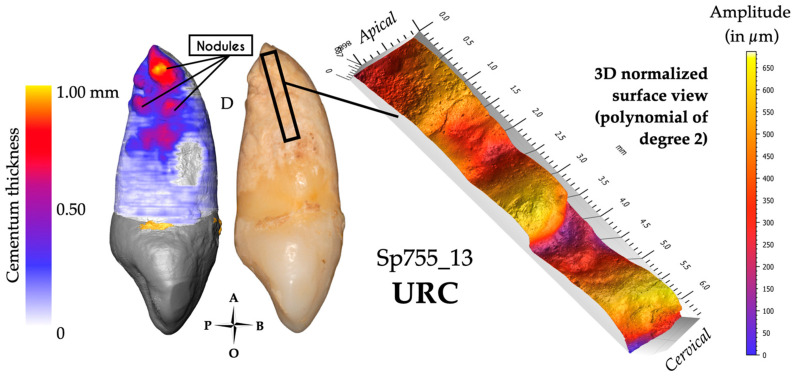
Results for specimen Sp755_13. The 3D thickness map (left), photograph (center) and 3D topography (right) of a region of interest (black frame on the photograph) show the very undulating surface of this atypical apposition displaying the maximum cementum thickness on this tooth (nodules). For this group, hypercementosis was scored 3.3.m, with an average of 1035 µm of the maximum cementum thickness. This surface was scored +S2 (typical profile of these two impacted teeth).

Both canines showed a mixed form of hypercementosis, which combines a significant diffuse hypercementosis covering the whole root from the apical third to the cervical root third, and a particular focal apposition. In Sp755_13, this mixed hypercementosis was scored as 3.3.m (type 3: diffuse and local type; stage 3: covering all of the three root thirds; m: moderate form), which means that this tooth displays a focal hypercementosis with a nodular form, extending from the apical to the medial root third (Figure 8). In Sp755_23, the mixed hypercementosis was also scored as 3.3.m; the focal apposition is limited to the apical third and is similar to a node form.

#### 3.1.2. Microtomography and 3D Thickness Maps

Both teeth are affected by hypercementosis covering the whole surface of their root, from cervix to apex, with a preferential apposition of cementum on the distal side of the root, with the maximum cementum thickness (*n*= 2; Sp755_13: 1000 μm; Sp755_23: 1070 μm) occurring under the form of nodes or nodules (Figure 8). For both canines, the thinnest cementum apposition was found on the buccal aspect of their root.

#### 3.1.3. Confocal Microscopy and 3D Topography Maps

For each tooth, two acquisitions were made: one focusing on the most important cementum apposition (distal side) and a second on the opposite (mesial) side. These canines show the same surface condition according to the observed face (distal side scored +S2 (Figure 8); mesial side scored -R2). Note that Sp755_13 shows the highest vertical elevation of the whole study sample (690 μm), higher than that of Sp755_23 (450 μm).

### 3.2. Infected Teeth

This group concerns four teeth with no preserved crown (i.e., Sp114_14, Sp335_23, Sp914_43, and Sp1172_15; Supplementary Information S1 pp. 9–11, 12–14 and 18–21, respectively; Figure 8). Several factors may have induced tooth crown loss, such as a major carious process destroying the crown, or severe wear reaching the roof of the pulp cavity. These two processes may also be combined. Most of the time, when the crown breaks off, the pulp gets exposed, which is reported to affect cementum apposition [52]. We hypothesize that these teeth were infected because oral bacteria passed through the exposed pulp. At an advanced stage of infection, this may be expressed as a dental abscess [53].

#### 3.2.1. Visual Examination

**Figure 9:**
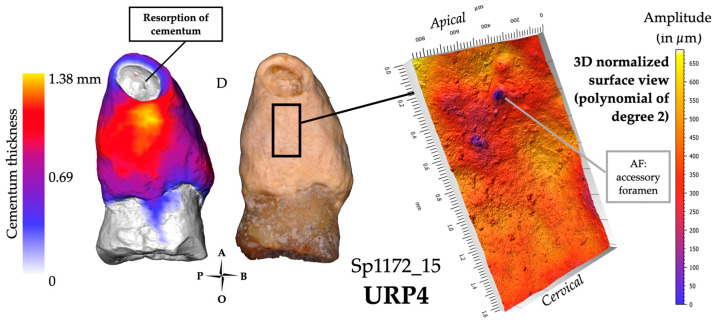
Results for specimen Sp1172_15, an example of the infected teeth profile. The 3D thickness map (left), photograph (center) and 3D topography of an area of interest (black frame on the photograph; right). For this group, the significant extent of the hypercementosis was mainly scored 1.4.m and the average of the maximum cementum thickness was 1740 µm.

In archeological dental specimens, ascertaining tooth infection may be determined by the observation of resorption and fenestration of the alveolar bone. Since the integrity of the cemento-enamel junction may be affected when the crown breaks off, neither wear nor the condition of the cervical root third could be safely scored in these teeth. Occlusal wear was therefore scored as “NA”. These teeth were assigned the highest carious stage because of the substantial damage only leaving a partial tooth root (Si/Sta classification: 1/4). Two teeth out of four (i.e., Sp114_14 and Sp335_23) showed fenestration in the bone socket, indicating a severe apical abscess. Hypercementosis was scored as stage 4 for all teeth. Three of these teeth have a diffuse form of hypercementosis (Sp_335_23, Sp914_43, and Sp1172_15; Figure 9) and three have the mixed form of Sp114_14. The form of hypercementosis is marked for Sp114_14, Sp335_23, and, Sp1172_15 (Figure 9) and moderate for Sp914_43.

#### 3.2.2. Microtomography and 3D Thickness Maps

Overall, in this group of infected teeth, the maximum cementum thickness is on average 1740 μm (*n* = 4; range: 1380–2160 μm, SD: 375 µm) and the apposition is non-preferential. Two teeth (Sp335_23 and Sp1172_15) show traces of cementum resorption at the root apex (Figure 9). Sp114_14 shows a particular aspect of its dentine on the apical root third in the 2D micro-CT slices, suggesting resorbed dentine had been replaced by the secretion of compensatory cementum (Supplementary Information S1 pp. 9–11). In the same tooth, the cementum shows an atypical overgrowth (see Methods for a definition). Except for Sp1172_15, the external aspect of the cementum in these infected teeth may suggest a continuous remodeling of this tissue (Figure 9).

#### 3.2.3. Confocal Microscopy and 3D Topography Maps

On average, the vertical elevation of cementum reaches 320 μm in these four infected teeth, with the lowest value at 110 μm (Sp1172_15) and the highest value at 475 μm (Sp114_14). Overall, the 3D topography of the cementum on these teeth shows a high amplitude and a smooth surface. The tooth Sp1172_15 is different in having a low vertical elevation with a relief of small amplitude, and its surface texture is smooth both distally and mesially (Figure 9). Smaller holes are visible on both surfaces, which may be interpreted as accessory foramina. This group has a high cementum thickness, and surface specificities (e.g., resorption, remodeling marker) that may suggest compensatory apposition in response to chronic infection.

### 3.3. Hypofunctional Teeth

The third group concerned hypofunctional teeth, which involves teeth with a minimal degree of wear (score 2 or 3 after [50]) or which had lost their antagonist teeth *ante-mortem*. This group comprised six teeth. The micro-CT scans revealed that Sp1230_44 had two coalescent roots hidden under the cementum; therefore, this tooth was excluded from this sample of single-rooted teeth.

#### 3.3.1. Visual Examination

Two teeth (Sp20_35 and Sp914_34) are slightly worn (score: 2; Figure 10), while the three others (Sp17_15, Sp173_45, and Sp1230_34) display a slightly more advanced stage of wear (score: 3). Only one tooth (Sp1230_34) presents some dental calculus on the root surface just below the cervical line. All specimens preserved their adjacent teeth, except for Sp17_15, which lacks its distal neighboring tooth. Sp173_45 does not have any antagonistic teeth and appears super-erupted (i.e., continued eruption of the tooth beyond the occlusal plane), which caught our attention. All the teeth have hypercementosis scored as 1.2.m.

#### 3.3.2. Microtomography and 3D Thickness Maps

**Figure 10:**
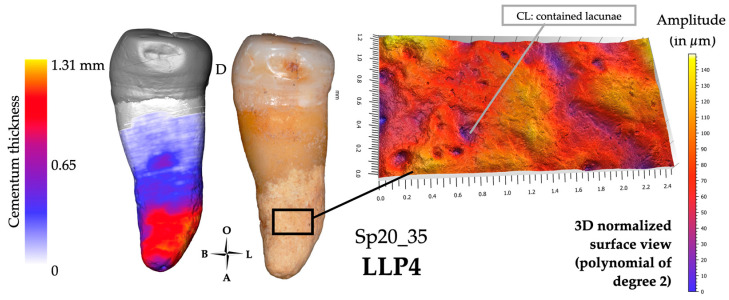
Results for individual Sp20_35, which may represent the group of hypofunctional teeth. The 3D thickness map (left), photograph (center) and 3D topography of a region of interest (black frame on the photograph; right) show the distribution of cementum, which is not preferential. For this group, hypercementosis was scored as 1.2.m or M, with an average maximum cementum thickness of 1410 µm.

For this group of hypofunctional teeth, the maximum cementum thickness reached an average of 1410 μm (*n* = 5; range: 1240–1640 μm, SD: 154 µm), with no consistent location on a preferential side of the tooth. The apposition is thus non-preferential, and the occurrences of maximum thickness of cementum are not restricted to a delimited area of the root surface (Figure 10).

#### 3.3.3. Confocal Microscopy and 3D Topography Maps

The vertical elevation of the cementum has an average of 150 μm, with the lowest value at 110 μm (Sp1230_34) and the highest value at 180 μm (Sp20_35; Figure 10). For Sp17_15, the data were acquired in an area of cementum fracture, so it was not considered in the group average. Most of the tooth roots show flat relief textures, with a low amplitude and a low frequency pattern. However, two teeth show a different cementum configuration: Sp173_45 is a super-erupted tooth with a root surface of relatively low amplitude, and smooth texture, but with numerous small-diameter depressions (i.e., 0.02–0.1 mm). The mesial side of the second tooth, Sp20_35, shows the typical surface of this group (i.e., scored -S2), yet with several small depressions (i.e., 0.01–0.03 mm) with an irregular outline, which we interpret as lacunae of resorption (Figure 10). The mesial side of the Sp20_35 root shows a stronger amplitude with larger and better-defined lacunae (i.e., 0.05–0.2 mm) with sharp borders, which could result from fibrous insertions.

### 3.4. Hyperfunctional Teeth

**Figure 11:**
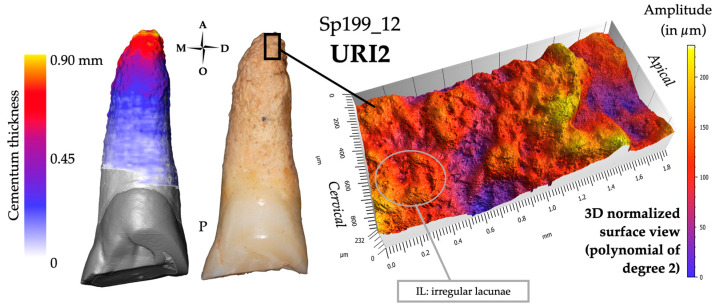
Results for individual Sp199_12, which may represent the group of hyperfunctional teeth. The 3D thickness map (left), photograph (center) and 3D topography of a region of interest black frame on the photograph; right) show the distribution of cementum, which is preferential for this group with an apical and lingual location. The average of the maximum cementum thickness is 1205 µm. The surface profiles have mainly a rough surface texture, scored R.

The last group comprises 12 teeth described as hyperfunctional because their wear is either substantially pronounced (e.g., DEG: 3–8) or shows an atypical aspect, such as spiral grooves (e.g., Sp591_12), oblique wear facets (e.g., Sp471_11), or other unusual configurations that do not correspond to regular wear patterns (e.g., Sp876_35). We consider these teeth to have been subjected to significant mechanical loads. Specimen Sp1010_44 was excluded from the sample following the inspection of the micro-CT data, which revealed that this tooth actually had two roots concealed by the overlaying cementum (similar to the case of Sp1230_44 mentioned above). Note that all the maxillary incisors of our sample were classified in this group (Figure 11).

#### 3.4.1. Visual Examination

Most cases of cementum apposition are concentrated in the apical root third, but visual examination of hypercementosis does not give a consensus of classification [29]. One tooth was scored 1.1.m (i.e., Sp591_12), five teeth were scored 1.2.m (i.e., Sp39_35, Sp199_11, Sp335_13, Sp876_35, and Sp914_33), three teeth were scored 1.3.m (i.e., Sp199_12, Sp810_13, and Sp1135_11; Figure 11), three teeth have a mixed profile of hypercementosis and were scored 3.2, two with marked hypercementosis were scored “M” with node-like apposition (i.e., Sp479_11 and Sp1172_24) and one with moderate hypercementosis was scored “m” with nodular apposition (i.e., Sp810_23).

#### 3.4.2. Microtomography and 3D Thickness Maps

The maximum cementum thickness is on average 1205 μm (*n* = 12; range: 740—1970 μm, SD: 349 µm). The apposition is preferential, with an apico-lingual location for all maxillary incisors (Figure 11). In addition, 10 out of the 12 teeth have their smallest cementum apposition on the buccal surface of their root. Four teeth show specific shapes of cementum apposition: one nodule-like (Sp810_23), two node-like apposition (Sp479_11 and Sp1172_24), and a single tooth (Sp1135_11) displaying a ridge-like apposition on its buccal side. These types of hypercementosis are found on teeth with a high degree of wear (scores 6 and 7).

#### 3.4.3. Confocal Microscopy and 3D Topography Maps

The vertical elevation of the cementum has an average of 300 μm, with the highest value at 665 μm in Sp479_11 with a characteristic node-shaped apposition. The data acquisition for Sp591_12 was performed in an area of cementum fracture, so it was not considered in the group average.

Among the 12 teeth of the hyperfunctional group, three profiles look similar and have already been encountered in the impacted group (i.e., Sp755_13 and Sp755_23). They are characterized by the nodule and node apposition types. The surface condition was scored +S2, indicating a surface with high amplitude, a smooth aspect with low frequency of patterns (e.g., Sp491_11 and Sp810_23). The other profiles have a rough surface texture, scored R (Figure 11).

After defining these groups and their specific patterns of cementum apposition (Figure 8, Figure 9, Figure 10 and Figure 11), it appeared that several teeth displayed mixed features. Indeed, they could classify in several groups at the same time, which justified the establishment of a last group called “mixed condition”.

### 3.5. Mixed Condition

Ten teeth showed a mixed apposition profile and are characterized by the combined presence of severe wear (score > 5) and inflammatory or infectious phenomena. Six teeth present a degree of wear greater than or equal to 6 (i.e., Sp583_45, Sp666_33, Sp709_31, Sp884_34, Sp884_35, and Sp1300_14). Six teeth have a carious lesion (i.e., Sp666_33, Sp735_45, Sp876_44, Sp876_45, Sp914_35, and Sp1300_14). Sp666_33 shows a specific combination of node-type apposition, heavy wear (score: 7), a carious lesion (score: 2/2) and a calculus deposit (Supplementary Information S1, pp. 82–84). Two cases of localized spike-like projection are also present in this group (Sp583_45 and Sp709_31). These projections are positioned in the most apical part of the root, at the tip of the apex. They could be related to the fusion of the bone and cementum resulting in an ankylosis of the tooth onto the floor of the bony socket. These same teeth show calculus deposits, especially noticeable on Sp709_31. Six out of the ten teeth, among which was Sp583_45, have no antagonist. In our overall sample, there were only eight teeth without antagonists. Two teeth (Sp884_34 and Sp884_35) attributed to this group “mixed condition”, and without antagonist belong to the same individual and show a very advanced wear stage (score: 8), which even resulted in pulp exposure in Sp884_35. In this tooth, cementum apposition takes the form of an atypical overgrowth (similar to Sp114_14, classified as an infected tooth).

Furthermore, specimens Sp583_45, Sp666_33, and Sp1300_14 (See Supplementary Information S1, pp. 79; pp. 82; pp. 107) have a significant number of missing teeth, suggesting poor oral health. All the studied teeth in these specimens were classified as ‘mixed’, likely indicating that the teeth of these individuals were subjected to various conditions successively or concurrently (e.g., wear, carious lesions, periodontal disease), ultimately resulting in a near-total loss of their dentition.

The maximum cementum thickness in this “mixed condition” group reached 1625 μm on average (N = 10; range: 1100—2770 μm; SD: 529 µm), and is found on the distal side of the root for half of this group. The highest values were found in Sp583_45 at 2770 μm with a localized spike-like projection, and in Sp666_33 at 2350 μm with a node-like apposition (Supplementary Information S1, pp. 83). It can also be noted that, in carious teeth, the side of maximal apposition of cementum coincides with that of the carious lesions. The vertical elevation of cementum has an average value of 330 μm (range: 175—550 μm). The most common surface aspect of this group scores +R2. The tooth with node-like apposition (Sp666_33) also looks like impacted teeth or hyperfunctional teeth, which have the same focal hypercementosis profile (i.e., Sp479_11, Sp755_23). Its surface condition was noted as +S2.

Our results, thus, revealed different apposition patterns with several surface topographies that can be correlated with supposed etiological contexts (Figure 8, Figure 9, Figure 10 and Figure 11).

### 3.6. Statistical Analyses

From the qualitative and quantitative data collected above, we assessed and identified statistically meaningful links between the various analyzed variables, and to test *a posteriori* the potential statistical definition of the five groups of supposed etiologies (i.e., impaction, infection, hypofunction, hyperfunction, and the mixed condition). Detailed results are presented in Supplementary Information S2.

**Figure 12:**
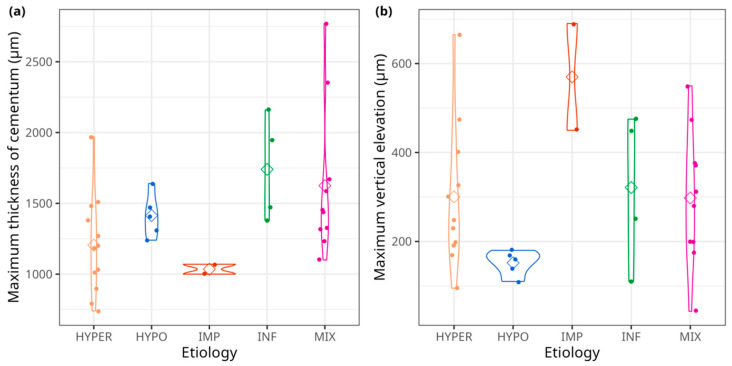
Individual specimen values and mean values of (**a**) maximum thickness of cementum (MAX_THI, in µm) and (**b**) maximum vertical elevation of cementum (MAX_MICRO, in µm) by etiology in the Sains-en-Gohelle sample. Mean values for each etiology are symbolized by diamonds. HYPER = hyperfunctional teeth; HYPO = hypofunctional teeth; IMP = impacted teeth; INF = infected teeth; MIX = mixed condition.

The violin plots (Figure 12) revealed that two variables, the maximum thickness of cementum (MAX_THI; in µm) and the maximum vertical elevation of cementum (MAX_MICRO; in µm) allow for the clear distinction between impacted teeth (IMP) and hypofunctional teeth (HYPO) from the rest of the groups. Both IMP and HYPO exhibited narrower ranges of MAX_THI, with mean values approaching 1000 µm for impacted teeth and around 1500 µm for hypofunctional teeth. In contrast, the other groups (i.e., the hyperfunctional (HYPER), infected (INF), and mixed (MIX) groups) displayed a much broader range of variation, spanning from approximately 200 to 3000 µm of maximum cementum thickness, with a considerable overlap. Concerning MAX_MICRO, the two IMP teeth showed again a narrower variability and high elevations around 600 µm, while hypofunctional teeth exhibited a more restricted variability around 170 µm, albeit in the lower range compared to other groups (approximatively 300 µm). The hyperfunctional, infected, and mixed groups presented entirely overlapping vertical elevation distributions, with mean values around 300 µm.

**Figure 13:**
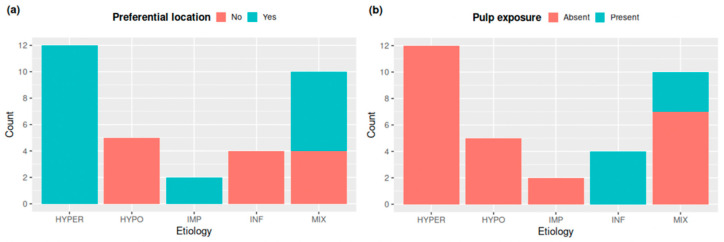
Barplots for (**a**) Preferential location and (**b**) Pulp exposure, depending on etiology. “Count” is for sample size and scores are as follows: 0 = absence or 1 = presence. HYPER = hyperfunctional teeth; HYPO = hypofunctional teeth; IMP = impacted teeth; INF = infected teeth; MIX = mixed condition.

The barplots (Figure 13) highlight two main determining variables in the clear separation of groups. A preferential location of cementum apposition was only found in the HYPER, IMP, and MIX groups. In contrast, pulp exposure was observed only in the INF and MIX groups.

**Figure 14:**
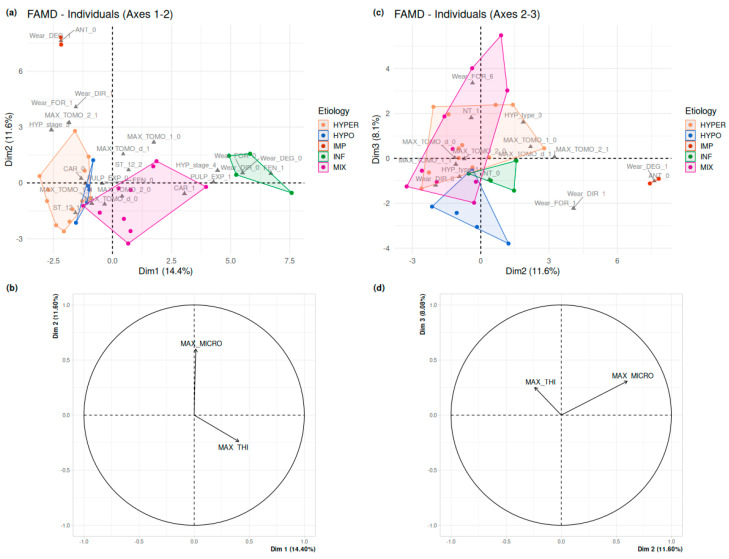
Factor Analysis of Mixed Data (FAMD) of the dataset. (**a**,**b**) Results for the first two principal axes; only those factor levels reaching a quality of representation (cos^2^) greater than 0.56 are represented on (**a**). (**c**,**d**) Results for the principal axes 2 and 3; only those factor levels reaching a quality of representation (cos^2^) greater than 0.45 are represented on (**c**). See Supplementary Information S2 for details. HYPER = hyperfunctional teeth; HYPO = hypofunctional teeth; IMP = impacted teeth; INF = infected teeth; MIX = mixed condition.

Following these univariate analyses, a Factor Analysis of Mixed Data (FAMD, [62]) was performed. In this analysis, two continuous variables, three ordinal traits related to dental wear, and a subset of qualitative traits were considered. Some non-polymorphic traits, meaning those that were consistently equal to 0 or 1 across the entire sample, and a trait observed in only one individual, were excluded from the analysis (Supplementary Information S2). The etiological groups were identified after running the FAMD by displaying convex hulls on Figure 14a,c. Axis 1 explained 14.4% of the total variance, quite similar to Axis 2, which reached 11.6%, while Axis 3 only explained 8.1% of the total variance of the sample.

Axis 1 separated almost completely the teeth identified as HYPER (lower values) from the MIX (higher values). The HYPO teeth tightly clustered with the highest values of the HYPER group. The HYPER and the HYPO teeth are characterized by an absence of carious lesion or pulp exposure (CAR = 0; PULP_EXP = 0), and by a cementum apposition at the apical root third (MAX_TOMO_1 = 1). High values along Axis 2 separated the two IMP teeth well from any other groups, while they all overlapped along Axes 1 and 3. They are characterized by an unworn tooth crown (Wear_DEG_ = 1) and the inability to score the feature “antagonist tooth” (ANT = 0). The INF teeth also represent an isolated cluster, with high values along Axis 1, while there was some overlap with HYPER, HYPO and MIX following Axes 2 and 3. As expected, the INF teeth were characterized not only by the inability to score the three features of wear (Wear_DEG, Wear_FOR, Wear_DIR) on their broken-off crown, but also by the presence of bone fenestration (FEN = 1), of pulp exposure (PULP_EXP = 1) and a partially or fully damaged cemento-enamel junction (HYP_Stage = 4). Axis 3 separated mostly the INF, IMP and HYPO teeth (lower values) from the clusters of HYPER and MIX teeth (higher values). As already shown in the barplots on wear (Figure S3 in Supplementary Information S1), the two later clusters involve the variables attesting of heavy wear stages.

As for continuous variables, the maximum vertical elevation of cementum (MAX_MICRO, in µm) is the most contributive variable, which seems to separate the HYPER and HYPO from the MIX, INF and IMP (Figure 14b,d). The maximum thickness of cementum (MAX_THI, in µm) has a weaker effect and seems to distinguish HYPER and IMP from MIX and INF.

**Figure 15:**
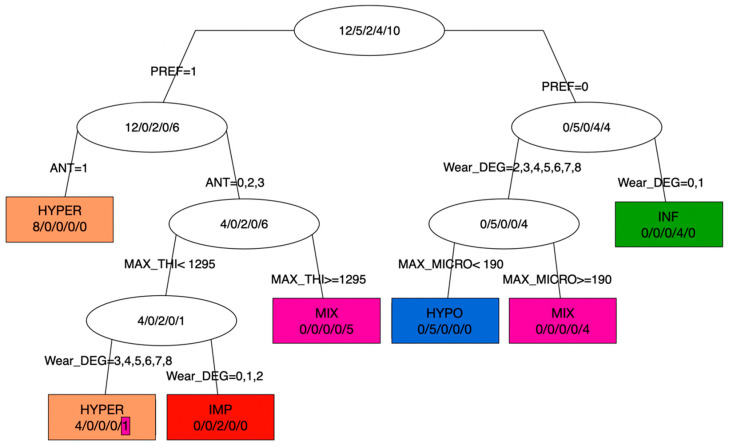
Decision tree for explaining the etiology using all covariates. Terminal leaves are represented as rectangles, while intermediate nodes are represented as ellipses. In each node, the majority class is displayed, along with the number of individuals in the classes HYPER/HYPO/IMP/INF/MIX, respectively. PREF: absence (0) or presence (1) of preferential location of cementum on the root surface; ANT: antagonist teeth (0: not relevant because the tooth of interest is impacted; 1: presence; 2: *ante-mortem* loss; 3: not applicable); MAX-THI: maximum thickness of cementum (in µm), MAX_MICRO: maximum vertical elevation of the cementum surface (in µm); Wear_DEG: wear scores following Molnar et al. [50]. HYPER = hyperfunctional teeth; HYPO = hypofunctional teeth; IMP = impacted teeth; INF = infected teeth; MIX = mixed condition.

The decision tree (Figure 15) exhibits a high level of terminal group purity (six out of seven), indicating that each etiology group was accurately classified based on the measured criteria. The entire sample is contained within the root of the tree (i.e., HYPER: *n* = 12; HYPO: *n* = 5; IMP: *n* = 2; INF: *n* = 4; MIX: *n* = 10).

The first node (i.e., branch bifurcation) is based on preferential cementum apposition (PREF: 0 for absence, 1 for presence), establishing a clear distinction between IMP/HYPER on one side (PREF = 1) and HYPO/INF (PREF = 0) on the other, while teeth from the mixed condition (MIX) are found to be about equally distributed on either side. This initial dichotomy forms the foundation of this classification.

In cases of preferential location of cementum (PREF = 1), and of presence of the antagonist tooth (ANT = 1), the second node distinctly separated eight teeth clustering into a pure group, HYPER. Alternatively, at this second node, when the antagonist tooth was lost *ante-mortem* or could not be scored (ANT = 2, or 0, 3, respectively), the remaining teeth were classified as a group of hyperfunctional (HYPER), impacted (IMP) or mixed condition (MIX) teeth. From the third node, the maximum cementum thickness (MAX_THI in µm) is the key discriminatory variable, with a threshold at 1295 µm. This clearly distinguishes the IMP/HYPER groups which have a MAX_THI inferior to that threshold, while five MIX teeth cluster in a pure terminal group, with a MAX_THI equal to or greater than 1295 µm. From the IMP-HYPER cluster, a fourth node was based on the degree of wear (Wear_DEG: 0: not applicable because crown not preserved; from 1: no wear to 8: heavy wear, where the tooth crown is totally worn away, and the chewing surface is on the root itself), modified after Molnar [50]’s classification. Two teeth cluster in a pure group of impacted teeth (IMP) for a score of 0, 1, or 2. The other terminal group involves teeth with a degree of wear greater than 3, and contains four hyperfunctional teeth and a MIX tooth (Sp735_45).

In the case of the non-preferential apposition of cementum (i.e., PREF = 0), the first node also involves the degree of wear, with a HYPO/MIX cluster on one side (Wear_DEG > 2) and, on the other side, a pure group of INF teeth for which wear could not be scored or was absent (Wear_DEG = 0 or 1). Finally, the HYPO and MIX teeth were distinguished as pure clusters by a threshold in maximum vertical elevation (MAX_MICRO, in µm); all HYPO teeth had a value < 190 µm.

Finally, each step of the classification process contributed to the overall purity of the groups, ensuring the precise identification of different etiologies and highlighting the case of a MIX tooth, Sp735_45, classified as HYPER.

## 4. Discussion

Using microtomography and confocal microscopy, the present work aimed to investigate whether different patterns of hypercementosis could be distinguished, and whether each of these patterns could be related to a specific etiology. The finer understanding of cementum apposition in our archeological sample could contribute to better characterize that of other archeological and fossil human groups, and thus to discuss oral health, and para- and non-masticatory activities.

The Sains-en-Gohelle series was chosen for its substantial number of individuals, for the quality of preservation of the skeletal remains, and particularly for the relatively poor oral health status documented in this sub-actual population (7th–17th century A.D.). d’Incau [46] has reported on the identification and the frequency (30.95%, i.e., 126 out of the 407 individuals studied) of hypercementosis in this series. This frequency seems high in comparison to what Kim et al. [43] documented in a contemporary population (Department of Oral Radiology, School of Dentistry, Kyung Hee University during January 1984 to December 1989), with only 8.2 % within 4236 individuals. A series from Clermont-Ferrand (Puy-de-Dôme, Amadeo street “Centre Hospitalier Sainte-Marie”, France [67]), which dates from a period between the High Middle Ages and the early Classical Middle Ages (4th–10th century A.D.), shows a frequency of 16.83% for 101 individuals [46,67]. Besides these results, which concern sub-actual and present-day populations, Martin-Francés et al. [68] determined a much higher frequency of hypercementosis (61.57%) among a group of 242 teeth from European Middle Pleistocene hominin specimens from Sima de Los Huesos (Sierra de Atapuerca, Spain). Le Cabec et al. [30] reported that almost all of the Neanderthal specimens they investigated showed hypercementosis (*n* = 95 anterior teeth). Other articles report the presence of hypercementosis on teeth from the past, including Neanderthal individuals (e.g., [69,70,71,72]), as well as individuals from earlier (e.g., Plio-Pleistocene, [73]; early Pleistocene, [68]; Late Pleistocene from South Africa, [74]) or more recent times (e.g., 8–3.5 ka B.P., [75]).

Hypercementosis is therefore very common in past populations. Our results revealed four distinct groups of types of cementum apposition in the Sains-en-Gohelle sample, with characteristic profiles of their assumed oral environment (i.e., impacted teeth, infected teeth, hypofunctional teeth, and hyperfunctional teeth).

### 4.1. Impacted Teeth Are Characterized by a Diffuse and Asymmetric Hypercementosis Distribution

The diffuse hypercementosis covering almost the entire root of these teeth (apposition from the apex to the cervical third) could be a marker of impaction. This type of hypercementosis, scored 3.3.m (type 3: diffuse and local type; stage 3: covering all of the three root thirds; and m: moderate form) and extending until the cemento-enamel junction, was not found in other teeth of the sample. Cementum apposition on impacted teeth has been described in the literature, the suggested cause being age [55,76]. In the present study, the average maximum cementum thickness of this impacted group is the lowest compared to the other groups (Table 2). However, the 3D thickness maps show preferential cementum apposition (especially on the distal side) as singular nodule or node shapes (Figure 8). In confocal microscopy, the surfaces showed a smooth aspect with a significant vertical elevation (Figure 8). These patterns seem to contradict the idea that an impacted tooth is passive and has a cementum layer that appends evenly and homogeneously throughout life along the length of the root and around the root axis. This profile of hypercementosis highlighted in our study confirms the hypothesis put forward by Massé et al. [29]. The impacted tooth has a normal periodontium; we could hypothesize that the eruption process was blocked, as malposition or an obstacle would prevent the proper eruption of these teeth to occur. We suggest that eruption events apply forces to the periodontal ligament. Compression zones could potentially result from blocked eruption. Azaz et al. [55] suggested that continuous altered eruptive forces may act as a factor stimulating apposition. The nodules and node appositions could be explained by these compression cycles; the apposition would be a compensatory response to these eruption events. Our results would be in agreement with the observations made by Zemsky [77] on histological sections, showing the preferential apposition of cementum on one side of the tooth root, predominantly located in the middle to apical root thirds, with a notable surface irregularity.

Though extremely rare, impacted canines have been observed in fossil individuals. López-Valverde et al. [78] and Dean et al. [79] described an impacted left permanent canine belonging to a Neanderthal from northern Spain (mandible SDR 7-8, Cueva de El Sidrón-Asturias, Spain, 90–40 ka). Bailey and Hublin [40] described an isolated left mandibular canine (canine Xb Z8, Grotte du Renne at Arcy-Sur-Cure, France) showing no trace of occlusal or proximal wear and suggested that it was an inclusion. This tooth shows a hypercementosis similar to the impacted canines in our sample (see Figure 4.4 in [40]). The hypercementosis is diffuse, covering almost the entire root, and cementum seems to be thicker on the distal side. This thickening is at the junction between the apical and middle root thirds. The presence of an impacted mandibular canine has also been described on the Le Moustier 1 Neanderthal adolescent [80]. It would be very interesting to look at micro-CT data of these teeth to further investigate their root morphology and to characterize their cementum distribution.

### 4.2. Infected Teeth Have an Extensive and Non-Preferential Hypercementosis Distribution

The second group identified concerned teeth that had lost their crowns as a result of a carious process and/or heavy wear. The maximum thickness of cementum shows the highest average in the sample. In response to an infectious process, cementum seems to deposit more extensively and would surround the root uniformly.

According to Beauval et al. [45], the overall frequency of carious lesions in the Sains-en-Gohelle series is 74.8 % for adults. These values are particularly high and exceed those found in other European medieval series (e.g., [81]). Expectedly, this high prevalence in carious lesions is to be correlated with a diet rich in cereals and carbohydrates [82].

Carious lesions form following a progressive process and are induced by the consumption of sugar-rich diet and poor oral hygiene. Early caries are limited to the enamel and may not cause any symptomatic manifestation. However, when the process becomes more extensive, the dental pulp is no longer protected from its external (oral) environment, leading to inflammation (e.g., [83]).

We propose that an initial compensatory response consists of forming hypercementosis. If the inflammation is left untreated, tooth decay continues its progression, and bacteria pass through the dentine to reach and contaminate the pulp, resulting in an infection. An abscess may form around the root, thus destroying the infected bone and even creating fenestration of the dental alveolus. A second episode of hypercementosis formation may occur in response to this infection. This hypothetical multiple apposition would explain the high cementum thickness in this group and could also explain the mixed patterns found in our sample where apposition is moderate and homogeneous on the root (e.g., Sp876_45 and Sp914_35) in response to inflammation due to early caries.

Caries are a marker of oral health but also provide information on the diet of past populations [84]. Carious lesions are more frequently described at the beginning of the Holocene, some 11,000 years ago, with the Neolithic revolution (e.g., [85]). Yet, among the Middle Paleolithic hominids, dental caries remain rare [36,80]. In fact, the Neolithic is marked by the increased use of animal husbandry and agriculture. This revolution saw a rapid and unprecedented increase in the quantity of carbohydrates in the diet, provided by cereals (e.g., [84,85]). However, some cases have caught our attention, suggesting that caries may be older than previously thought. Lacy et al. [86] described a case of hypercementosis on a mandibular molar dated to the Late Pleistocene (China—ca. 100 ka B.P.). The presence of hypercementosis was attributed to a major carious lesion resulting in pulp exposure. Similarly to Sp666_33 in our sample, the cementum seemed to be preferentially deposited on the same side as the caries (Supplementary Information S1, pp. 82–84). Tillier et al. [37] described a Neanderthal maxillary incisor exhibiting extensive decay (KMH27—ULI2, Kebara Cave, Israel, 60 ka B.P.; [87]), where an apposition of cementum was observed in the cervical half of the root. Micro-CT data would enable the assessment of whether the distribution and thickness of cementum in these teeth would match the same pattern as that found in our sample. Beauval et al. [45] also reported that approximately 86 % of adult individuals from Sains-en-Gohelle exhibited dental calculus, indicating poor oral hygiene within this population. However, it should be noted that the potential loss of information and the preservation of such remains make these results tentative in nature. The amount of calculus and its location below the cemento-enamel junction may be indicative of a periodontal disease, which is also known as a factor regulating cementum apposition. It should be noted that the frequency of dental calculus may be high in Paleolithic hunter-gatherer populations. However, in the context of archeological studies, it is important to consider biases and the loss of information that occur over time. These factors can have an impact on the actual frequency of observed dental calculus. The physico-chemical properties of saliva play a major role in calculus formation. If the saliva is not permanently acidic, as in the case of a protein-rich diet (e.g., raw meat and fish), then food deposits can mineralize and turn into calculus [88]. This may explain why calculus is found in such high quantities in these populations (e.g., [68]). Our results showed that in combination with other factors, cementum apposition appeared to be important (e.g., Sp_666_33; Supplementary Information S1 pp. 82–84). We suggest that periodontal disease causes deposition of compensatory cementum, in addition to an already-existing factor (e.g., [42]). This is possibly the case for the “Red Lady” (mandibular teeth of a Magdalenian human skeleton from the site of El Mirón cave, Spain; 18 ka B.P.) described by García-González et al. [39]. Based on micro-CT data, they described an extensive and generalized cementum apposition in this specimen, with cementum thickness ranging from 260 to 1380 μm [39]. More specifically, in the molars and premolars, the deposition affects the entire root height, but in the incisors, it only appears in the apical half of the root both on the mesio-distal and bucco-lingual sides [39]. Those instances of generalized hypercementosis can be found in cases of the Paget’s disease (e.g., [89]). Regarding the “Red Lady”, the authors have nevertheless excluded this possibility after conducting bone analysis [39]. Nevertheless, they did not produce high-resolution hypercementosis profiles of these teeth. Considering the extensive wear present in this specimen, we propose that a combination of these two factors (e.g., periodontal disease and intensive masticatory effort) was at play. We hypothesize that the location of maximum cementum thickness would align with that observed in hyperfunctional teeth.

### 4.3. Hypofunctional Teeth Reveal a Moderate, Non-Preferential and Diffuse Pattern of Hypercementosis

The third group defined concerns hypofunctional teeth either because the degree of wear was minimal or because the antagonist tooth was lost *ante-mortem*. They all showed the same pattern of hypercementosis (i.e., hypercementosis scored at 1.2.m). These teeth had the lowest average maximum cementum thickness of our sample, with non-preferential apposition. In addition, the vertical elevation was always lower than 200 μm.

Two teeth are of particular interest since they seem to correspond to two distinct phenomena: (i) the first concerns mesial drift, i.e., the tendency of the teeth to move towards the anterior portion of the dental arcade with age, due to strong interproximal wear [90]. The origin of the physiological mesial drift in posterior teeth is not fully understood, yet cementum remodeling was suggested to result from these tooth movements (e.g., [91,92]). Specimen Sp20_35 (Figure 10) has a similar cementum thickness on its mesial and distal sides. However, it has a slightly different mesial surface topography, with small irregular lacunae in the cementum on the mesial side and more regular lacunae of larger diameter on the distal side. We hypothesize that this tooth was undergoing a mesial drift, with remodeling of its cementum with lacunae of resorption (mesial side) and fibrous insertions (i.e., Sharpey’s fibers) in tension (distal side). The hypothesis of a fibrous tension creating regular lacunae on the surface of the cementum also applies to Sp173_45, and corresponds to the second phenomenon: super eruption (ii). Nasmyth [56] was one of the first to correlate the fact that teeth super-erupted as soon as they lost their antagonists, and that this phenomenon could lead to excessive cementum production. We propose that by super-erupting the tooth initiates a tension of the Sharpey’s fibers (i.e., fibers of the periodontal ligament holding the tooth root in its bone socket) and causes a compensatory cementum apposition to the space left empty.

### 4.4. Hyperfunctional Teeth Show a Preferential Hypercementosis with an Apical Localization

The last group comprised teeth described as hyperfunctional because they show either significant or atypical wear. The degree and/or orientation of wear would be correlated with cementum apposition.

Dental wear affects 91.4 % of the adults in the Sains-en-Gohelle series with conditions that are not a priori purely food-related. Differential wear between the maxillary and mandibular teeth led Beauval et al. [45] to consider para-masticatory activities.

Many authors have already suggested that intensive and repetitive masticatory efforts in past populations would lead to hypercementosis (e.g., [68,69,93,94]). Our micro-CT data showed that cementum apposition was preferential, with an apico-palatal location for maxillary incisors. These results are in line with those described by Le Cabec et al. [30] on a large series of Neanderthal anterior teeth. These authors correlated this cementum apposition with the teeth-as-tools hypothesis. Other para-masticatory (e.g., “right-handed stuff-and-cut actions” in D2735 and D211, Dmanisi hominins, Georgia, ~1.77 Ma B.P.; [73]) or non-masticatory (e.g., “tooth pick groove” on the M1 and M2 of Aubésier 10 dated to 300–100 Ka B.P.; [95]) activities in past populations have been described.

Hypercementosis apposition seems to correlate with wear, both in degree and orientation. For a few specimens, we could make assumptions regarding the direction of the slope of the wear facets, and the point where occlusal pressure was applied. For instance, for Sp39_35 (see in Supplementary Information S1, pp. 40–42), the wear extends from mesial to distal, and from the cuspal aspect of the mesio-occlusal side to the cervical aspect of the disto-occlusal side of the crown. The associated preferential cementum apposition was observed on the mesial and apical sides of the root in this tooth. The preferential cementum apposition appeared to be located on the side opposite to the point of application of occlusal stress. The repeated movement on the occlusal surface would cause a lever arm, whose center would be located between the cervical and middle root thirds ([96,97]). This lever arm would cause the root apex to have a compression zone (i.e., the apex would be pushed against its periodontium) and a tension zone (i.e., the apex would pull on its periodontal attachment) (see Figure 5 in [29]). Our results support the hypothesis of Le Cabec et al. [30], who proposed that cementum apposition would be more abundant in compression areas. Moreover, the surface topography of the teeth presently studied showed rough, ridge-shaped profiles (e.g., Sp1135_11, Supplementary Information S1, pp. 75–78), preferentially found on the buccal aspect and suggesting areas of fibrous tension (i.e., Sharpey’s fibers).

### 4.5. Which Diagnostic Criteria for Hypercementosis in the Sains-en-Gohelle Sample?

Results from the statistical analyses highlight several criteria that accurately classify hypercementotic teeth according to a given etiology (see Section 3.6, and Supplementary Information S2). The decision tree (Figure 15) enabled to visualize this dichotomic classification with key criteria distinguishing groups that are almost consistently pure (six out of seven) based on these patterns of cementum apposition.

The quantitative analysis of the microtomographic data, corroborated by statistical analyses, revealed a clear distinction between two types of groups. On the one hand, hyperfunctional and impacted teeth are characterized by a preferential cementum apposition, suggesting a response to specific mechanical actions (e.g., hypotheses of para-masticatory activities for hyperfunctional teeth and eruption events for impacted teeth). On the other hand, infected and hypofunctional teeth exhibit a more homogeneous distribution of cementum around the root. As expected, some teeth from the mixed condition fell in various places on the tree (Figure 15), this is easily explained by the nature of this group, encompassing teeth that have changed their condition over time, and thus accumulated characteristics of cementum apposition that match with several of the pure and well-defined groups. This is the case of a mixed-condition tooth, Sp735_45, being classified as hyperfunctional. Similarly, in the FAMD analysis (Figure 14), the frequent overlap of the mixed condition teeth may be explained by multiple etiological episodes undergone by the same tooth over time, thus accumulating features from the other groups.

Importantly, this statistical approach allowed the identification of distinctive and dichotomic criteria for each etiological group. The summary of the most significant of them is listed in Table 3. This systematic and integrated approach represents a significant advancement in our understanding of the responses of cementum to various etiological factors. However, it is important to keep in mind that these criteria and classifications are based on a sample coming from a single population from France, and dated from the Middle Ages to the early modern period.

### 4.6. Future Directions of Research

In spite of using a new methodological approach to characterize hypercementosis in different etiological contexts, the current study involves several limitations (e.g., the sample size and the nature of the sample based on a single population). We propose several lines of future research, which could certainly enrich our understanding of the process at play in hypercementotic teeth. First, one should consider examining teeth from extant populations with well-documented ethnological contexts of para-masticatory use of their front teeth, such as Inuit communities from Greenland (e.g., see [30]). It would also be of great interest to investigate the patterns of hypercementosis in hunter-gatherer populations, such as the Hadza from Tanzania, since there are documented differences in diet and the use of teeth as tools between men and women [30]. Another line of research should obviously involve studying thin sections of hypercementotic teeth (for which permission would be granted to perform a histological section) to better understand the biology and the fine microstructure of cementum. Last, an elemental mapping of key elements (e.g., Ca, Sr, Zn; see [98,99,100]) would provide further valuable information to characterize the deposition patterns of hypertrophic cementum, and about physiology and life history.

## 5. Conclusions

Hypercementosis is frequently found in past populations and may be a marker of non-dietary activities and oral health. The manifestations of hypercementosis are poorly defined in the literature, and overall, not yet well understood. Our findings indicate that factors others than those commonly invoked in the literature, such as age or occlusal stress, may contribute to this excessive growth of cementum. Hypercementosis can be related to impaction, peri-apical infection (i.e., dental abscess) following extensive caries or excessive wear leading to pulpal exposure and infectious contamination. It can also be caused by physiological phenomena such as continuous eruption or mesial drift, which will compensate for the loss of an antagonist tooth or for occlusal wear. We have shown that all these different etiologies may be characterized by specific cementum apposition profile. Our findings would enable us to reconstruct the life history of a past individual and provide insights into their para- or non-masticatory behavior through the study of hypercementosis. Due to this carious lesion, it is likely that the Neanderthal incisor described by Tillier et al. [37] has a completely different pattern from that found in the Neanderthal sample studied by Le Cabec et al. [30]. This marker of activity and health of the individual would give information even in the case of isolated teeth, frequent in the fossil record.

We also demonstrated that different local factors may be responsible for the formation of hypercementosis and could occur in succession on the same tooth (“mixed condition” group). They can induce different forms of cementum apposition and the most recent apposition can, in some cases, obliterate the previous. To some extent, our results could allow one, even on isolated teeth, to determine, at least partially, the oral condition or the masticatory activity of its owner.

Our work has shed light on the various manifestations of hypercementosis and their pattern of apposition, but it has also led to new research questions. The present sample should be extended to a larger sample, involving contemporary populations whose para-masticatory activities have been documented (e.g., Inuit population of Greenland; [9]) in order to validate our results. Furthermore, populations coming from different geographical regions would provide insights into population and dietary variations. Neanderthal specimens would considerably enrich this research and would allow not only the exploration of their non- and para- masticatory activities through the analysis of cementum apposition, but also the investigation of their oral condition. This approach would offer novel insights into their exceptional adaptive processes, encompassing both morphological and behavioral adaptations that unfolded over time to address shifting environments and diverse challenges. The investigation of dental cementum and its nuanced characteristics holds the potential to unveil how Neanderthals evolved to confront specific environmental conditions, dietary resources, and lifestyles, thereby providing a distinct perspective on the evolutionary history of this human lineage.

## Figures and Tables

**Table 1:** Study sample of hypercementotic teeth from the French archeological site of Sains-en-Gohelle (7th to 17th centuries A.D.). Individuals were sexed according to the associated cranial and postcranial remains [45]. Tooth labeling is recalled following the convention of FDI and BA (See Figure 1).

Individual	Sex	Age (yrs)	Tooth Identification	
			FDI	BA
Sp17	F	20–39	15	URP4
Sp20	IND	>20	35	LLP4
Sp39	F	20–49	35	LLP4
Sp114	F	20–60	14	URP3
Sp173	M	>60	45	LRP4
Sp199	M	>30	11	URI1
			12	URI2
Sp335	M	>18	13	URC
			23	ULC
Sp479	M	20–60	11	URI1
Sp583	F	>60	45	LRP4
Sp591	M	20–39	12	URI2
Sp666	M	>30	33	LLC
Sp709	I	>20	31	LLI1
Sp735	M	>20	45	LRP4
Sp755	F	20–39	13	URC
			23	ULC
Sp810	F	>20	13	URC
			23	ULC
Sp876	M	>30	45	LRP4
			35	LLP4
			44	LRP3
Sp884	F	>30	34	LLP3
			35	LLP4
Sp914	M	20–39	33	LLC
			34	LLP3
			35	LLP4
			43	LRC
Sp1010	M	>30	44	LRP3
Sp1135	M	>30	11	URI1
Sp1172	F	>30	15	URP4
			24	ULP3
Sp1230	M	>20	34	LLP3
			44	LRP4
Sp1300	MD	MD	14	URP3

Abbreviations stand as follows: Sp = burial (“sépulture” in French); F = female; M = male; I = indeterminate; MD = missing data (e.g., no skeletal parts preserved besides the teeth); yrs = years; FDI = Fédération Dentaire Internationale (World Dental Federation); BA = Biological Anthropology. Concerning the BA tooth identification, the code comprises three letters in upper case (since all teeth are permanent) and a numeral: for the first letter, ‘U’ stands for ‘upper’ (i.e., maxillary), while ‘L’ stands for ‘Lower’ (i.e., mandibular); for the second letter, ‘L’ stands for ‘left’, and ‘R’ for ‘right’; the third indicates the tooth type (‘I’ for incisor, ‘C’ for canine’, ‘P’ for premolar, and ‘M’ for molar), and the number indicates the tooth locus (‘I1’ is a central incisor while ‘P4’ is a second premolar).

**Table 2:** Detailed results of the hypercementosis characterization for each specimen investigated, with scoring of features from visual examination using macrophotographs, and inner dental tissues characterization using micro-CT scans.

		Specimens					Visual Examination									Microtomography—3D Thickness Maps						Confocal Microscopy—3D Topography Maps							
			Tooth	Occlusal Wear			CAR Si/Sta	Pulp EXP	IMP	HC	Bone Context					MAX THI (µm)	MAX LOC	MIN LOC	PREF LOC	Note		MAX LOC	MAX VE (µm)	ST	Note	OF	MAX VE (µm)	ST	Note
				DEG	DIR	FOR					FEN	CAL	NT	ANT															
Group 1: Impacted teeth		Sp755_13	URL	1	1	1	-	0	1	3.3.m	0	0	-	-		1000	2d	<	Yes	Nds (d)		2d	690	+S2	Nds	2m	100	-R2	
		Sp755_23	ULC	1	1	1	-	0	1	3.3.m	0	0	-	-		1070	2d	<	Yes	NOD (d)		2d	450	+S2	IL	2m	90	-R2	IL
																													
Group 2: Infected teeth		Sp114_14	URP3	NA	NA	NA	1/4	M	0	3.4.M	1	0	0	1		1470	m	<	No	OG		m	475	+S2					
		Sp335_23	ULC	NA	NA	NA	1/4	M	0	1.4.M	1	0	2	1		2160	<	>	No	RES (>)		<	250	+R2					
		Sp914_43	LRC	NA	NA	NA	1/4	M	0	1.4.m	0	0	0	NA		1950	d	m	No	-		d	450	+S2					
		Sp1172_15	URP4	NA	NA	NA	1/4	M	0	1.4.M	0	0	1d	1		1380	d	No	No	RES (d)		d	110	-S2	AF	m	220	-S2	AF
																													
Group 3: Hypo functional teeth		Sp17_15	URP4	3	4	2	-	0	0	1.2.m	0	0	1d	1		1640	2m	<	No	FR (2m)		2m	160	-R2	FR				
		Sp20_35	LLP4	2	6	2	-	0	0	1.2.m	0	0	0	1		1310	1m	<	No	-		1m	180	-S2	IL	2d	150	+S2	CL
		Sp173_45	LRP4	3	6	3	-	0	0	1.2.m	0	0	0	2		1470	1>	<	No	SE		1>	170	-S1	CL				
		Sp914_34	LLP3	2	1	1	-	0	0	1.2.m	0	0	0	NA		1410	1d	No	No	-		1d	140	-S2					
		Sp1230_34	LLP3	3	1	1	-	0	0	1.2.m	0	1	0	1		1240	1m	No	No	-		1m	110	-R2					
		Sp1230_44 *	URP3	3	1	1	-	0	0	1.2.M	0	0	0	1		1280	1d	m	No	2 roots									
																													
Group 4: Hyper functional teeth		Sp39_35	LLP4	8	4	2	-	0	0	1.2.m	0	0	2	1		740	1m	No	Yes	-		1m	400	+R2					
		Sp199_11	URI1	6	6	4	-	0	0	1.2.m	0	0	0	1		1380	1>	<	Yes	RES (1<)		1>	170	-R1					
		Sp199_12	URI2	6	4	4	-	0	0	1.3.m	0	0	0	1		900	1>	<	Yes	-		1>	230	+R1	IL	1<	350	+R1	
		Sp335_13	URC	4	2	2	-	0	0	1.2.m	0	0	2	1		1970	1d	<	Yes	FR (1>m)		1d	325	+R1					
		Sp479_11	URI1	7	2	2	-	0	0	3.2.M	0	0	0	1		1480	1>	<	Yes	NOD (m>)		1m>	665	+S2	NOD				
		Sp591_12	URI2	5	4	6	-	0	0	1.1.m	0	0	1d	1		1510	1>	<	Yes	FR (1>)		1d	192	-R2	FR				
		Sp810_13	URC	3	2	2	-	0	0	1.3.m	0	0	0	NA		1030	1>	<	Yes	-		1>	200	+R1					
		Sp810_23	ULC	3	2	2	-	0	0	3.2.m	0	0	0	NA		790	2>	<	Yes	Nds (>)		2>	475	+S2	Nds				
		Sp876_35	LLP4	6	2	6	-	0	0	1.2.m	0	1	2	2		1010	1>	<	Yes	-		1>	250	+R2					
		Sp914_33	LLC	4	4	4	-	0	0	1.2.m	0	0	0	NA		1270	1>	No	Yes	-		1>	95	-R1					
		Sp1010_44 *	LRP3	5	4	6	-	0	0	3.2.M	0	0	1d	NA		2080	1d	m	Yes	2 roots									
		Sp1172_24	ULP3	6	2	4	-	0	0	3.2.M	0	0	1d	1		1200	2d	<	Yes	NOD (d)									
		Sp1135_11	URI1	6	4	5	-	0	0	1.3.m	0	0	0	1		1180	1>d +2m	1<	Yes	RID (<)		1m	300	+R1		2<d	260	+R1	RID
																												
Group 5: Mixed conditions		Sp583_45	LRP4	6	4	2	-	0	0	3.2.M	0	1	1d	2		2770	1	<	Yes	LSP (1)		2>	43	-S2					
		Sp666_33	LLC	7	5	6	2/2	0	0	3.2.M	0	1	1m	NA		2350	1m	<	Yes	NOD, CAR (m)		1m	475	+S2	NOD				
		Sp709_31	LLI1	6	6	4	-	0	0	3.1.m	0	1	0	NA		1330	1<	No	Yes	LPS (<)		<	550	+R1	LSP				
		Sp735_45	LRP4	4	6	3	2/4	C	0	1.2.M	0	0	1d	2		1230	1m	No	Yes	CAR (m)		1m	175	-R1		d	118	-S2	IL
		Sp876_44	LRP3	NA	NA	NA	1/4	M	0	1.4.m	0	1	0	2		1320	1d	No	Yes	RES (m<)		1d	200	+R2					
		Sp876_45	LRP4	4	6	3	2/2	0	0	1.2.M	0	1	0	2		1670	1d	<	No	FR (1>)		2d	200	+R2					
		Sp884_34	LLP3	8	3	6	-	0	0	3.4.M	0	0	1m	1		1450	d	No	No	W		1d	370	+R2					
		Sp884_35	LLP4	8	7	6	-	W	0	3.4.M	0	0	1d	2		1590	d>	<	Yes	OG (>)		>	310	+R2					
		Sp914_35	LLP4	3	3	3	2/3	0	0	1.2.m	0	0	0	NA		1100	1d	No	No	CAR (d)		1d	375	+R2					
		Sp1300_14	URP3	7	4	4	2/2	0	0	1.2.M	0	0	NA	2		1440	1m	>	No	CAR (m)		2m	280	+R1					
																													

DEG: degree; DIR: direction; FOR: form; CAR Si/Sta: carious lesion (Si/Sta classification); Pulp EXP: pulp exposure; IMP: impacted teeth; FEN: bone fenestration; CAL: calculus; NT: tooth loss in neighboring teeth; ANT: antagonist; C: pulp exposure due to carious lesion; W: pulp exposure due to occlusal wear; M: mixed context, pulp exposure due to carious lesion and/or occlusal wear; MAX THI: maximum thickness; MAX LOC: location of maximum cementum thickness (the number corresponds to the location on the root; when the cemento-enamel junction was not visible, the number was omitted); MIN LOC: location of minimum cementum thickness (if no side could be clearly identified, "No" was used); PREF LOC: preferential location; 1: apical root third; 2: middle root third; 3: cervical root third; m: mesial; d: distal; <: buccal; >: lingual; Nds: nodules; NOD: nodes; OG: atypical overgrowth; RID: ridges; LSP: localized spike-like projection; RES: resorption; FR: fracture; SE: super-erupted tooth; MAX VE: maximum vertical elevation; ST: surface texture; OF: other face; IL: irregular lacunae; CL: contained lacunae; AF: accessory foramen; *: teeth excluded from the sample following the inspection of the µCT data, which revealed that these teeth actually had two roots concealed by the overlaying cementum; NA: not available.

**Table 3:** Significant criteria extracted from the statistical results (See Section 3.6) enabling to characterize the five etiological groups.

Group	Decisive Criteria
Impacted teeth	Preferential apposition of cementum No antagonist tooth No wear, no carious lesion, no pulp exposure Hypercementosis of Stage 3 (cementum apposition from apical to cervical root third) Maximum thickness of cementum < 1295 µm
Infected teeth	No preferential apposition of cementum Pulp exposure/carious lesions
Hypofunctional teeth	No preferential cementum apposition Wear degree of 2 or 3 Hypercementosis of Stage 2 (cementum apposition from apical to middle root third) Maximum vertical elevation of cementum < 190 µm
Hyperfunctional teeth	Preferential apposition of cementum Hypercementosis of Stage 1 (cementum apposition at the apical root third) Wear degree ≥ 6 No carious lesion or pulp exposure Maximum thickness of cementum < 1295 µm
Mixed condition	Overlapping characteristics in FDMA, indicating likely multiple etiological episodes on the same tooth 60% with and 40% without preferential apposition of cementum 40% with maximum elevation of cementum ≥ 190 µm if wear degree ≥ 2 and no preferential apposition 60% with preferential apposition of cementum and presence of antagonist teeth Among these 60%: 50% with maximum cementum thickness of ≥1295 µm, and 10% with maximum cementum thickness of <1295 µm, and wear degree ≥ 3

## Data Availability

The data used in this study are contained within the Supplementary Information S1 or referenced within the text. Raw data supporting the reported results are available on Zenodo (https://zenodo.org/records/10357391, accessed on 12 December 2023).

## Supplementary Materials

The following supporting information can be downloaded at: https://www.mdpi.com/article/10.3390/biology13010043/s1, Supplementary Information S1: Photographs of each tooth, microtomography with 3D cementum thickness maps, and confocal microscopy with 3D topography maps are presented for the whole sample. References [50,62,64,65,101,102,103] are cited in the Supplementary Information. Supplementary Information S2: Statistical methods and results for the univariate and multivariate analyses to explore the link between the measured variables and the five proposed etiologies.
