# Supplementary material for: Unraveling the Life History of Past Populations through Hypercementosis: Insights into Cementum Apposition Patterns and Possible Etiologies Using Micro-CT and Confocal Microscopy
Source: Biology (Basel). 2024 Jan 13;13(1):43. doi: 10.3390/biology13010043 (PMC10813066; doi:10.3390/biology13010043)
Supplement: Supplementary file 1 [file biology-13-00043-s001.zip › Supplementary Information S1.pdf]

Sp755_13

Tooth	Occlusal Wear			CAR Si/Sta	Pulp EXP	IMP	Hypercementosis	Bone Context			
	DEG	DIR	FOR					FEN	CAL	NT	ANTA
**URC**	1	1	1	-	0	1	3.3.m	0	0	-	-

URC

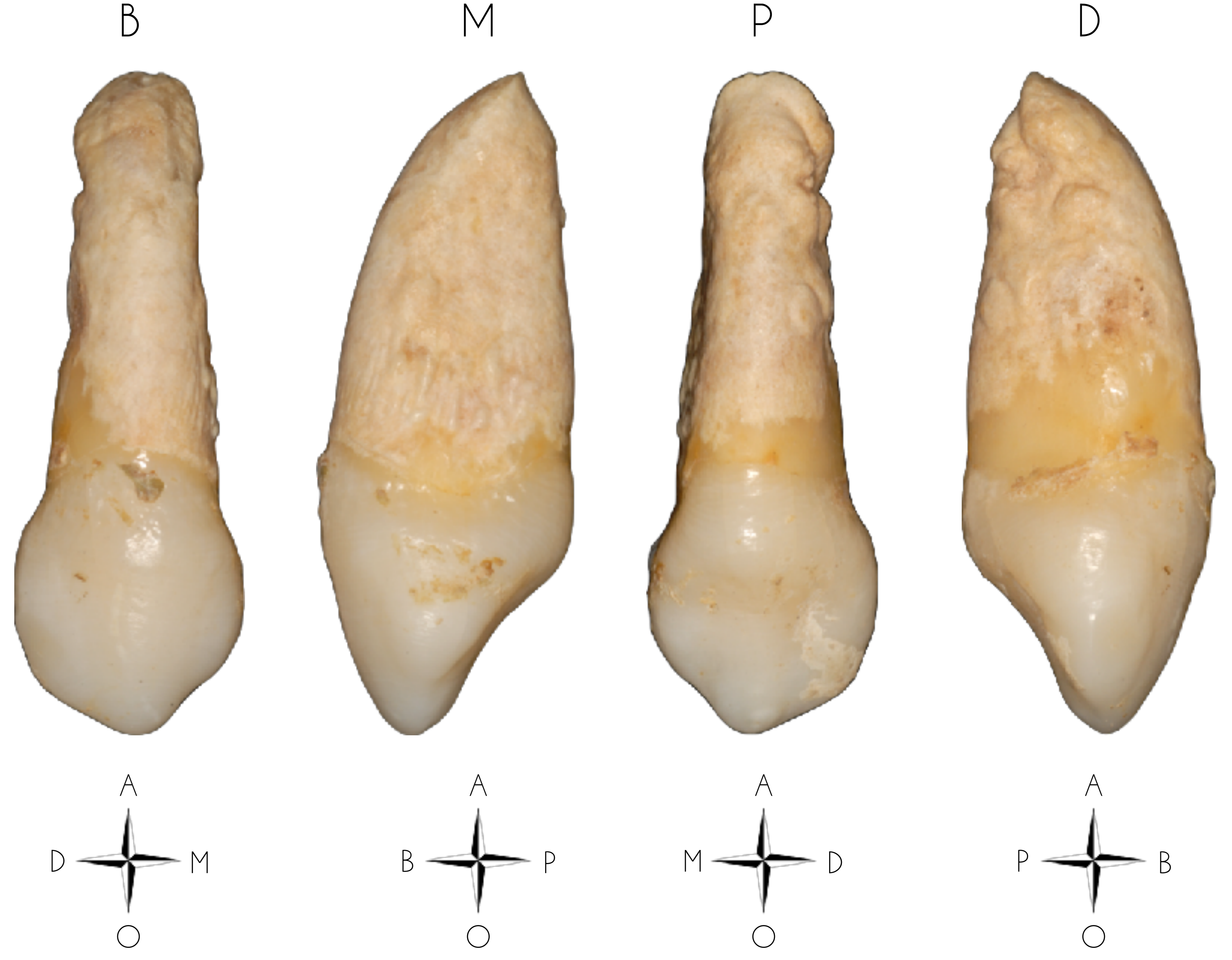

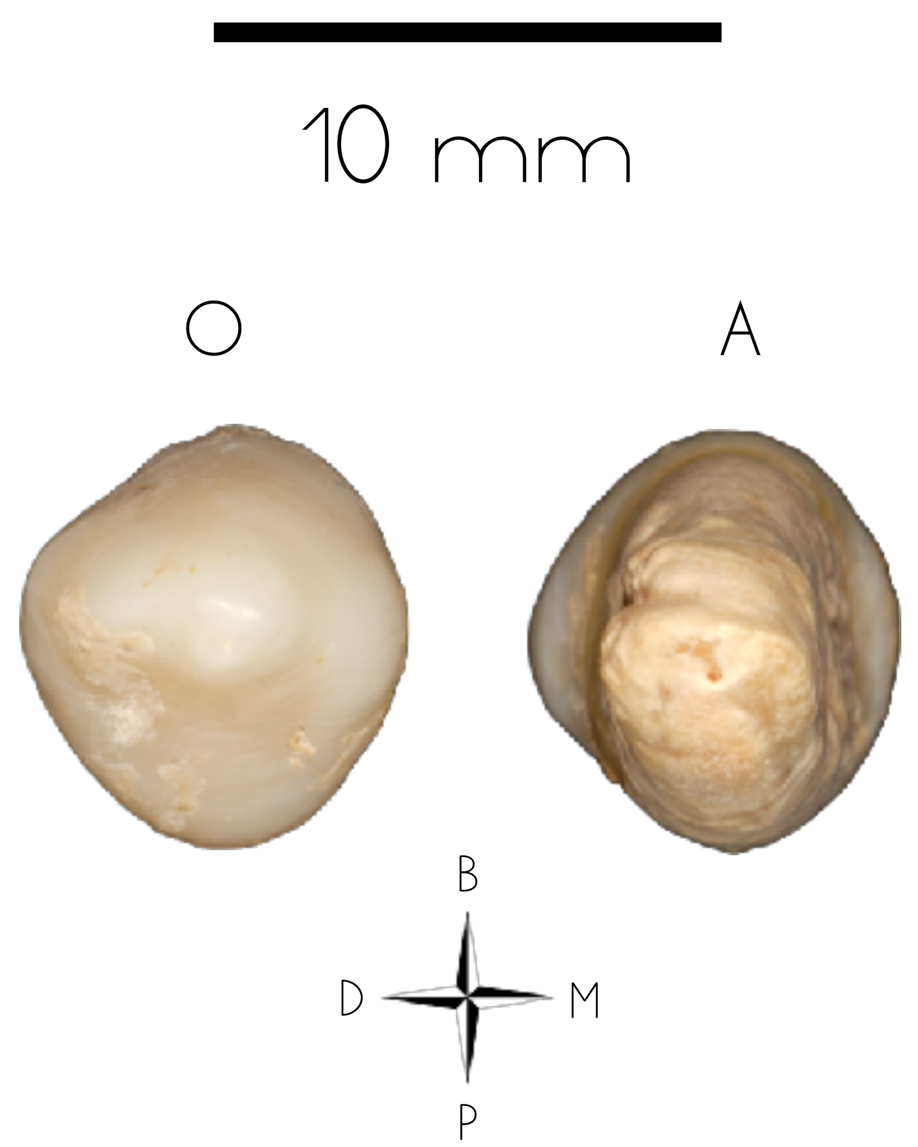

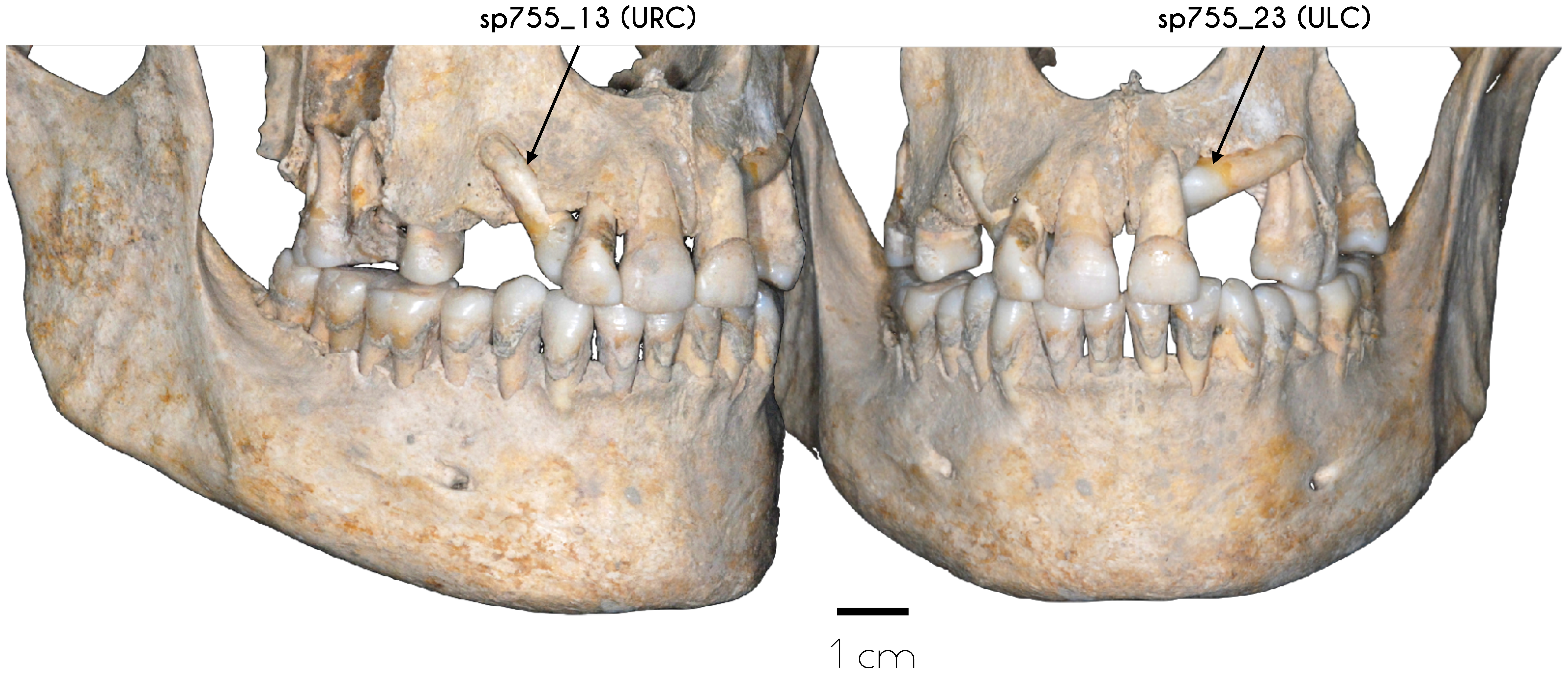

DEG: degree; **DIR:** direction; **FOR:** form - **CAR**: carious lesion - **Pulp EXP:** pulp exposure - **IMP**: impaction - **FEN:** fenestration; **CAL:** calculus; **NT:** tooth loss in neighbouring teeth; **ANTA:** antagonist

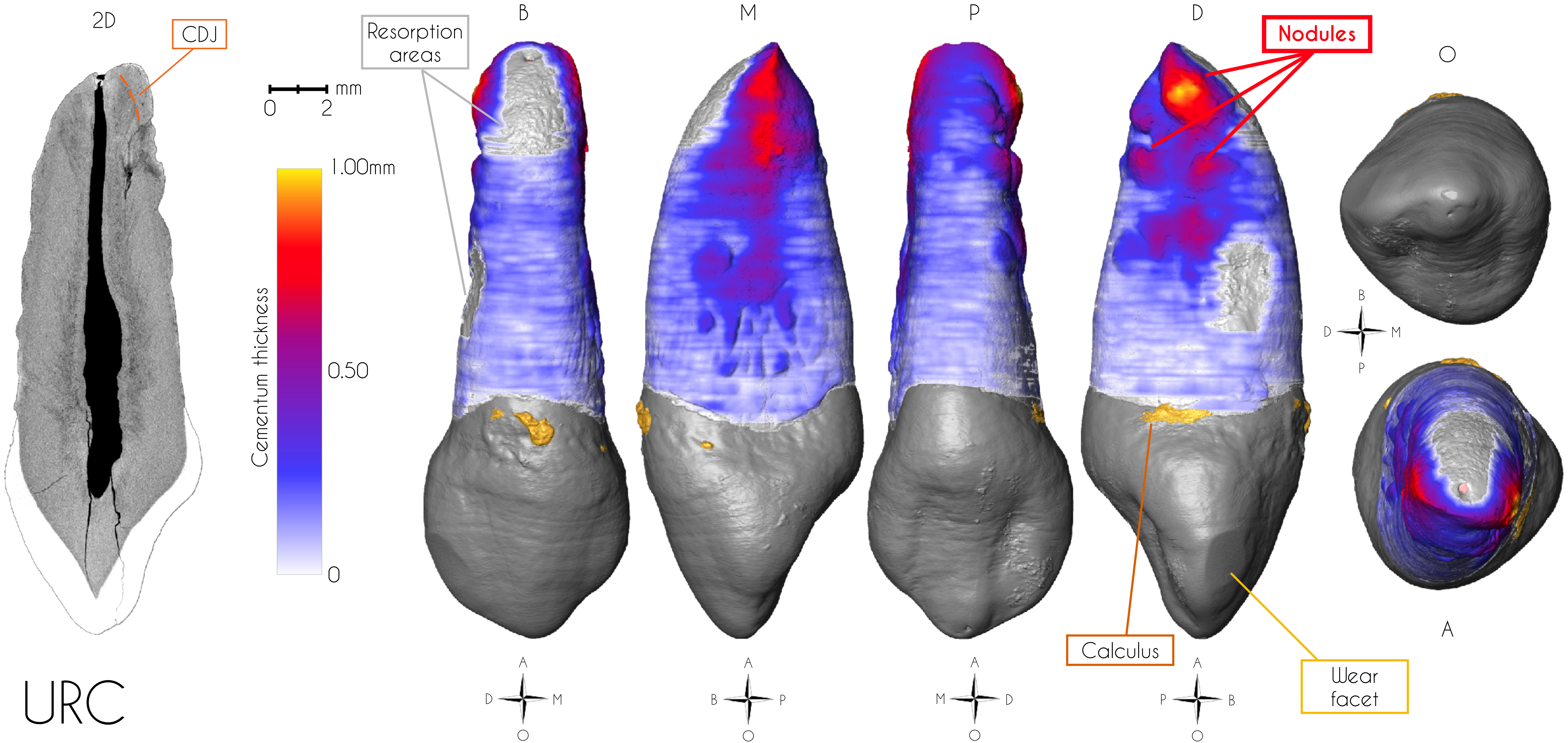

Sp755_13

Microtomography - Thickness 3D maps				
MAX THI (μm)	LOC MAX	LOC MIN	PREF LOC	Note
1000	2d	<	Yes	Nds (d)

MAX THI: maximum thickness of cementum;
LOC MAX: location of maximum thickness;
LOC MIN: location of minimum thickness;
(1: apical root third; 2: middle root third; 3: cervical root third; m: mesial; d: distal; <: buccal; >: lingual)
PREF LOC: preferential location;
Nds: nodules (m: mesial; d: distal; <: buccal; >: lingual)

Impacted

URC

Face	Range	Light	Width	Height	Columns/Rows	Factor	Led Color	Gain	Gamma	Overlapping
D	747	5.24%	0.88mm	6.60mm	1/11	2	Blue	1.5	1.8	10 %

Sp755_13

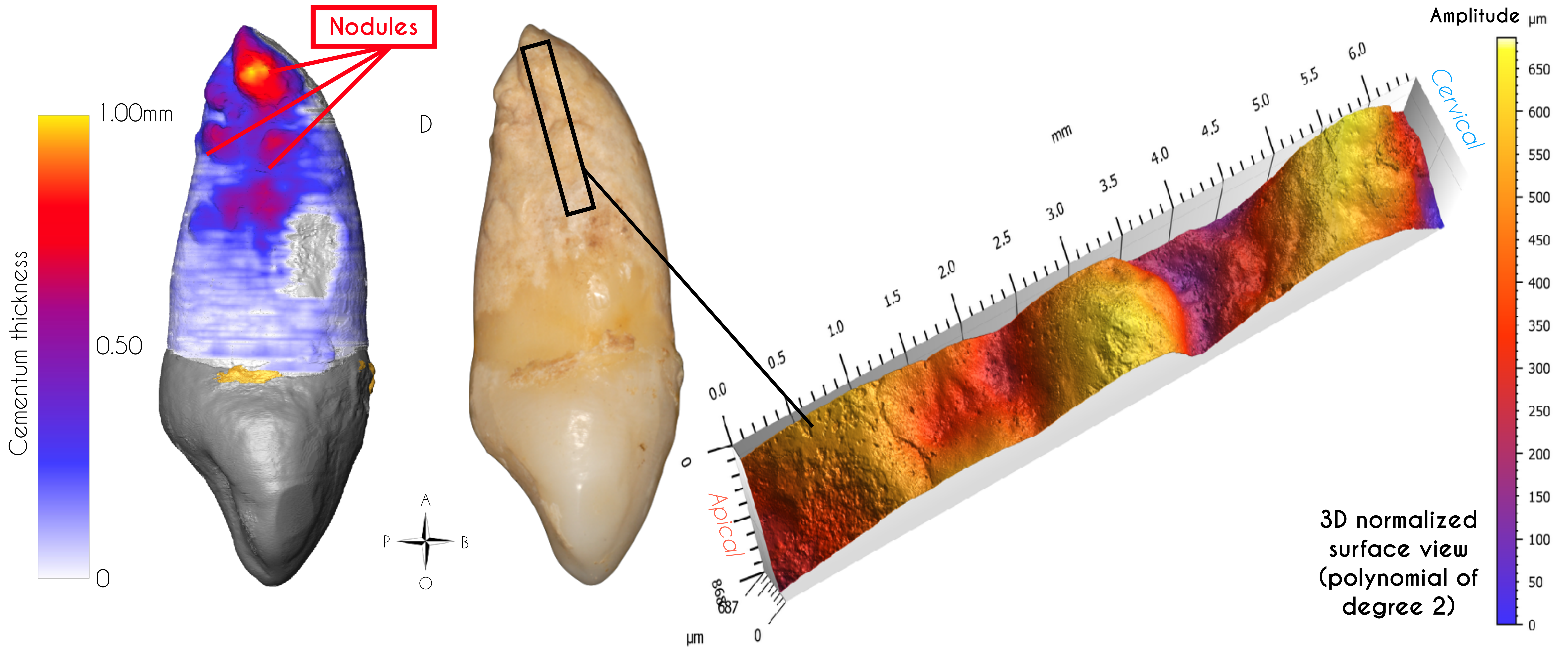

Confocal microscopy - Topography 3D maps			
LOC MAX	MAX VE (µm)	ST	Note
2d	690	+S2	Nds

LOC MAX: location of maximum thickness (1: apical root third; 2: middle root third; 3: cervical root third; m: mesial; d: distal; <: buccal; >: lingual);
MAX VE: maximum vertical elevation;
ST: surface texture; : scored (**+**) if it is high, (**-**) if it is low; **S:** smooth or **R:** rough; the frequency of occurrence of the elevations scored (**1**) if the frequency is high, (**2**) if the frequency is low.
Nds: Nodules

Impacted

URC

Face	Range	Light	Width	Height	Columns/Rows	Factor	Led Color	Gain	Gamma	Overlapping
M	1029	5.00%	0.88mm	3.04mm	1/5	2	Blue	1.5	1.8	10 %

Sp755_13

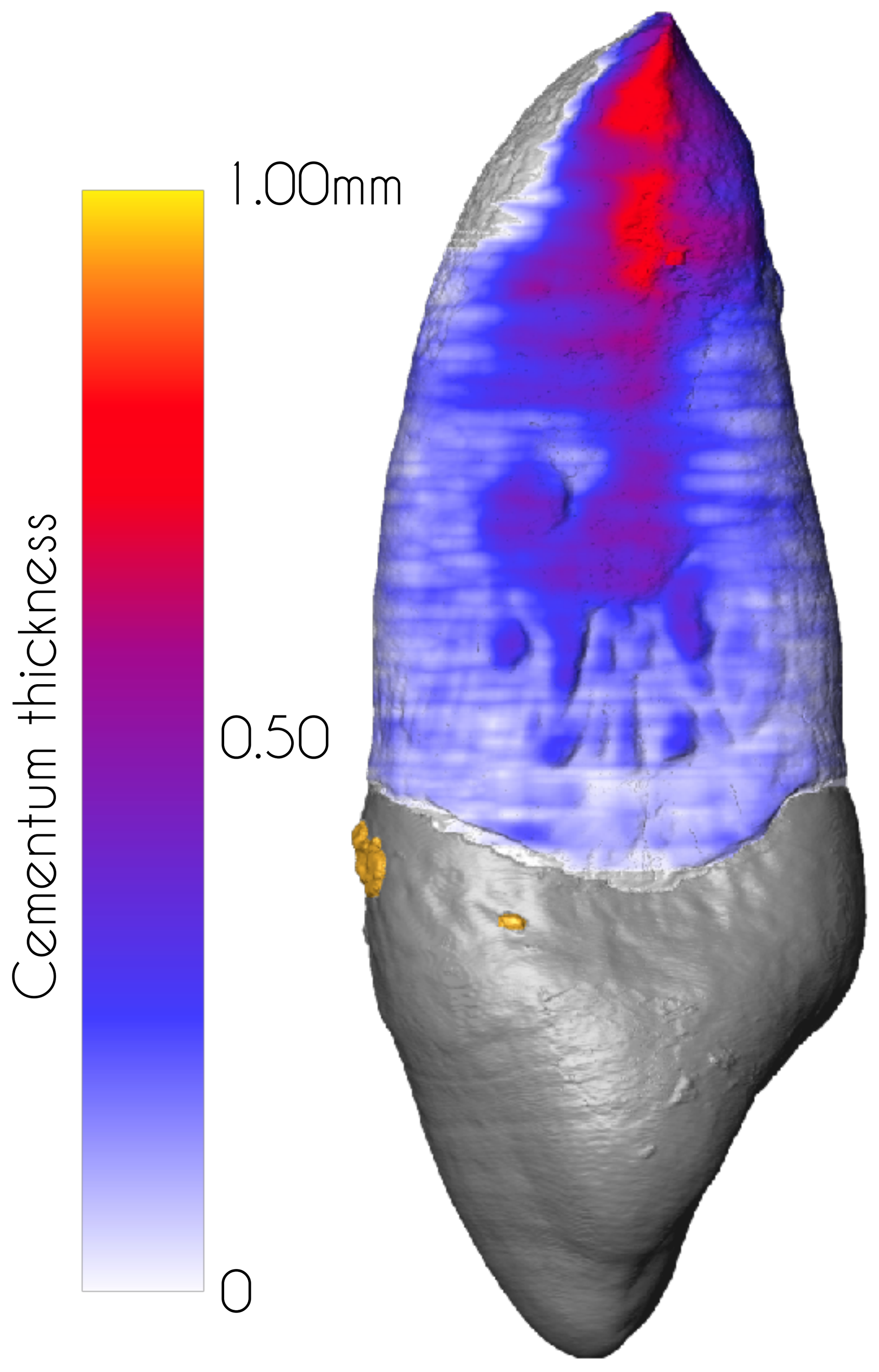

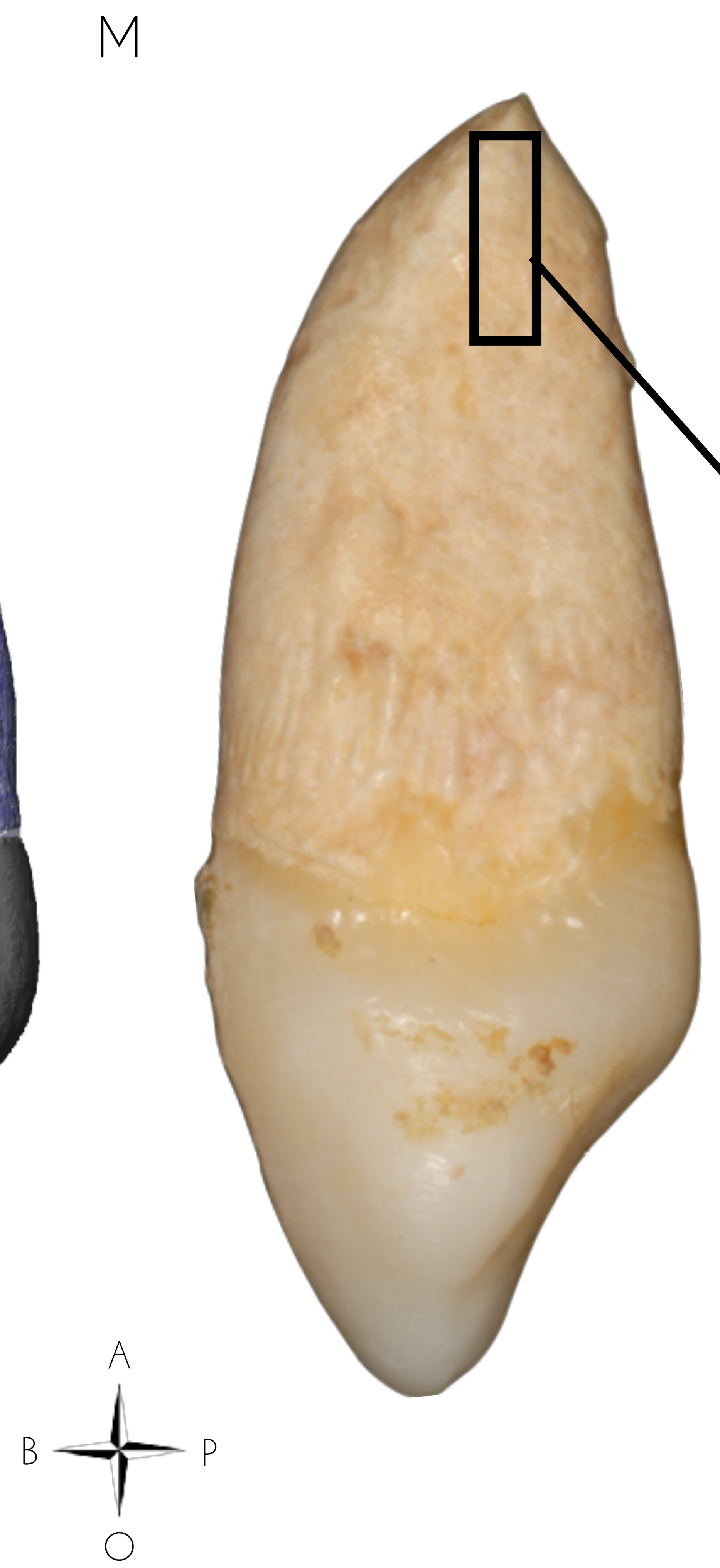

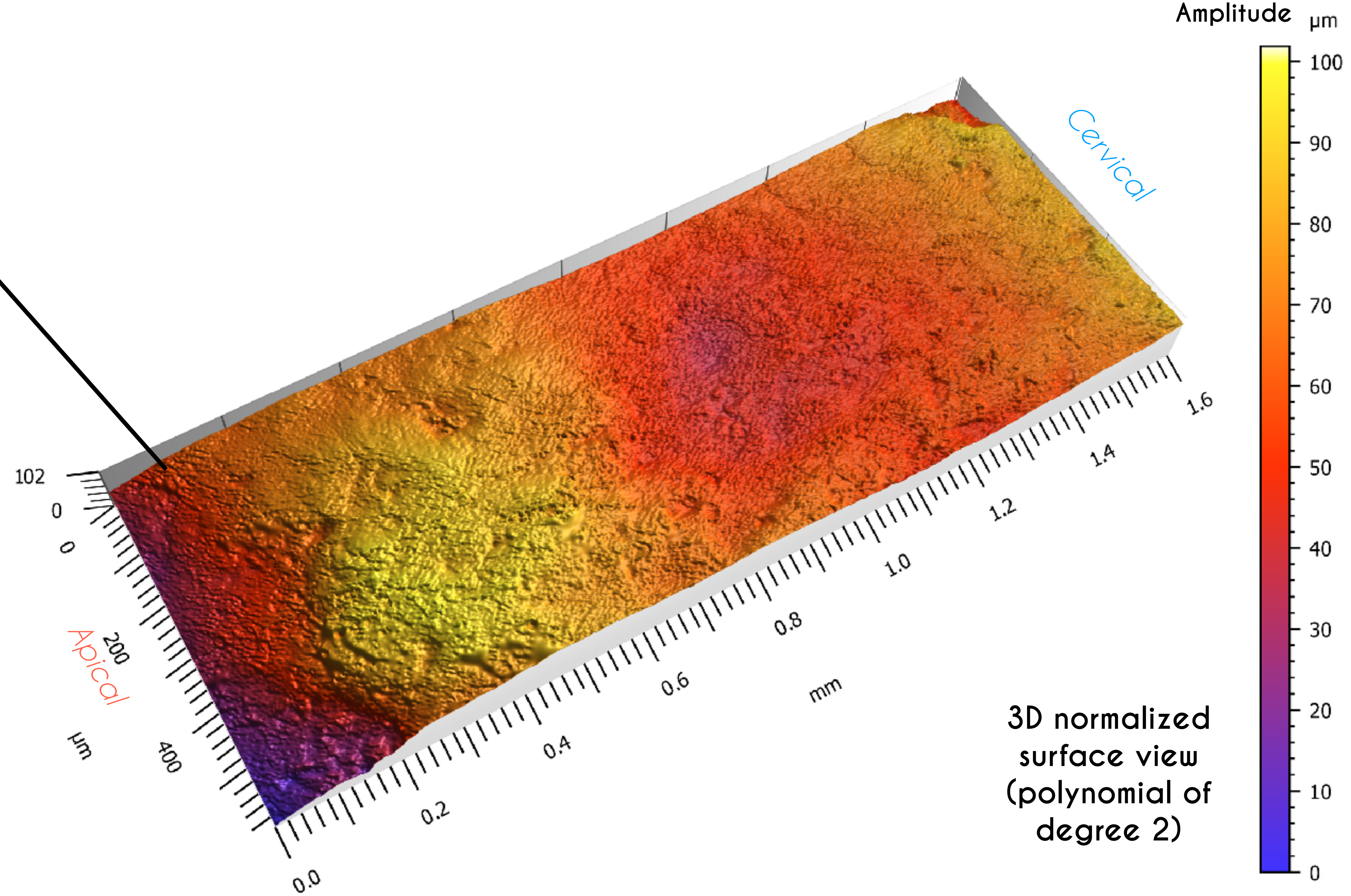

3D normalized surface view (polynomial of degree 2)

Confocal microscopy - Topography 3D maps			
OF	MAX VE (µm)	ST	Note
2m	100	-R2	-

OF: other face (1: apical root third; 2: middle root third; 3: cervical root third; m: mesial; d: distal; <: buccal; >: lingual);
MAX VE: maximum vertical elevation;
ST: surface texture; : scored (**+**) if it is high, (**-**) if it is low; **S:** smooth or **R:** rough; the frequency of occurrence of the elevations scored (**1**) if the frequency is high, (**2**) if the frequency is low.

Impacted

Sp755_23

Tooth	Occlusal Wear			CAR Si/Sta	Pulp EXP	IMP	Hypercementosis	Bone Context			
	DEG	DIR	FOR					FEN	CAL	NT	ANTA
**ULC**	1	1	1	-	0	1	3.3.m	0	0	-	-

ULC

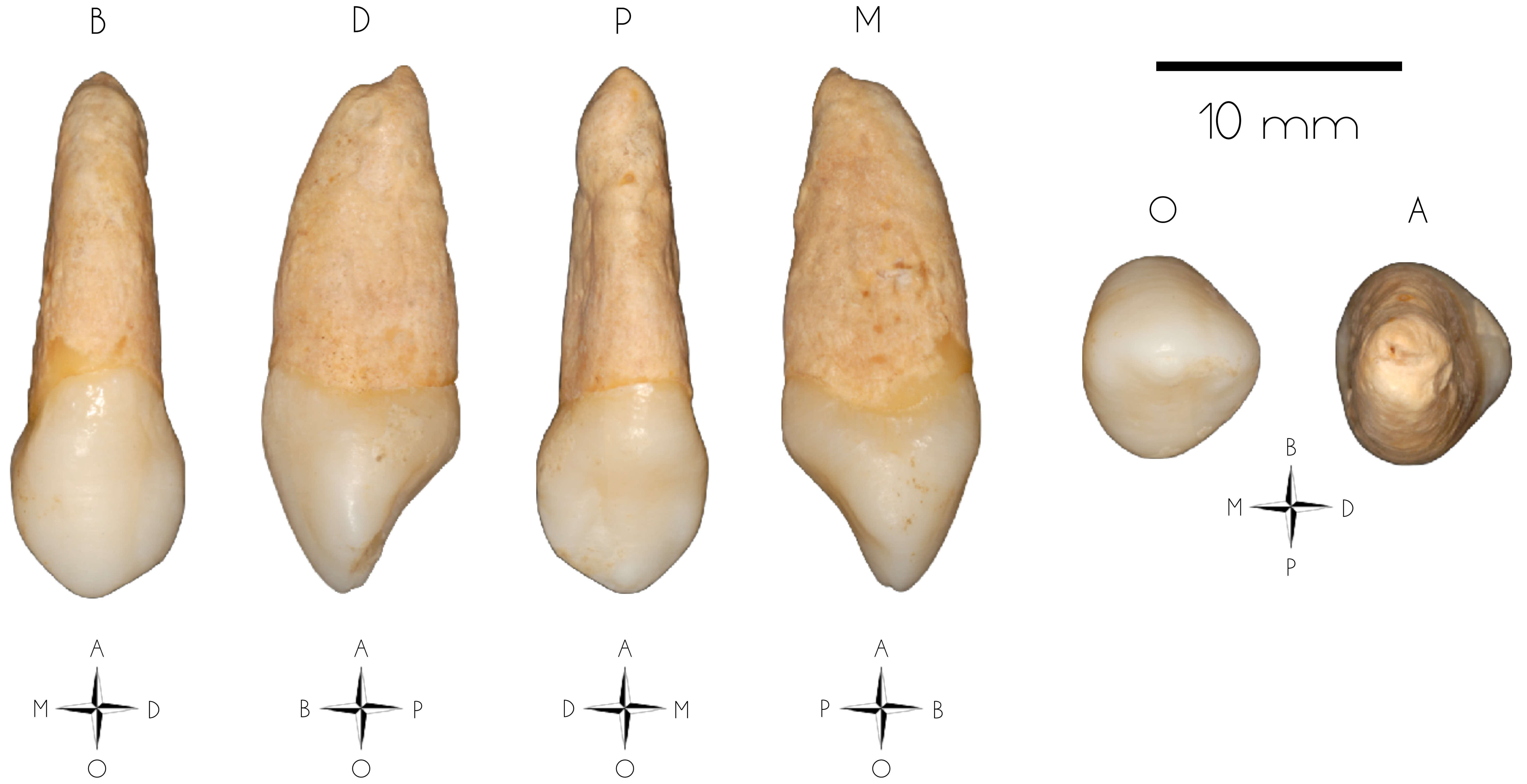

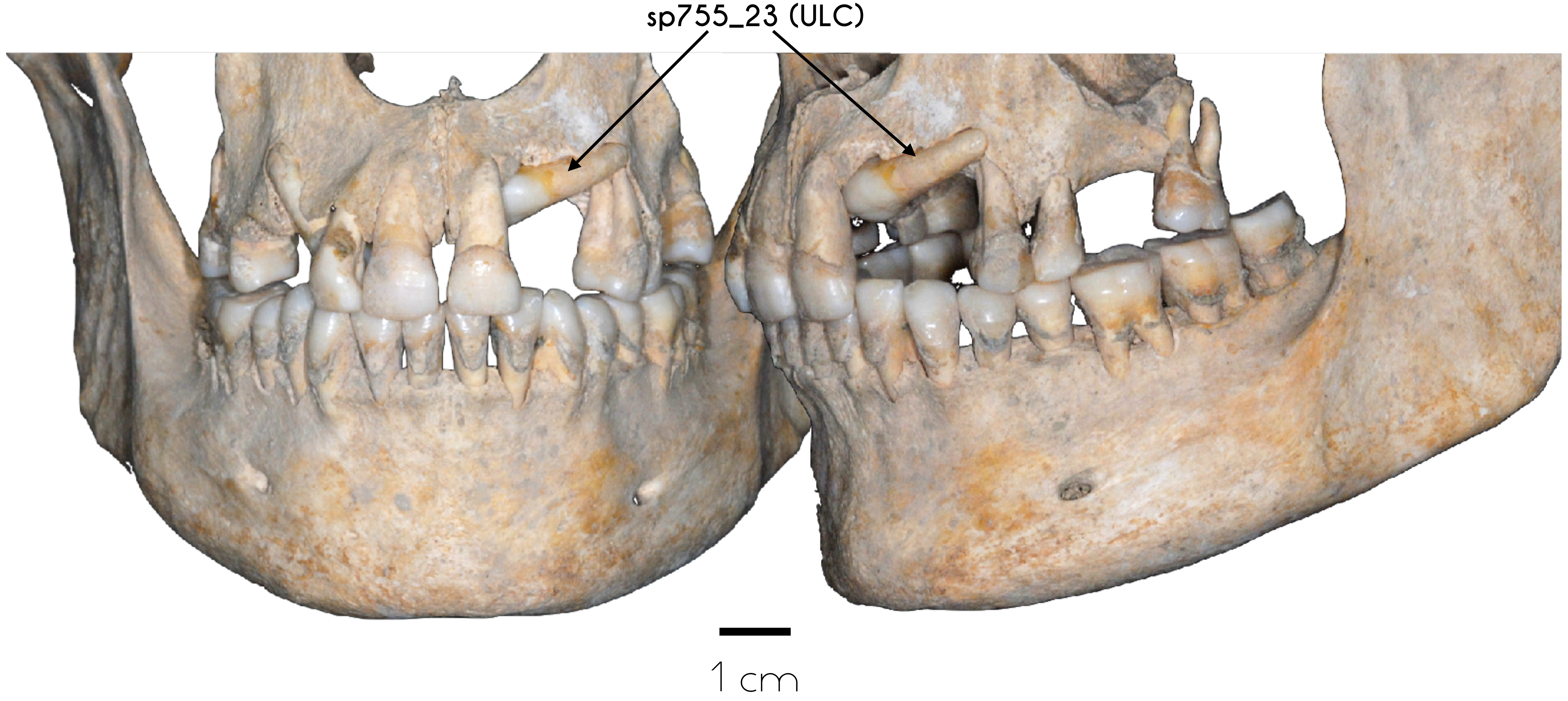

DEG: degree; **DIR:** direction; **FOR:** form - **CAR**: carious lesion - **Pulp EXP:** pulp exposure - **IMP**: impaction - **FEN:** fenestration; **CAL:** calculus; **NT:** tooth loss in neighbouring teeth; **ANTA:** antagonist

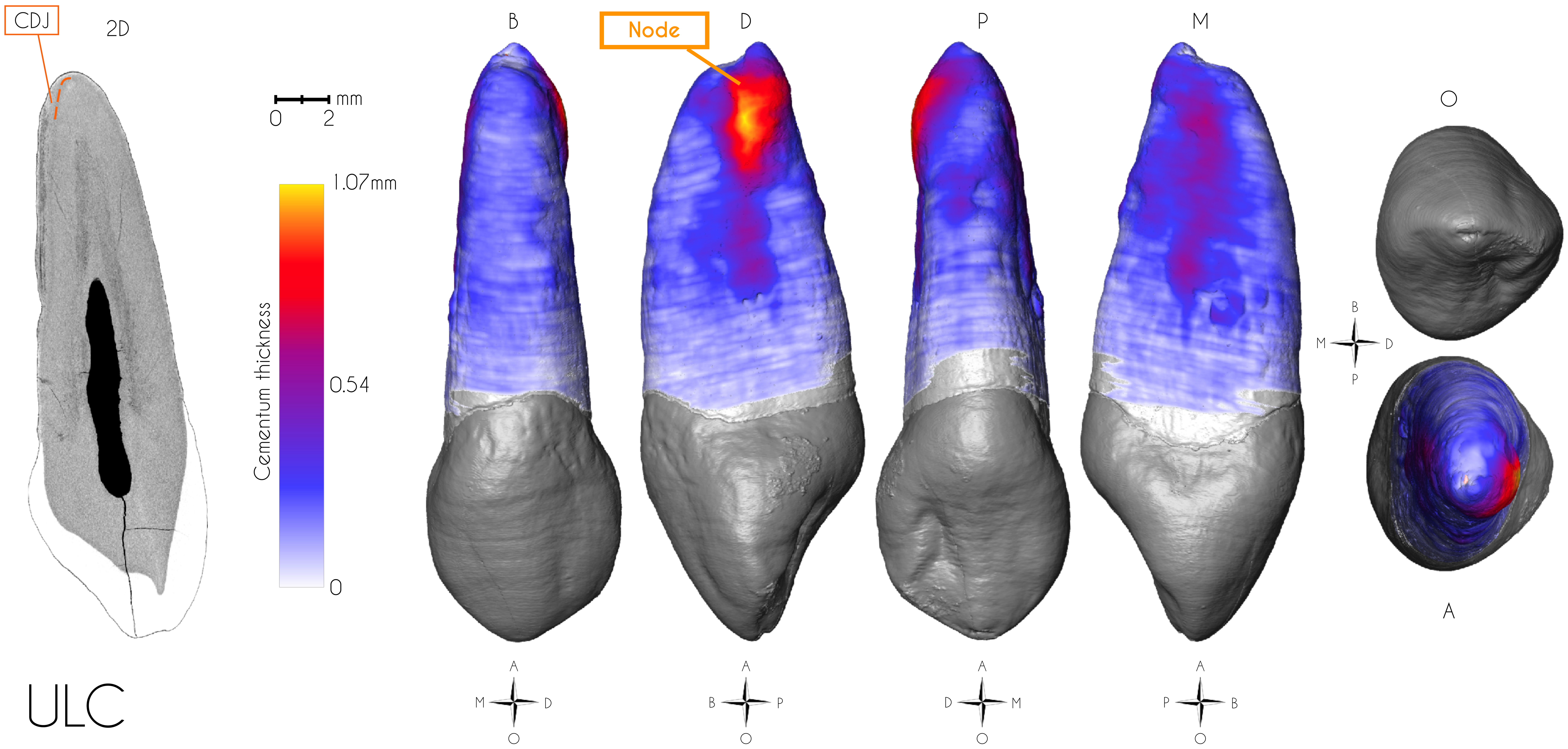

Sp755_23

Microtomography - Thickness 3D maps				
MAX THI (µm)	LOC MAX	LOC MIN	PREF LOC	Note
1070	2d	<	Yes	NOD (d)

MAX THI: maximum thickness of cementum;
LOC MAX: location of maximum thickness;
LOC MIN: location of minimum thickness;
(1: apical root third; 2: middle root third; 3: cervical root third; m: mesial; d: distal; <: buccal; >: lingual)
PREF LOC: preferential location;
NOD: node (m: mesial; d: distal; <: buccal; >: lingual)

Impacted

ULC

Face	Range	Light	Width	Height	Columns/Rows	Factor	Led Color	Gain	Gamma	Overlapping
D	1014	4.97%	1.67mm	4.82mm	2/8	2	Blue	1.5	1.8	10 %

Sp755_23

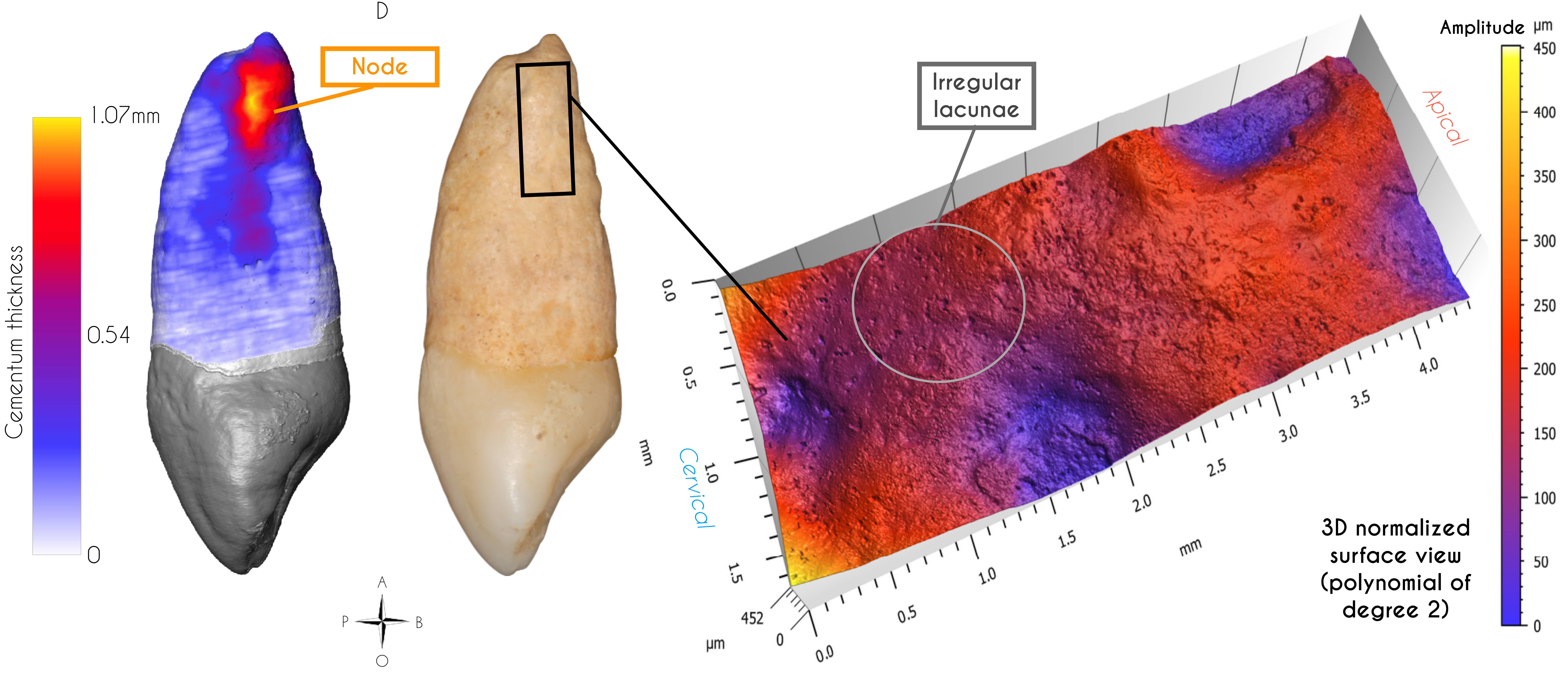

Confocal microscopy - Topography 3D maps			
LOC MAX	MAX VE (µm)	ST	Note
2d	450	+S2	IL

LOC MAX: location of maximum thickness (1: apical root third; 2: middle root third; 3: cervical root third; m: mesial; d: distal; <: buccal; >: lingual);
MAX VE: maximum vertical elevation;
ST: surface texture; : scored **(+)** if it is high, **(-)** if it is low; **S:** smooth or **R:** rough; the frequency of occurrence of the elevations scored **(1)** if the frequency is high, **(2)** if the frequency is low.
IL: irregular lacunae

Impacted

ULC

Face	Range	Light	Width	Height	Columns/Rows	Factor	Led Color	Gain	Gamma	Overlapping
M	751	5.50%	0.88mm	4.23mm	1/7	2	Blue	1.47	1.61	10 %

Sp755_23

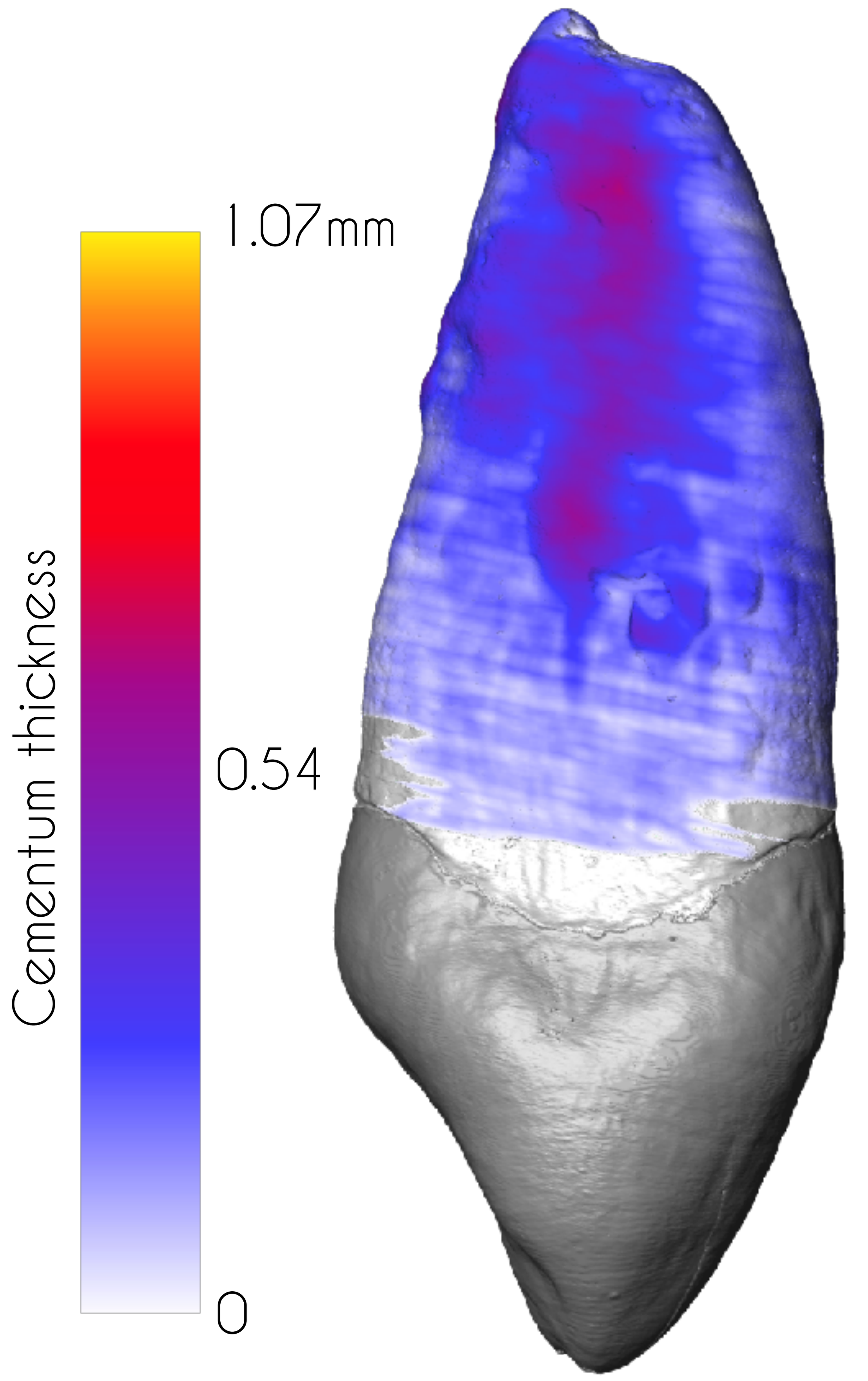

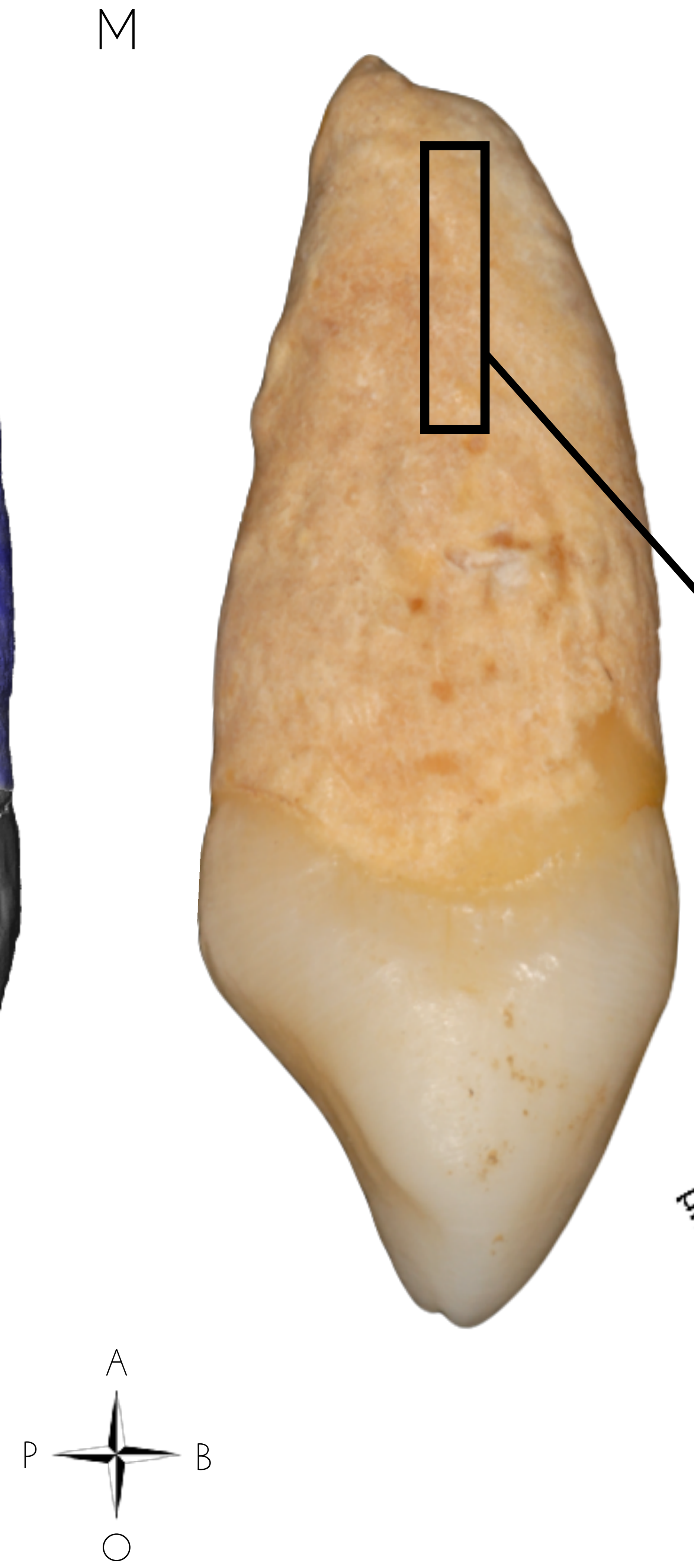

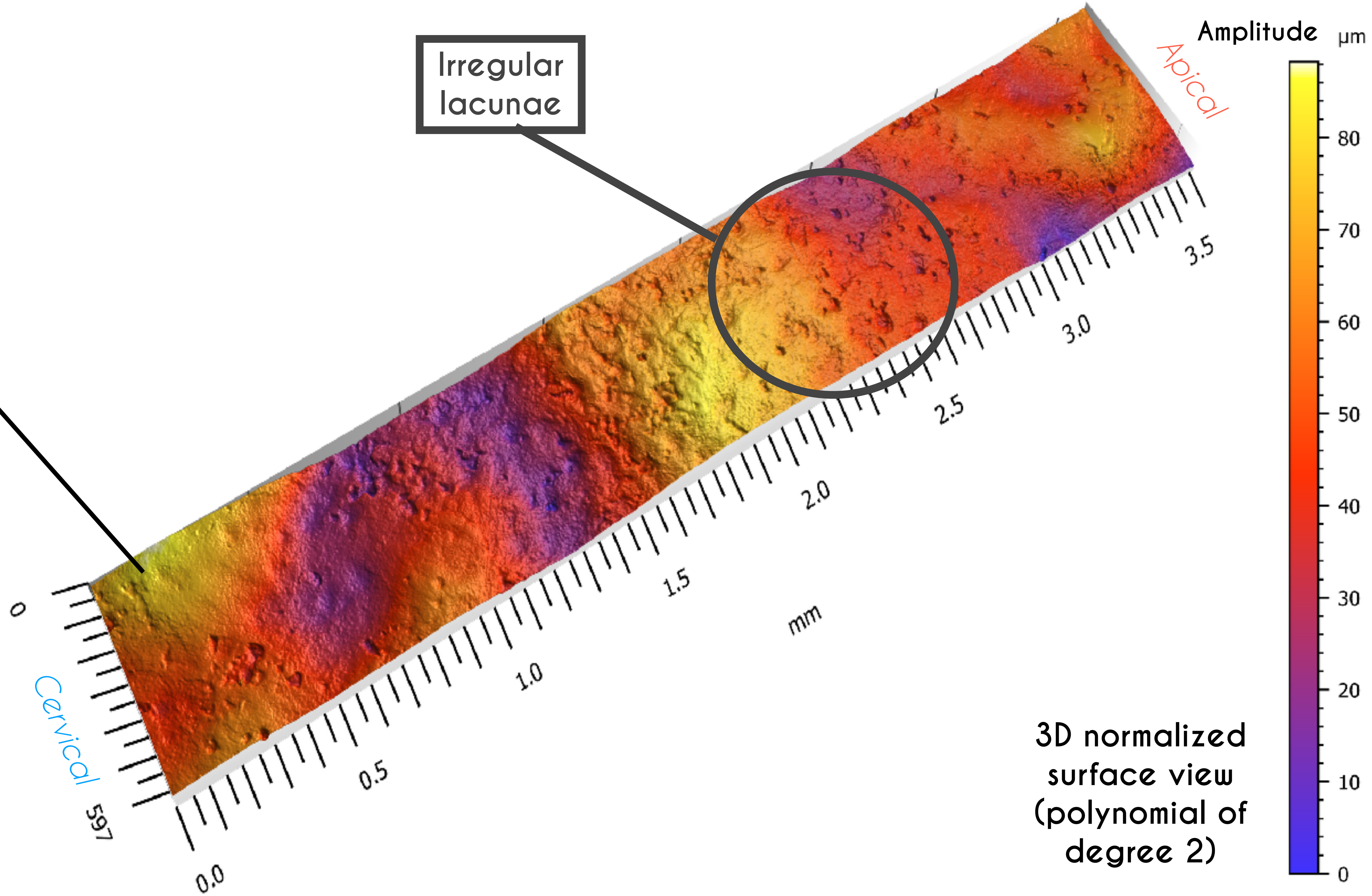

Confocal microscopy - Topography 3D maps			
OF	MAX VE (µm)	ST	Note
2m	90	-R2	IL

OF: other face;
MAX VE: maximum vertical elevation;
ST: surface texture; : scored (**+**) if it is high, (**-**) if it is low; **S:** smooth or **R:** rough; the frequency of occurrence of the elevations scored (**1**) if the frequency is high, (**2**) if the frequency is low.
IL: irregular lacunae

Impacted

Sp114_14

Tooth	Occlusal Wear			CAR Si/Sta	Pulp EXP	IMP	Hypercementosis	Bone Context			
	DEG	DIR	FOR					FEN	CAL	NT	ANTA
**URP3**	**NA**	**NA**	**NA**	**1/4**	**M**	**0**	**3.4.M**	**1**	**0**	**0**	**1**

URP3

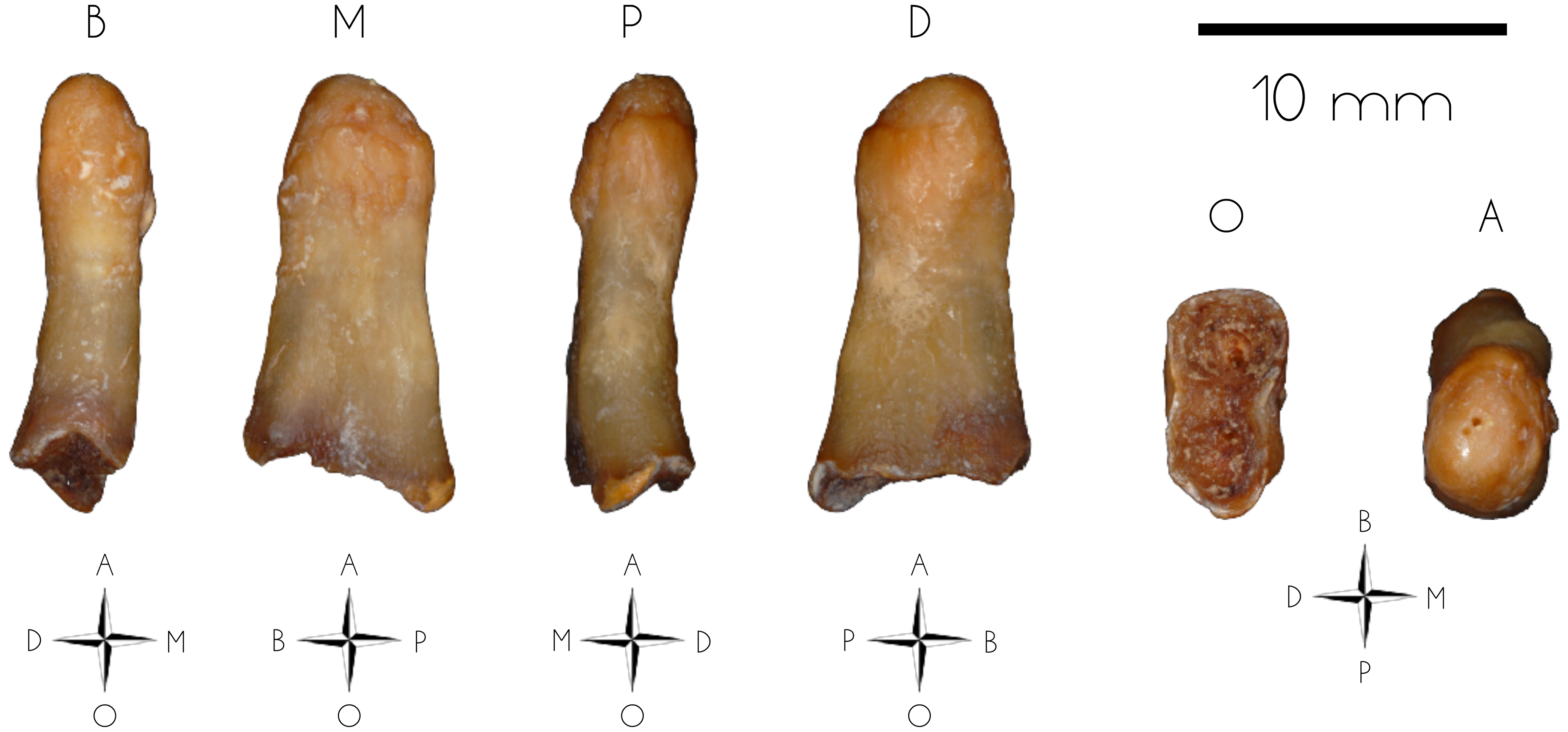

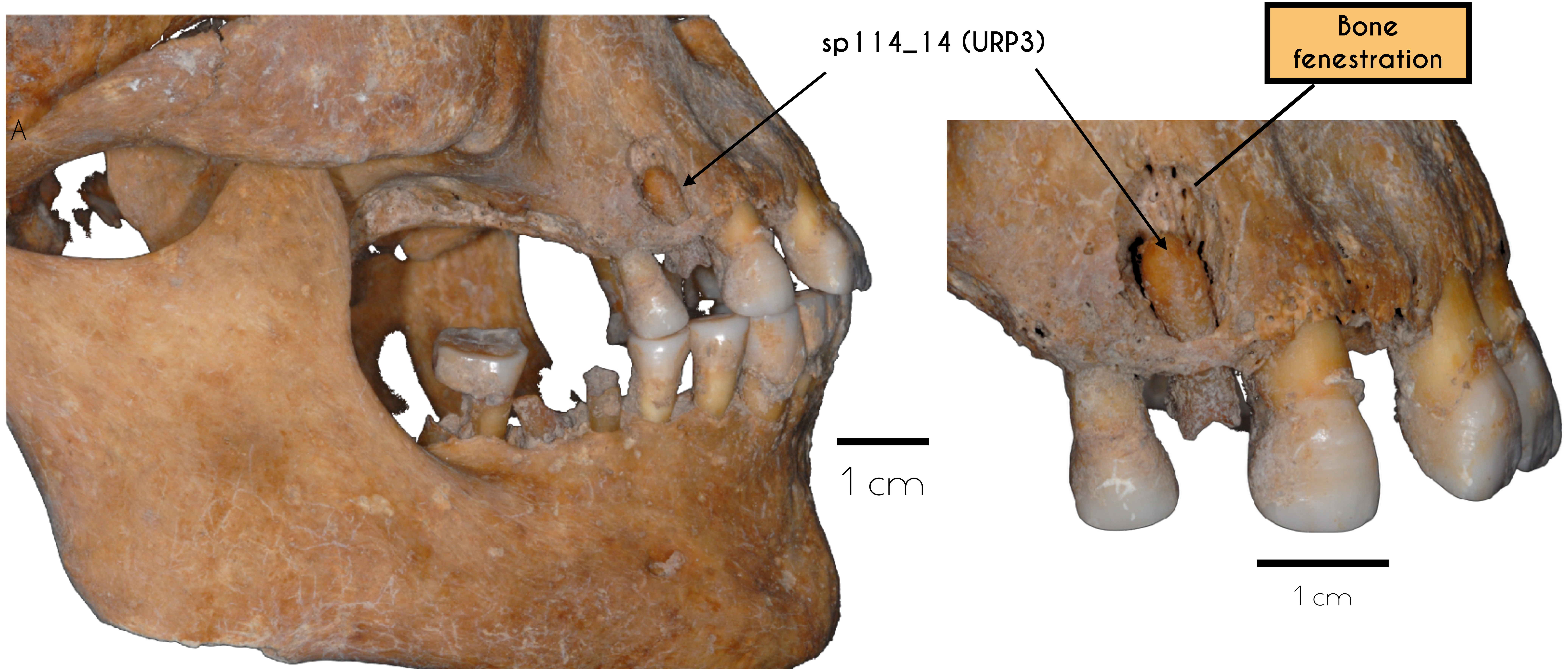

DEG: degree; **DIR:** direction; **FOR:** form - **CAR**: carious lesion - **Pulp EXP:** pulp exposure - **IMP**: impaction - **FEN:** fenestration; **CAL:** calculus; **NT:** tooth loss in neighbouring teeth; **ANTA:** antagonist

Sp114_14

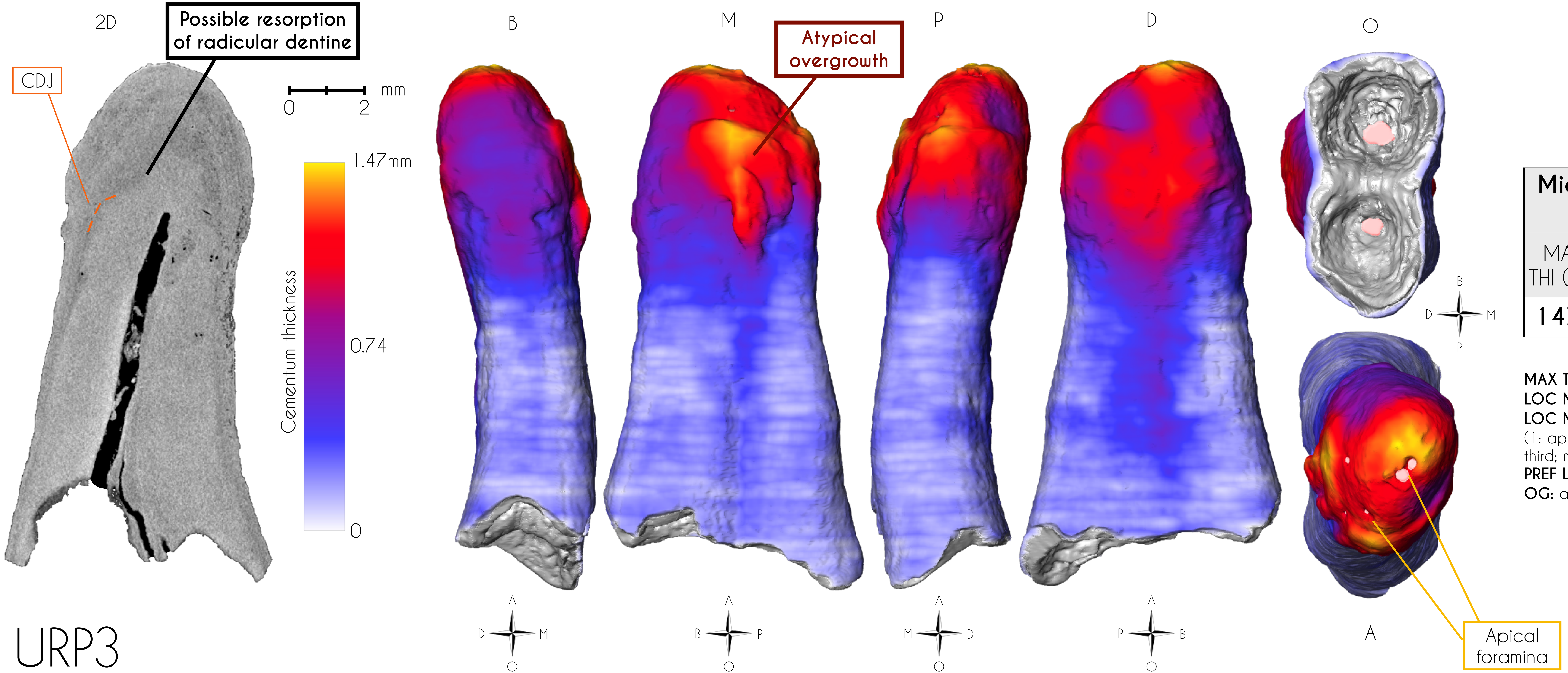

Microtomography - Thickness 3D maps				
MAX THI (μm)	LOC MAX	LOC MIN	PREF LOC	Note
1470	m	<	No	OG

MAX THI: maximum thickness of cementum;
LOC MAX: location of maximum thickness;
LOC MIN: location of minimum thickness;
(1: apical root third; 2: middle root third; 3: cervical root third; m: mesial; d: distal; <: buccal; >: lingual)
PREF LOC: preferential location;
OG: atypical overgrowth

Infected

URP3

Face	Range	Light	Width	Height	Columns/Rows	Factor	Led Color	Gain	Gamma	Overlapping
MP	1032	7.00%	0.88mm	3.63mm	1/6	2	Blue	1.5	1.8	10 %

Sp114_14

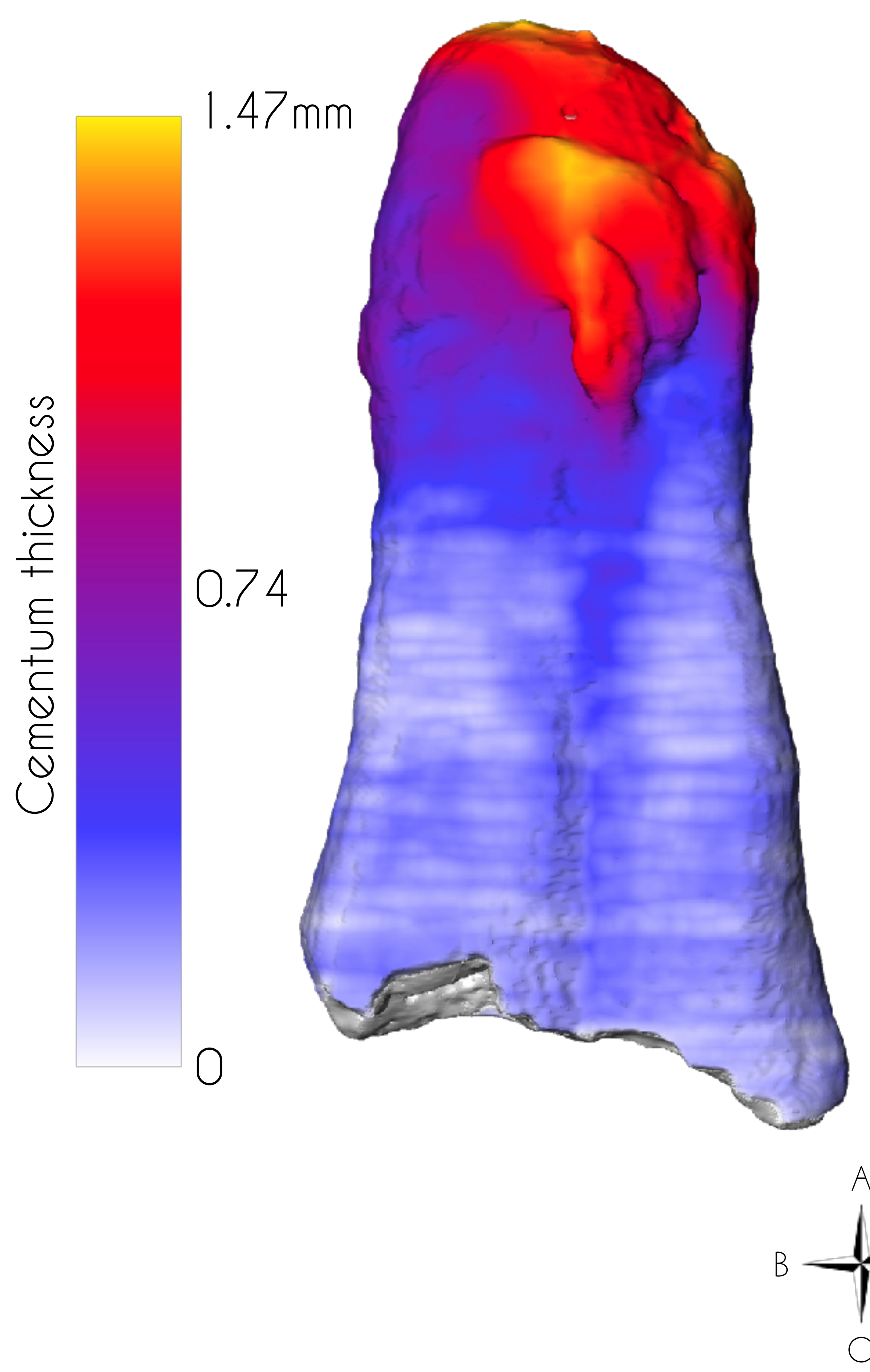

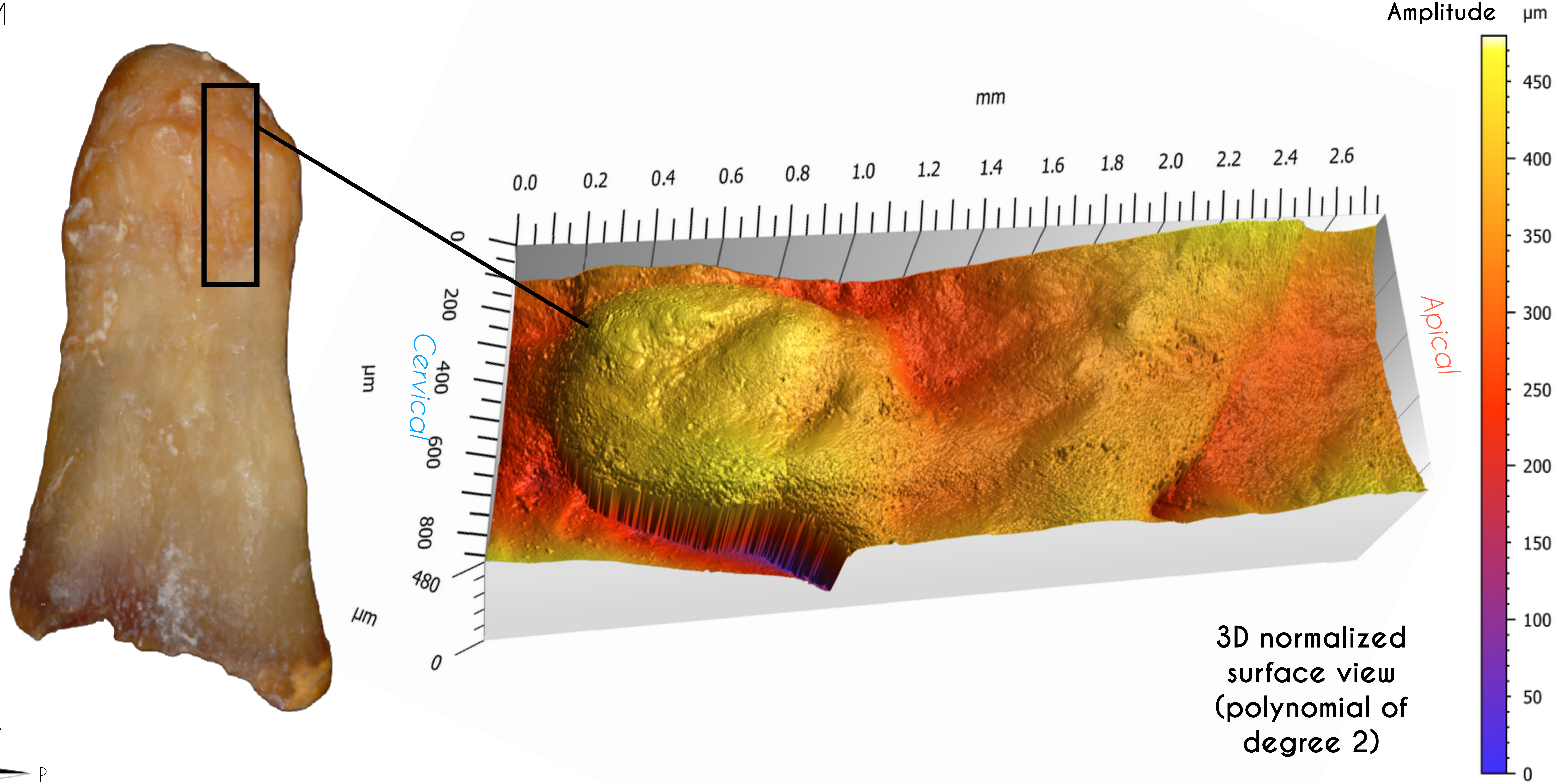

3D normalized surface view (polynomial of degree 2)

Confocal microscopy - Topography 3D maps			
LOC MAX	MAX VE (µm)	ST	Note
m	475	+S2	-

LOC MAX: location of maximum thickness (1: apical root third; 2: middle root third; 3: cervical root third; m: mesial; d: distal; <: buccal; >: lingual);
MAX VE: maximum vertical elevation;
ST: surface texture; : scored (**+**) if it is high, (**-**) if it is low; **S:** smooth or **R:** rough; the frequency of occurrence of the elevations scored (**1**) if the frequency is high, (**2**) if the frequency is low.

Infected

Sp335_23

Tooth	Occlusal Wear			CAR Si/Sta	Pulp EXP	IMP	Hypercementosis	Bone Context			
	DEG	DIR	FOR					FEN	CAL	NT	ANTA
**ULC**	**NA**	**NA**	**NA**	**1/4**	**M**	**0**	**1.4.M**	**1**	**0**	**2**	**1**

ULC

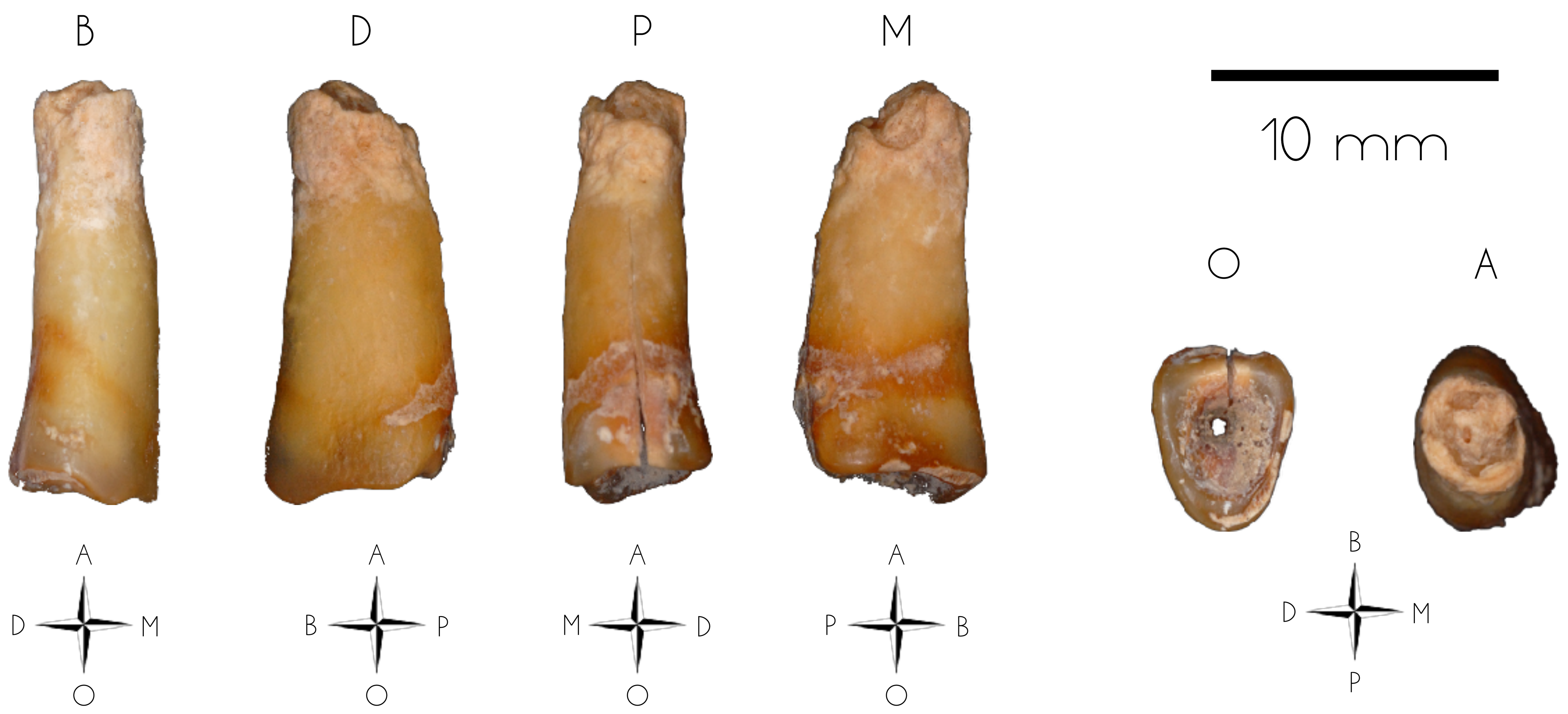

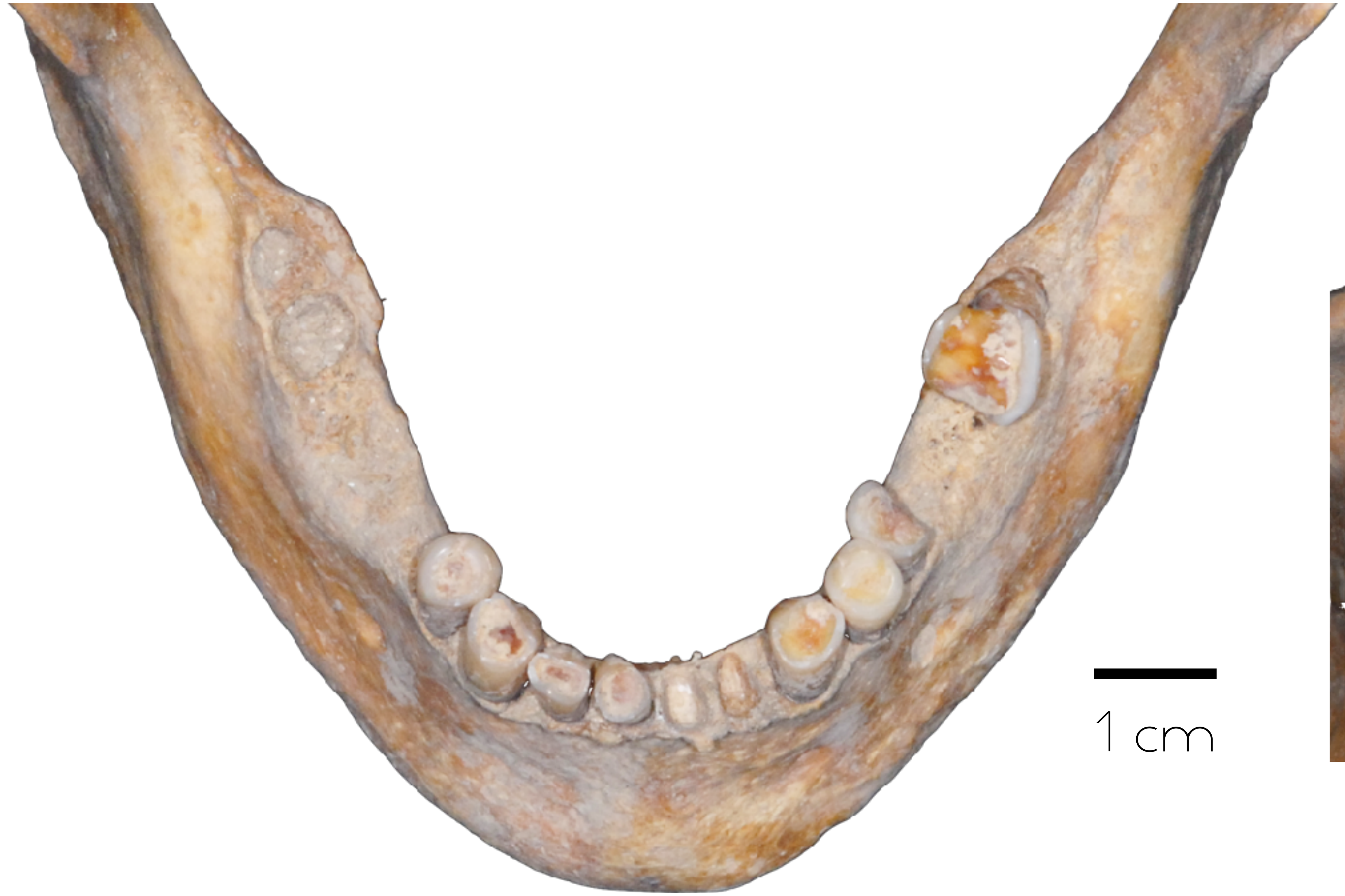

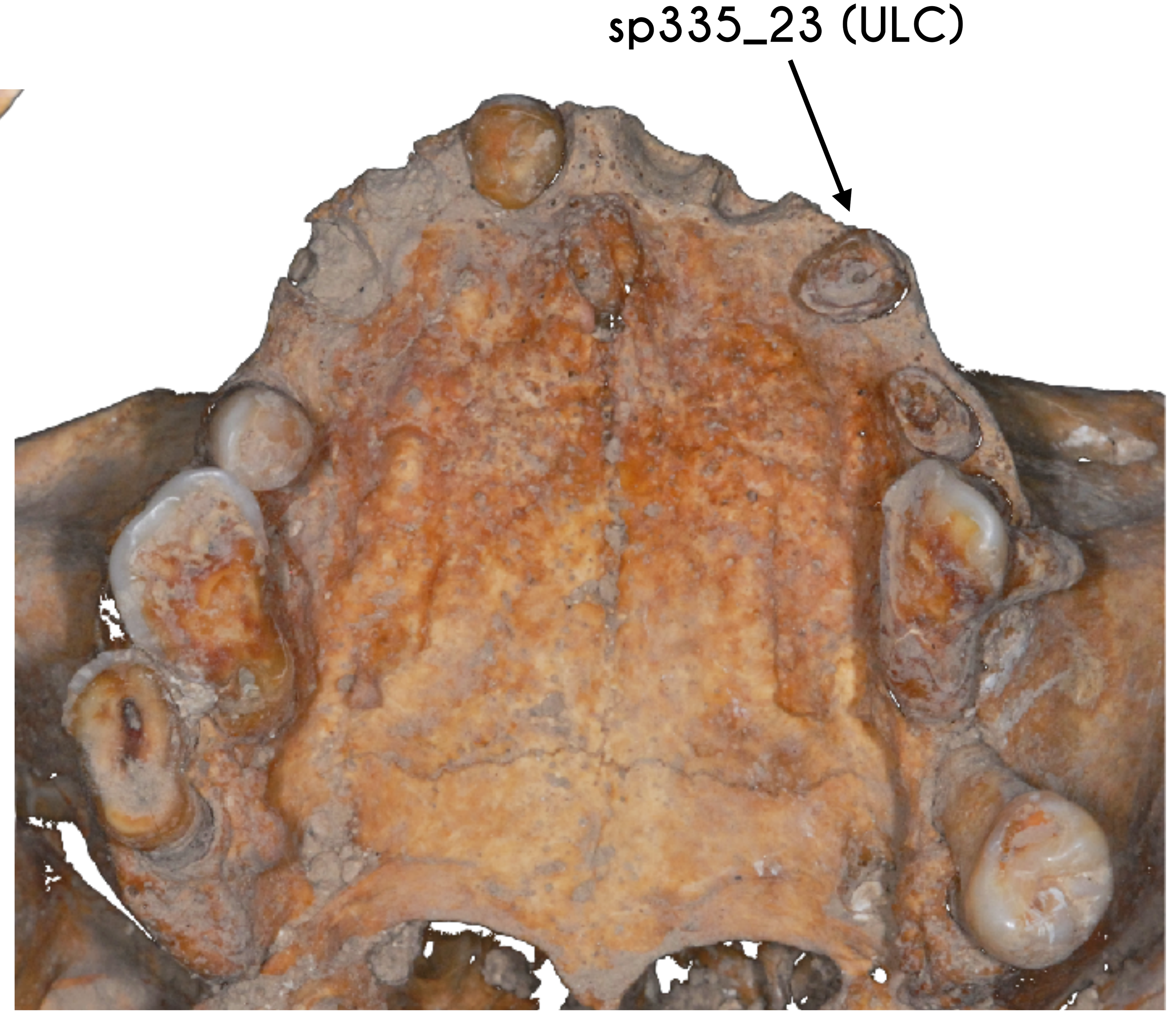

DEG: degree; **DIR:** direction; **FOR:** form - **CAR**: carious lesion - **Pulp EXP:** pulp exposure - **IMP**: impaction - **FEN:** fenestration; **CAL:** calculus; **NT:** tooth loss in neighbouring teeth; **ANTA:** antagonist

Sp335_23

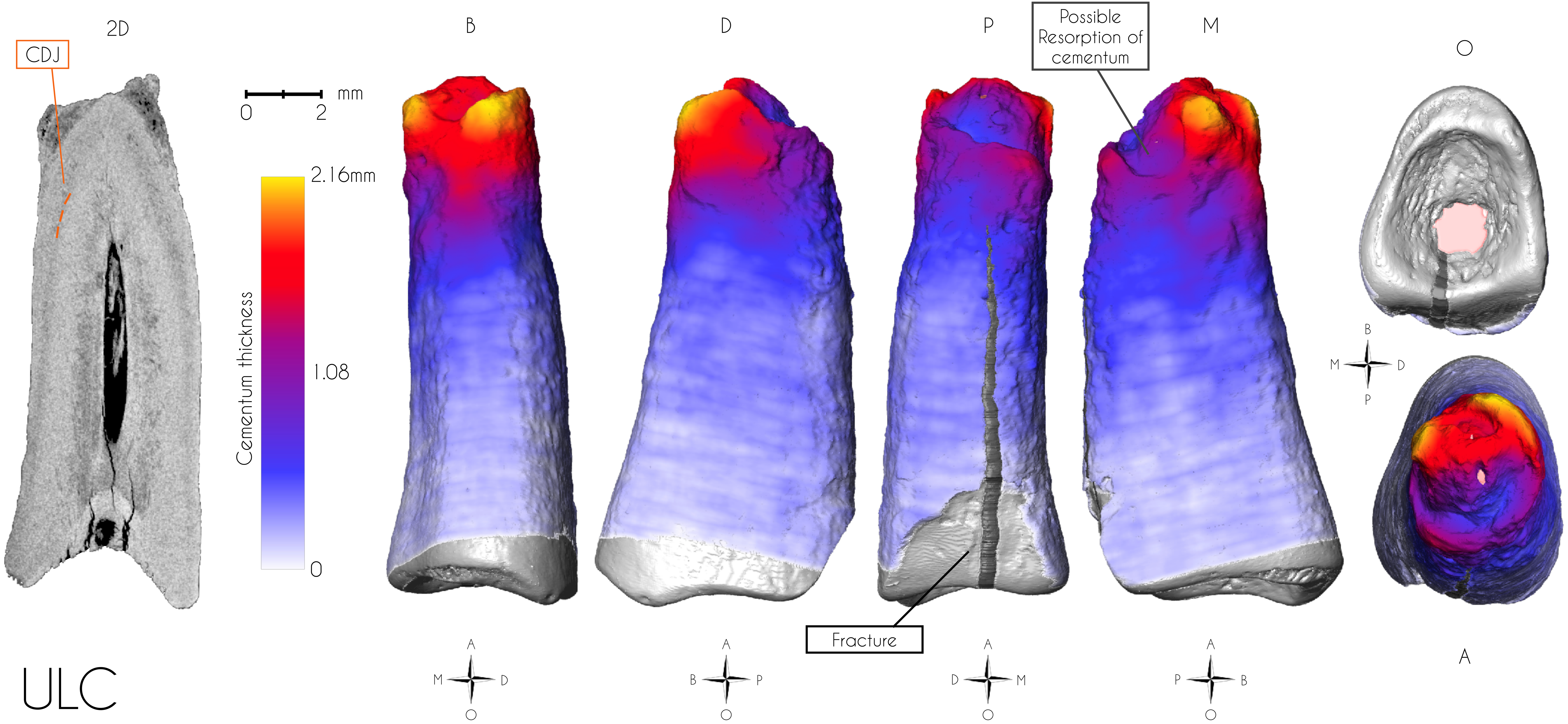

Microtomography - Thickness 3D maps				
MAX THI (µm)	LOC MAX	LOC MIN	PREF LOC	Note
2160	<	>	No	RES (>)

MAX THI: maximum thickness of cementum;
LOC MAX: location of maximum thickness;
LOC MIN: location of minimum thickness;
PREF LOC: preferential location;
RES: resorption;
(<: buccal; >: lingual)

Infected

ULC

Face	Range	Light	Width	Height	Columns/Rows	Factor	Led Color	Gain	Gamma	Overlapping
BD	1206	4.71%	0.88mm	2.44mm	1/4	2	Blue	1.5	1.8	10 %

Sp335_23

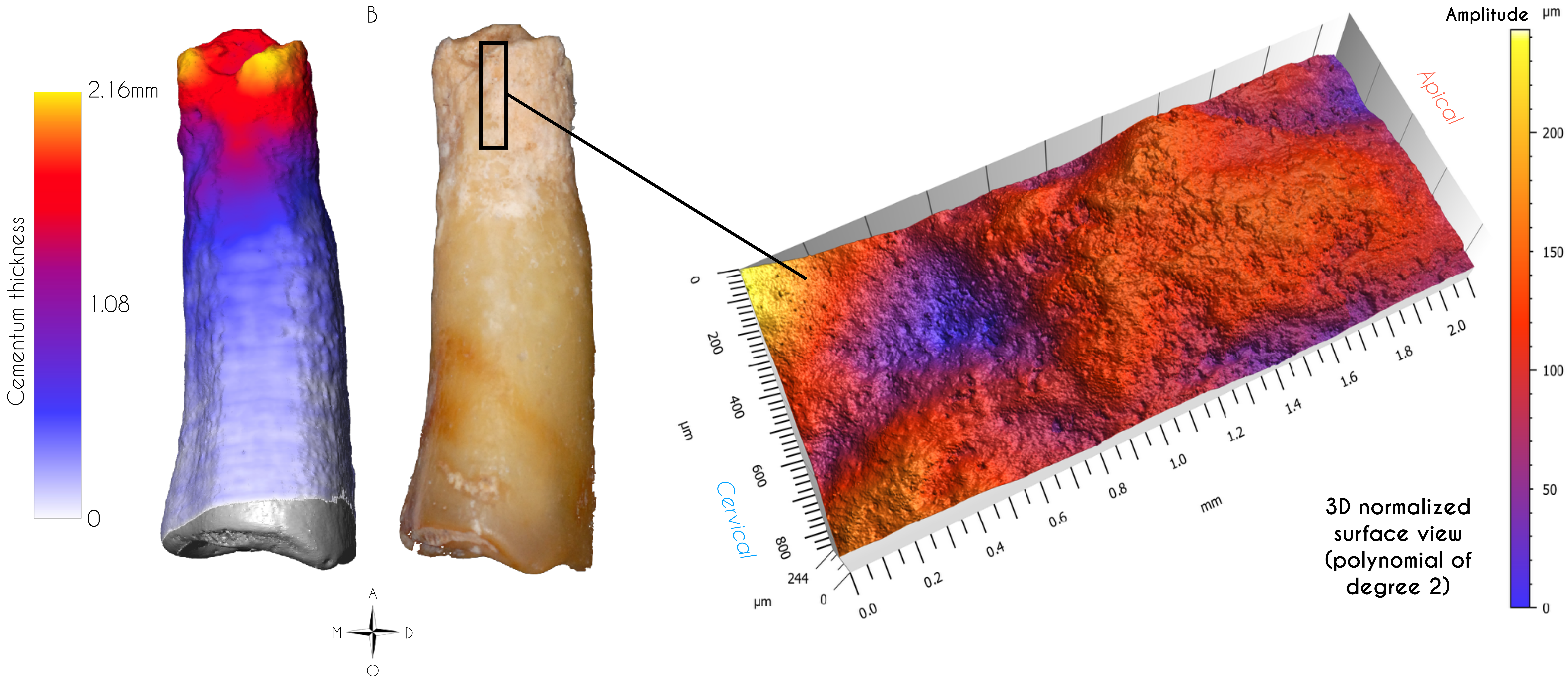

Confocal microscopy - Topography 3D maps			
LOC MAX	MAX VE (µm)	ST	Note
<	250	+R2	-

LOC MAX: location of maximum thickness (1: apical root third; 2: middle root third; 3: cervical root third; m: mesial; d: distal; <: buccal; >: lingual);
MAX VE: maximum vertical elevation;
ST: surface texture; : scored (**+**) if it is high, (**-**) if it is low; **S:** smooth or **R:** rough; the frequency of occurrence of the elevations scored (**1**) if the frequency is high, (**2**) if the frequency is low.

Infected

Sp914_43

Tooth	Occlusal Wear			CAR Si/Sta	Pulp EXP	IMP	Hypercementosis	Bone Context			
	DEG	DIR	FOR					FEN	CAL	NT	ANTA
LRC	NA	NA	NA	1/4	M	0	1.4.m	0	0	0	NA

LRC

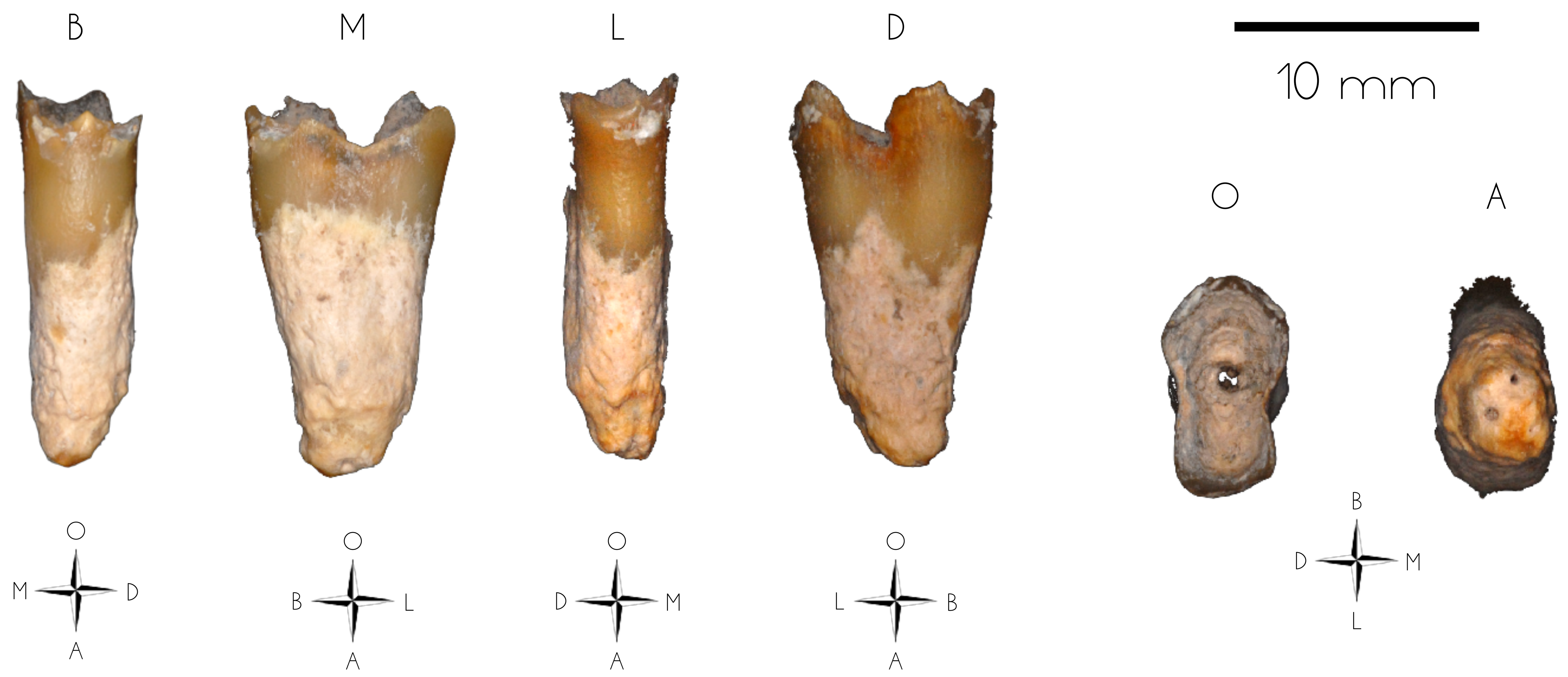

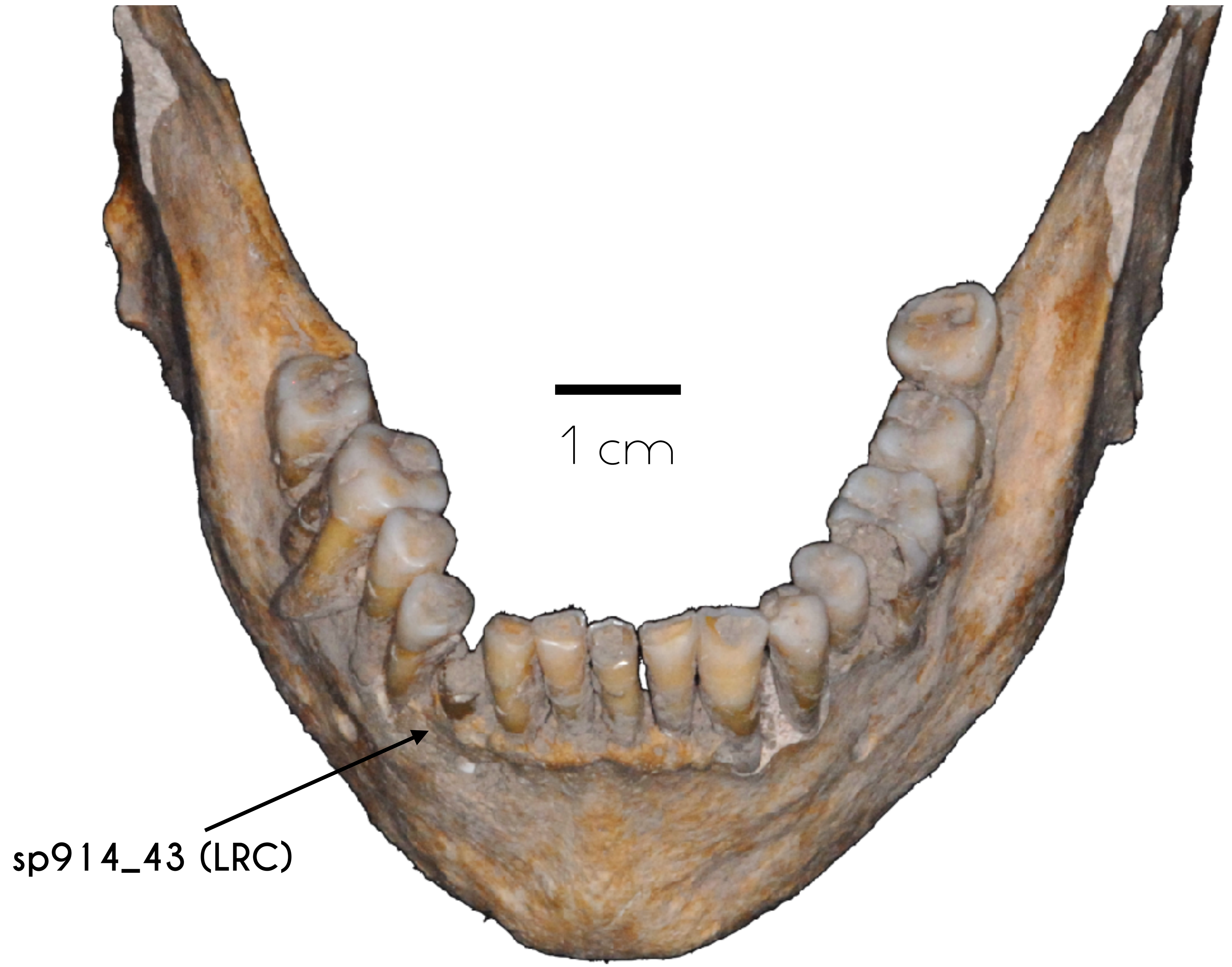

DEG: degree; **DIR:** direction; **FOR:** form - **CAR**: carious lesion - **Pulp EXP:** pulp exposure - **IMP**: impaction - **FEN:** fenestration; **CAL:** calculus; **NT:** tooth loss in neighbouring teeth; **ANTA:** antagonist

Sp914_43

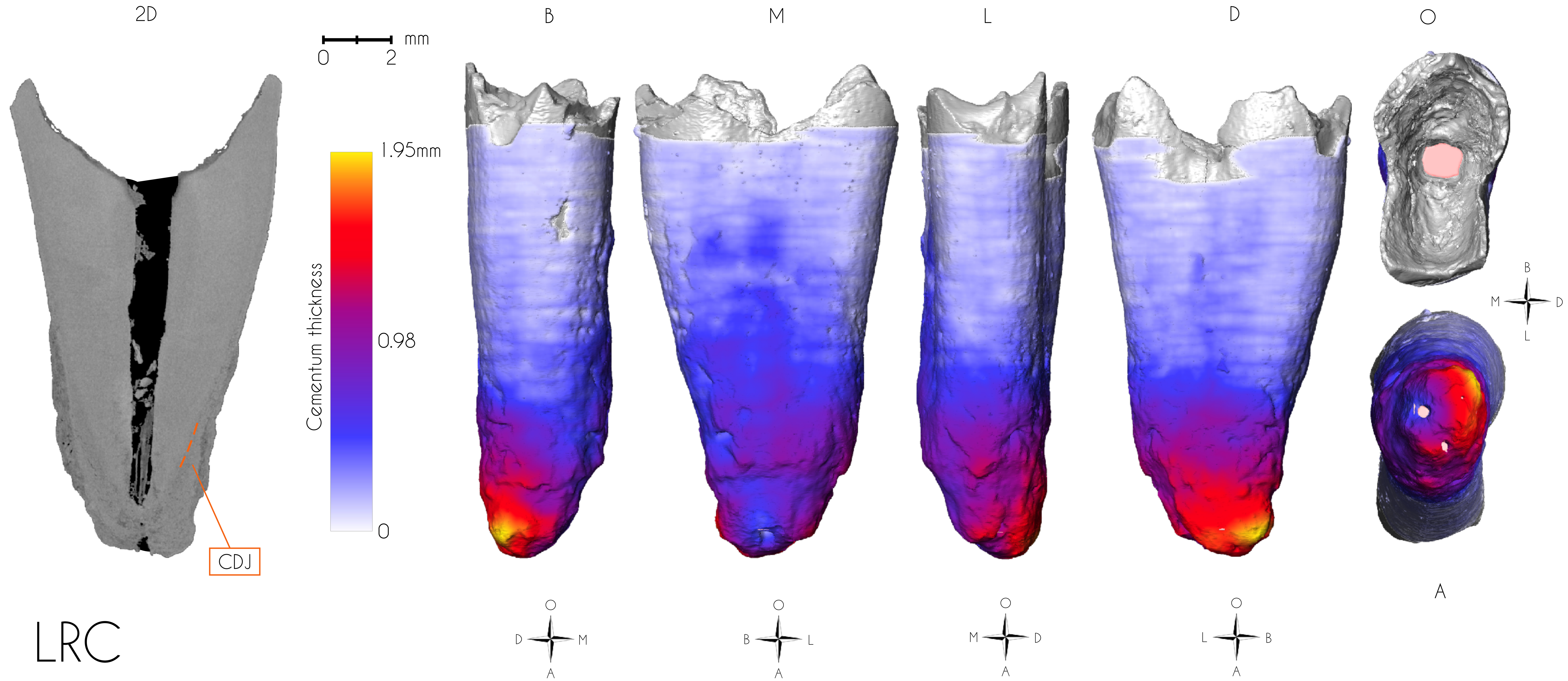

Microtomography - Thickness 3D maps				
MAX THI (μm)	LOC MAX	LOC MIN	PREF LOC	Note
1950	d	m	No	-

MAX THI: maximum thickness of cementum;
LOC MAX: location of maximum thickness;
LOC MIN: location of minimum thickness;
(m: mesial; d: distal)
PREF LOC: preferential location;

Infected

LRC

Face	Range	Light	Width	Height	Columns/Rows	Factor	Led Color	Gain	Gamma	Overlapping
BD	652	6.28%	0.88mm	3.63mm	1/6	2	Blue	1.47	1.61	10 %

Sp914_43

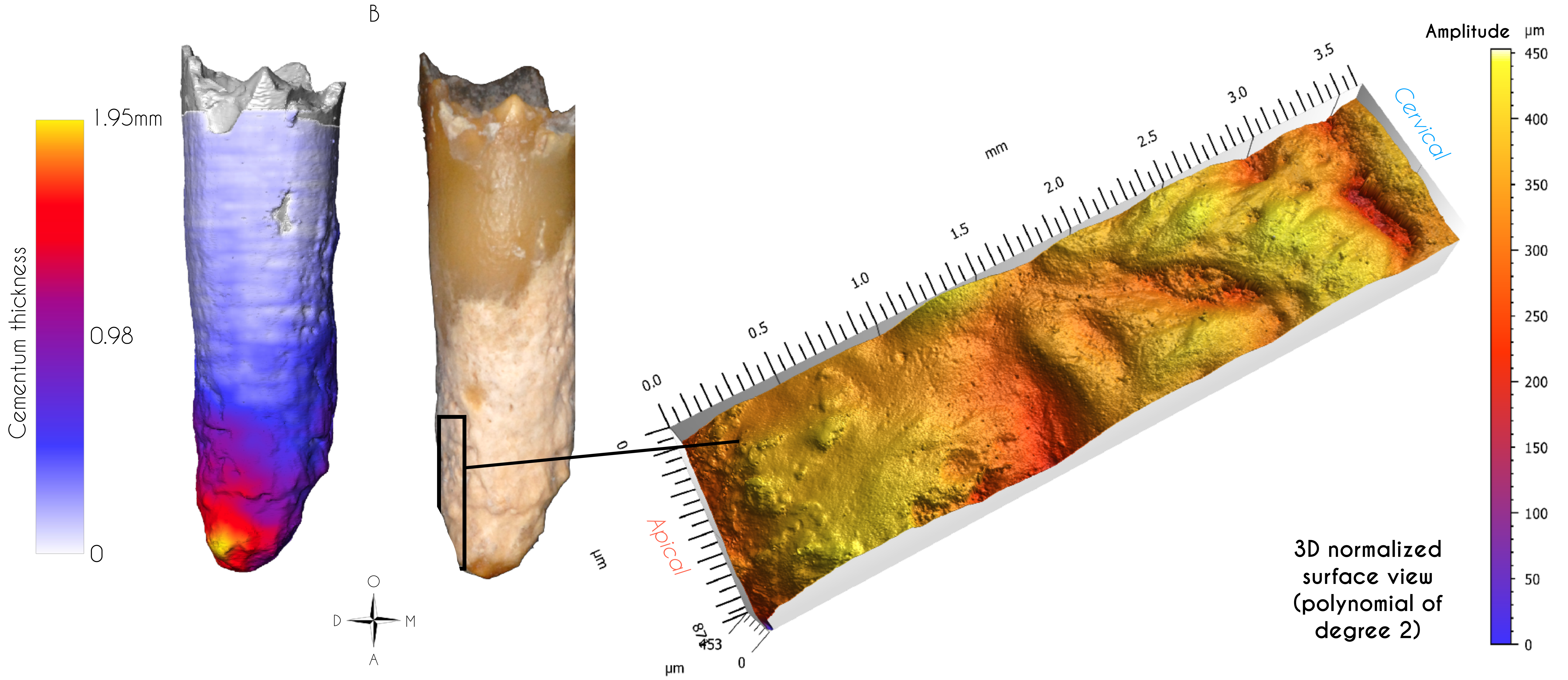

Confocal microscopy - Topography 3D maps			
LOC MAX	MAX VE (µm)	ST	Note
D	450	+S2	-

LOC MAX: location of maximum thickness (1: apical root third; 2: middle root third; 3: cervical root third; m: mesial; d: distal; <: buccal; >: lingual);
MAX VE: maximum vertical elevation;
ST: surface texture; : scored **(+)** if it is high, **(-)** if it is low; **S:** smooth or **R:** rough; the frequency of occurrence of the elevations scored **(1)** if the frequency is high, **(2)** if the frequency is low.

Infected

Sp1172_15

Tooth	Occlusal Wear			CAR Si/Sta	Pulp EXP	IMP	Hypercementosis	Bone Context			
	DEG	DIR	FOR					FEN	CAL	NT	ANTA
**URP4**	**NA**	**NA**	**NA**	**1/4**	**M**	**0**	**1.4.M**	**0**	**0**	**1d**	**1**

URP4

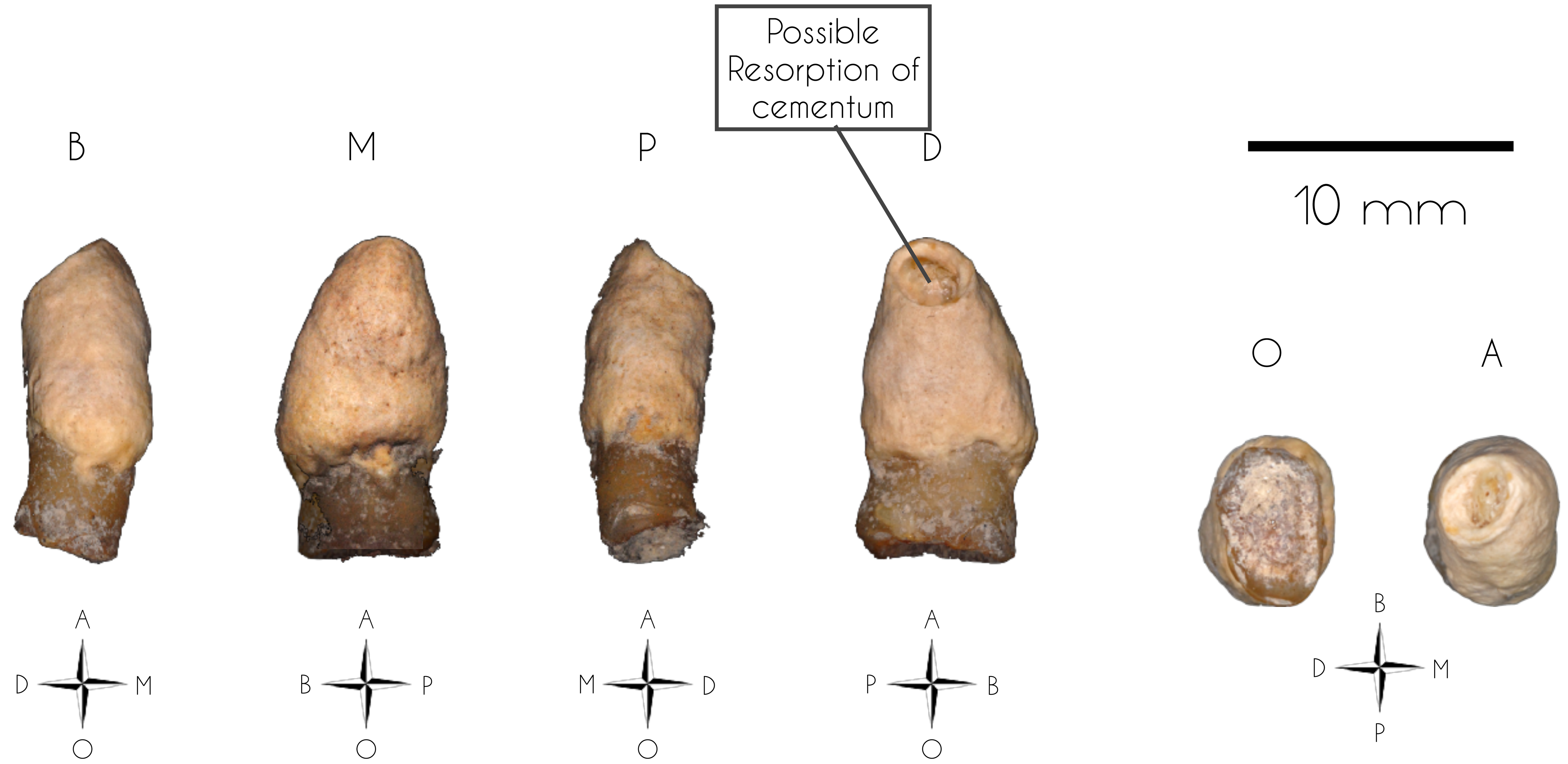

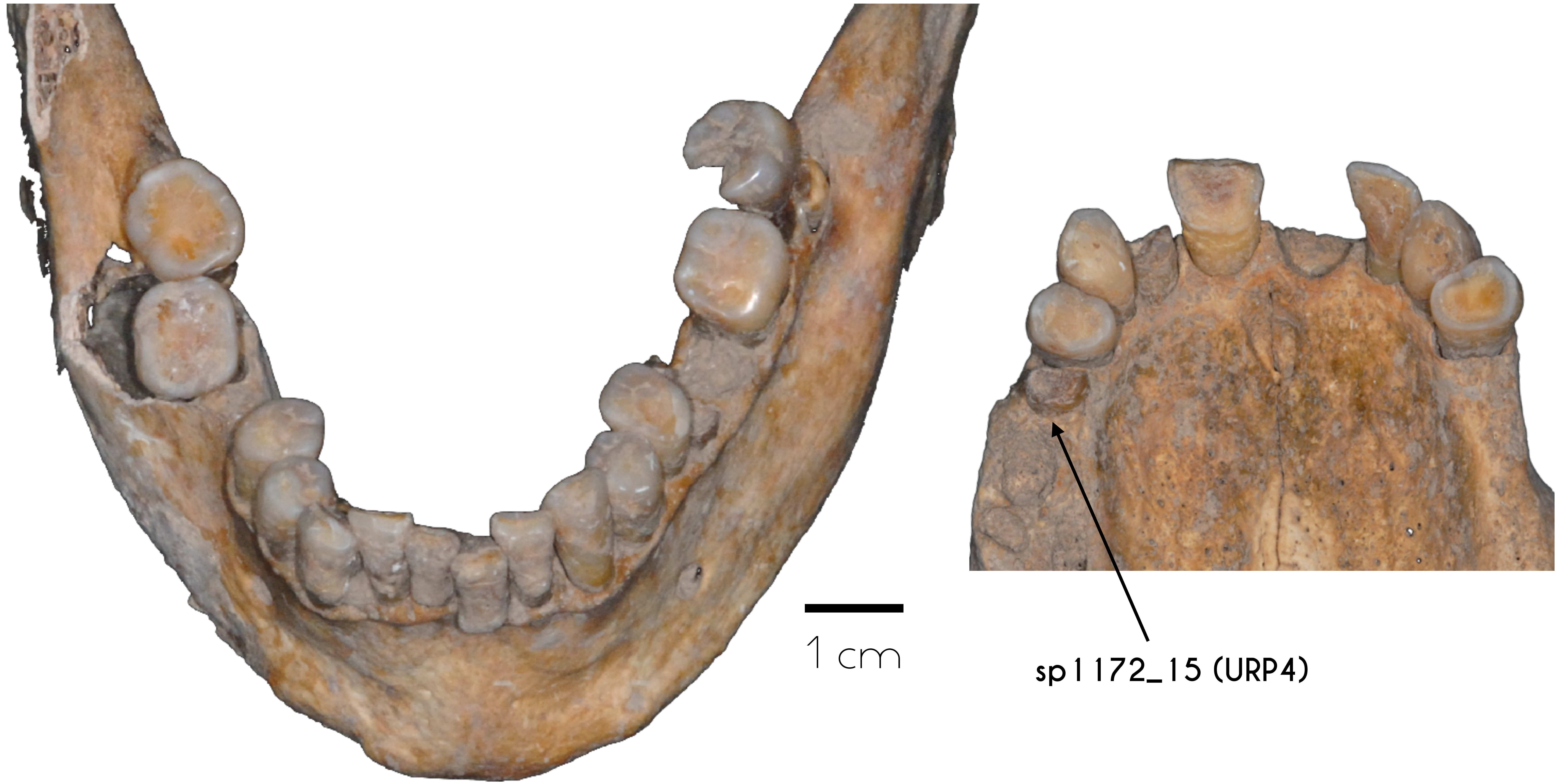

DEG: degree; **DIR:** direction; **FOR:** form - **CAR**: carious lesion - **Pulp EXP:** pulp exposure - **IMP**: impaction - **FEN:** fenestration; **CAL:** calculus; **NT:** tooth loss in neighbouring teeth; **ANTA:** antagonist

Sp1172_15

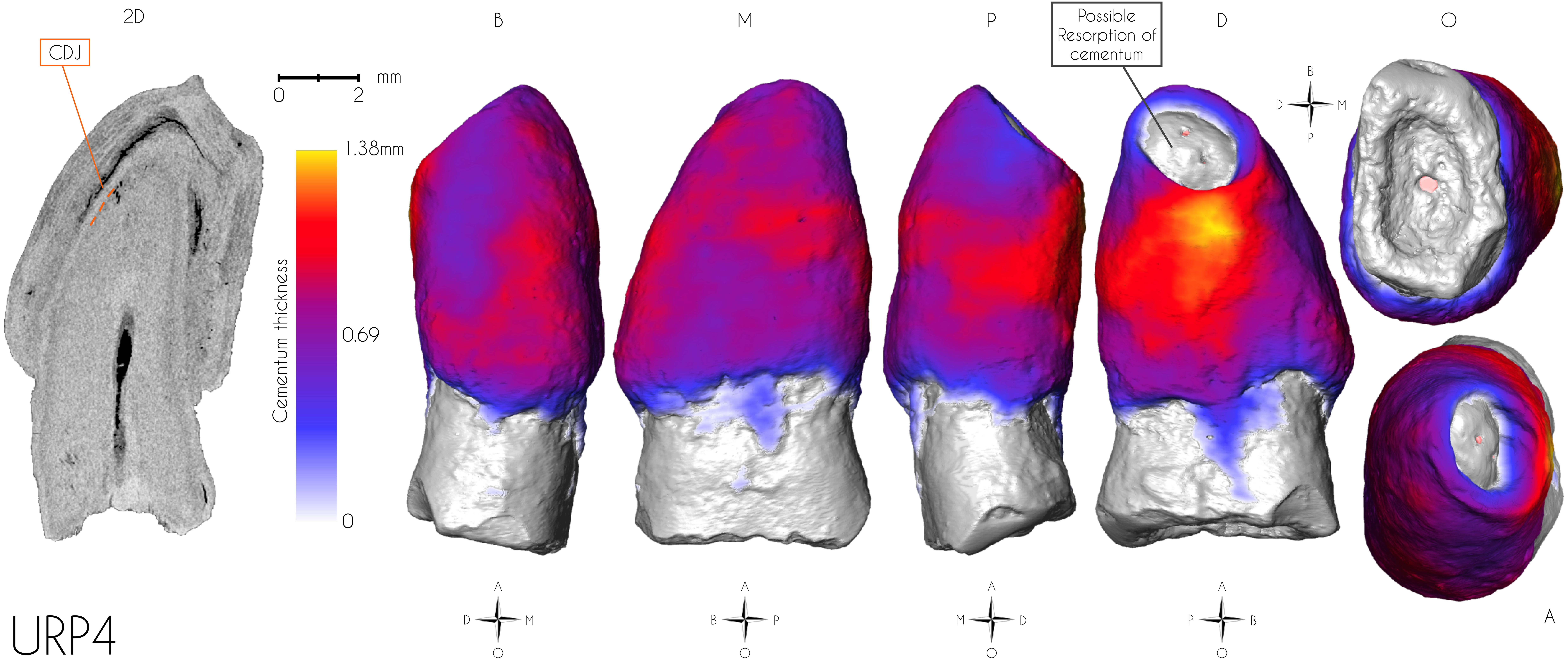

URP4

Microtomography - Thickness 3D maps				
MAX THI (µm)	LOC MAX	LOC MIN	PREF LOC	Note
1380	d	No	No	RES (d)

MAX THI: maximum thickness of cementum;
LOC MAX: location of maximum thickness;
LOC MIN: location of minimum thickness;
PREF LOC: preferential location;
RES: resorption;
(d: distal)

Infected

URP4

Face	Range	Light	Width	Height	Columns/Rows	Factor	Led Color	Gain	Gamma	Overlapping
D	523	5.50%	1.67mm	2.44mm	2/4	2	Blue	1.47	1.61	10 %

Sp1172_15

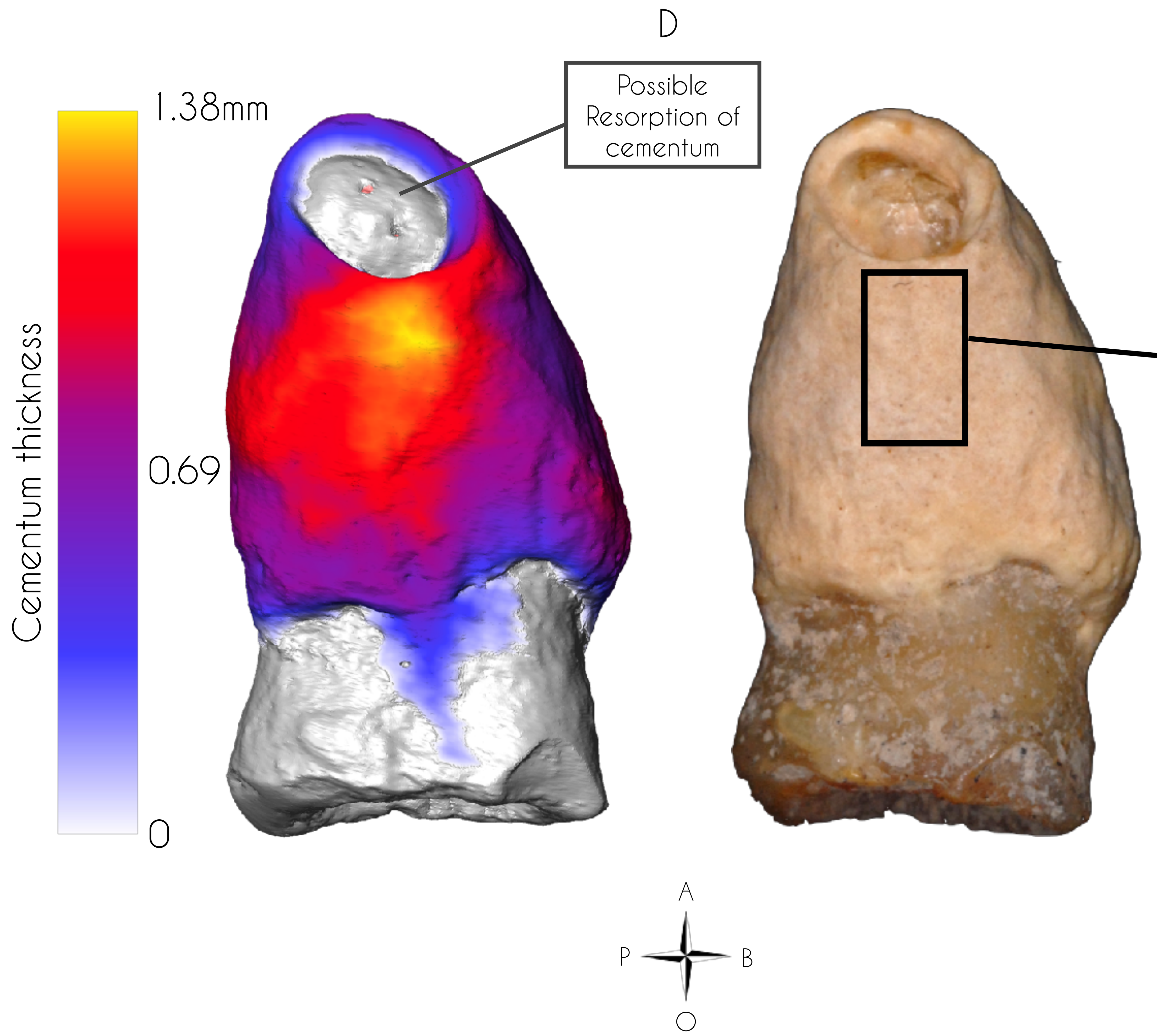

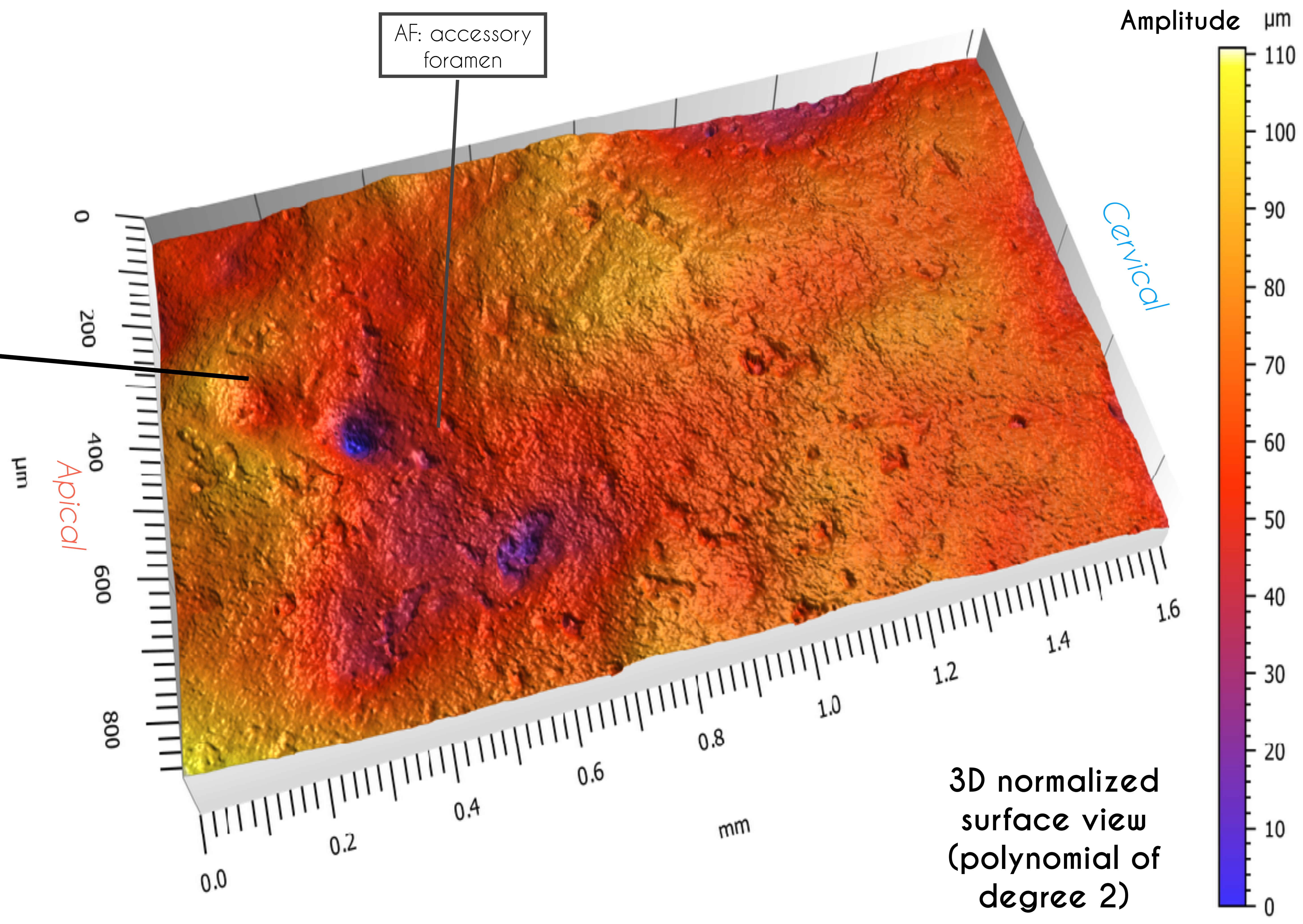

Confocal microscopy - Topography 3D maps			
LOC MAX	MAX VE (µm)	ST	Note
D	110	-S2	AF

LOC MAX: location of maximum thickness (1: apical root third; 2: middle root third; 3: cervical root third; m: mesial; d: distal; <: buccal; >: lingual);
MAX VE: maximum vertical elevation;
ST: surface texture; : scored (**+**) if it is high, (**-**) if it is low; **S:** smooth or **R:** rough; the frequency of occurrence of the elevations scored (**1**) if the frequency is high, (**2**) if the frequency is low.
AF: accessory foramen

Infected

URP4

Face	Range	Light	Width	Height	Columns/Rows	Factor	Led Color	Gain	Gamma	Overlapping
M	435	5.50%	0.88mm	4.82mm	1/5	2	Blue	1.47	1.61	10 %

Sp1172_15

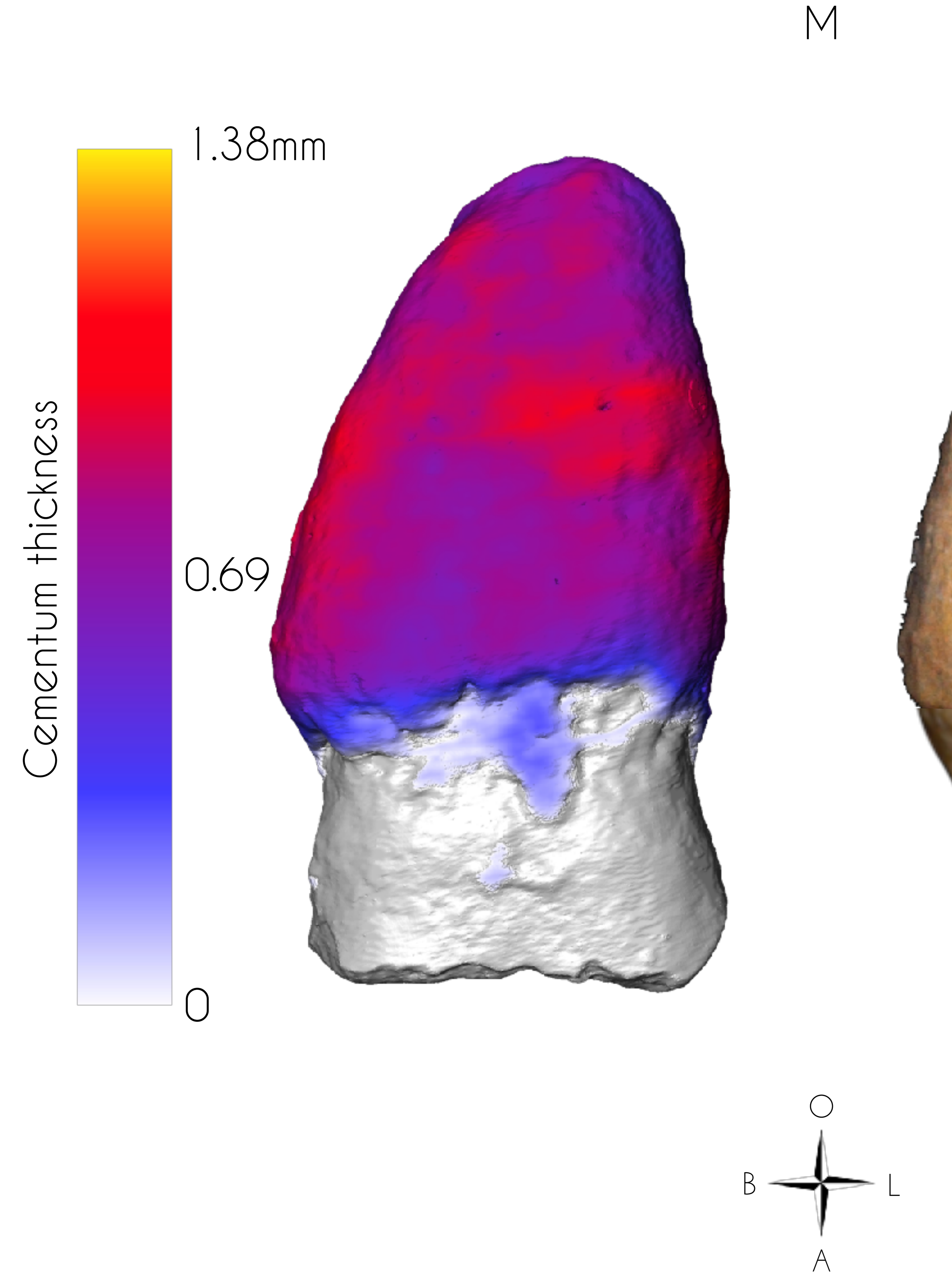

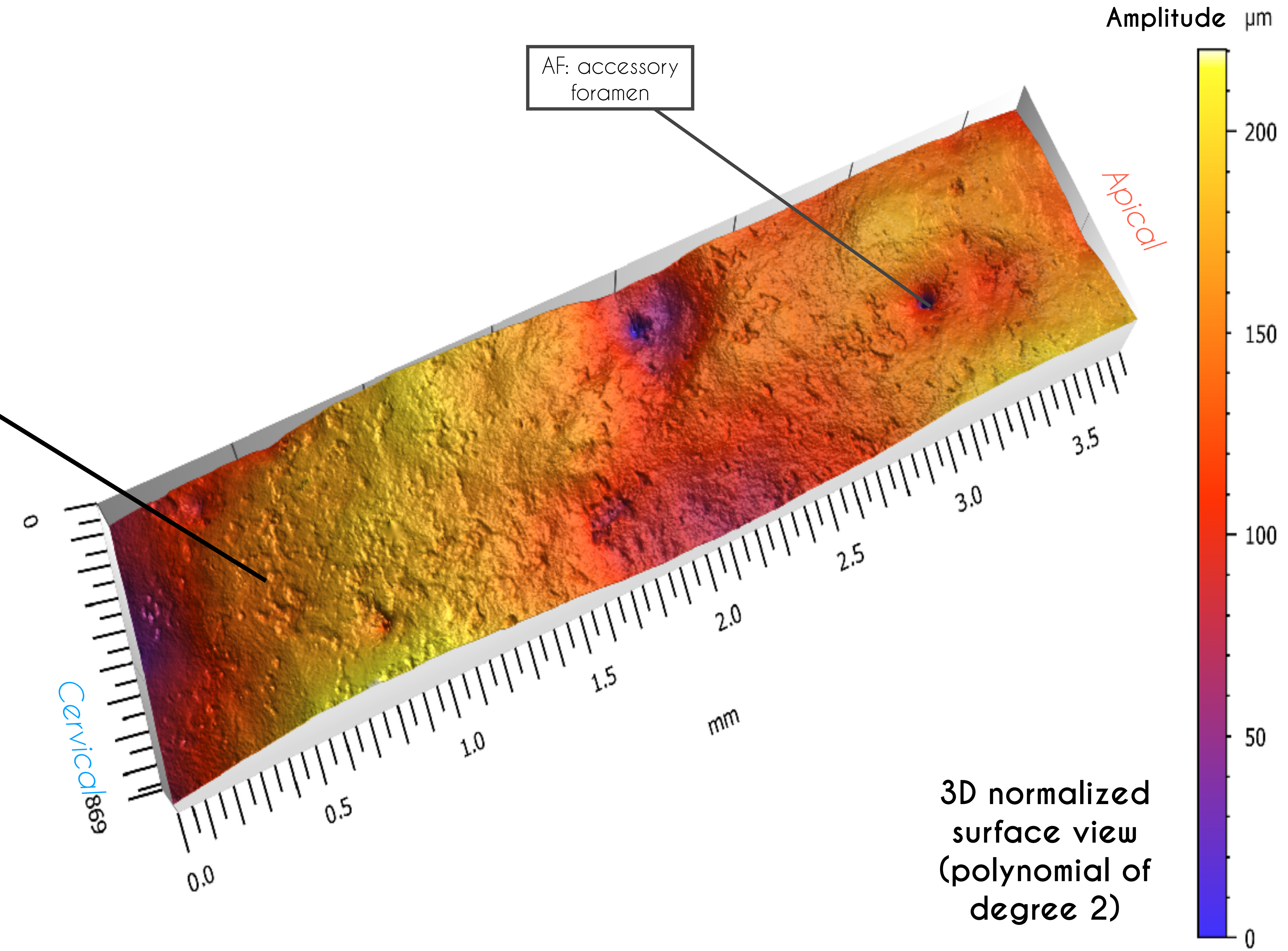

3D normalized surface view (polynomial of degree 2)

Confocal microscopy - Topography 3D maps			
OF	MAX VE (µm)	ST	Note
M	220	-S2	AF

OF: other face;
MAX VE: maximum vertical elevation;
ST: surface texture; : scored (**+**) if it is high, (**-**) if it is low; **S:** smooth or **R:** rough; the frequency of occurrence of the elevations scored (**1**) if the frequency is high, (**2**) if the frequency is low.
AF: accessory foramen

Infected

Sp17_15

Tooth	Occlusal Wear			CAR Si/Sta	Pulp EXP	IMP	Hypercementosis	Bone Context			
	DEG	DIR	FOR					FEN	CAL	NT	ANTA
**URP4**	3	4	2	-	0	0	1.2.m	0	0	1d	1

URP4

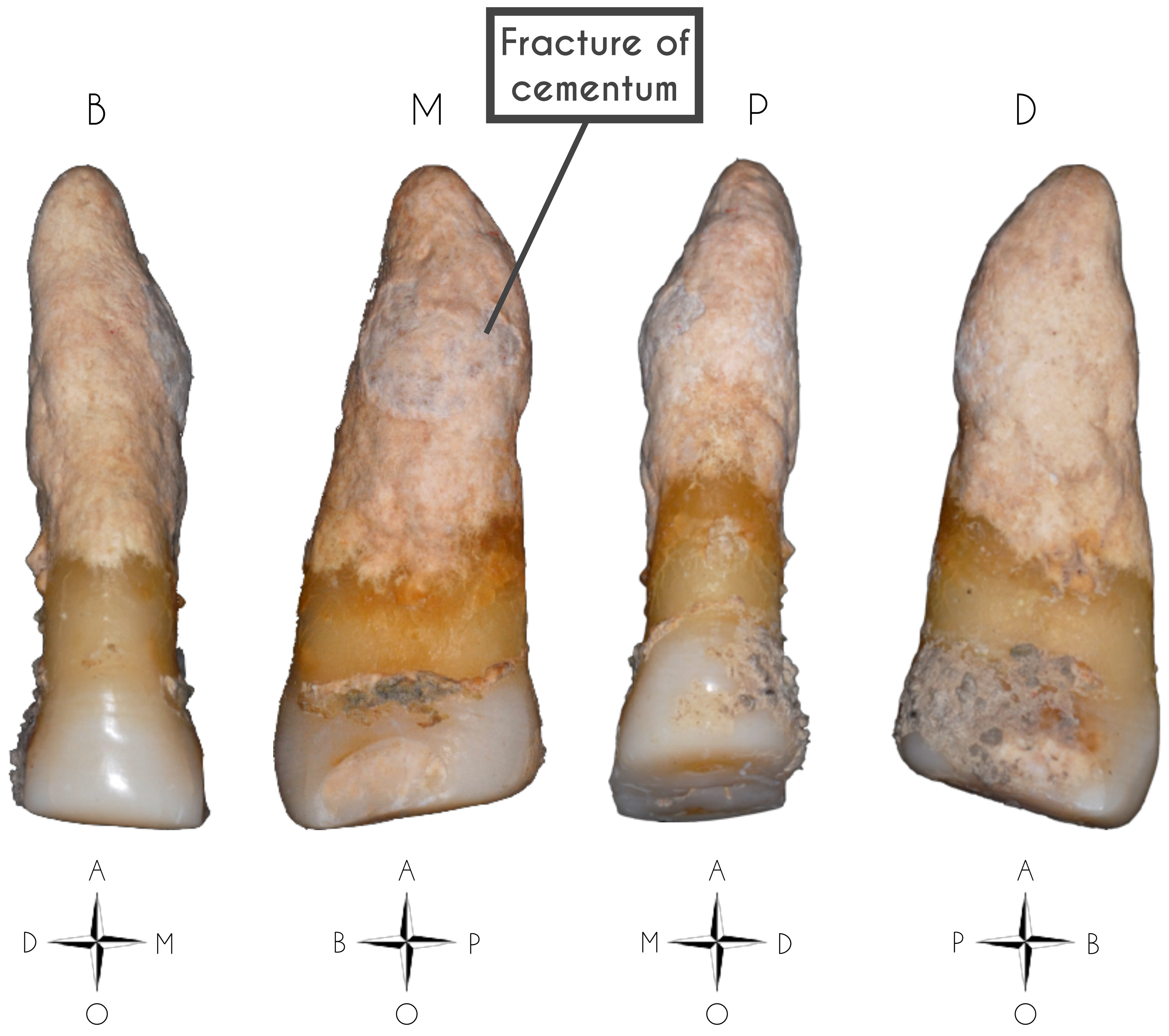

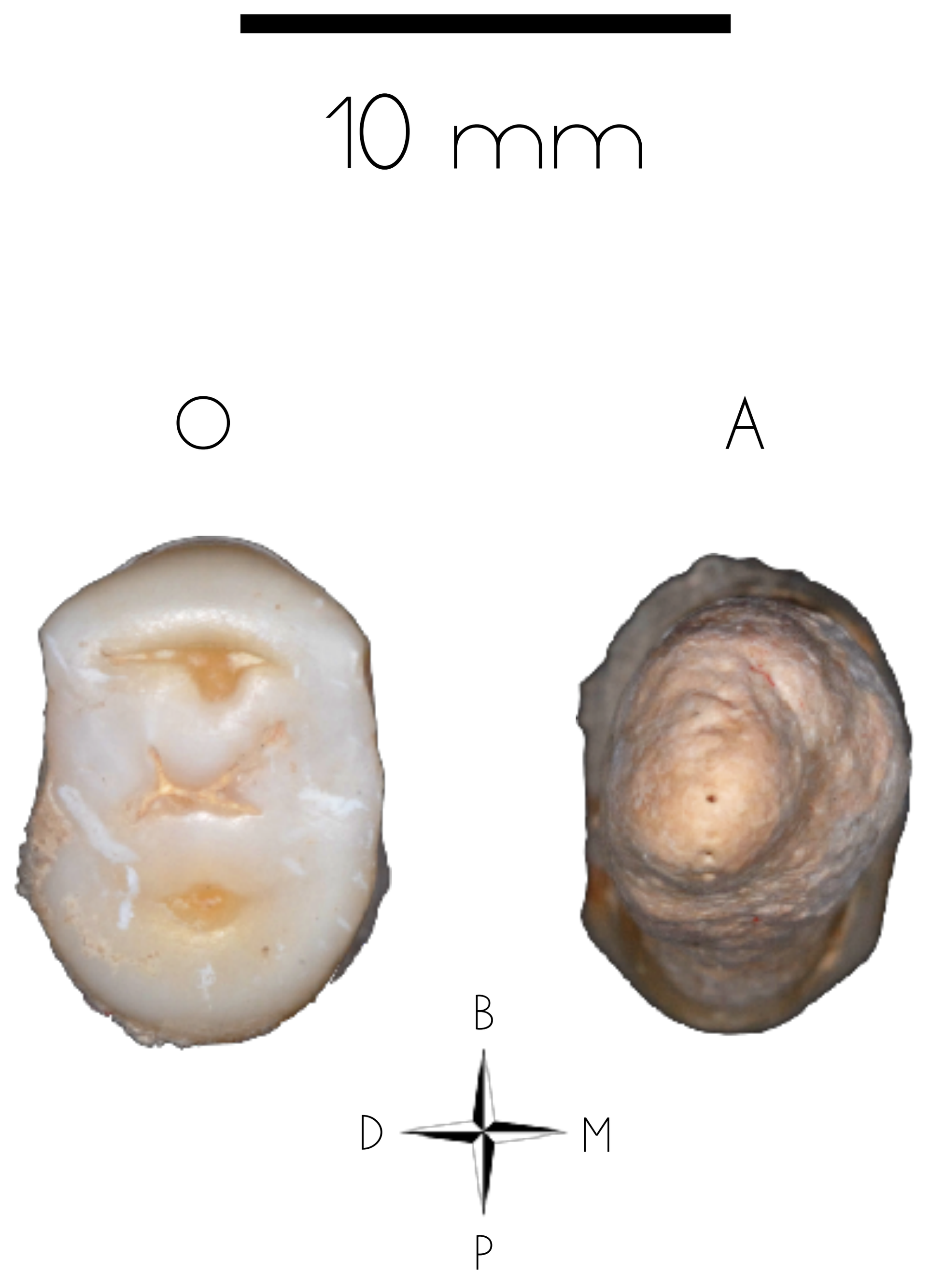

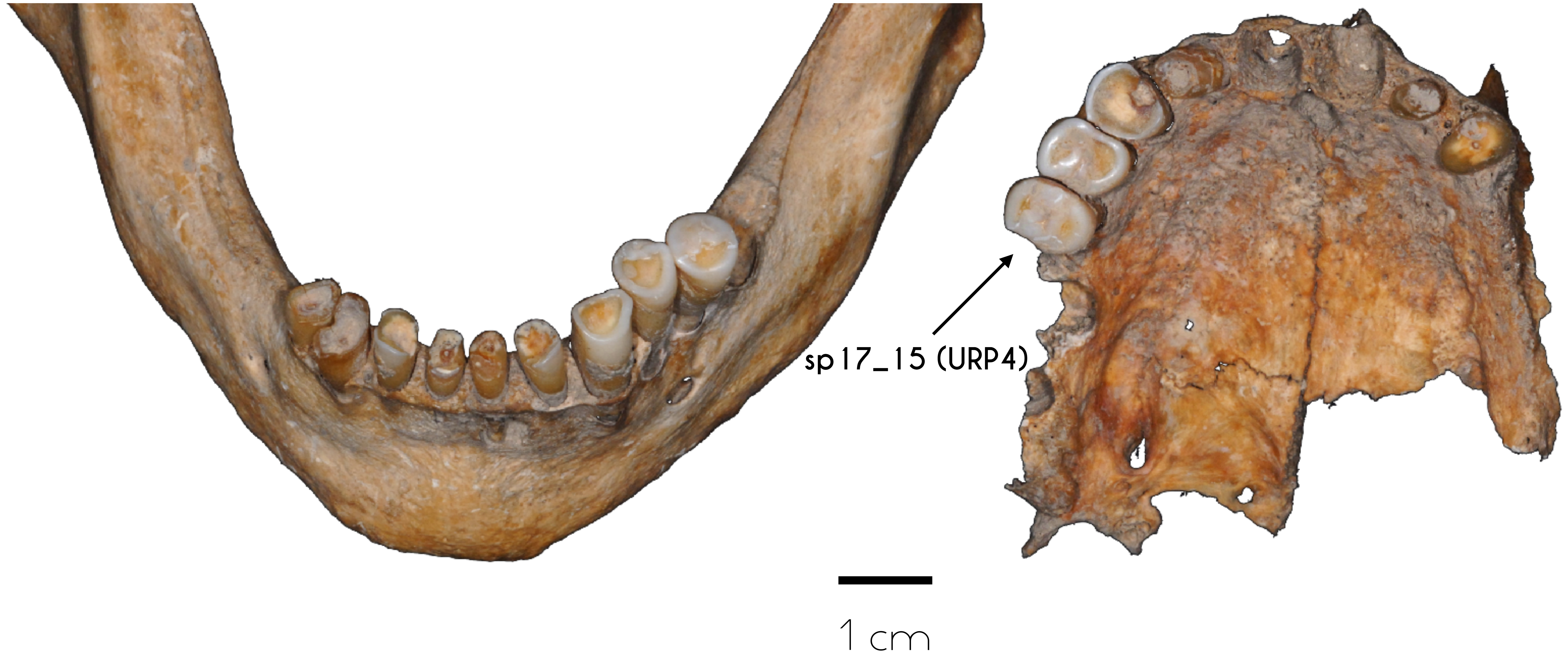

DEG: degree; **DIR:** direction; **FOR:** form - **CAR**: carious lesion - **Pulp EXP:** pulp exposure - **IMP**: impaction - **FEN:** fenestration; **CAL:** calculus; **NT:** tooth loss in neighbouring teeth; **ANTA:** antagonist

Sp17_15

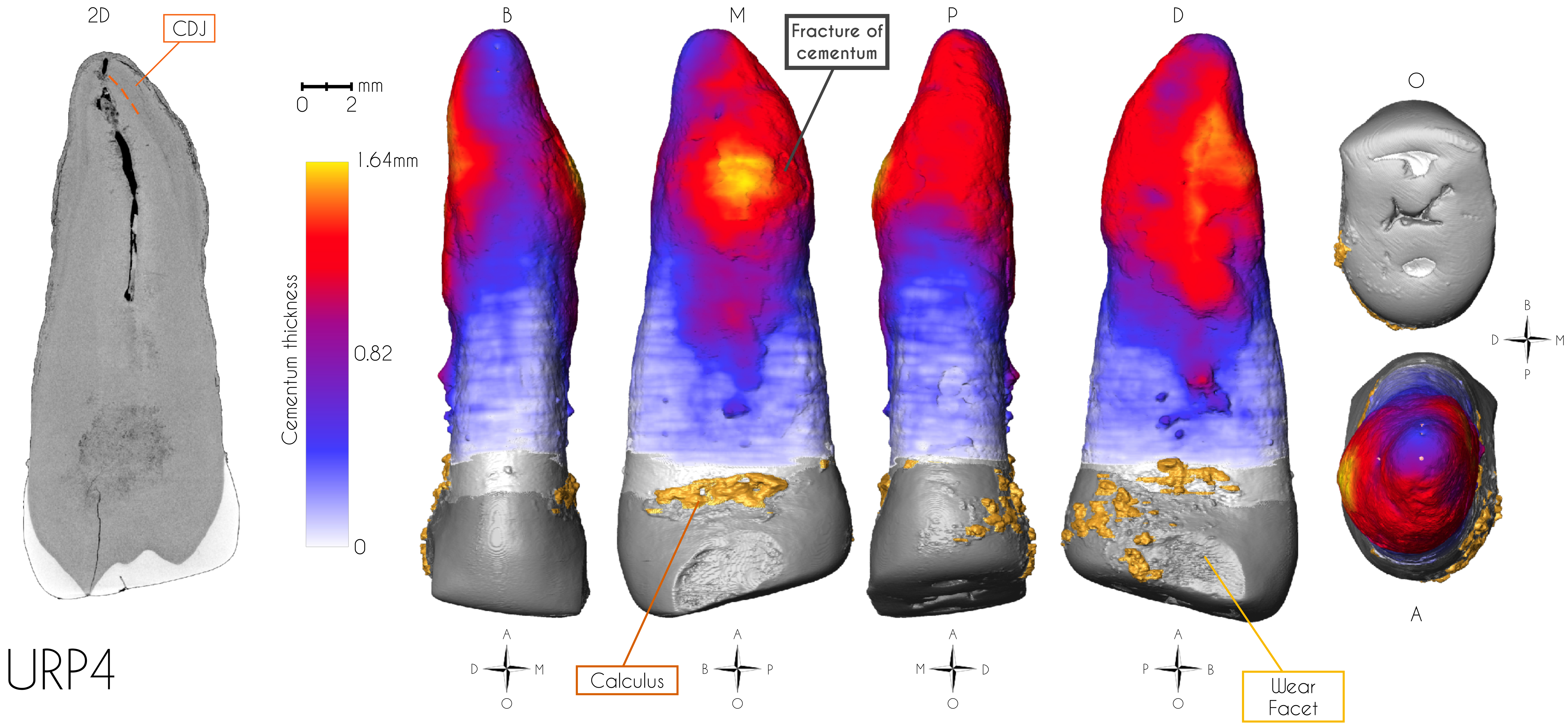

Microtomography - Thickness 3D maps				
MAX THI (µm)	LOC MAX	LOC MIN	PREF LOC	Note
1640	2m	<	No	FR (2m)

MAX THI: maximum thickness of cementum;
LOC MAX: location of maximum thickness;
LOC MIN: location of minimum thickness;
PREF LOC: preferential location;
FR: fracture of cementum
(1: apical root third; 2: middle root third; 3: cervical root third; m: mesial; d: distal; <: buccal; >: lingual)

<u>Hypofunctional</u>

URP4

Face	Range	Light	Width	Height	Columns/Rows	Factor	Led Color	Gain	Gamma	Overlapping
M	781	5.50%	2.46mm	1.85mm	3/3	2	Blue	1.47	1.61	10 %

Sp17_15

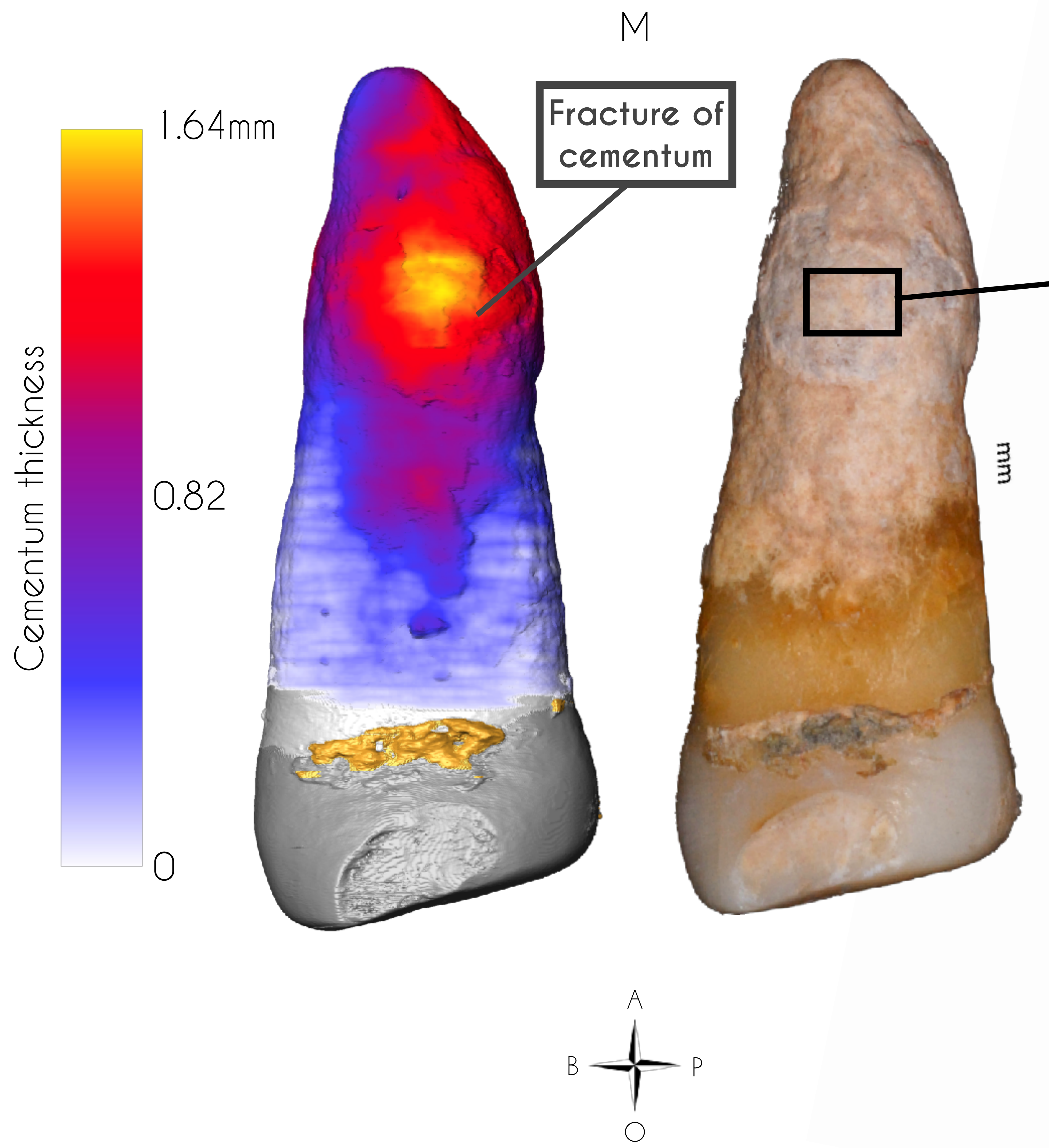

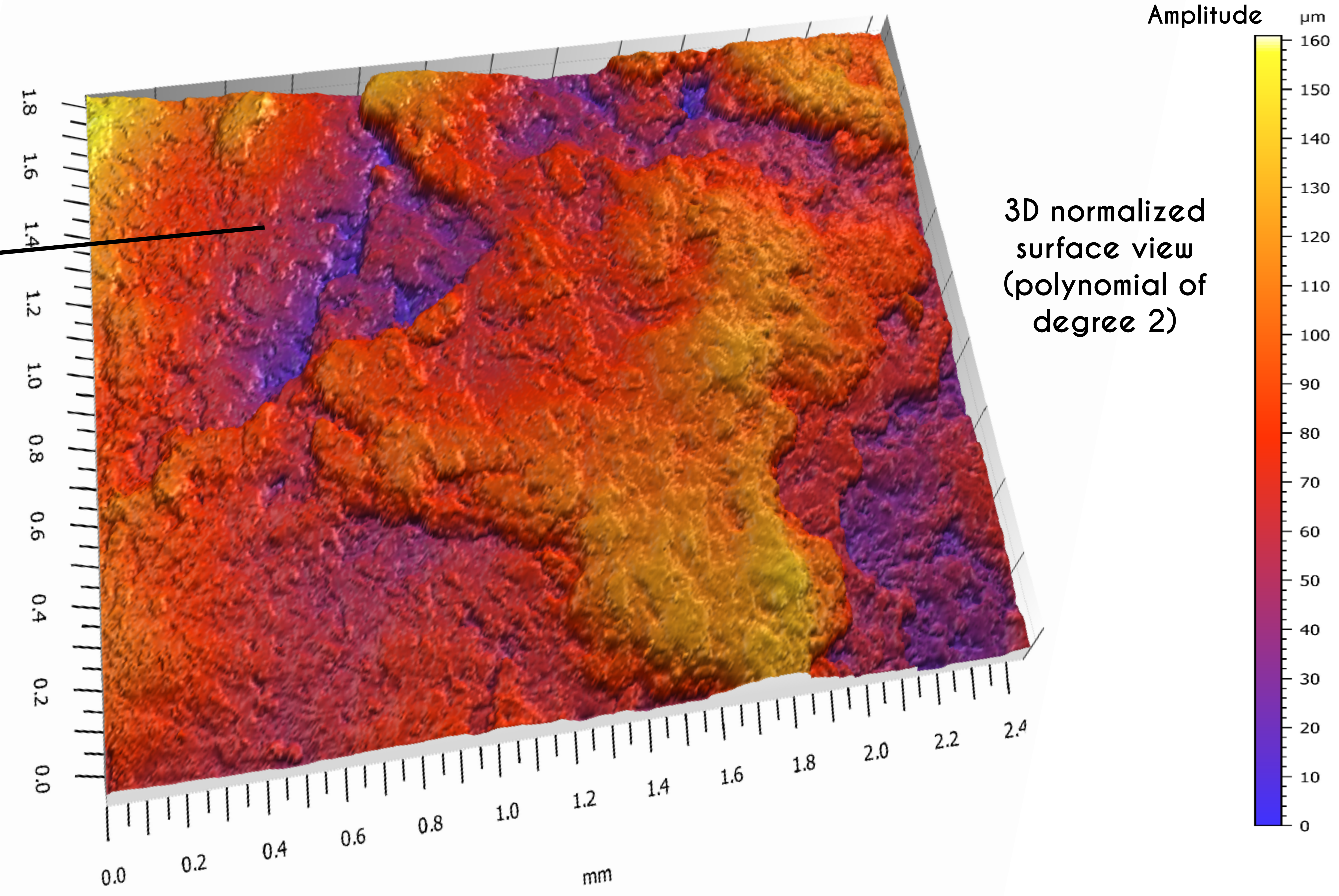

Confocal microscopy - Topography 3D maps			
LOC MAX	MAX VE (µm)	ST	Note
2m	160	-R2	FR

Atypical ST: fracture of cementum

LOC MAX: location of maximum thickness (1: apical root third; 2: middle root third; 3: cervical root third; m: mesial; d: distal; <: buccal; >: lingual);
MAX VE: maximum vertical elevation;
ST: surface texture; : scored **(+)** if it is high, **(-)** if it is low; **S:** smooth or **R:** rough; the frequency of occurrence of the elevations scored **(1)** if the frequency is high, **(2)** if the frequency is low.
FR: fracture of cementum

Hypofunctional

Sp20_35

Tooth	Occlusal Wear			CAR Si/Sta	Pulp EXP	IMP	Hypercementosis	Bone Context			
	DEG	DIR	FOR					FEN	CAL	NT	ANTA
**LLP4**	2	6	2	-	0	0	1.2.m	0	0	0	1

LLP4

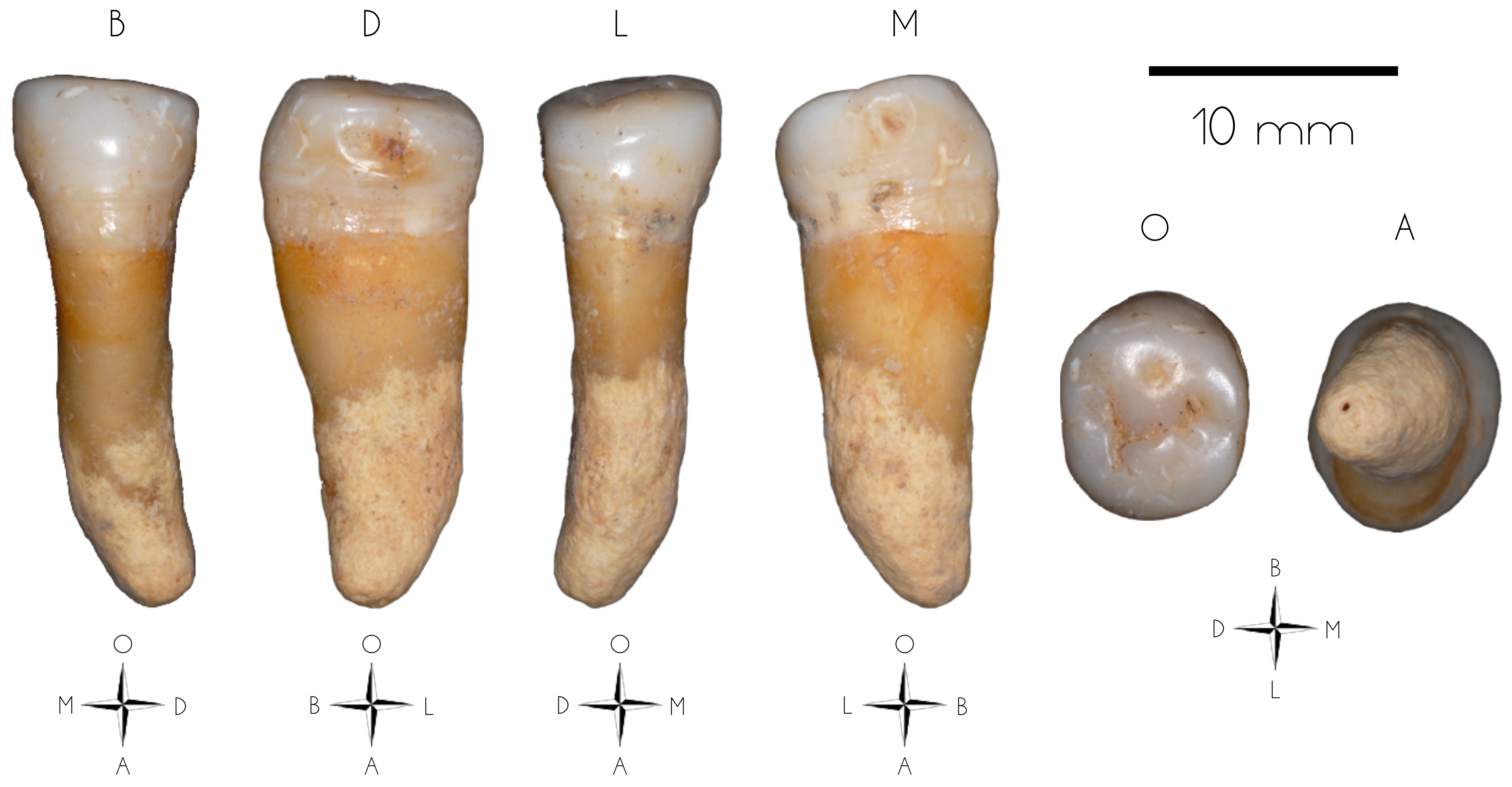

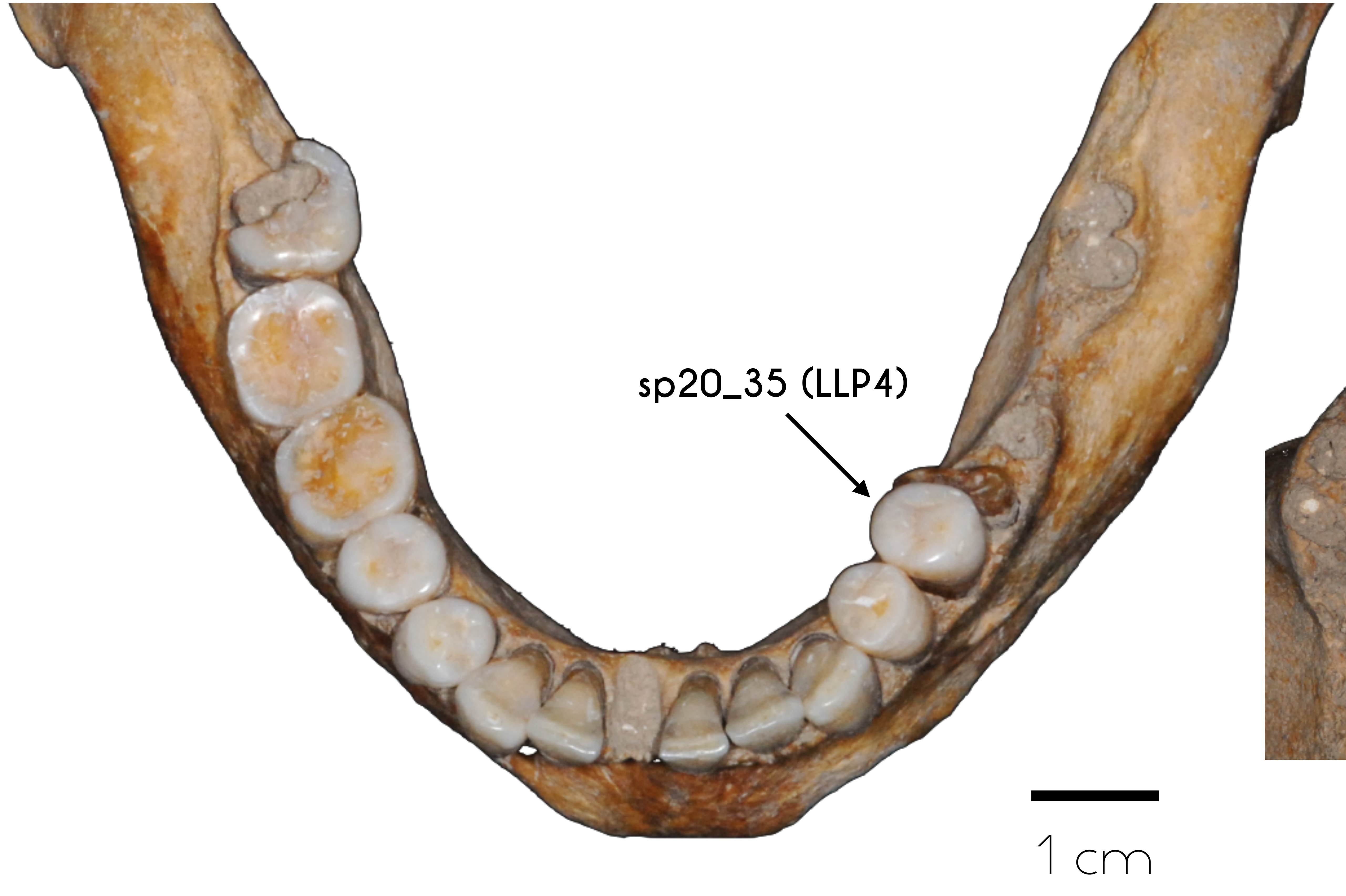

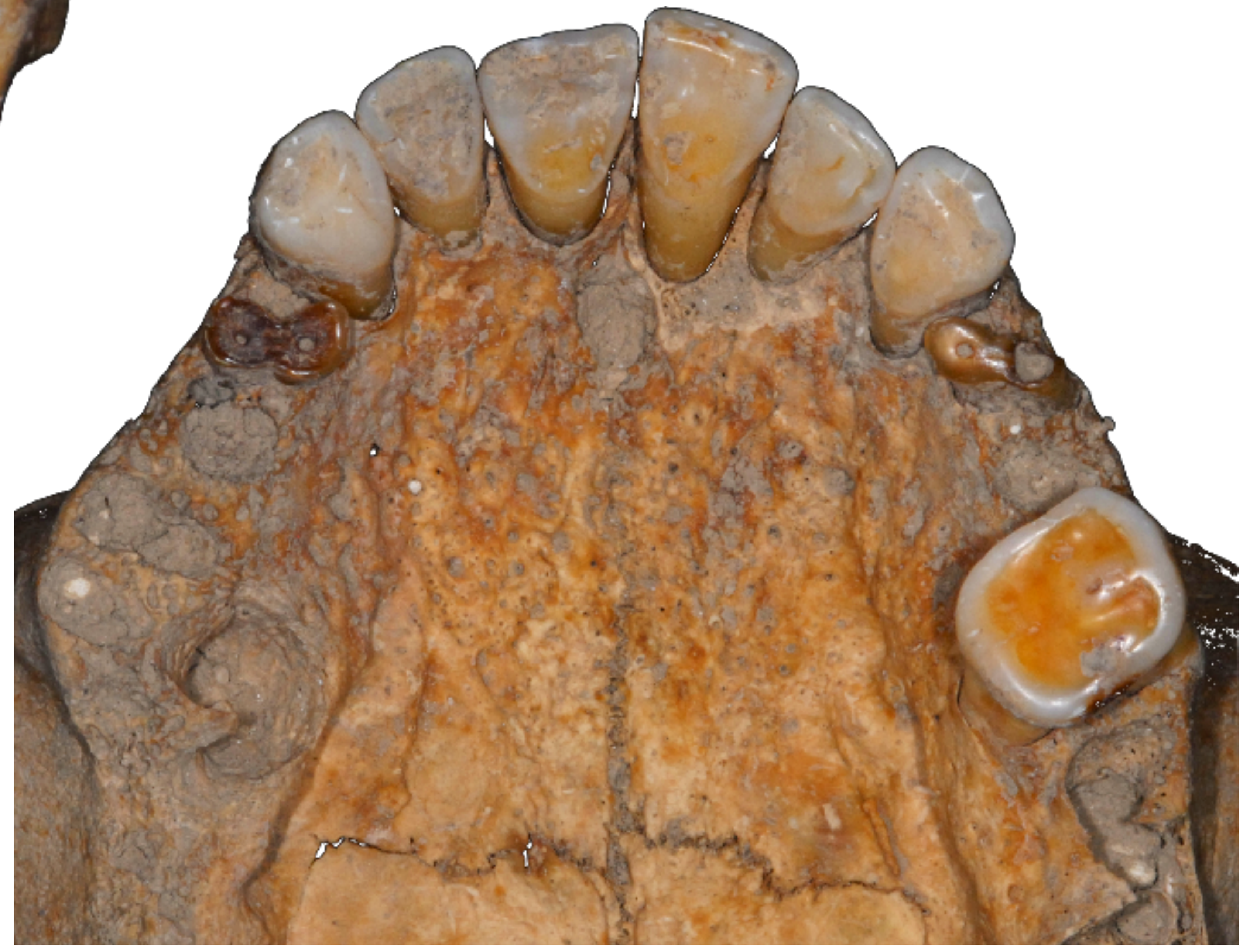

DEG: degree; **DIR:** direction; **FOR:** form - **CAR**: carious lesion - **Pulp EXP:** pulp exposure - **IMP**: impaction - **FEN:** fenestration; **CAL:** calculus; **NT:** tooth loss in neighbouring teeth; **ANTA:** antagonist

Sp20_35

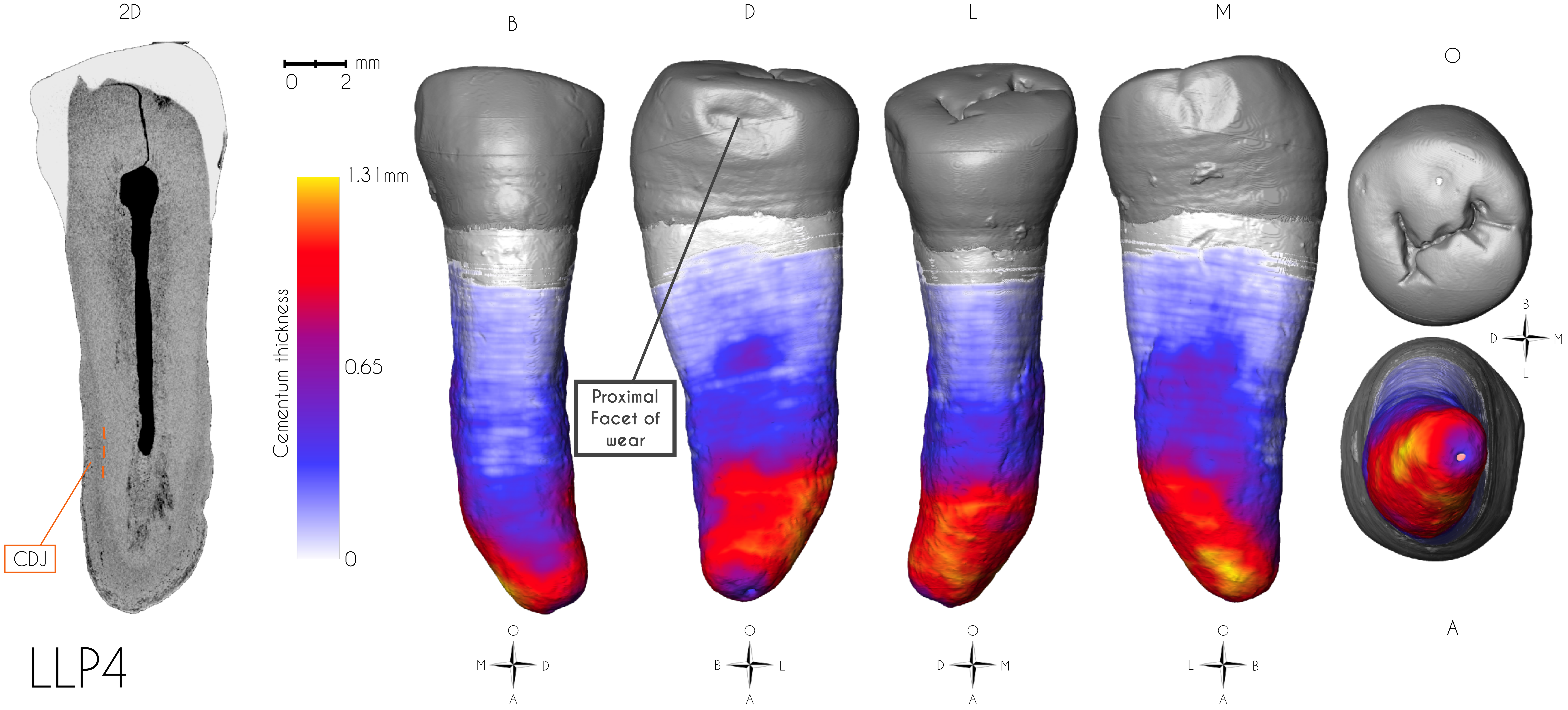

Microtomography - Thickness 3D maps				
MAX THI (μm)	LOC MAX	LOC MIN	PREF LOC	Note
**1310**	**1m**	**<**	**No**	**-**

MAX THI: maximum thickness of cementum;
LOC MAX: location of maximum thickness;
LOC MIN: location of minimum thickness;
(1: apical root third; 2: middle root third; 3: cervical root third; m: mesial; d: distal; <: buccal; >: lingual)
PREF LOC: preferential location;

Hypofunctional

LLP4

Face	Range	Light	Width	Height	Columns/Rows	Factor	Led Color	Gain	Gamma	Overlapping
M	1582	6.28%	2.46mm	1.85mm	3/3	2	Blue	1.47	1.61	10 %

Sp20_35

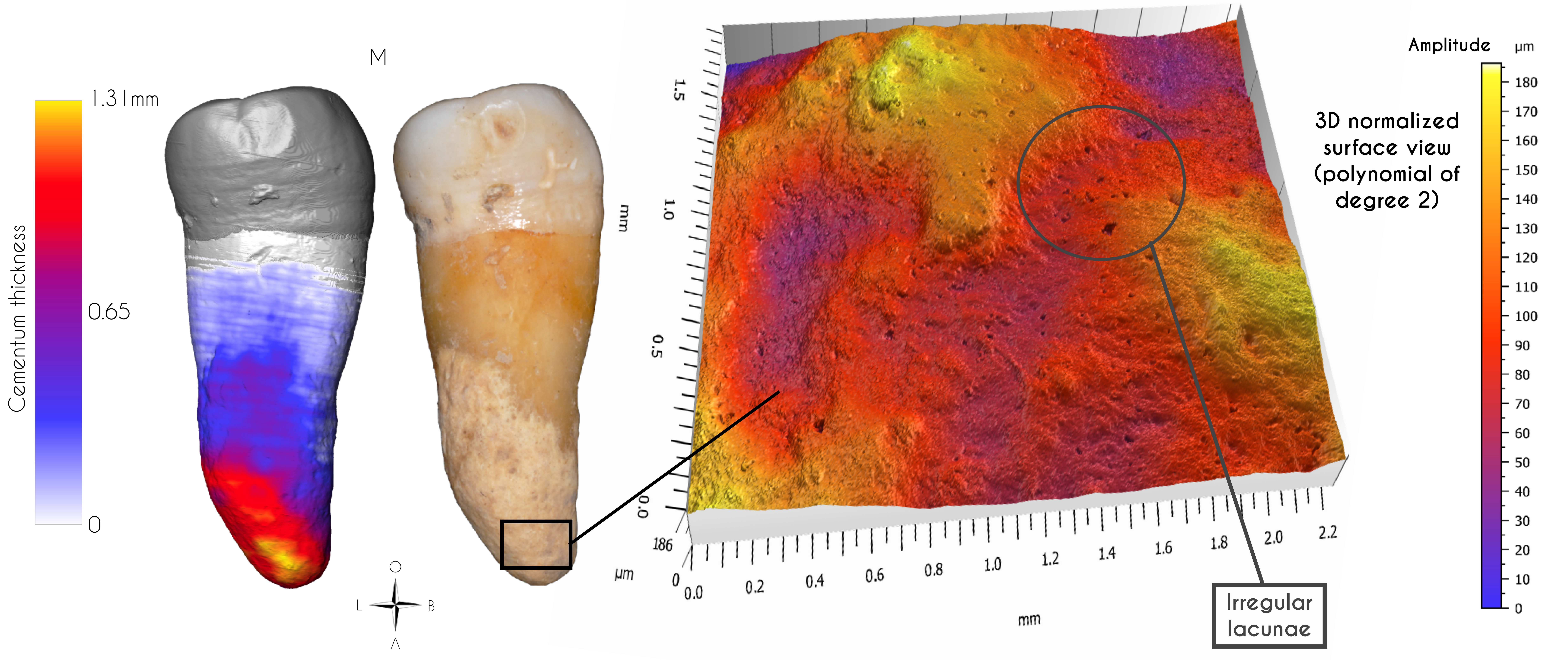

Confocal microscopy - Topography 3D maps			
LOC MAX	MAX VE (µm)	ST	Note
1m	180	-S2	IL

LOC MAX: location of maximum thickness (1: apical root third; 2: middle root third; 3: cervical root third; m: mesial; d: distal; <: buccal; >: lingual);
MAX VE: maximum vertical elevation;
ST: surface texture; : scored (**+**) if it is high, (**-**) if it is low; **S:** smooth or **R:** rough; the frequency of occurrence of the elevations scored (**1**) if the frequency is high, (**2**) if the frequency is low.
IL: irregular lacunae

Hypofunctional

LLP4

Face	Range	Light	Width	Height	Columns/Rows	Factor	Led Color	Gain	Gamma	Overlapping
D	688	4.97%	2.46mm	1.25mm	3/2	2	Blue	1.49	1.79	10 %

Sp20_35

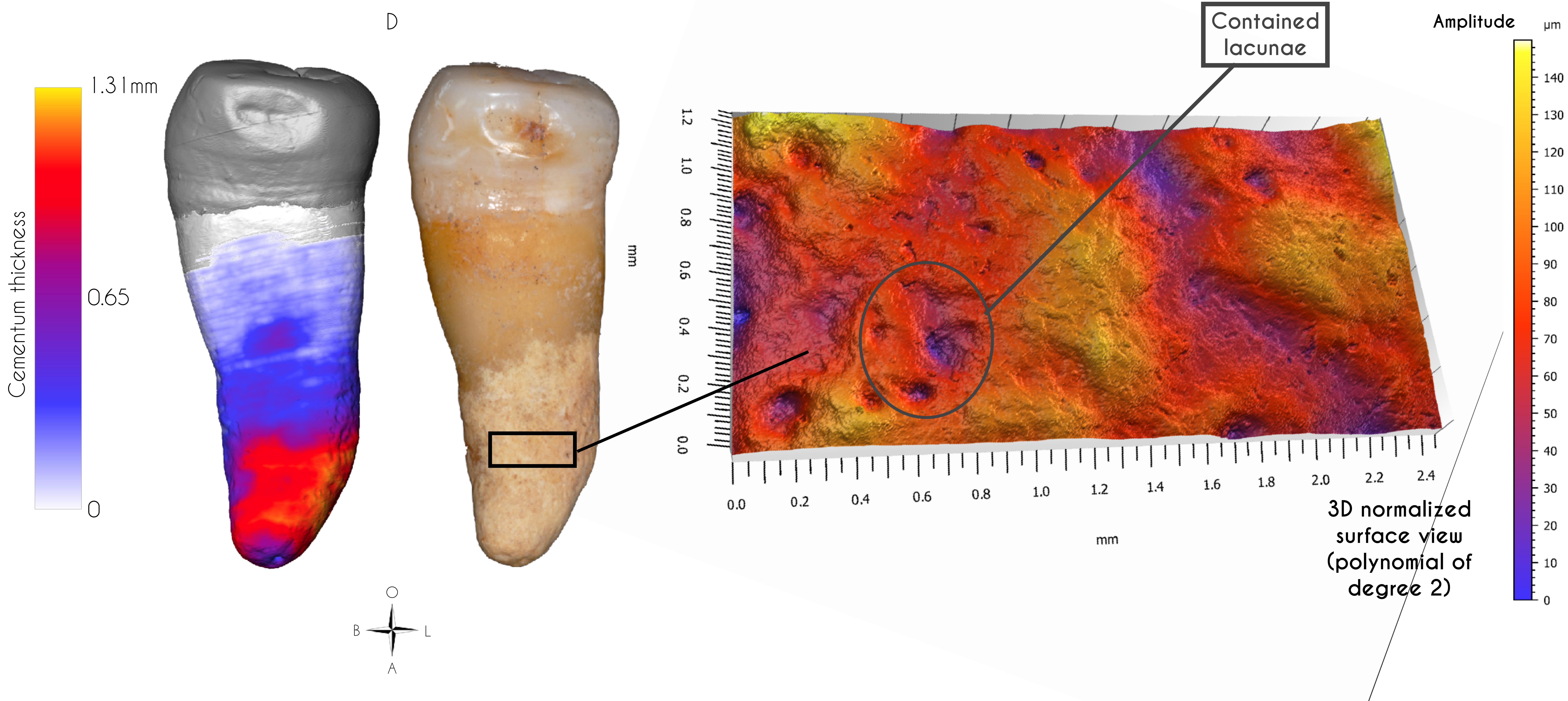

Confocal microscopy - Topography 3D maps			
OF	MAX VE (µm)	ST	Note
2d	150	+S2	CL

OF: other face;
MAX VE: maximum vertical elevation;
ST: surface texture; : scored **(+)** if it is high, **(-)** if it is low; **S:** smooth or **R:** rough; the frequency of occurrence of the elevations scored **(1)** if the frequency is high, **(2)** if the frequency is low.
CL: contained lacunae

Hypofunctional

Sp173_45

Tooth	Occlusal Wear			CAR Si/Sta	Pulp EXP	IMP	Hypercementosis	Bone Context			
	DEG	DIR	FOR					FEN	CAL	NT	ANTA
**LRP4**	3	6	3	-	0	0	1.2.m	0	0	0	2

LRP4

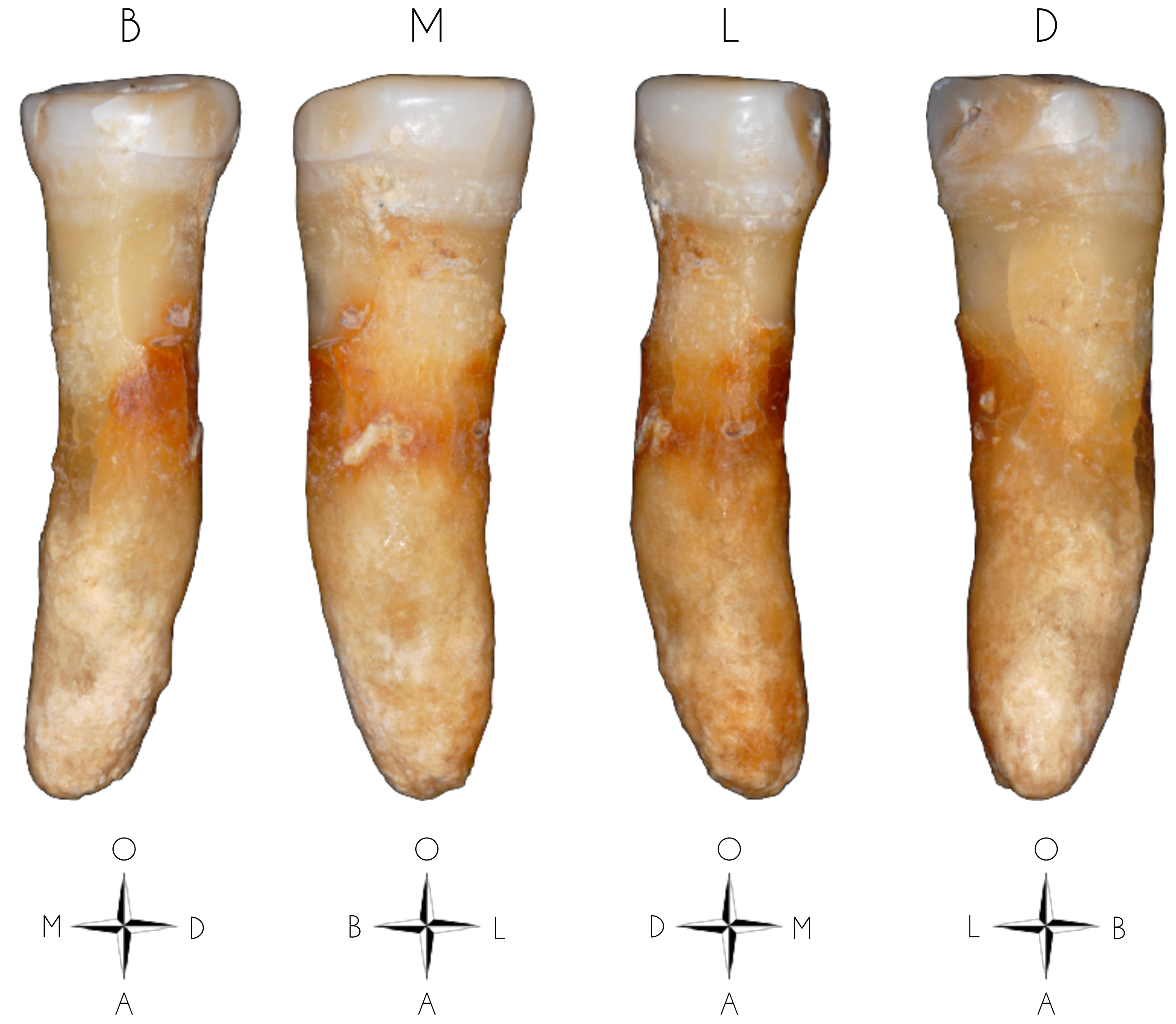

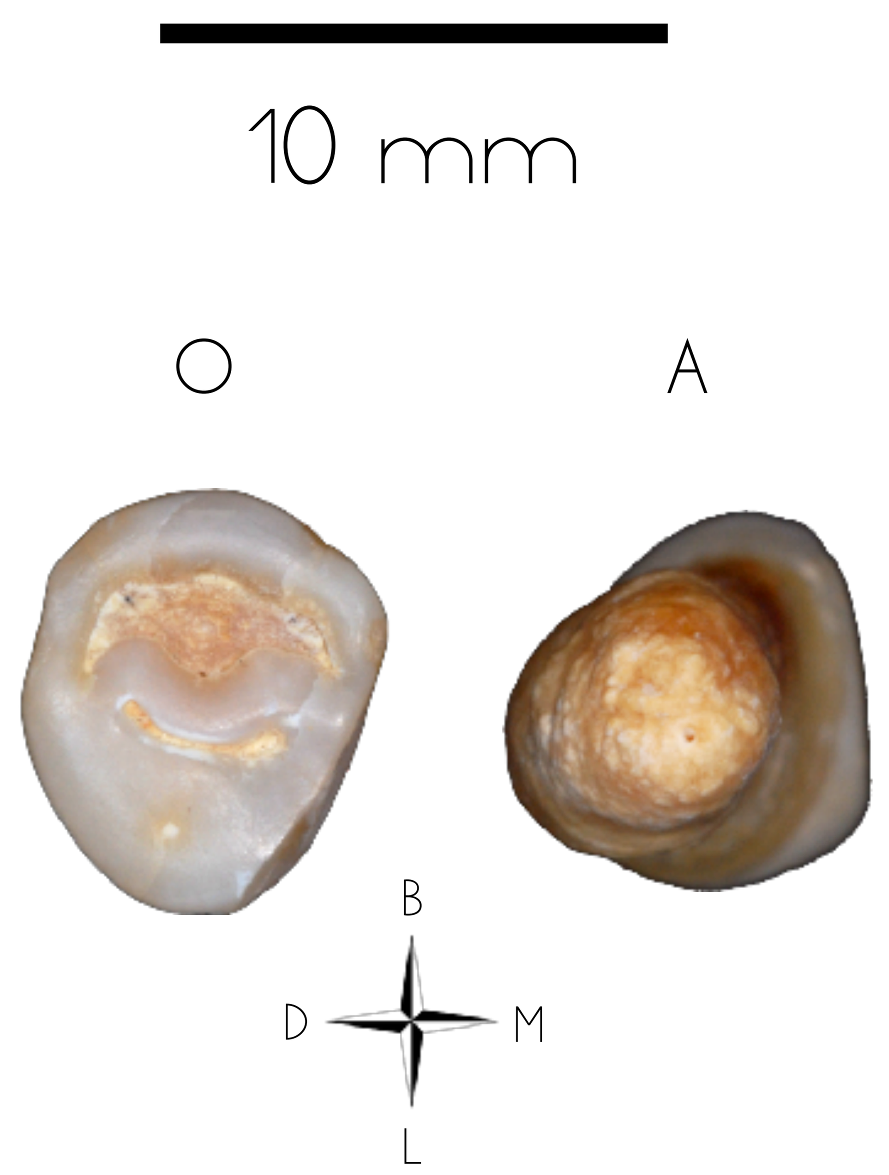

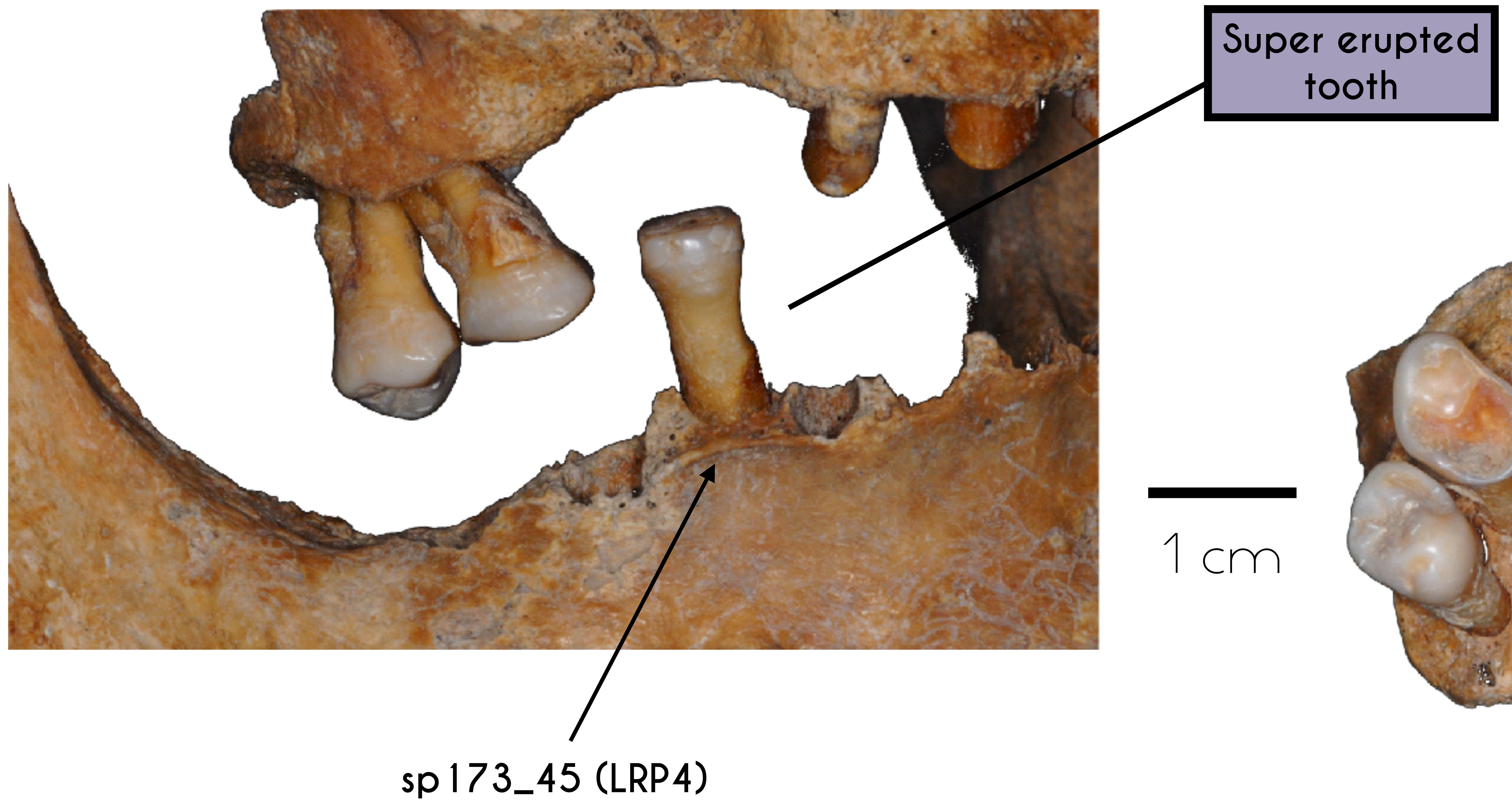

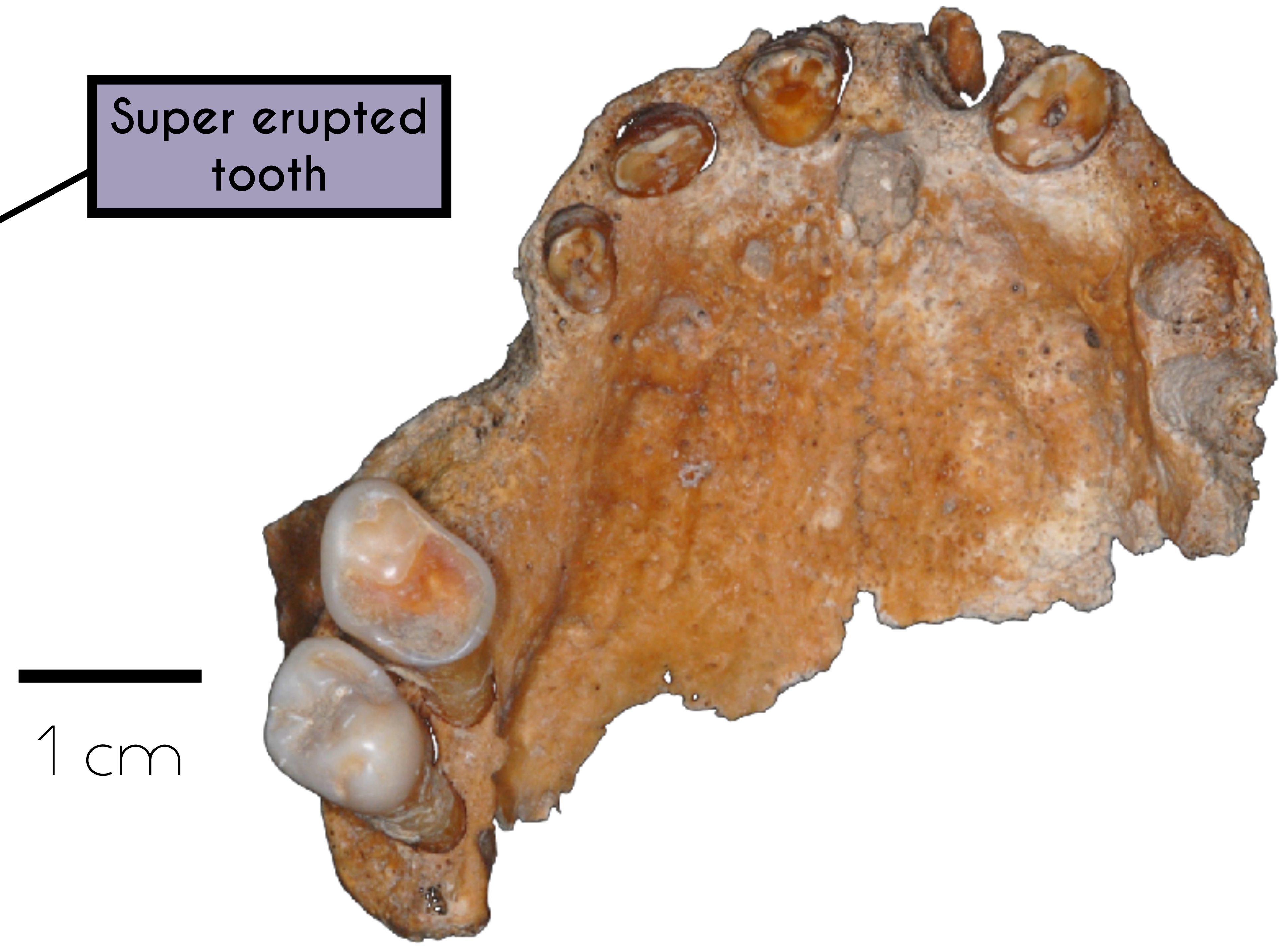

DEG: degree; **DIR:** direction; **FOR:** form - **CAR**: carious lesion - **Pulp EXP:** pulp exposure - **IMP**: impaction - **FEN:** fenestration; **CAL:** calculus; **NT:** tooth loss in neighbouring teeth; **ANTA:** antagonist

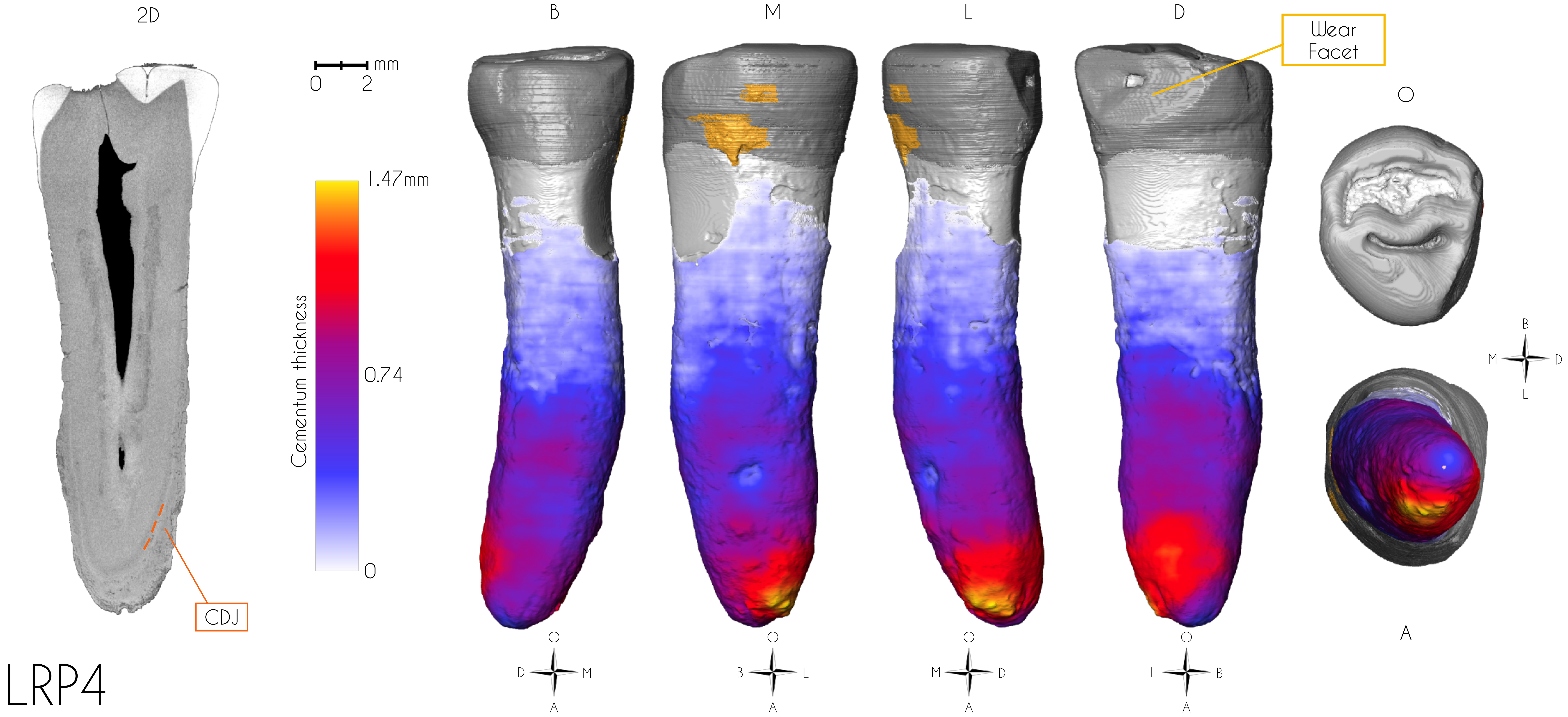

Sp173_45

Microtomography - Thickness 3D maps				
MAX THI (µm)	LOC MAX	LOC MIN	PREF LOC	Note
1470	1 >	<	No	SE

MAX THI: maximum thickness of cementum;
LOC MAX: location of maximum thickness;
LOC MIN: location of minimum thickness;
(1: apical root third; 2: middle root third; 3: cervical root third; m: mesial; d: distal; <: buccal; >: lingual)
PREF LOC: preferential location;
SE: super-erupted tooth

Hypofunctional

LRP4

Face	Range	Light	Width	Height	Columns/Rows	Factor	Led Color	Gain	Gamma	Overlapping
LM	1251	6.54%	0.88mm	4.82mm	1/8	2	Blue	1.5	1.8	10 %

Sp173_45

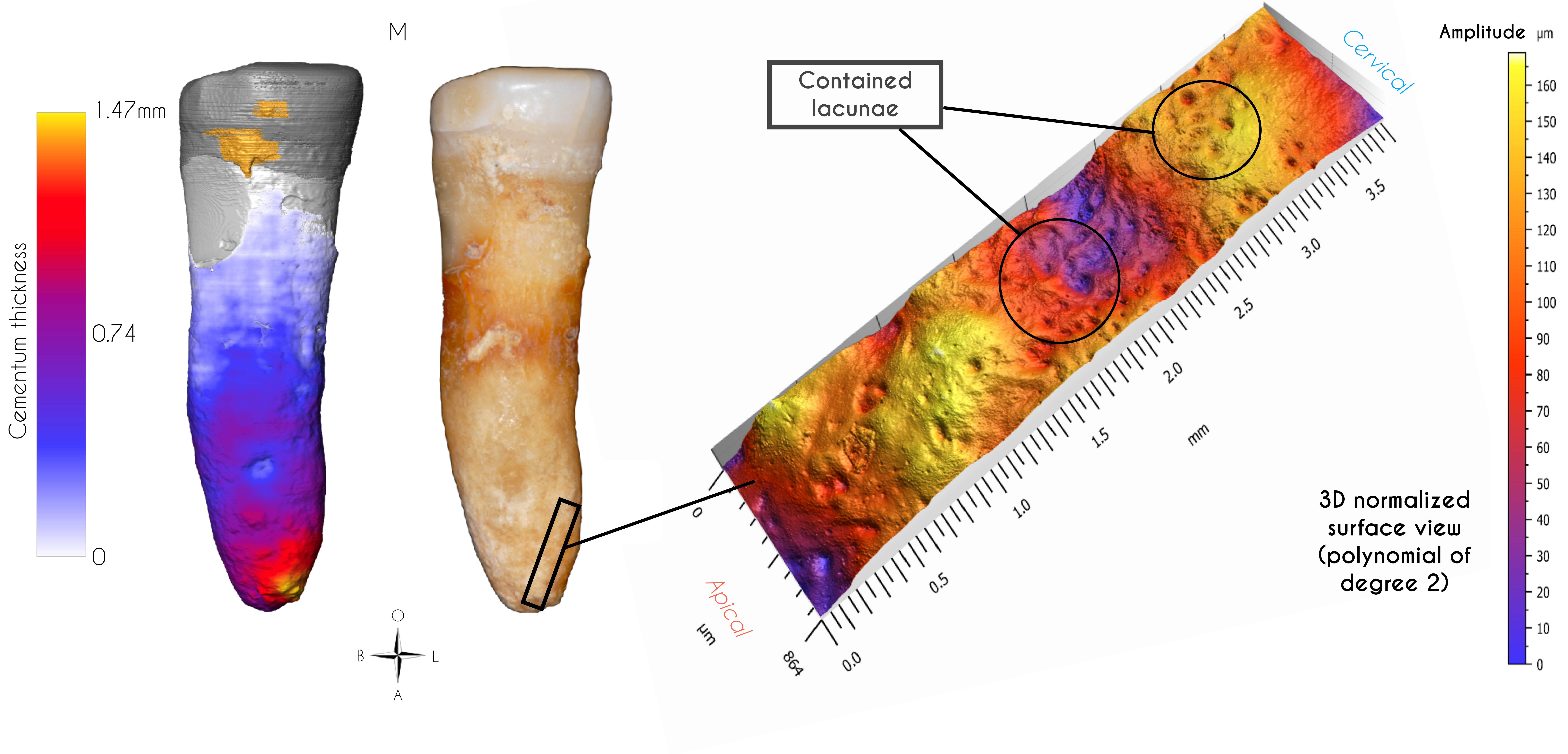

Confocal microscopy - Topography 3D maps			
LOC MAX	MAX VE (µm)	ST	Note
1>	170	+S1	CL

Sharpeys Fibers (super erupted tooth)

LOC MAX: location of maximum thickness (1: apical root third; 2: middle root third; 3: cervical root third; m: mesial; d: distal; <: buccal; >: lingual);
MAX VE: maximum vertical elevation;
ST: surface texture; : scored **(+)** if it is high, **(-)** if it is low; **S:** smooth or **R:** rough; the frequency of occurrence of the elevations scored (**1**) if the frequency is high, (**2**) if the frequency is low.
CL: contained lacunae

Hypofunctional

Sp914_34

Tooth	Occlusal Wear			CAR Si/Sta	Pulp EXP	IMP	Hypercementosis	Bone Context			
	DEG	DIR	FOR					FEN	CAL	NT	ANTA
**LLP3**	**2**	**1**	**1**	**-**	**0**	**0**	**1.2.m**	**0**	**0**	**0**	**NA**

LLP3

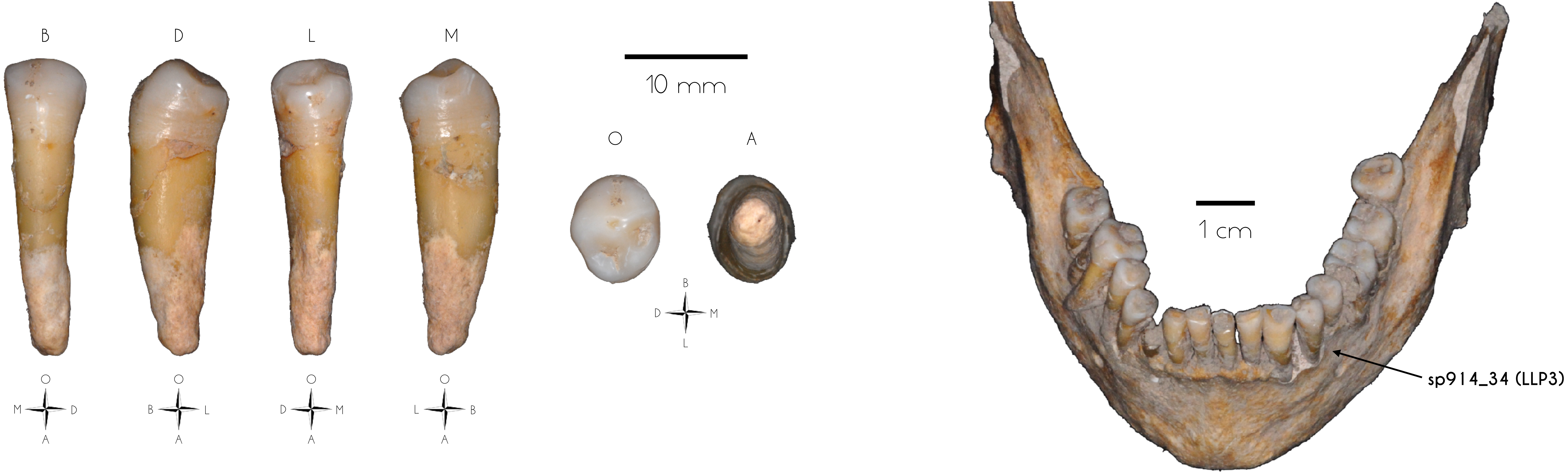

DEG: degree; **DIR:** direction; **FOR:** form - **CAR**: carious lesion - **Pulp EXP:** pulp exposure - **IMP**: impaction - **FEN:** fenestration; **CAL:** calculus; **NT:** tooth loss in neighbouring teeth; **ANTA:** antagonist

Sp914_34

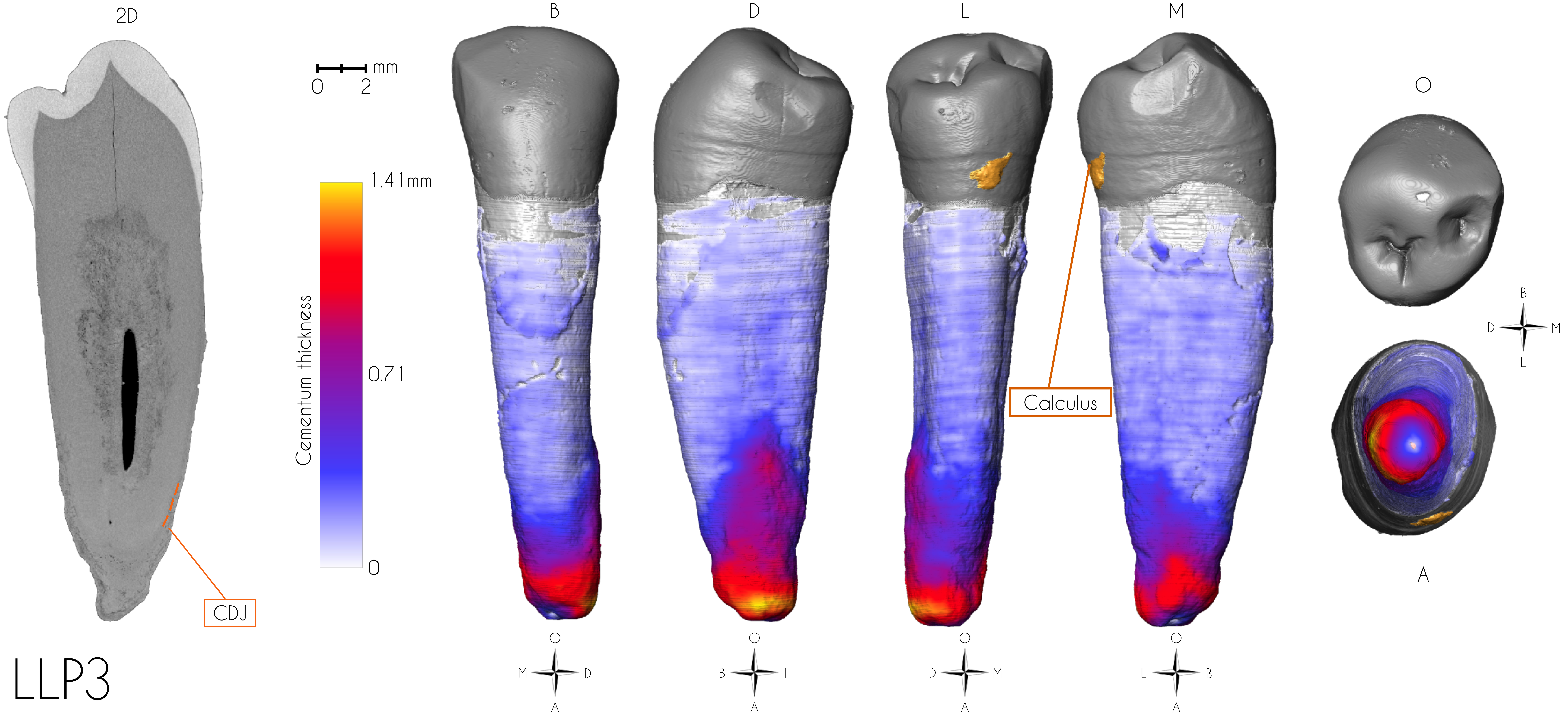

Microtomography - Thickness 3D maps				
MAX THI (µm)	LOC MAX	LOC MIN	PREF LOC	Note
1410	1d	No	No	-

MAX THI: maximum thickness of cementum;
LOC MAX: location of maximum thickness;
LOC MIN: location of minimum thickness;
(1: apical root third; 2: middle root third; 3: cervical root third; m: mesial; d: distal; <: buccal; >: lingual)
PREF LOC: preferential location;

<u>Hypofunctional</u>

LLP3

Face	Range	Light	Width	Height	Columns/Rows	Factor	Led Color	Gain	Gamma	Overlapping
D	454	4.71%	0.88mm	4.82mm	1/8	2	Blue	1.5	1.8	10 %

Sp914_34

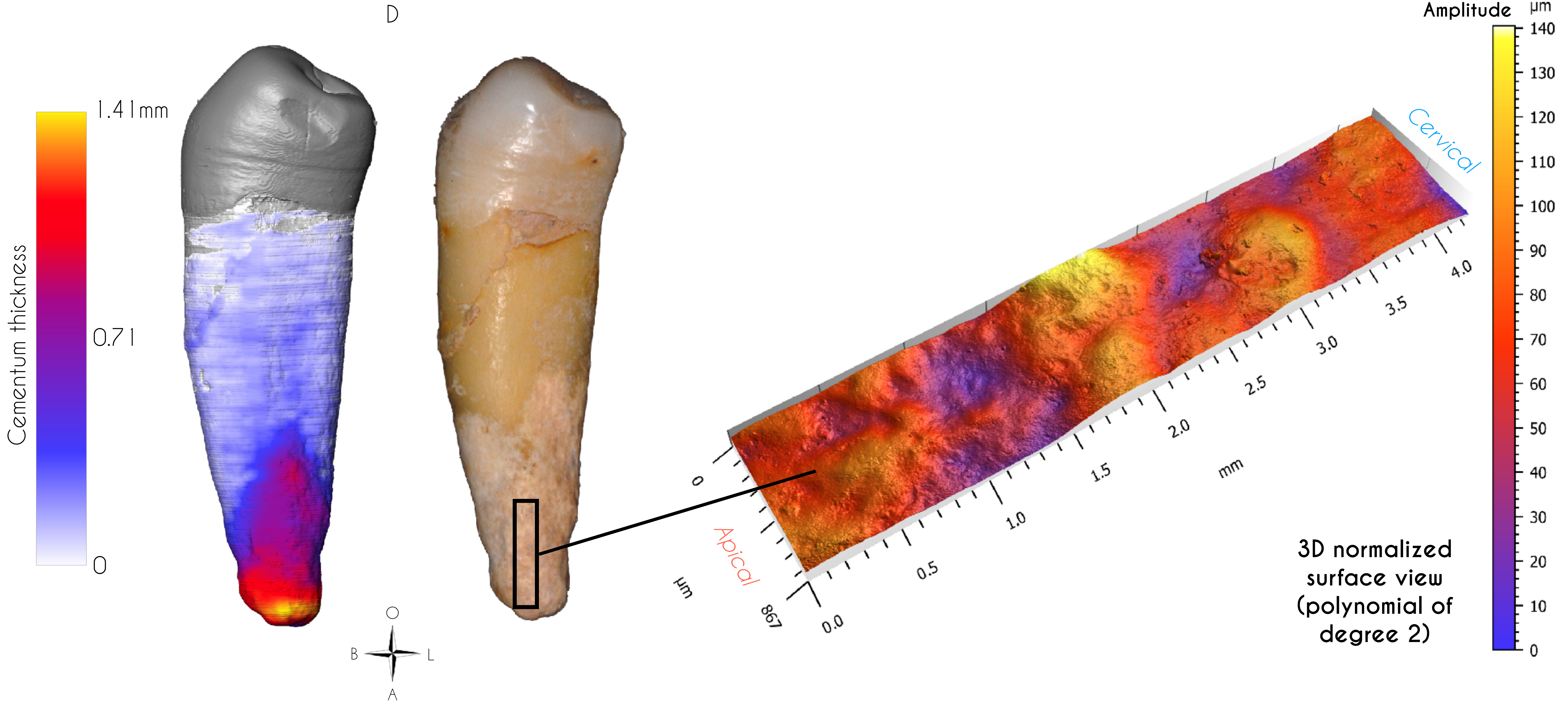

Confocal microscopy - Topography 3D maps			
LOC MAX	MAX VE (µm)	ST	Note
1d	140	-S2	-

LOC MAX: location of maximum thickness (1: apical root third; 2: middle root third; 3: cervical root third; m: mesial; d: distal; <: buccal; >: lingual);
MAX VE: maximum vertical elevation;
ST: surface texture; : scored (**+**) if it is high, (**-**) if it is low; **S:** smooth or **R:** rough; the frequency of occurrence of the elevations scored (**1**) if the frequency is high, (**2**) if the frequency is low.

Hypofunctional

Sp1230_34

Tooth	Occlusal Wear			CAR Si/Sta	Pulp EXP	IMP	Hypercementosis	Bone Context			
	DEG	DIR	FOR					FEN	CAL	NT	ANTA
**LLP3**	3	1	1	-	0	0	1.2.m	0	1	0	1

LLP3

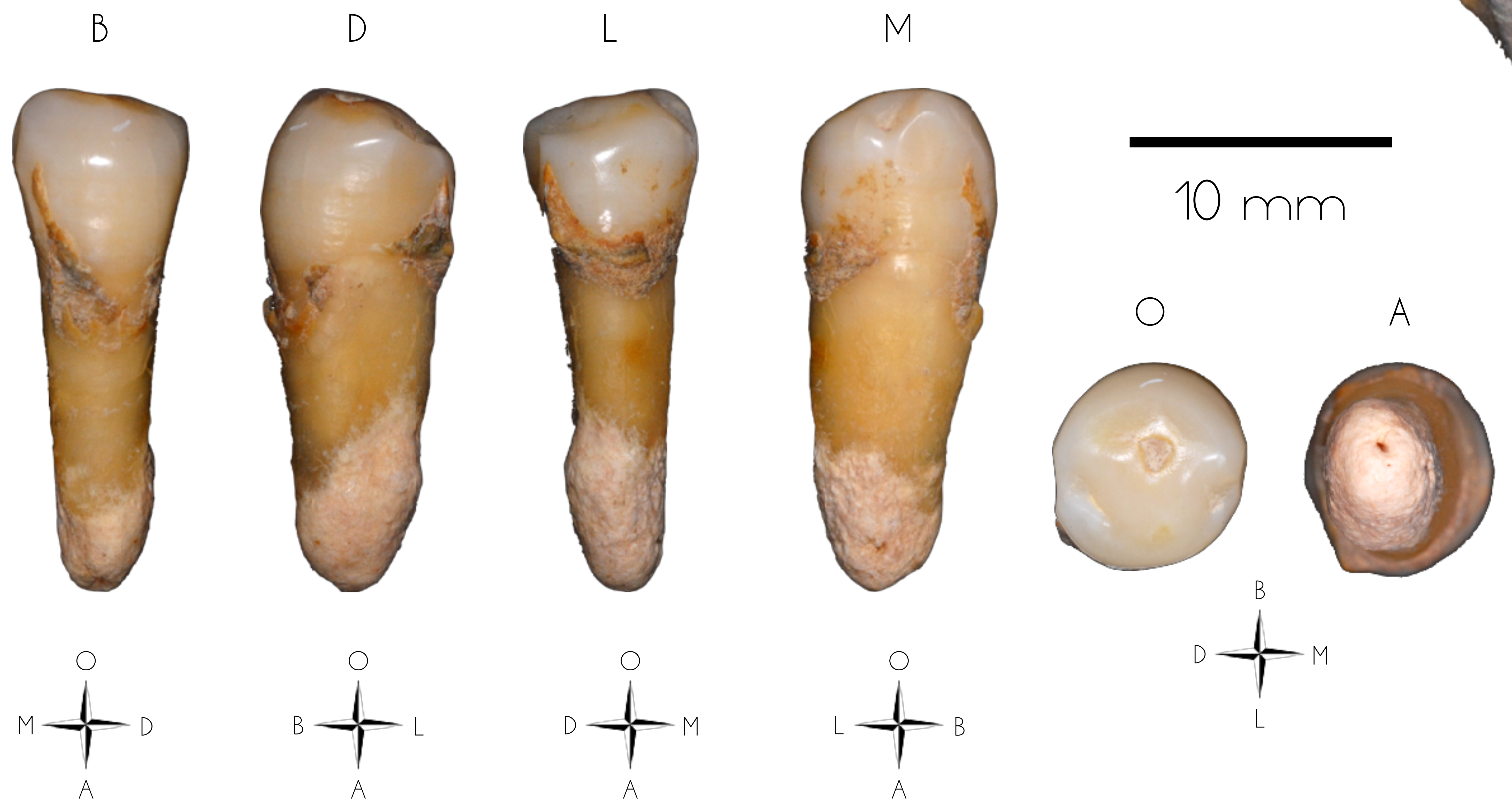

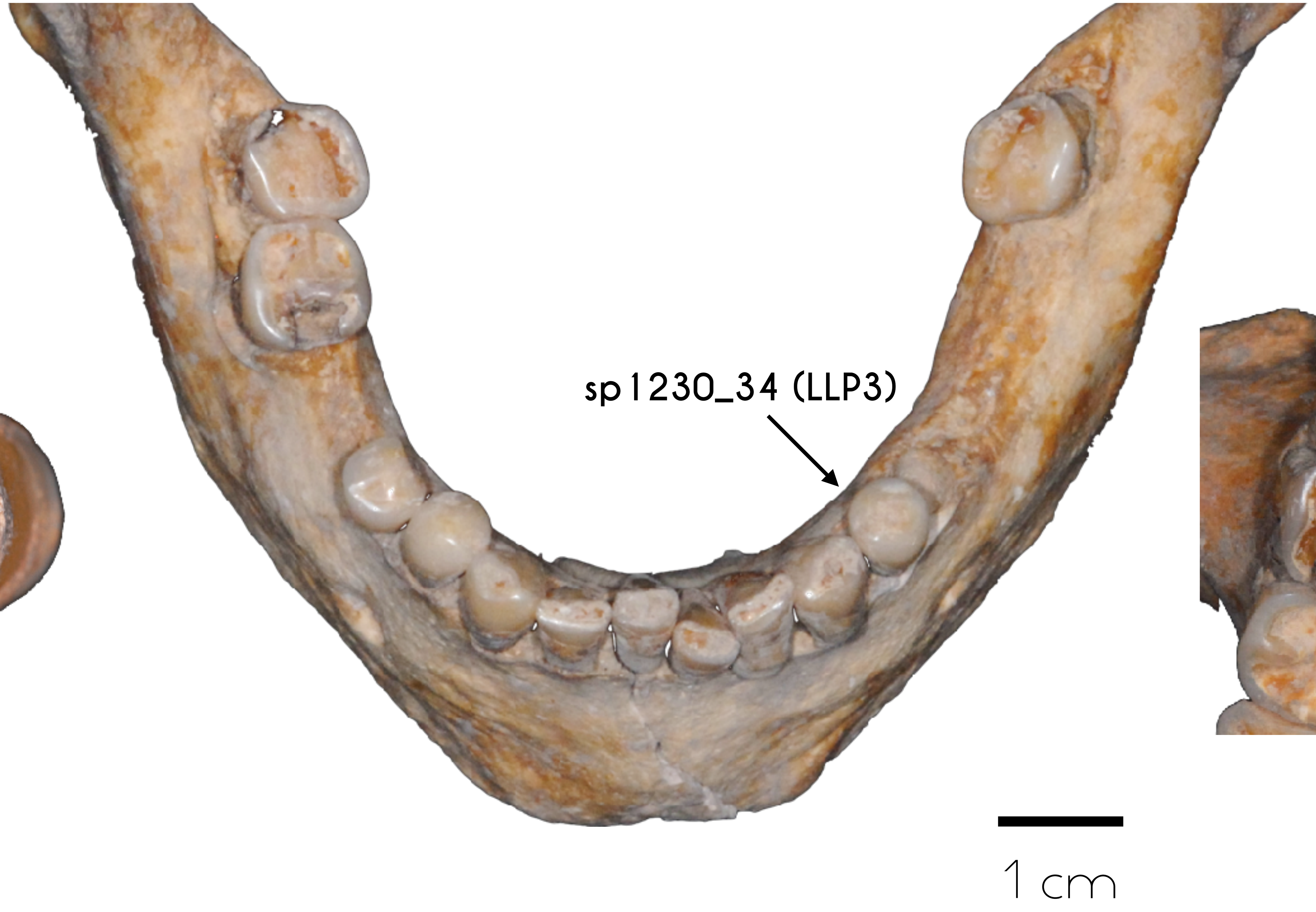

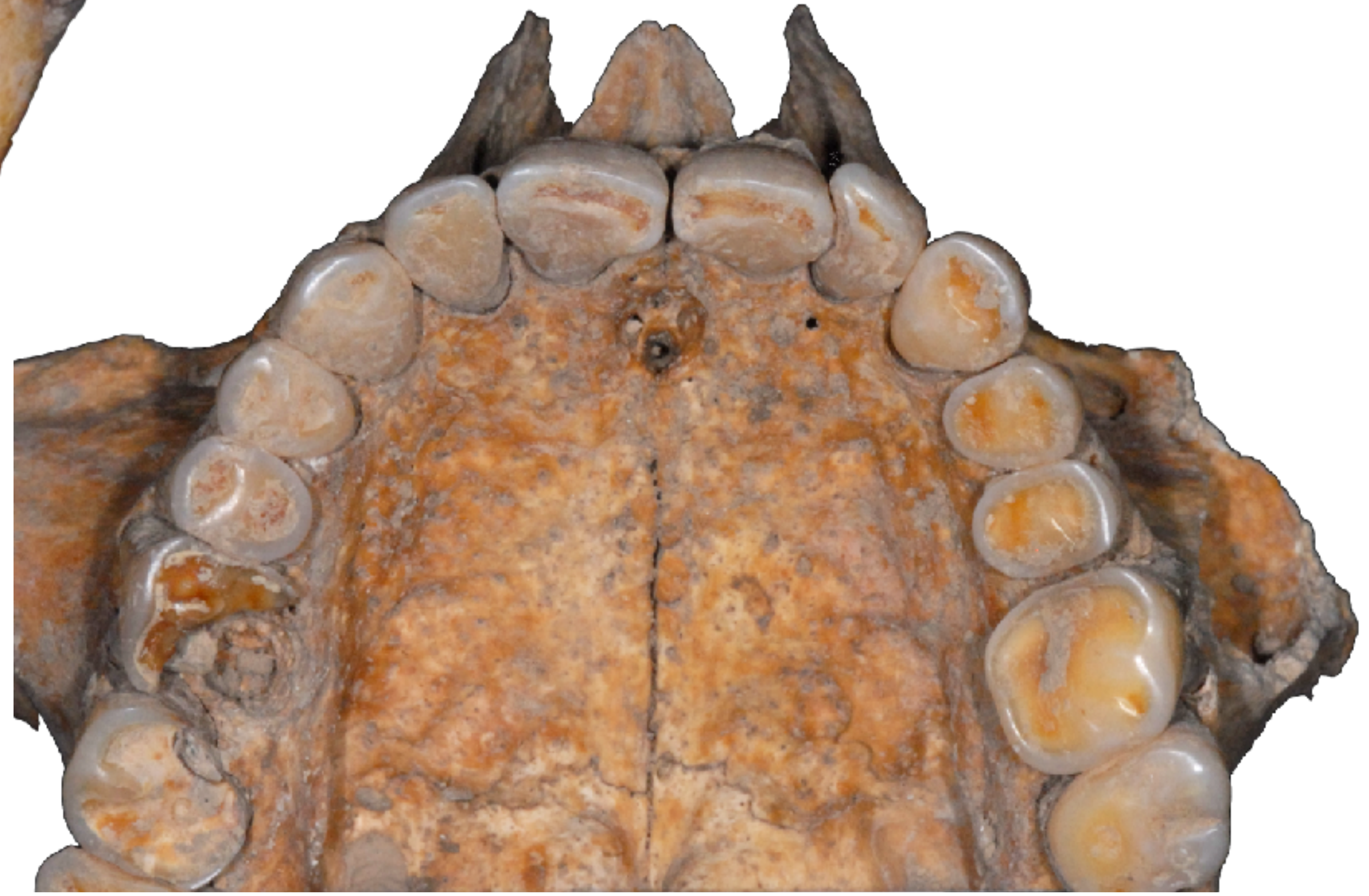

DEG: degree; **DIR:** direction; **FOR:** form - **CAR**: carious lesion - **Pulp EXP:** pulp exposure - **IMP**: impaction - **FEN:** fenestration; **CAL:** calculus; **NT:** tooth loss in neighbouring teeth; **ANTA:** antagonist

Sp1230_34

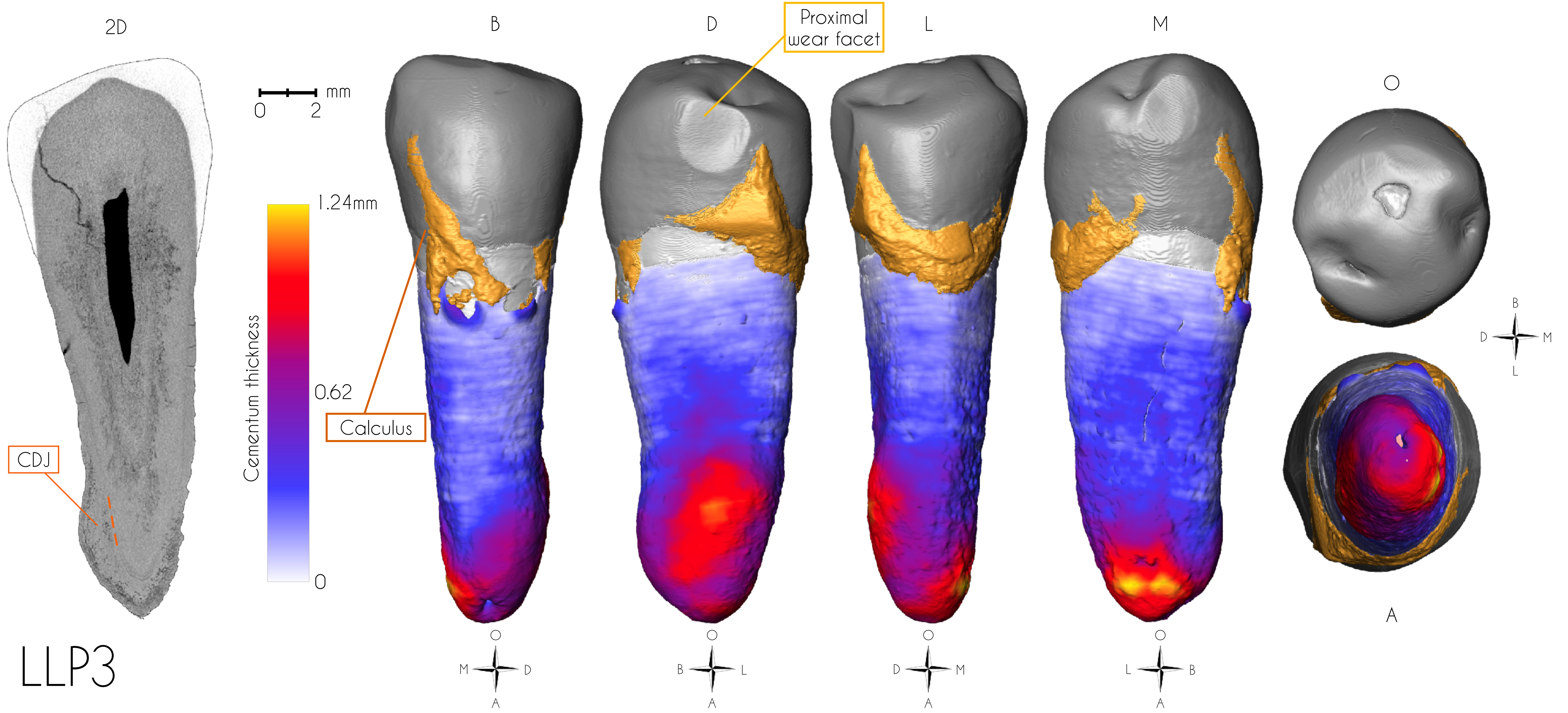

Microtomography - Thickness 3D maps				
MAX THI (µm)	LOC MAX	LOC MIN	PREF LOC	Note
1240	1m	No	No	-

MAX THI: maximum thickness of cementum;
LOC MAX: location of maximum thickness;
LOC MIN: location of minimum thickness;
(1: apical root third; 2: middle root third; 3: cervical root third; m: mesial; d: distal; <: buccal; >: lingual)
PREF LOC: preferential location;

<u>Hypofunctional</u>

LLP3

Face	Range	Light	Width	Height	Columns/Rows	Factor	Led Color	Gain	Gamma	Overlapping
LM	241	4.00%	1.67mm	1.25mm	2/2	2	Blue	1.47	1.61	10 %

Sp1230_34

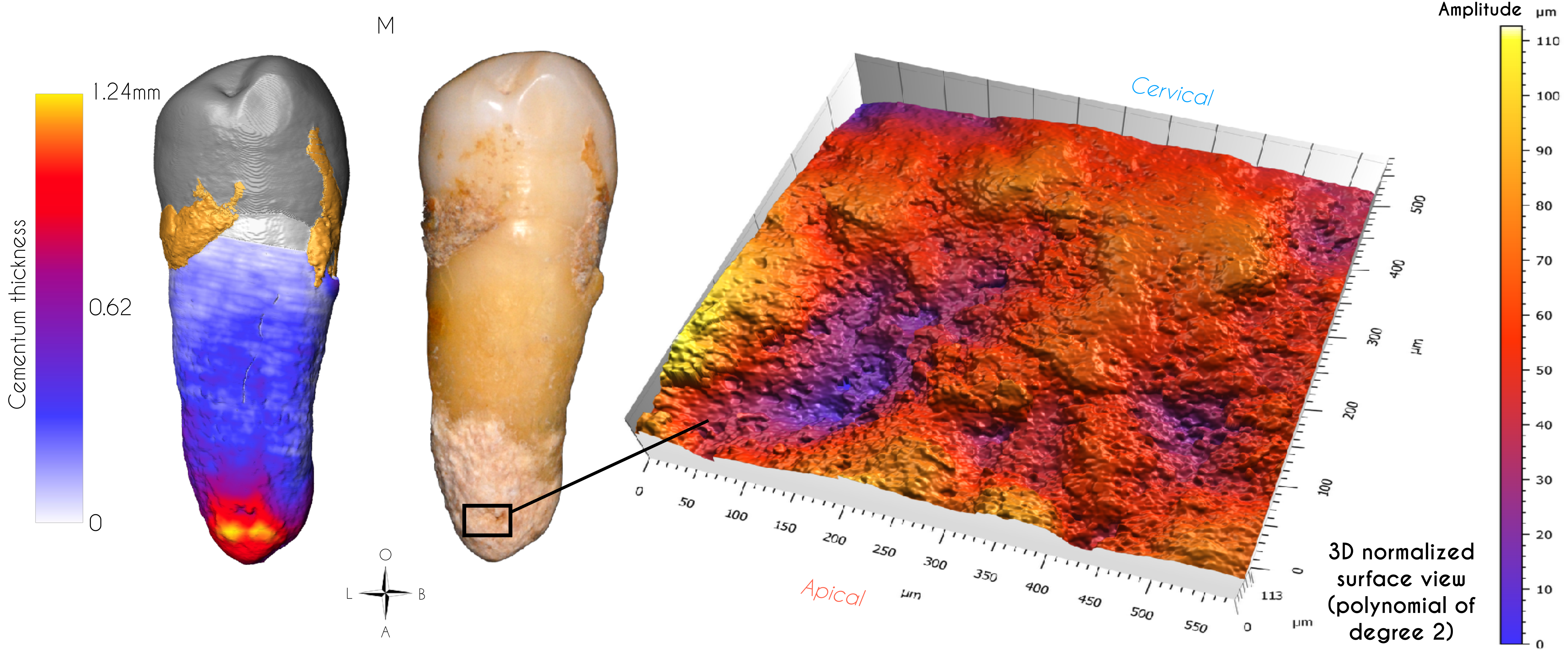

Confocal microscopy - Topography 3D maps			
LOC MAX	MAX VE (μm)	ST	Note
1m	110	-R2	-

LOC MAX: location of maximum thickness (1: apical root third; 2: middle root third; 3: cervical root third; m: mesial; d: distal; <: buccal; >: lingual);
MAX VE: maximum vertical elevation;
ST: surface texture; : scored **(+)** if it is high, **(-)** if it is low; **S:** smooth or **R:** rough; the frequency of occurrence of the elevations scored **(1)** if the frequency is high, **(2)** if the frequency is low.

Hypofunctional

Sp1230_44

Tooth	Occlusal Wear			CAR Si/Sta	Pulp EXP	IMP	Hypercementosis	Bone Context			
	DEG	DIR	FOR					FEN	CAL	NT	ANTA
**LRP3**	**3**	**1**	**1**		**0**	**0**	**1.2.M**	**0**	**0**	**0**	**1**

LRP3

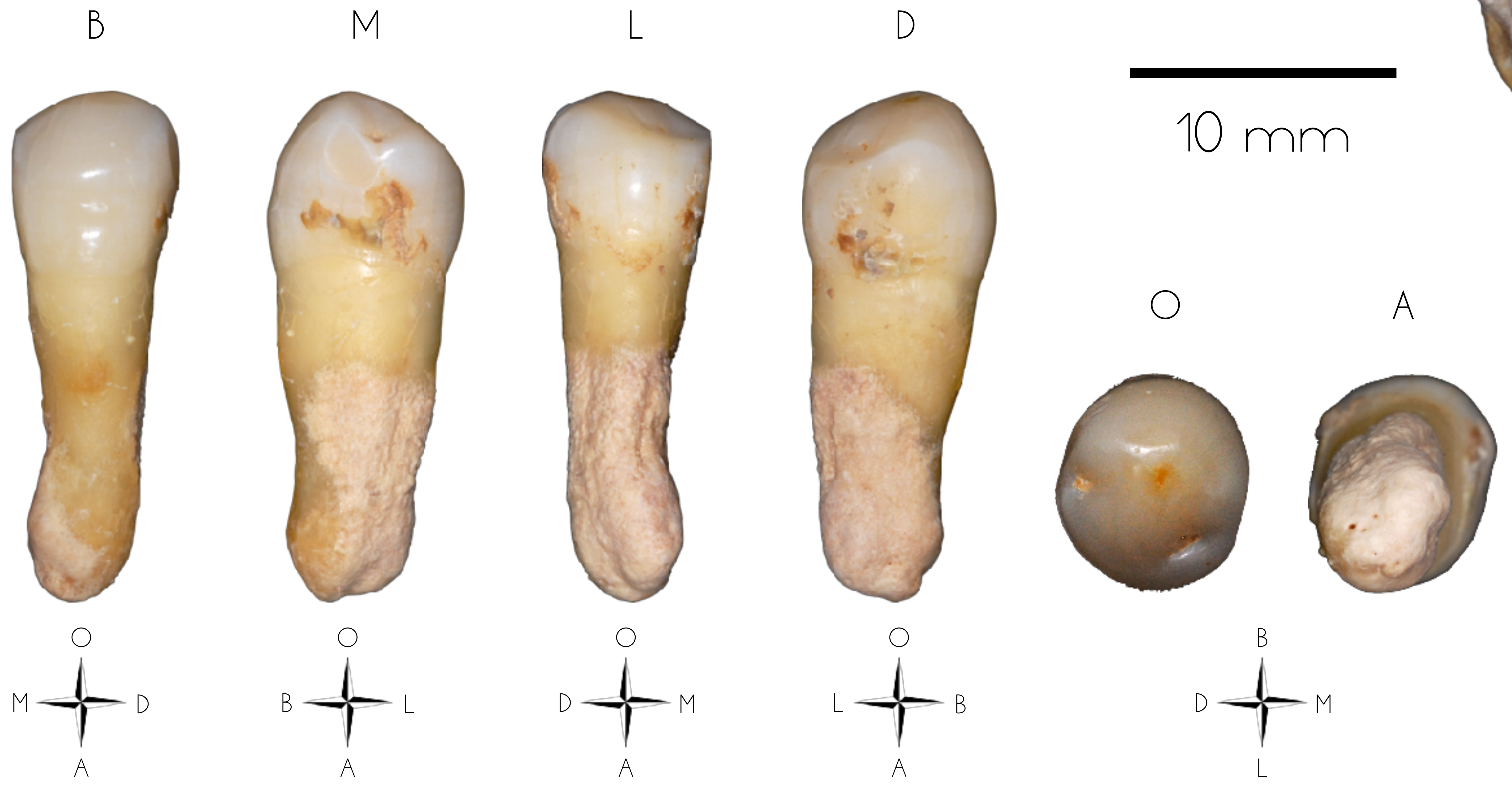

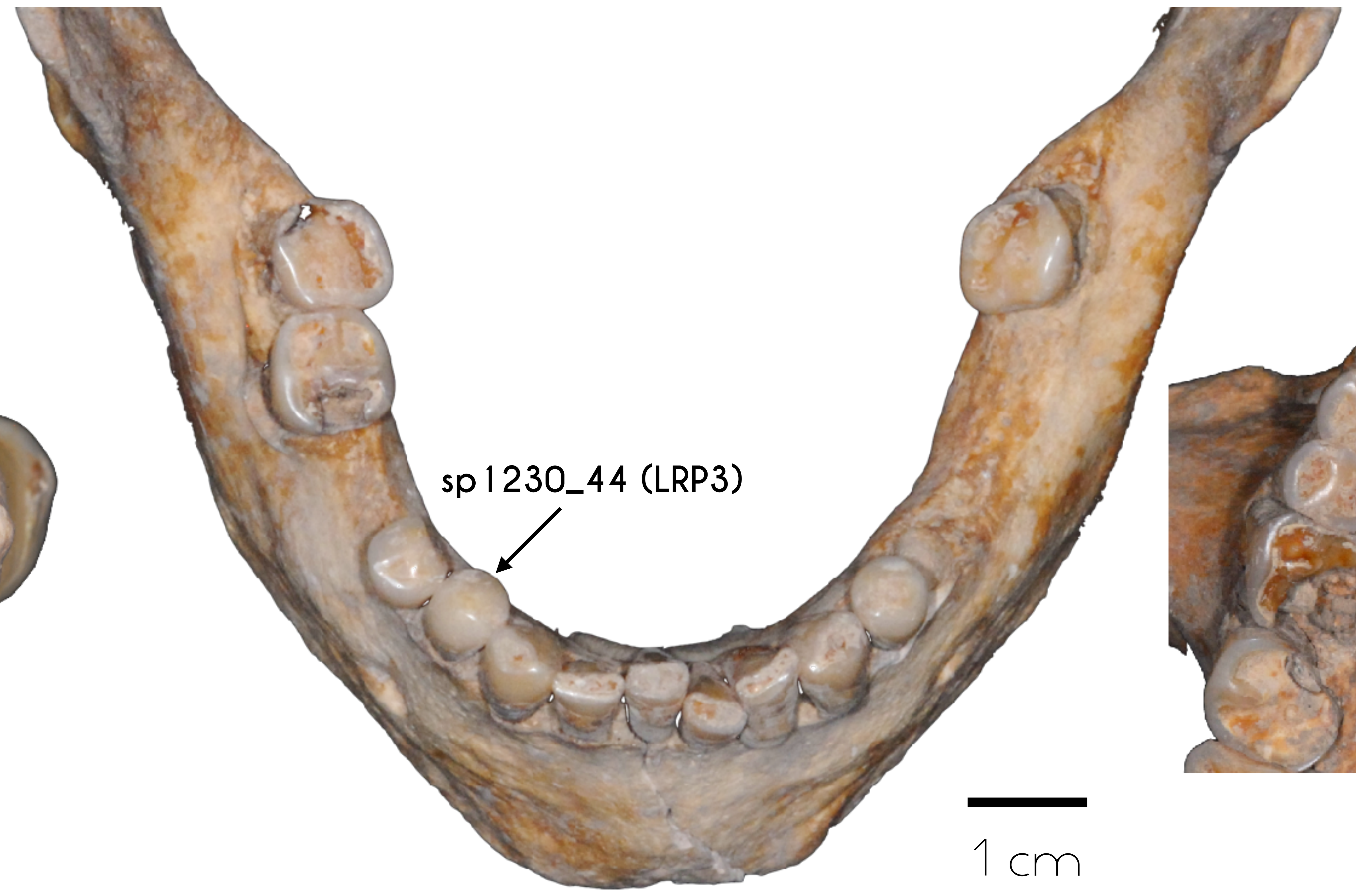

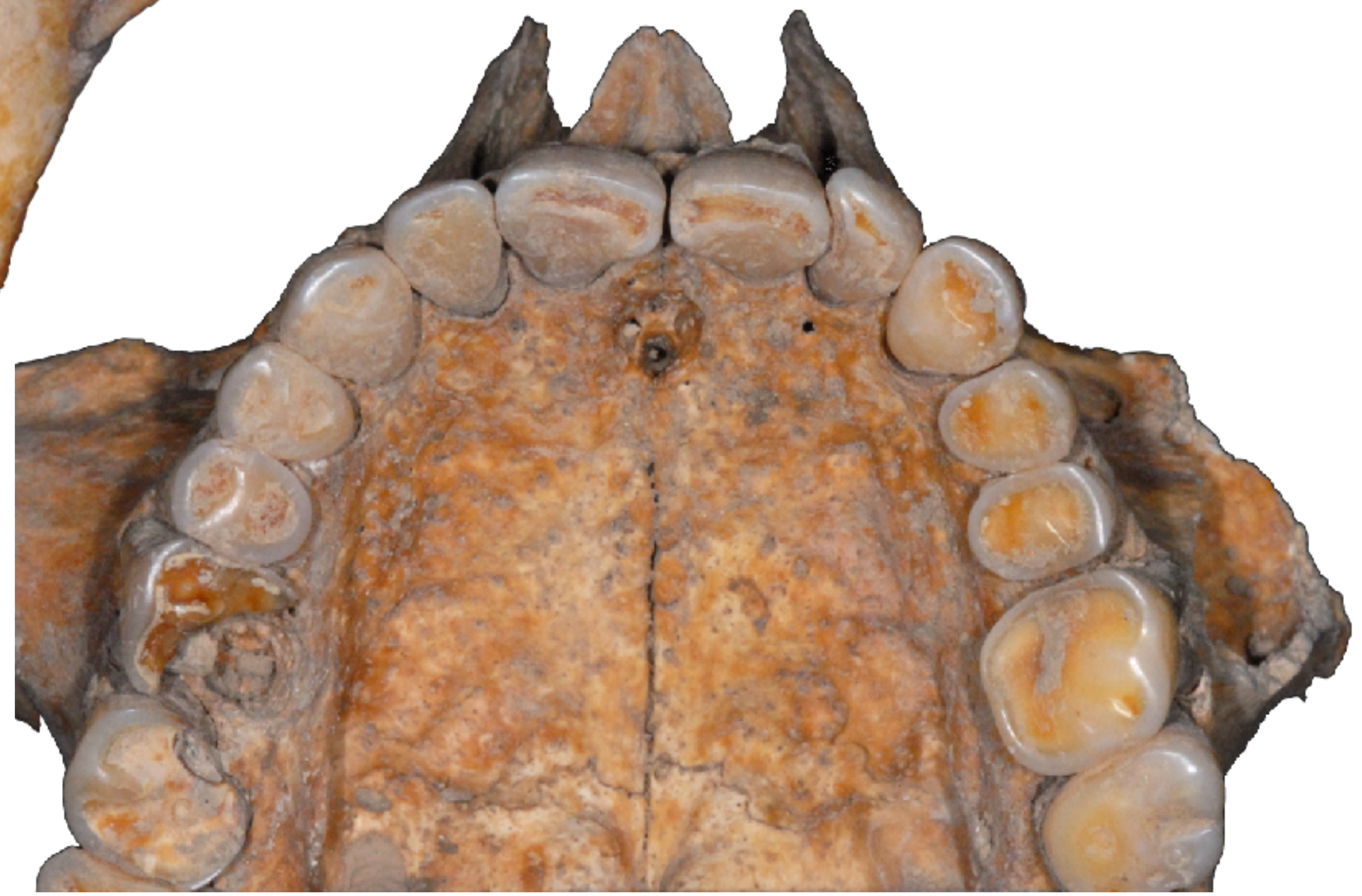

DEG: degree; **DIR:** direction; **FOR:** form - **CAR**: carious lesion - **Pulp EXP:** pulp exposure - **IMP**: impaction - **FEN:** fenestration; **CAL:** calculus; **NT:** tooth loss in neighbouring teeth; **ANTA:** antagonist

Sp1230_44

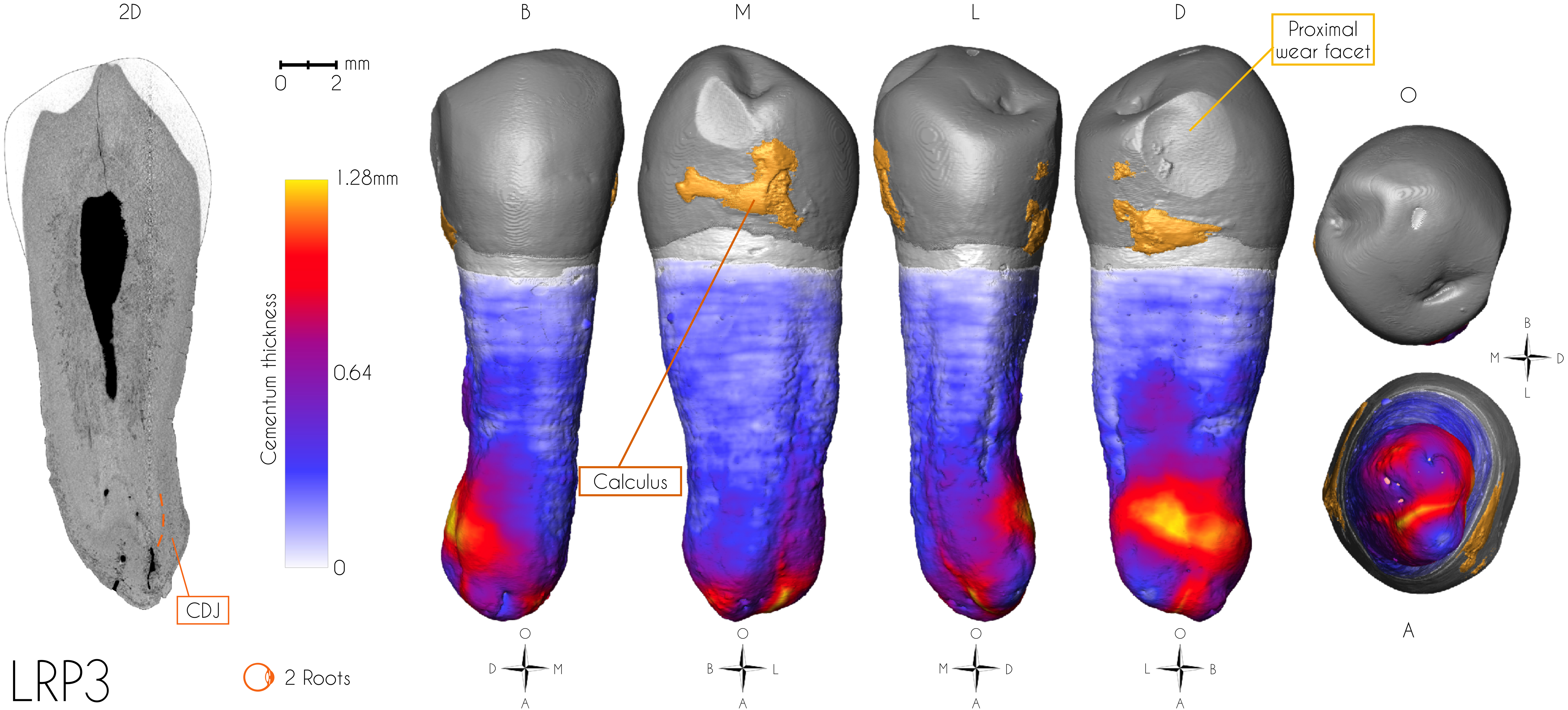

Microtomography - Thickness 3D maps				
MAX THI (µm)	LOC MAX	LOC MIN	PREF LOC	Note
1280	1d	m	No	2 roots

MAX THI: maximum thickness of cementum;
LOC MAX: location of maximum thickness;
LOC MIN: location of minimum thickness;
(1: apical root third; 2: middle root third; 3: cervical root third; m: mesial; d: distal; <: buccal; >: lingual)
PREF LOC: preferential location;

Excluded

Sp39_35

Tooth	Occlusal Wear			CAR Si/Sta	Pulp EXP	IMP	Hypercementosis	Bone Context			
	DEG	DIR	FOR					FEN	CAL	NT	ANTA
**LLP4**	**8**	**4**	**2**	**-**	**0**	**0**	**1.2.m**	**0**	**0**	**2**	**1**

LLP4

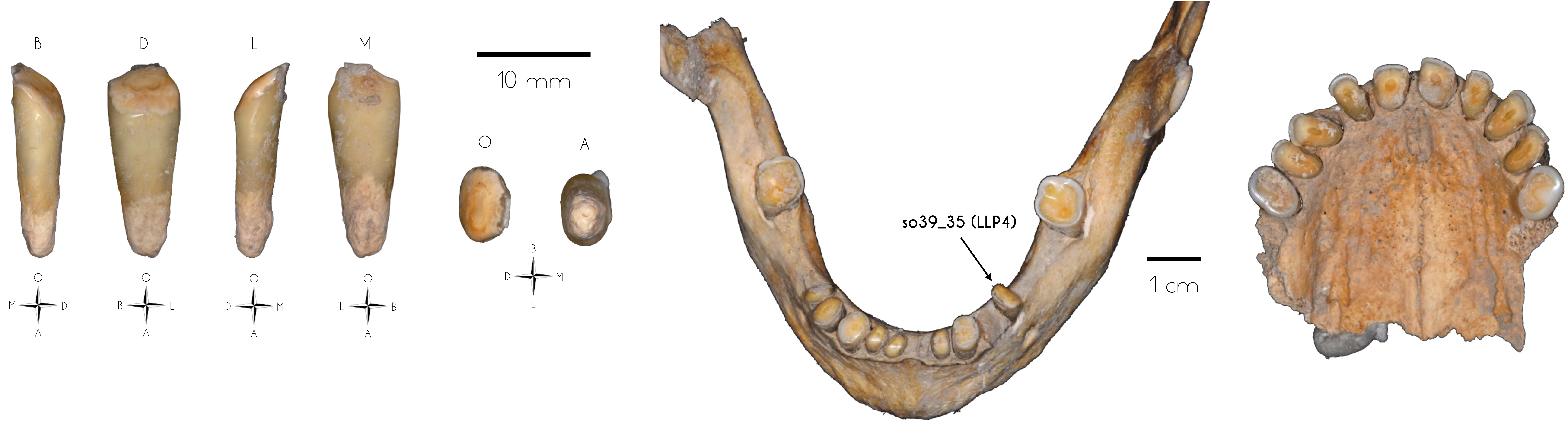

DEG: degree; **DIR:** direction; **FOR:** form - **CAR**: carious lesion - **Pulp EXP:** pulp exposure - **IMP**: impaction - **FEN:** fenestration; **CAL:** calculus; **NT:** tooth loss in neighbouring teeth; **ANTA:** antagonist

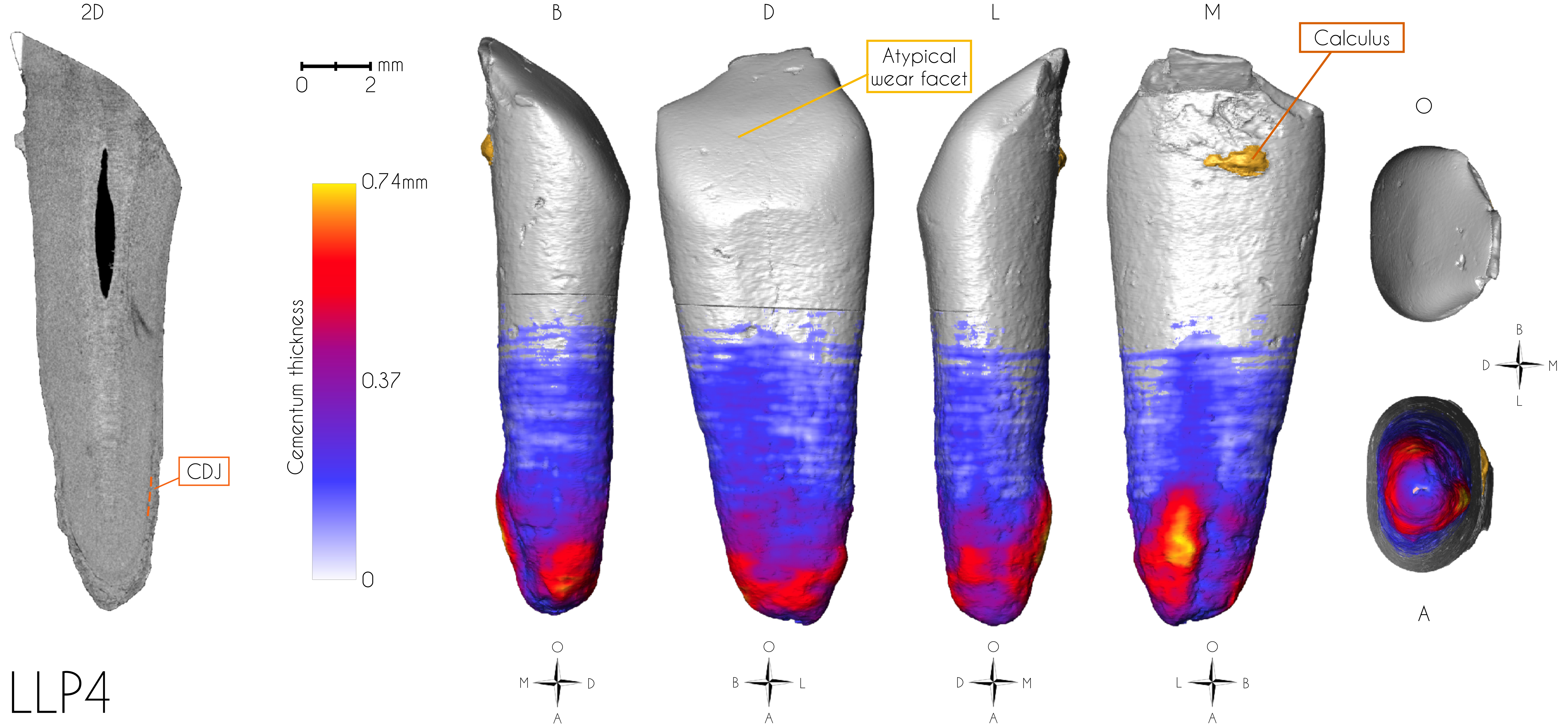

Sp39_35

Microtomography - Thickness 3D maps				
MAX THI (µm)	LOC MAX	LOC MIN	PREF LOC	Note
740	1m	No	Yes	-

MAX THI: maximum thickness of cementum;
LOC MAX: location of maximum thickness;
LOC MIN: location of minimum thickness;
(1: apical root third; 2: middle root third; 3: cervical root third; m: mesial; d: distal; <: buccal; >: lingual)
PREF LOC: preferential location;

Hyperfunctional

LLP4

Face	Range	Light	Width	Height	Columns/Rows	Factor	Led Color	Gain	Gamma	Overlapping
M	1310	5.24%	1.67mm	3.04mm	2/5	2	Blue	1.47	1.61	10 %

Sp39_35

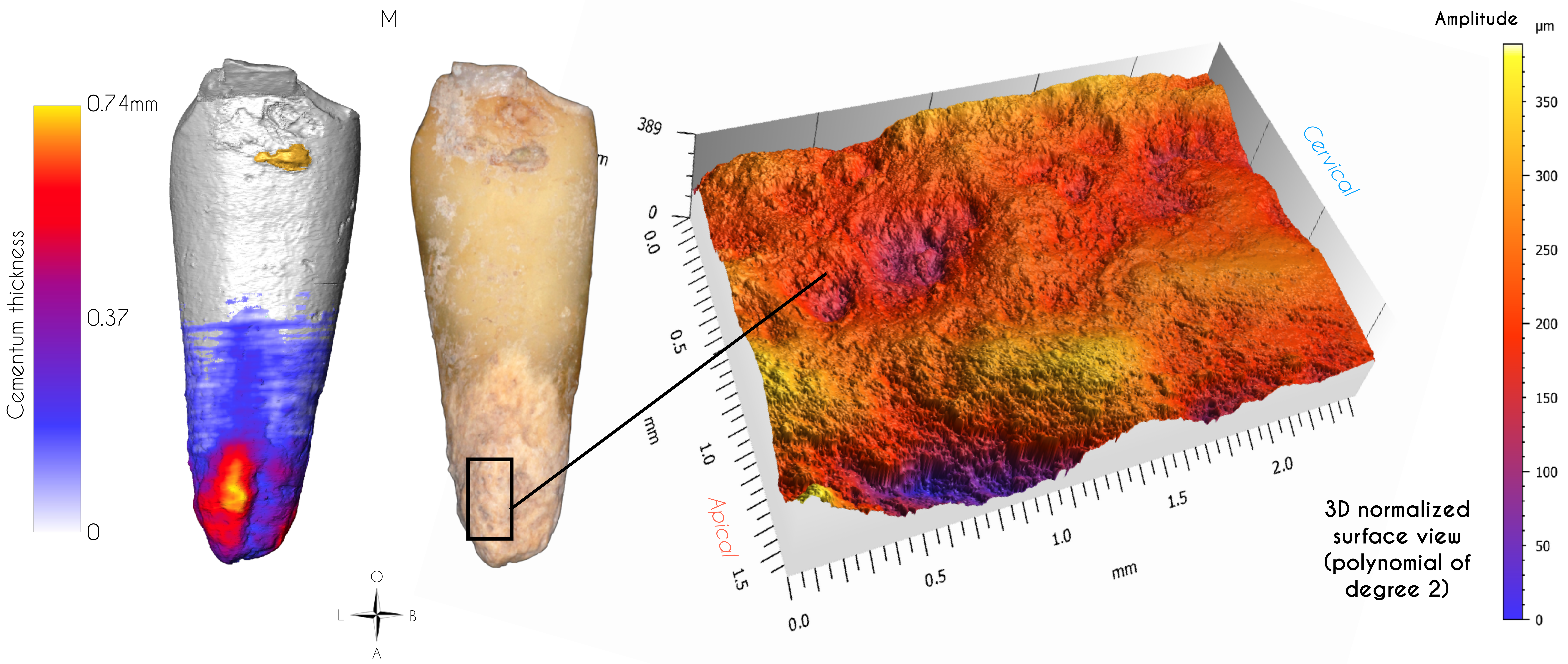

Confocal microscopy - Topography 3D maps			
LOC MAX	MAX VE (µm)	ST	Note
1m	400	+R2	-

LOC MAX: location of maximum thickness (1: apical root third; 2: middle root third; 3: cervical root third; m: mesial; d: distal; <: buccal; >: lingual);
MAX VE: maximum vertical elevation;
ST: surface texture; : scored (**+**) if it is high, (**-**) if it is low; **S:** smooth or **R:** rough; the frequency of occurrence of the elevations scored (**1**) if the frequency is high, (**2**) if the frequency is low.

Hyperfunctional

Sp199_11

Tooth	Occlusal Wear			CAR Si/Sta	Pulp EXP	IMP	Hypercementosis	Bone Context			
	DEG	DIR	FOR					FEN	CAL	NT	ANTA
**URI 1**	**6**	**6**	**4**	**-**	**0**	**0**	**1.2.m**	**0**	**0**	**0**	**1**

URI1

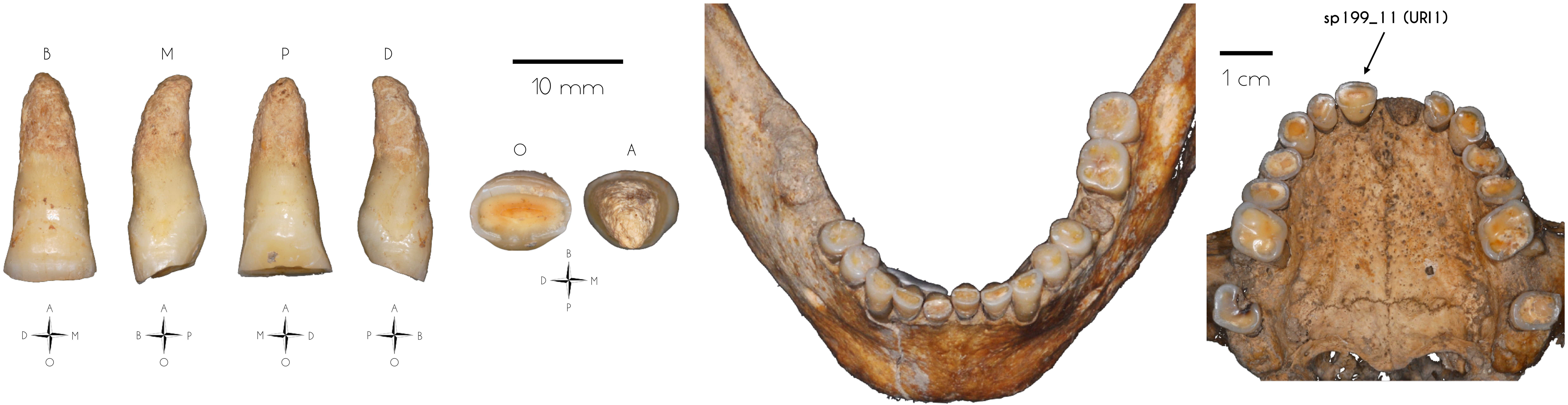

DEG: degree; **DIR:** direction; **FOR:** form - **CAR**: carious lesion - **Pulp EXP:** pulp exposure - **IMP**: impaction - **FEN:** fenestration; **CAL:** calculus; **NT:** tooth loss in neighbouring teeth; **ANTA:** antagonist

Sp199_11

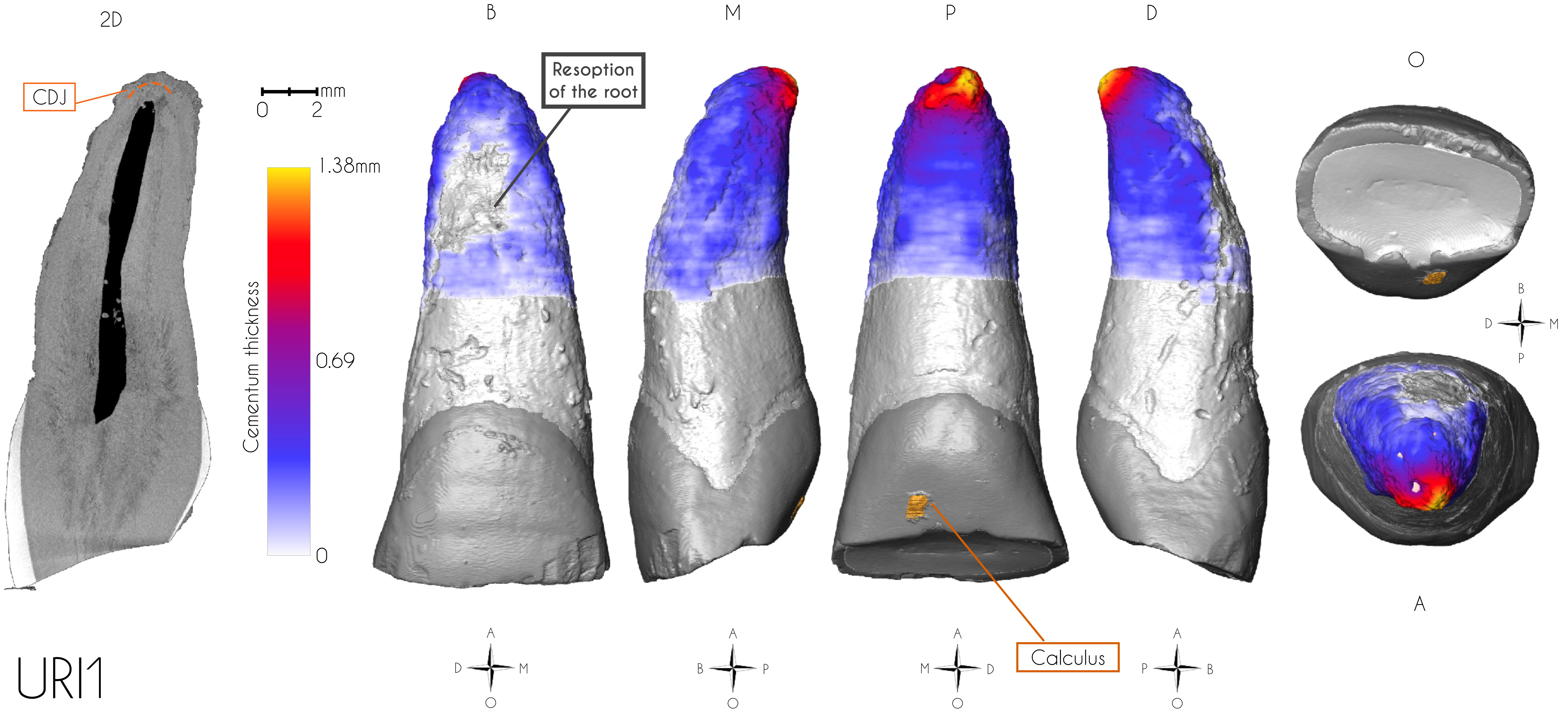

URI1

Microtomography - Thickness 3D maps				
MAX THI (μm)	LOC MAX	LOC MIN	PREF LOC	Note
1380	1>	<	Yes	RES (1<)

MAX THI: maximum thickness of cementum;
LOC MAX: location of maximum thickness;
LOC MIN: location of minimum thickness;
PREF LOC: preferential location;
RES: resorption of the root;
(1: apical root third; 2: middle root third; 3: cervical root third; m: mesial; d: distal; <: buccal; >: lingual)

Hyperfunctional

URI1

Face	Range	Light	Width	Height	Columns/Rows	Factor	Led Color	Gain	Gamma	Overlapping
P	577	4.00%	0.88mm	1.85mm	1/3	2	Blue	1.49	1.79	10 %

Sp199_11

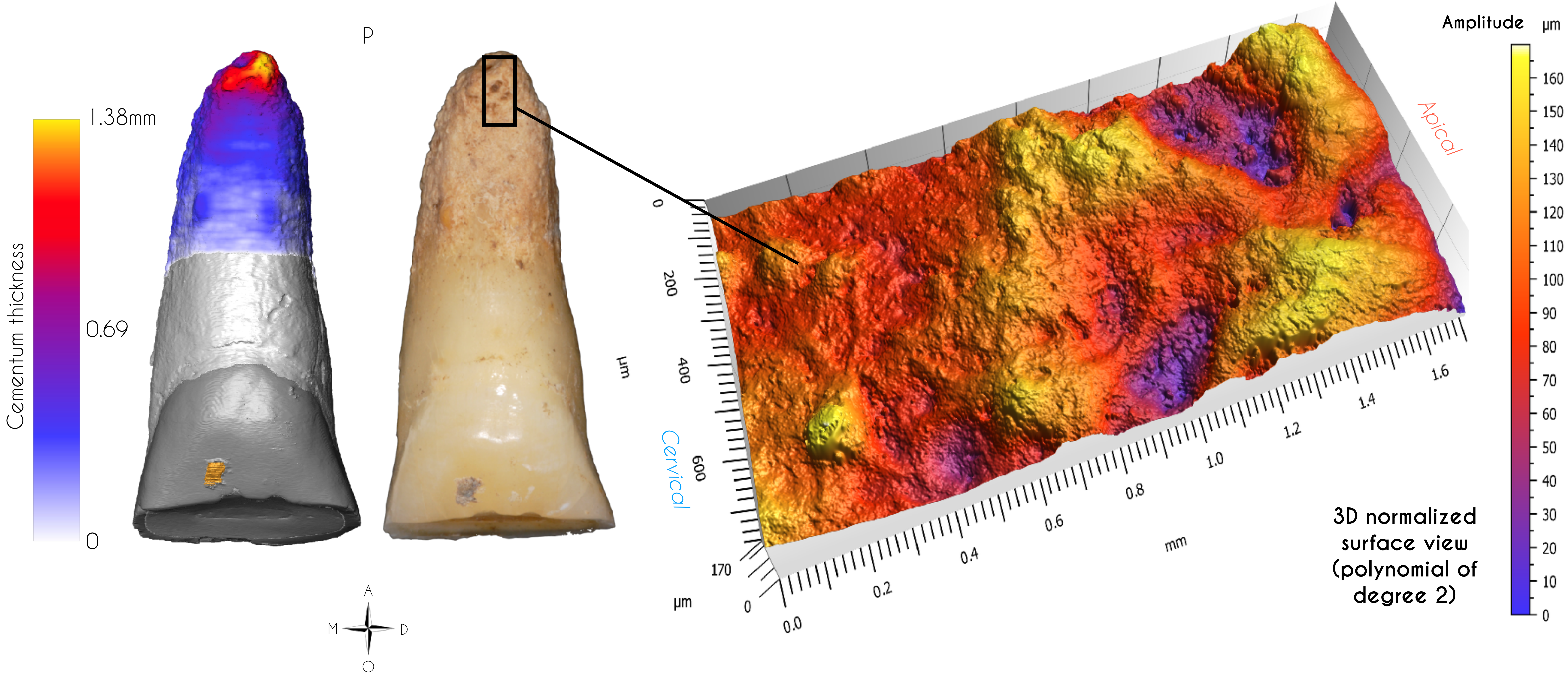

Confocal microscopy - Topography 3D maps			
LOC MAX	MAX VE (µm)	ST	Note
1>	170	+R1	-

LOC MAX: location of maximum thickness (1: apical root third; 2: middle root third; 3: cervical root third; m: mesial; d: distal; <: buccal; >: lingual);
MAX VE: maximum vertical elevation;
ST: surface texture; : scored (**+**) if it is high, (**-**) if it is low; **S:** smooth or **R:** rough; the frequency of occurrence of the elevations scored (**1**) if the frequency is high, (**2**) if the frequency is low.

Hyperfunctional

Sp199_12 | | URI2

Tooth	Occlusal Wear			CAR Si/Sta	Pulp EXP	IMP	Hypercementosis	Bone Context			
	DEG	DIR	FOR					FEN	CAL	NT	ANTA
**URI2**	**6**	**4**	**4**	**-**	**0**	**0**	**1.3.m**	**0**	**0**	**0**	**1**

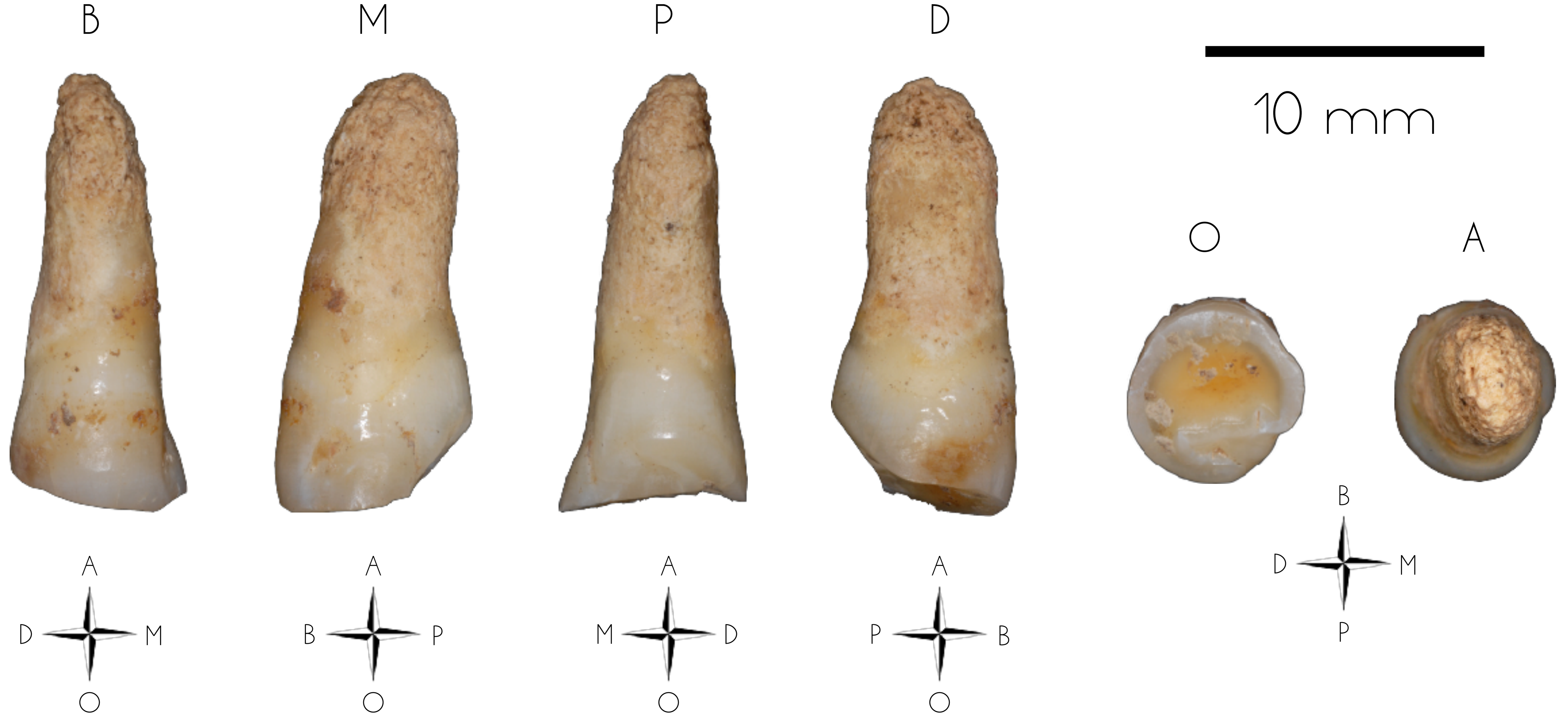

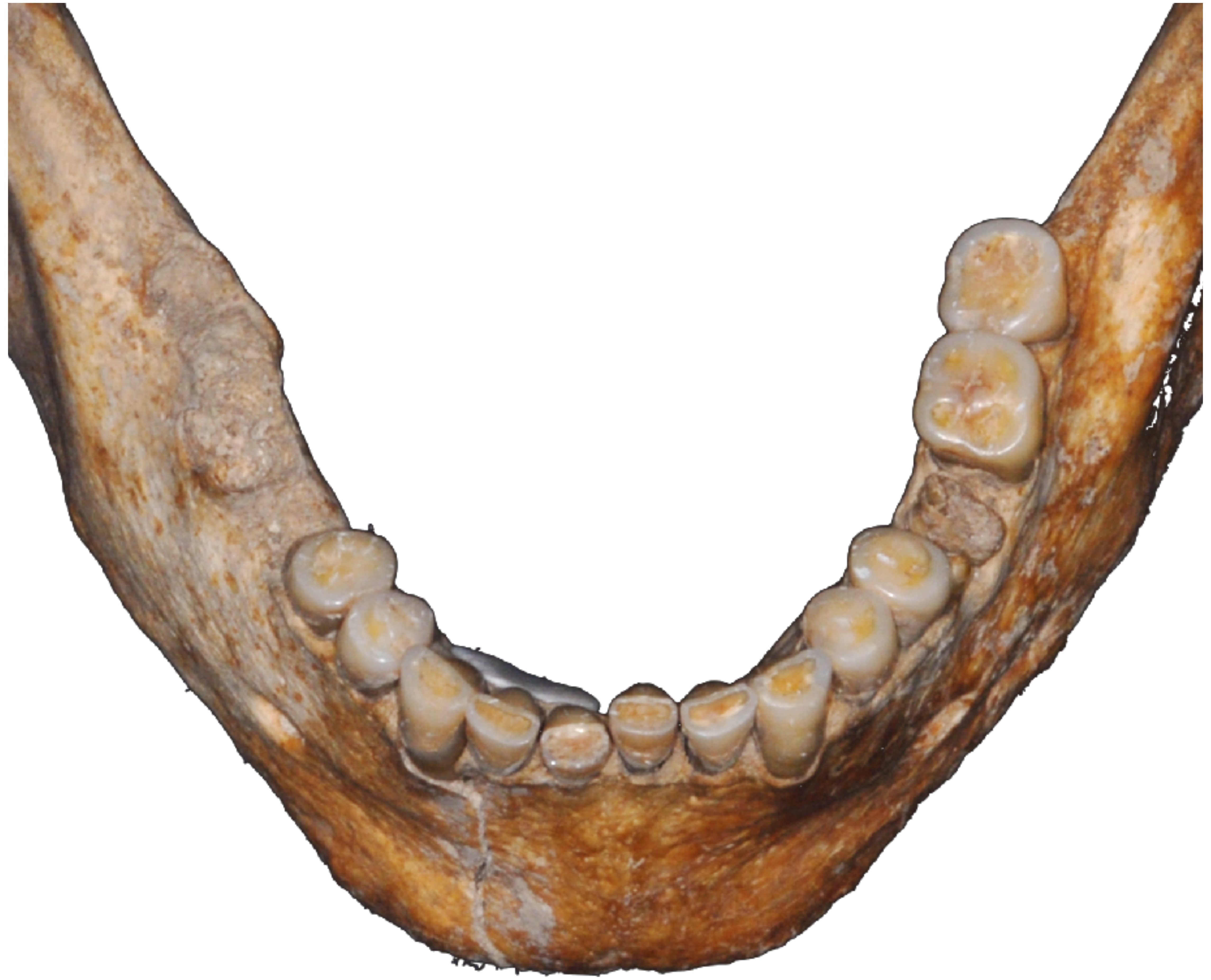

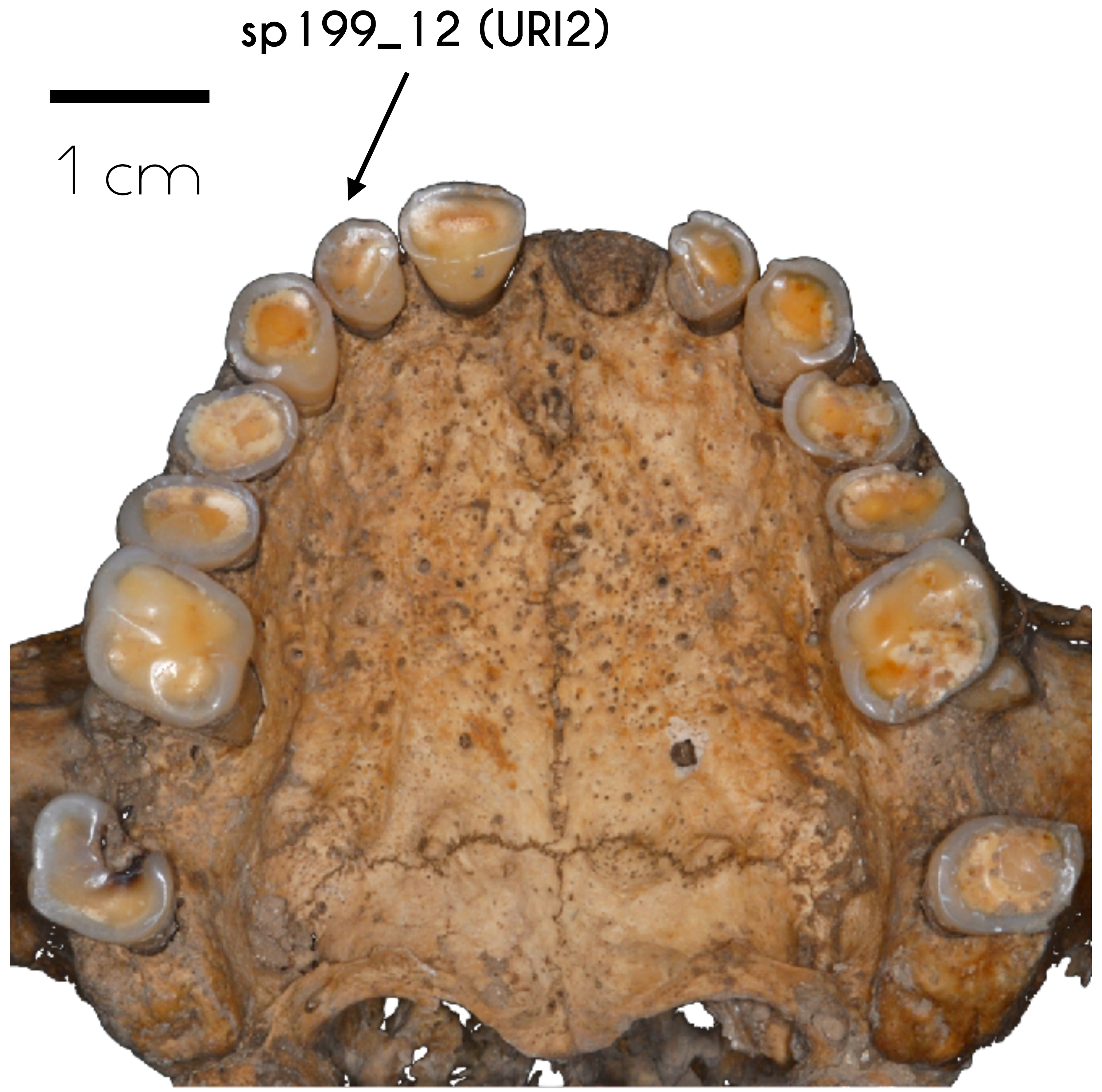

DEG: degree; **DIR:** direction; **FOR:** form - **CAR**: carious lesion - **Pulp EXP:** pulp exposure - **IMP**: impaction - **FEN:** fenestration; **CAL:** calculus; **NT:** tooth loss in neighbouring teeth; **ANTA:** antagonist

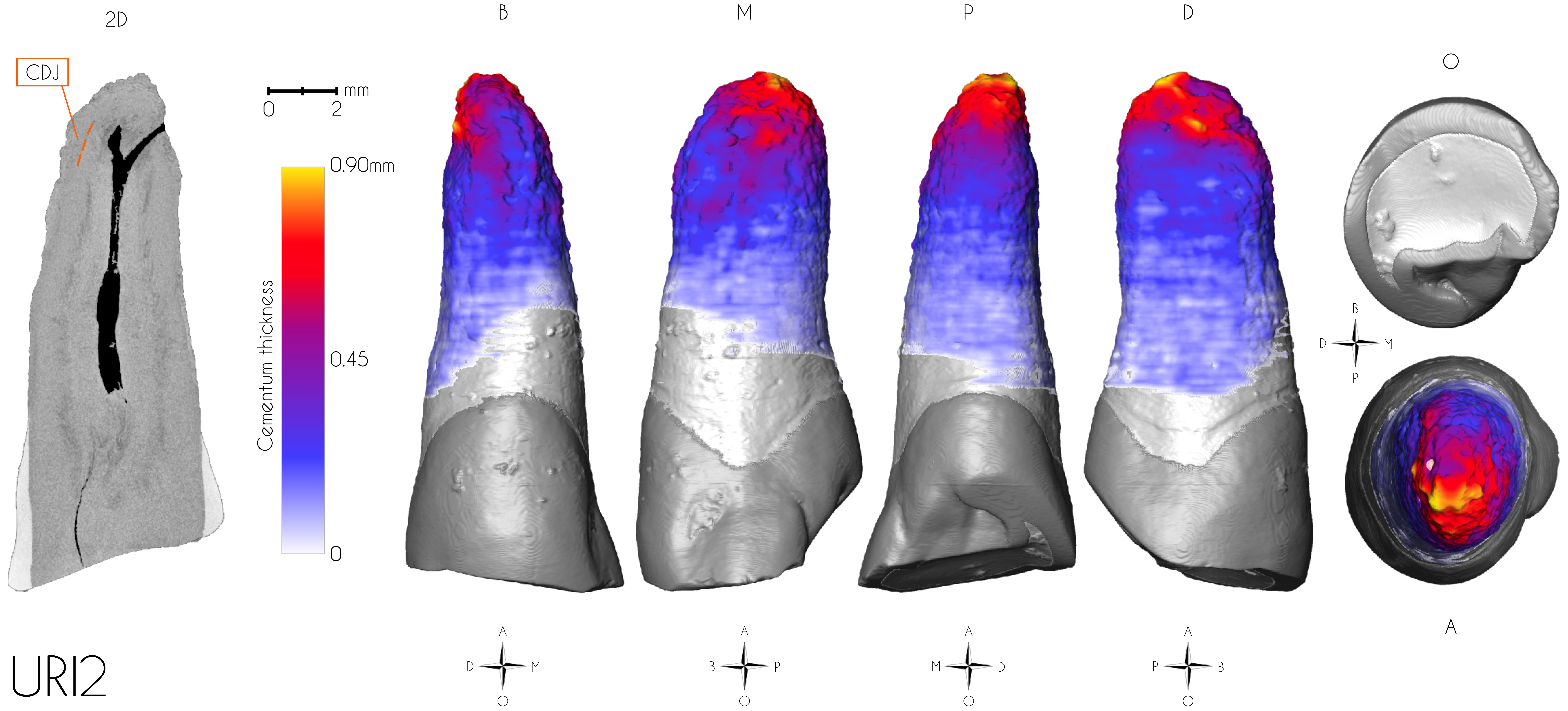

Sp199_12

Microtomography - Thickness 3D maps				
MAX THI (μm)	LOC MAX	LOC MIN	PREF LOC	Note
900	1>	<	Yes	-

MAX THI: maximum thickness of cementum;
LOC MAX: location of maximum thickness;
LOC MIN: location of minimum thickness;
(1: apical root third; 2: middle root third; 3: cervical root third; m: mesial; d: distal; <: buccal; >: lingual)
PREF LOC: preferential location;

Hyperfunctional

URI2

Face	Range	Light	Width	Height	Columns/Rows	Factor	Led Color	Gain	Gamma	Overlapping
P	949	6.91%	0.88mm	1.85mm	1/3	2	Blue	1.49	1.79	10 %

Sp199_12

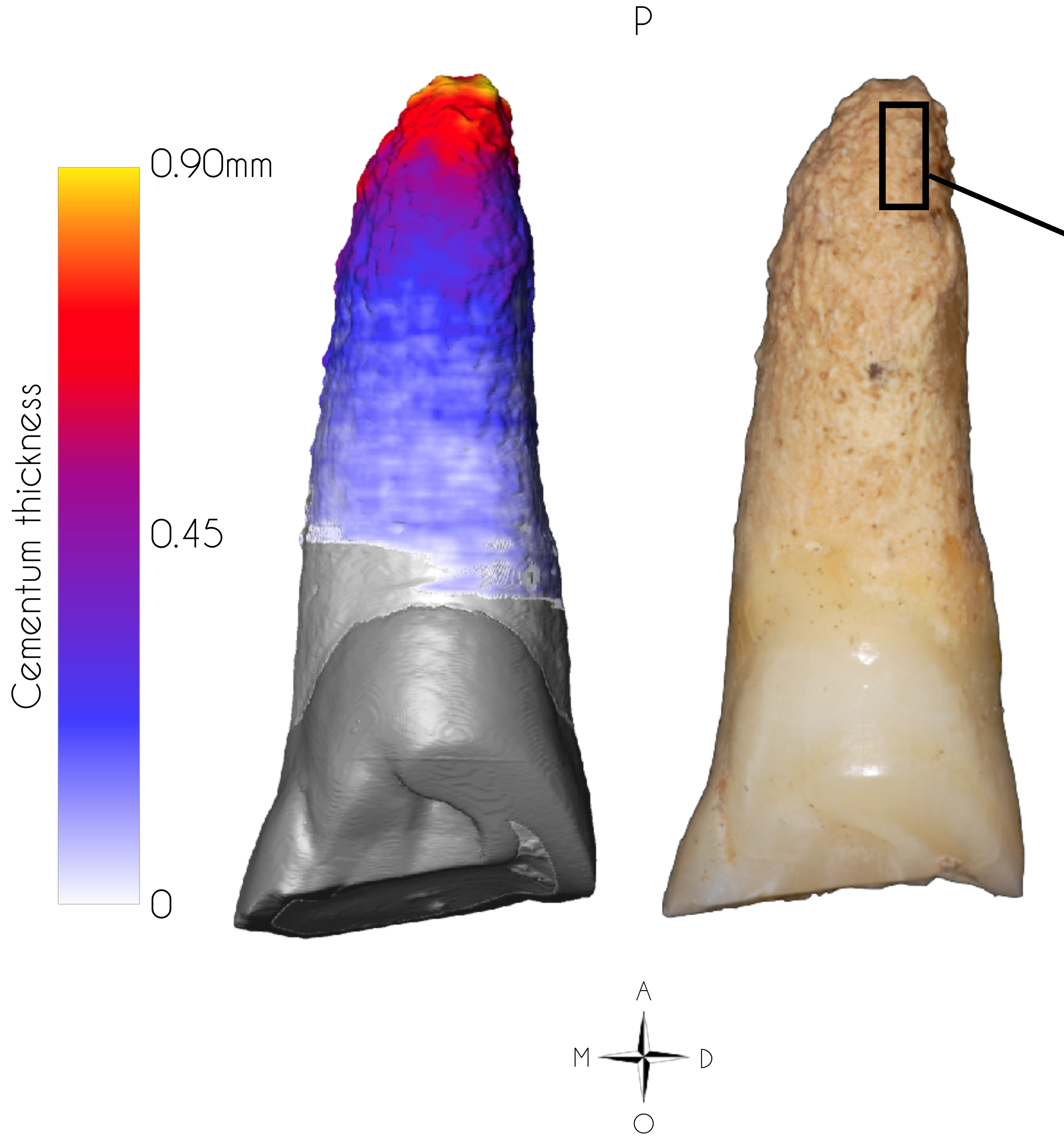

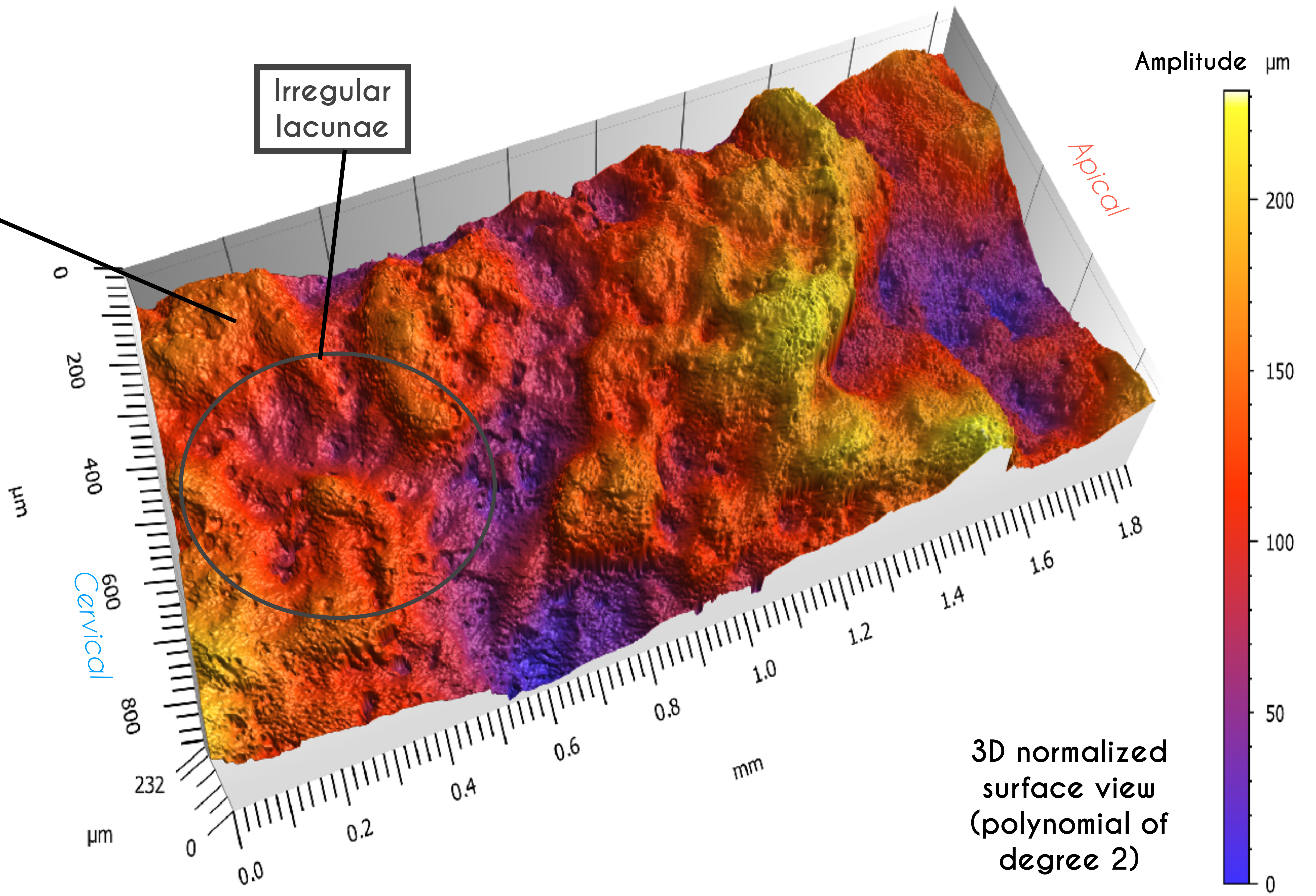

Confocal microscopy - Topography 3D maps			
LOC MAX	MAX VE (µm)	ST	Note
1>	230	+R1	IL

LOC MAX: location of maximum thickness (1: apical root third; 2: middle root third; 3: cervical root third; m: mesial; d: distal; <: buccal; >: lingual);
MAX VE: maximum vertical elevation;
ST: surface texture; : scored (**+**) if it is high, (**-**) if it is low; **S:** smooth or **R:** rough; the frequency of occurrence of the elevations scored (**1**) if the frequency is high, (**2**) if the frequency is low.
IL: irregular lacunae

Hyperfunctional

URI2

Face	Range	Light	Width	Height	Columns/Rows	Factor	Led Color	Gain	Gamma	Overlapping
BM	882	5.76%	0.88mm	2.44mm	1/4	2	Blue	1.49	1.79	10 %

Sp199_12

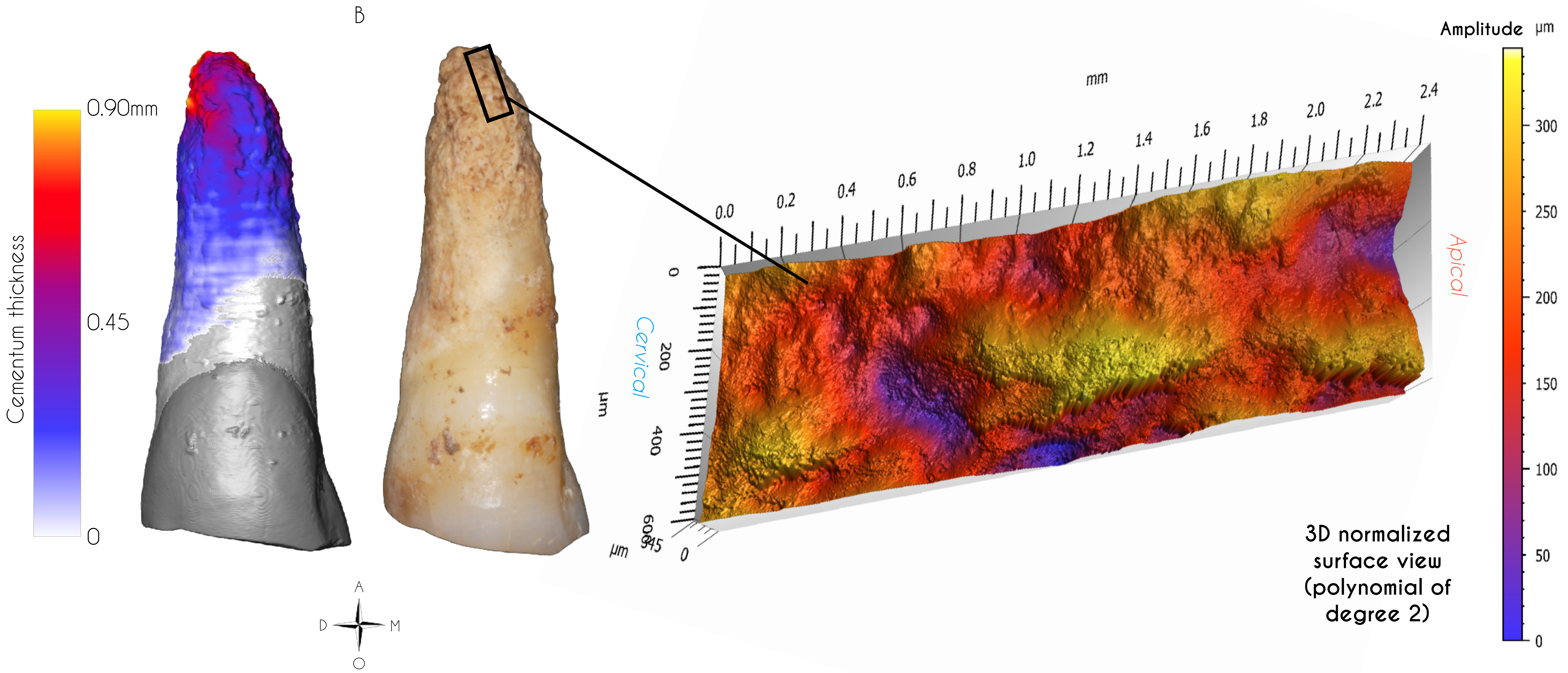

Confocal microscopy - Topography 3D maps			
OF	MAX VE (µm)	ST	Note
1<	350	+R1	-

OF: other face;
MAX VE: maximum vertical elevation;
ST: surface texture; : scored (**+**) if it is high, (**-**) if it is low; **S:** smooth or **R:** rough; the frequency of occurrence of the elevations scored (**1**) if the frequency is high, (**2**) if the frequency is low.

Hyperfunctional

Sp335_13

Tooth	Occlusal Wear			CAR Si/Sta	Pulp EXP	IMP	Hypercementosis	Bone Context			
	DEG	DIR	FOR					FEN	CAL	NT	ANTA
**URC**	**4**	**2**	**2**	**-**	**0**	**0**	**1.2.m**	**0**	**0**	**2**	**1**

URC

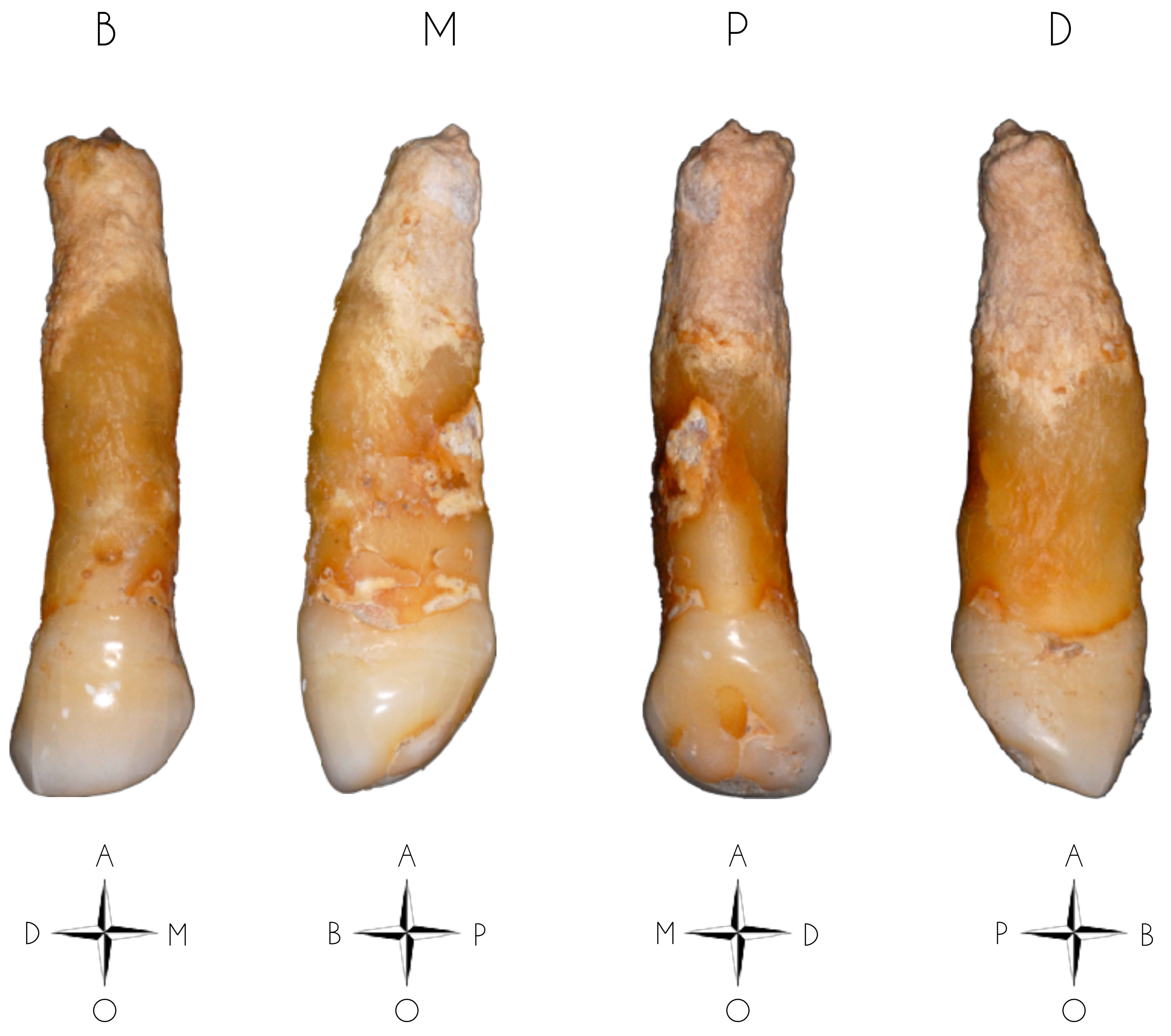

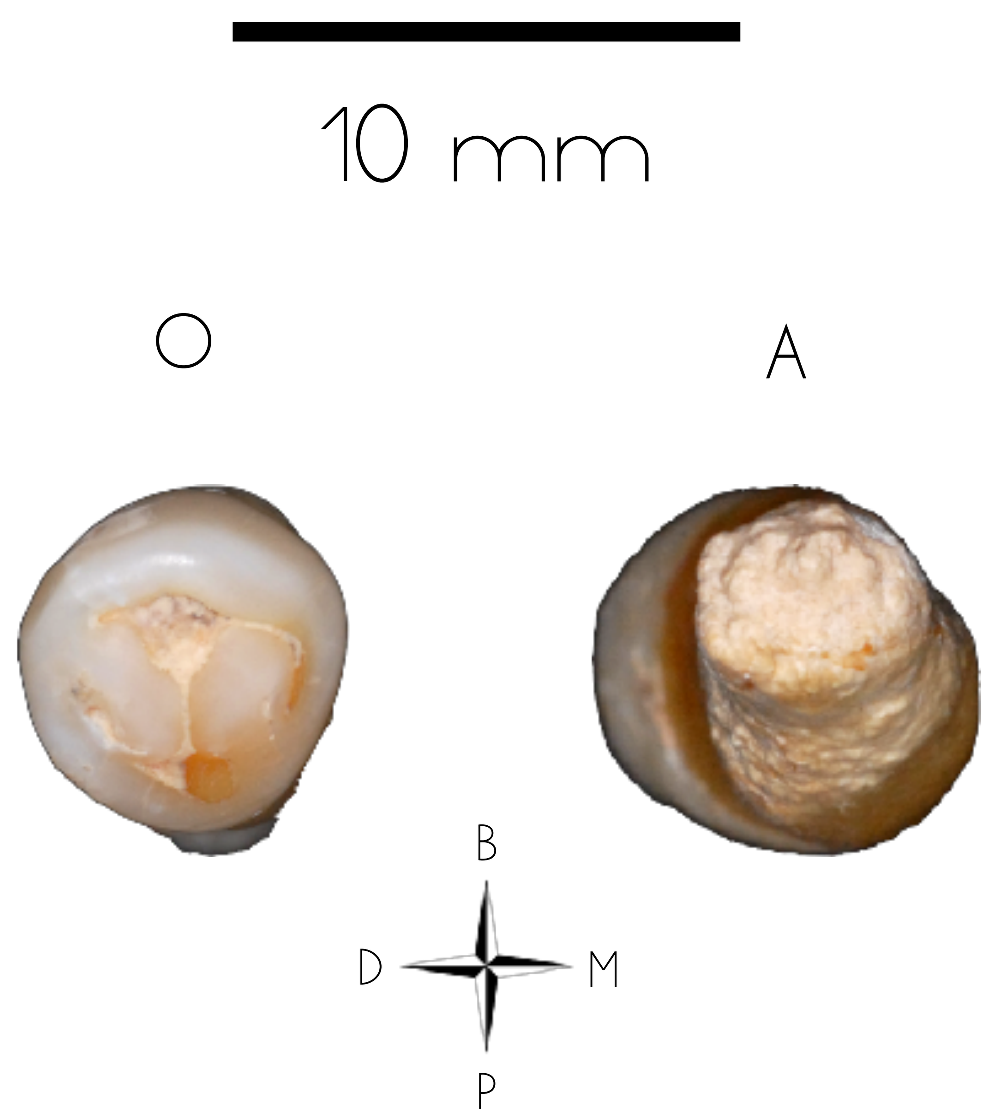

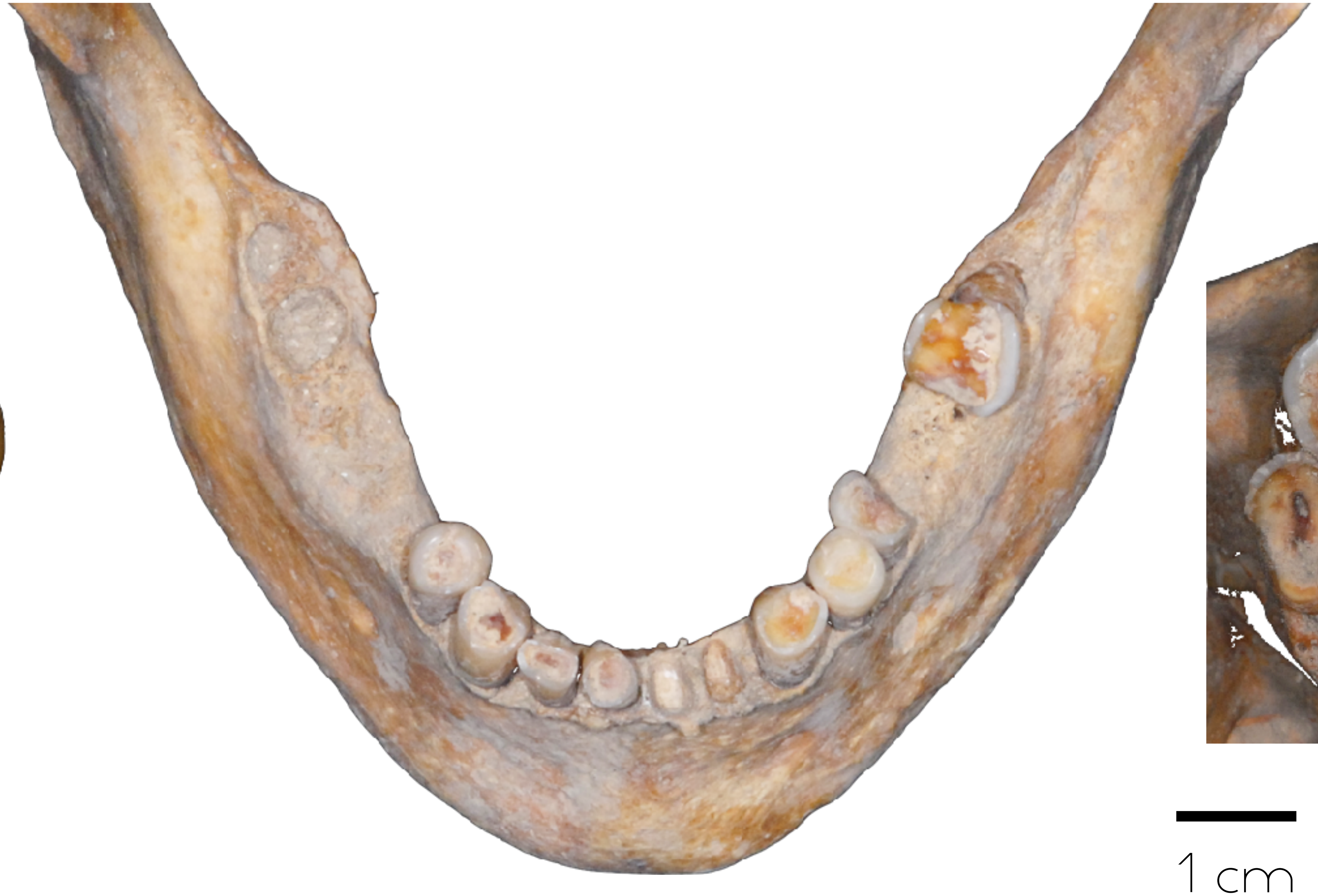

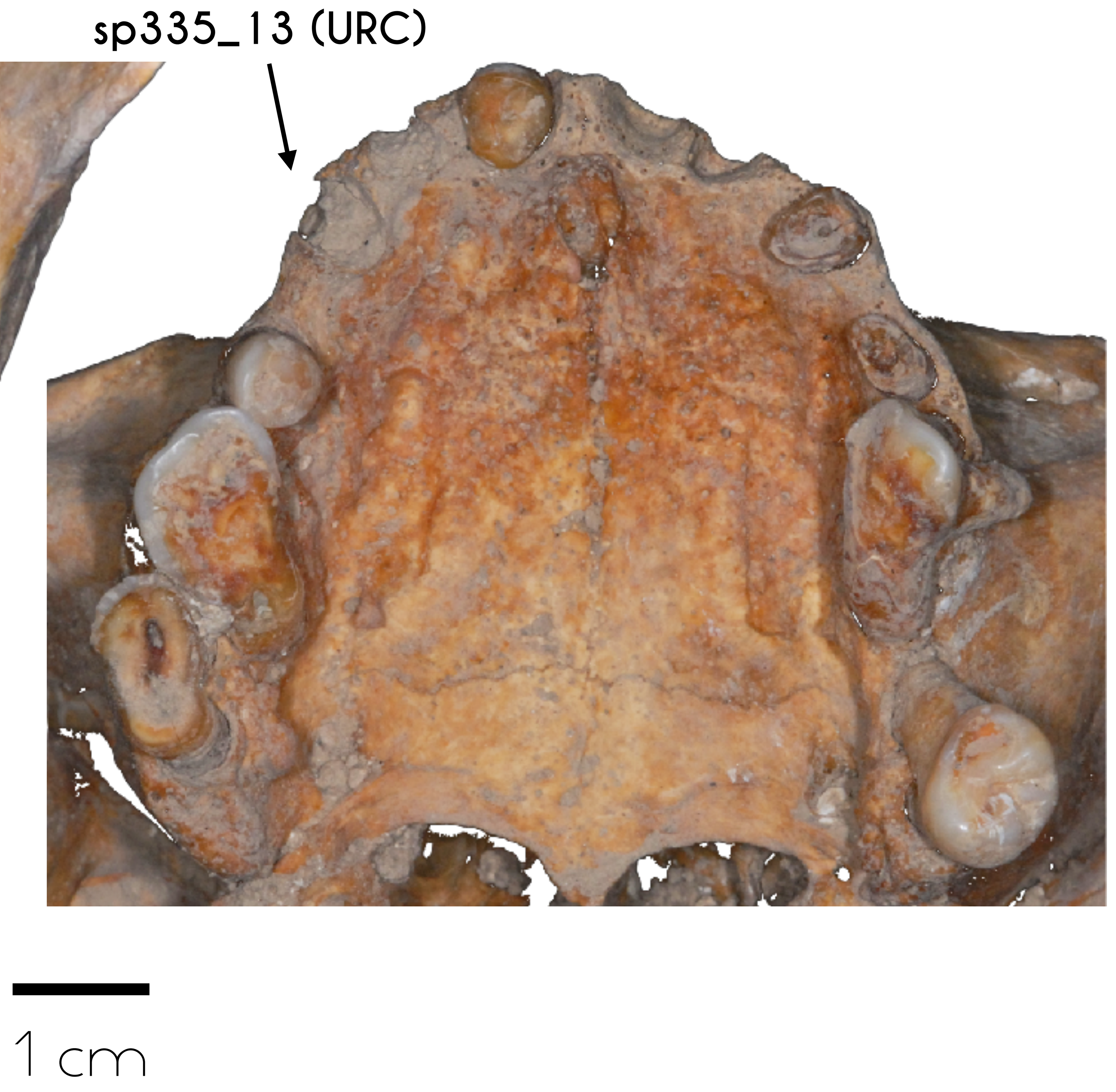

DEG: degree; **DIR:** direction; **FOR:** form - **CAR**: carious lesion - **Pulp EXP:** pulp exposure - **IMP**: impaction - **FEN:** fenestration; **CAL:** calculus; **NT:** tooth loss in neighbouring teeth; **ANTA:** antagonist

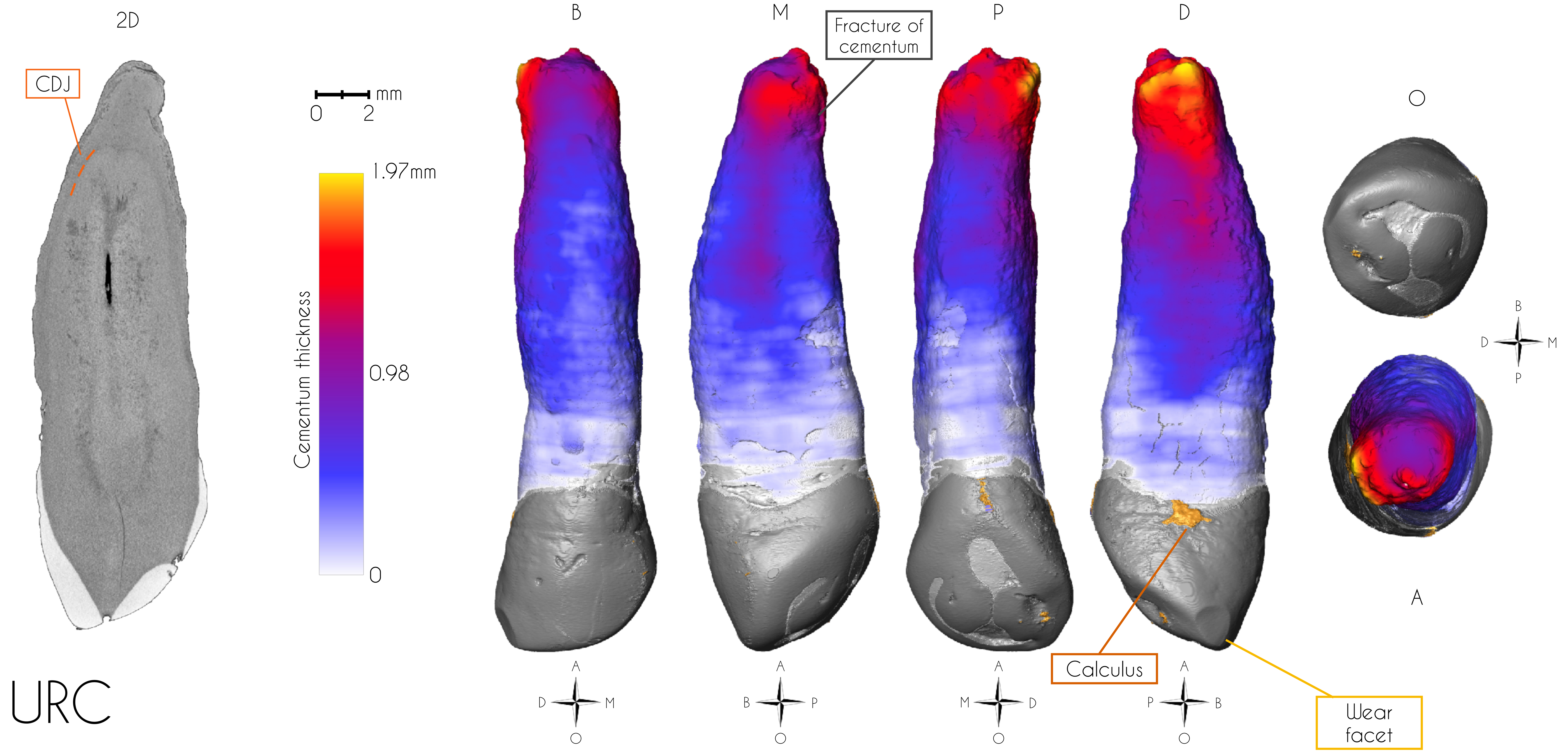

Sp335_13

Microtomography - Thickness 3D maps				
MAX THI (µm)	LOC MAX	LOC MIN	PREF LOC	Note
1970	1d	<	Yes	FR (1>m)

MAX THI: maximum thickness of cementum;
LOC MAX: location of maximum thickness;
LOC MIN: location of minimum thickness;
PREF LOC: preferential location;
FR: fracture of cementum;
(1: apical root third; 2: middle root third; 3: cervical root third; m: mesial; d: distal; <: buccal; >: lingual)

Hyperfunctional

URC

Face	Range	Light	Width	Height	Columns/Rows	Factor	Led Color	Gain	Gamma	Overlapping
D	960	6 %	0.88mm	6.01mm	1/10	2	Blue	1.5	1.8	10 %

Sp335_13

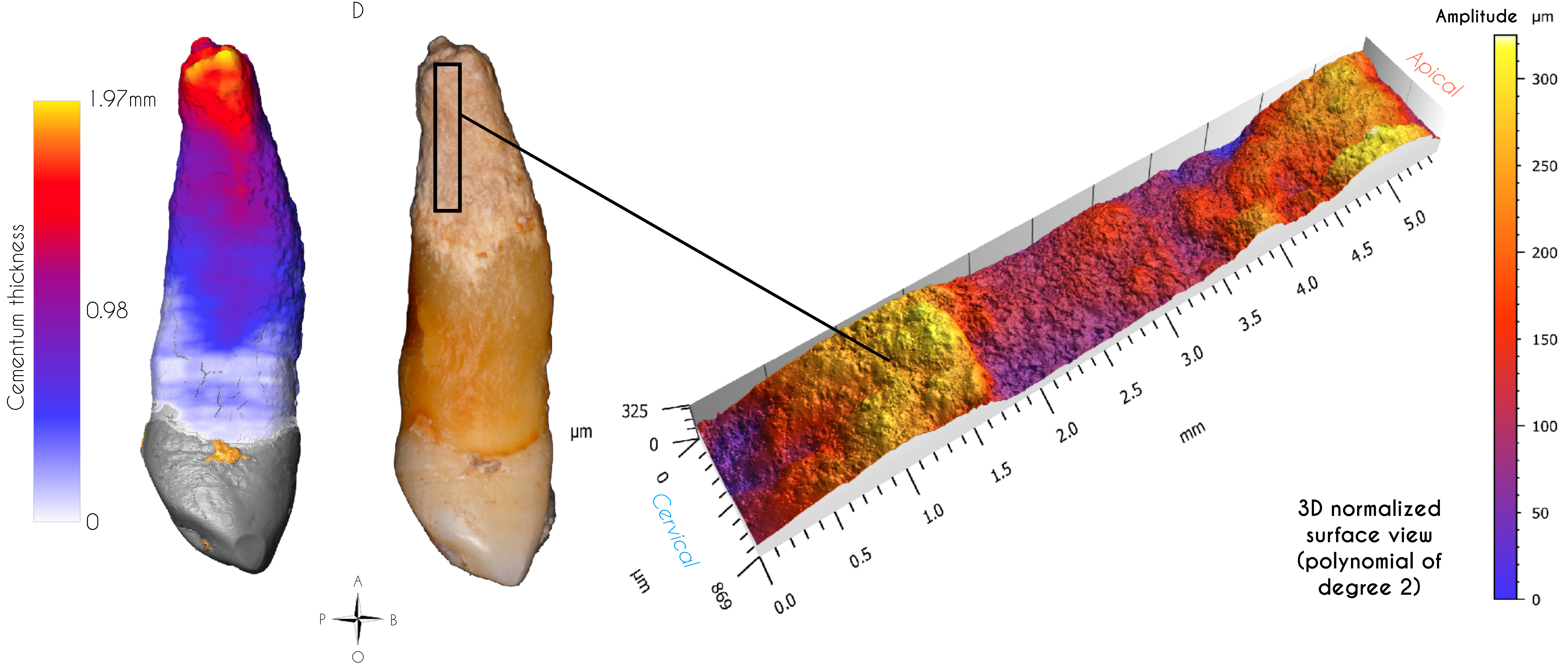

Confocal microscopy - Topography 3D maps			
LOC MAX	MAX VE (µm)	ST	Note
1d	325	+R1	-

LOC MAX: location of maximum thickness (1: apical root third; 2: middle root third; 3: cervical root third; m: mesial; d: distal; <: buccal; >: lingual);
MAX VE: maximum vertical elevation;
ST: surface texture; : scored (+) if it is high, (-) if it is low; **S:** smooth or **R:** rough; the frequency of occurrence of the elevations scored (1) if the frequency is high, (2) if the frequency is low.

Hyperfunctional

Sp479_11

Tooth	Occlusal Wear			CAR Si/Sta	Pulp EXP	IMP	Hypercementosis	Bone Context			
	DEG	DIR	FOR					FEN	CAL	NT	ANTA
URI 1	7	2	2	-	0	0	3.2.M	0	0	0	1

URI1

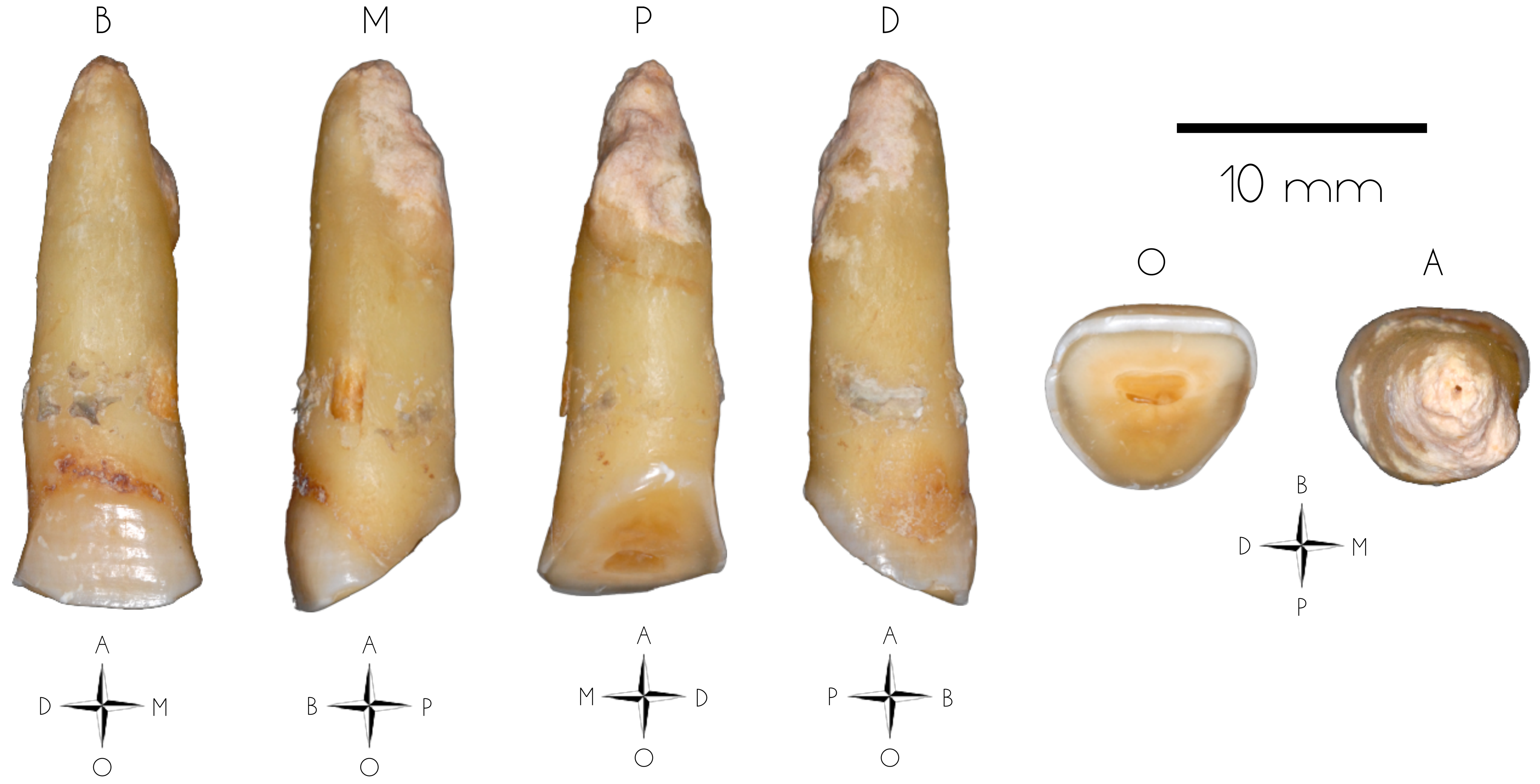

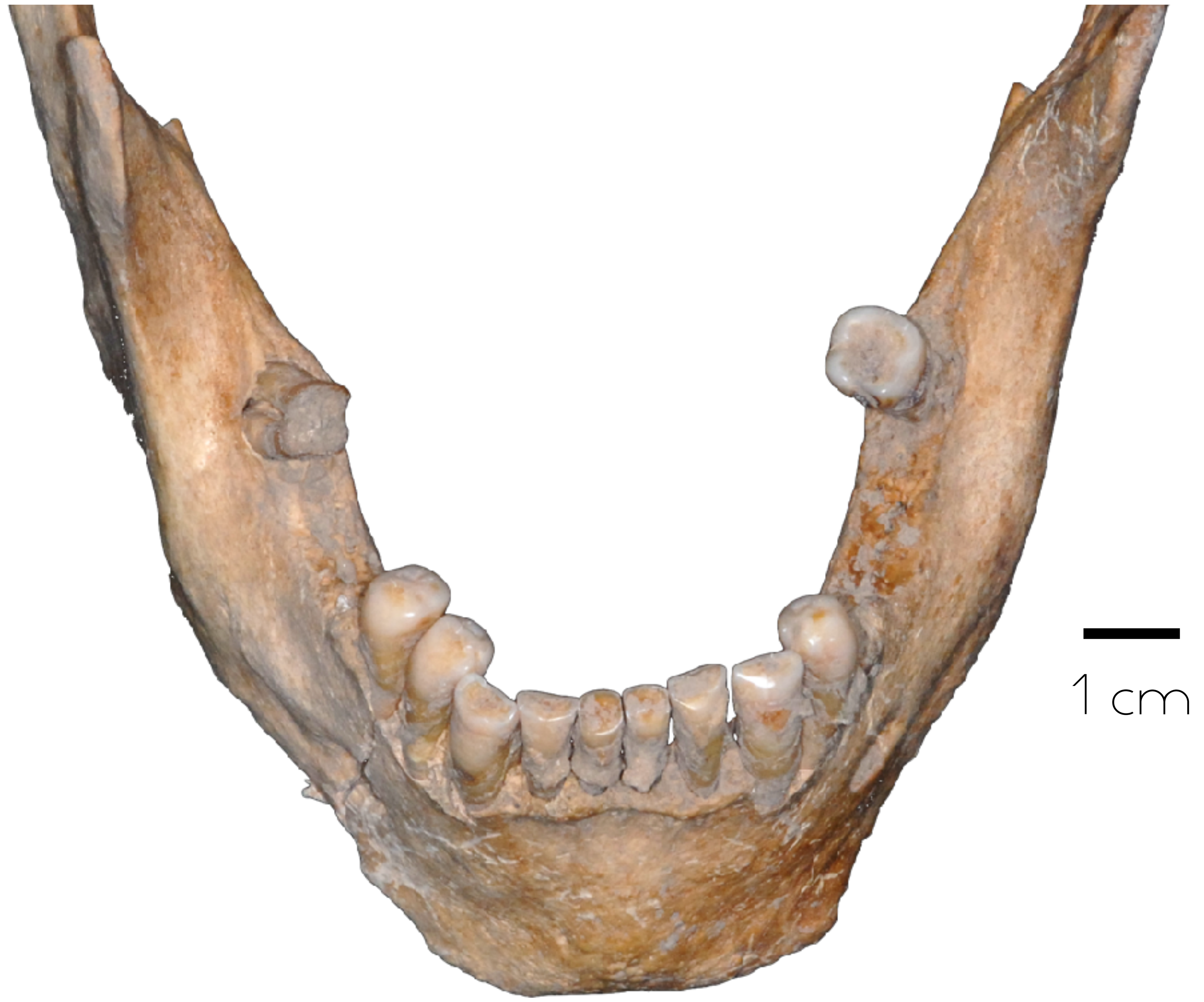

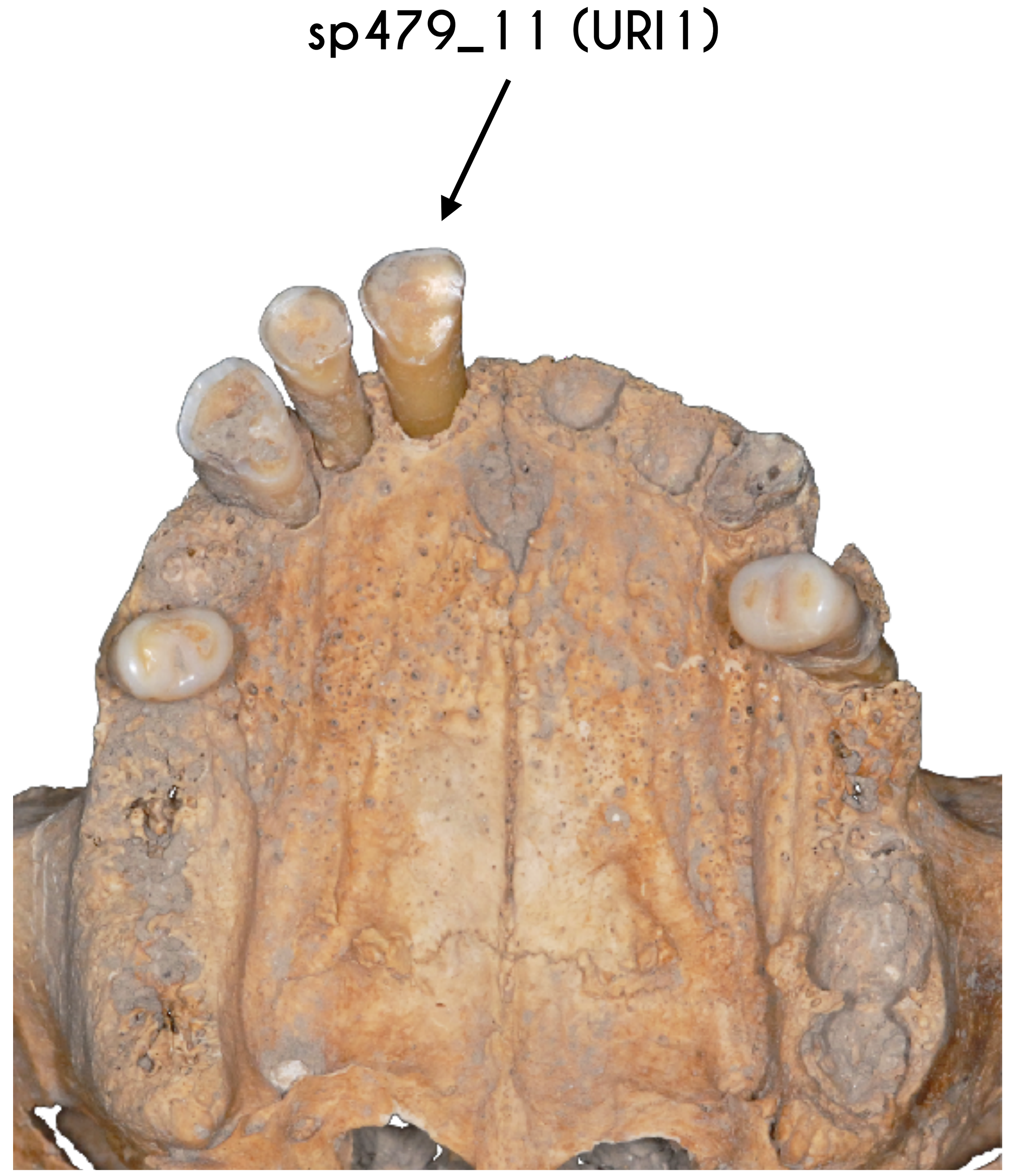

DEG: degree; **DIR:** direction; **FOR:** form - **CAR**: carious lesion - **Pulp EXP:** pulp exposure - **IMP**: impaction - **FEN:** fenestration; **CAL:** calculus; **NT:** tooth loss in neighbouring teeth; **ANTA:** antagonist

Sp479_11

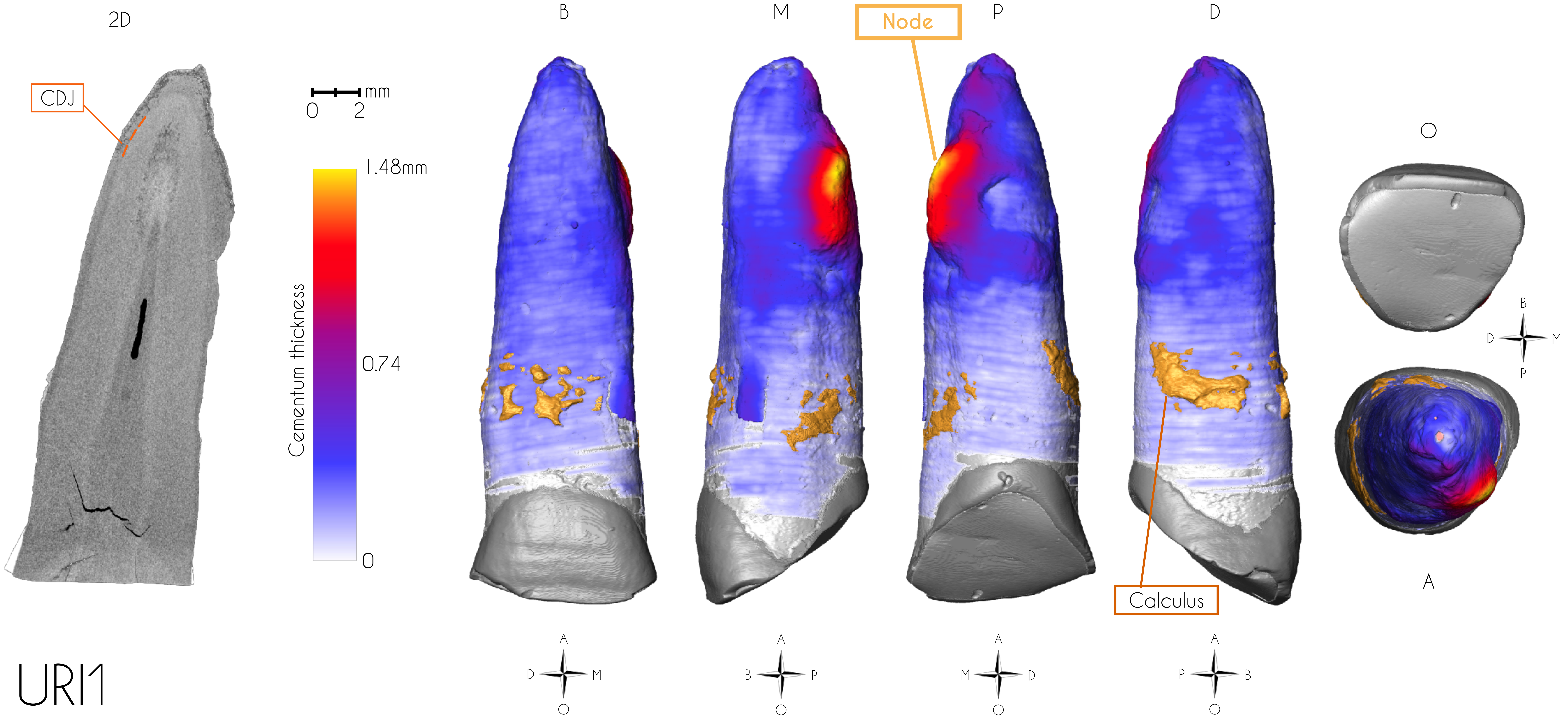

Microtomography - Thickness 3D maps				
MAX THI (μm)	LOC MAX	LOC MIN	PREF LOC	Note
1480	1>	<	Yes	NOD (m>)

MAX THI: maximum thickness of cementum;
LOC MAX: location of maximum thickness;
LOC MIN: location of minimum thickness;
PREF LOC: preferential location;
NOD: node;
(1: apical root third; 2: middle root third; 3: cervical root third; m: mesial; d: distal; <: buccal; >: lingual)

Hyperfunctional

URI1

Face	Range	Light	Width	Height	Columns/Rows	Factor	Led Color	Gain	Gamma	Overlapping
M	1310	5.24%	1.67mm	3.04mm	2/5	2	Blue	1.47	1.61	10 %

Sp479_11

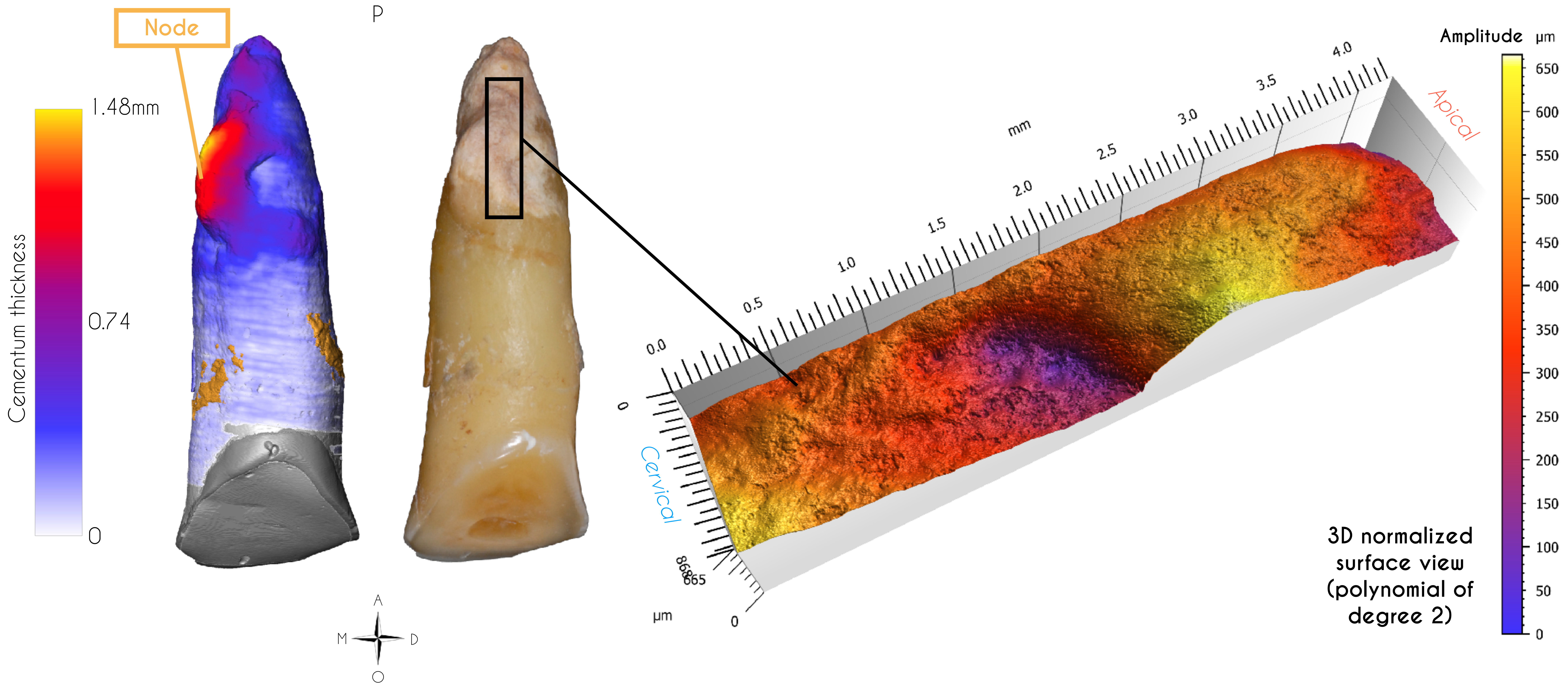

Confocal microscopy - Topography 3D maps			
LOC MAX	MAX VE (µm)	ST	Note
1 m>	665	+S2	NOD

LOC MAX: location of maximum thickness (1: apical root third; 2: middle root third; 3: cervical root third; m: mesial; d: distal; <: buccal; >: lingual);
MAX VE: maximum vertical elevation;
ST: surface texture; : scored (+) if it is high, (-) if it is low; **S:** smooth or **R:** rough; the frequency of occurrence of the elevations scored (1) if the frequency is high, (2) if the frequency is low.
NOD: node

Hyperfunctional

Sp591_12

Tooth	Occlusal Wear			CAR Si/Sta	Pulp EXP	IMP	Hypercementosis	Bone Context			
	DEG	DIR	FOR					FEN	CAL	NT	ANTA
**URI2**	**5**	**4**	**6**	**-**	**0**	**0**	**1.1.m**	**0**	**0**	**1d**	**1**

URI2

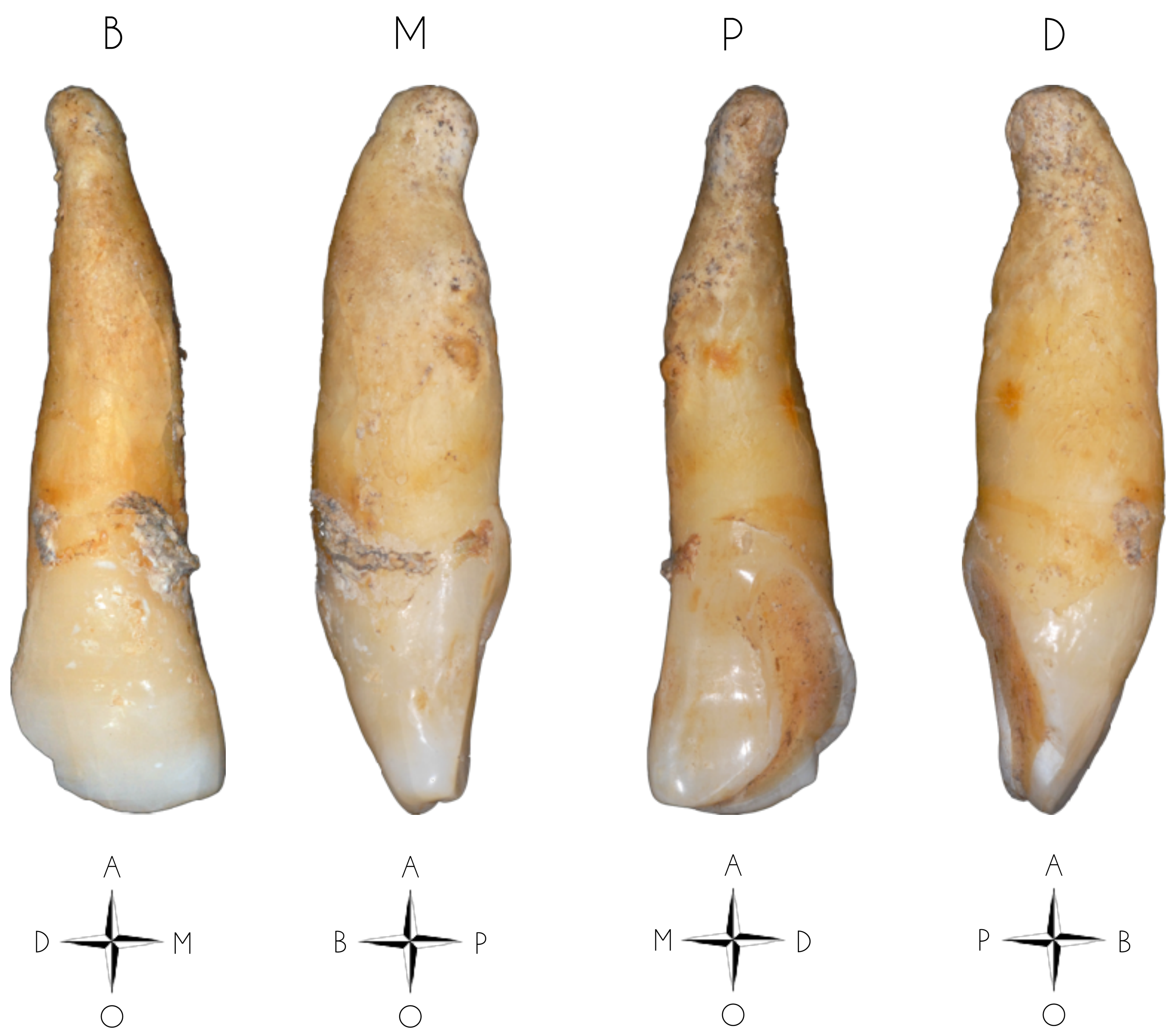

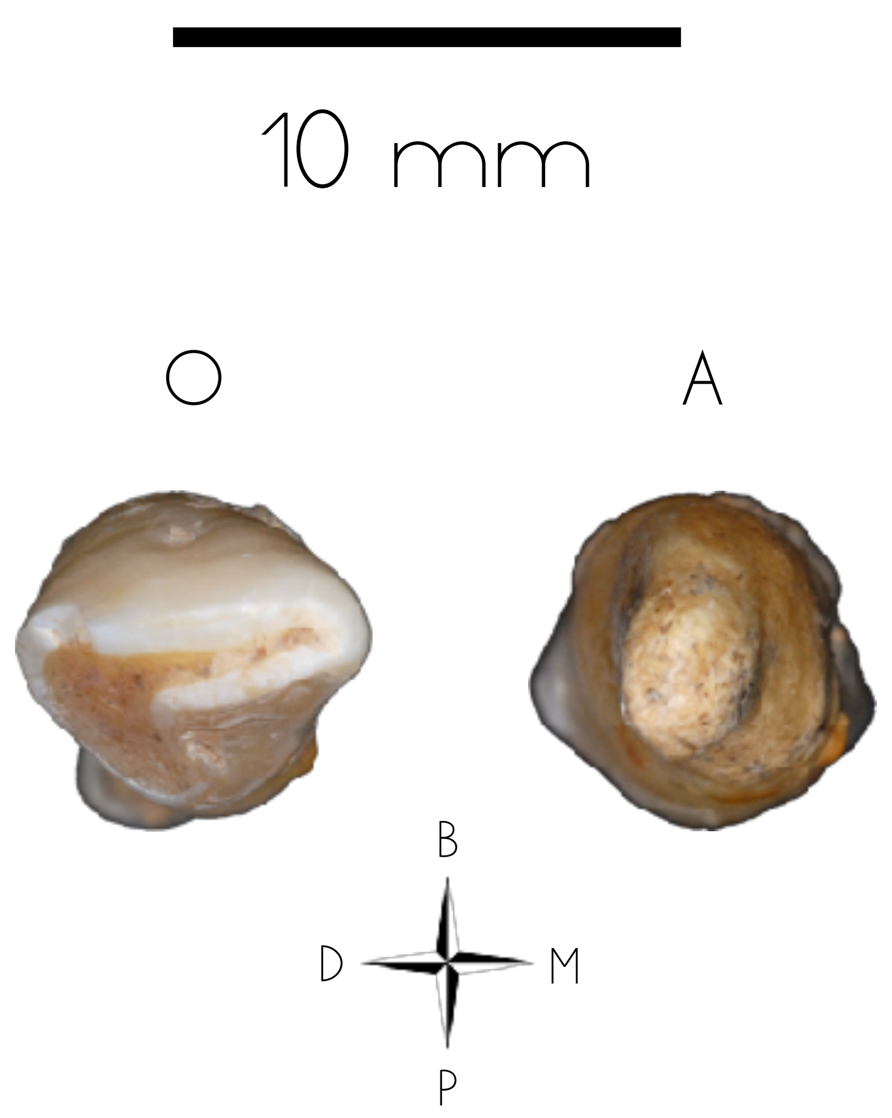

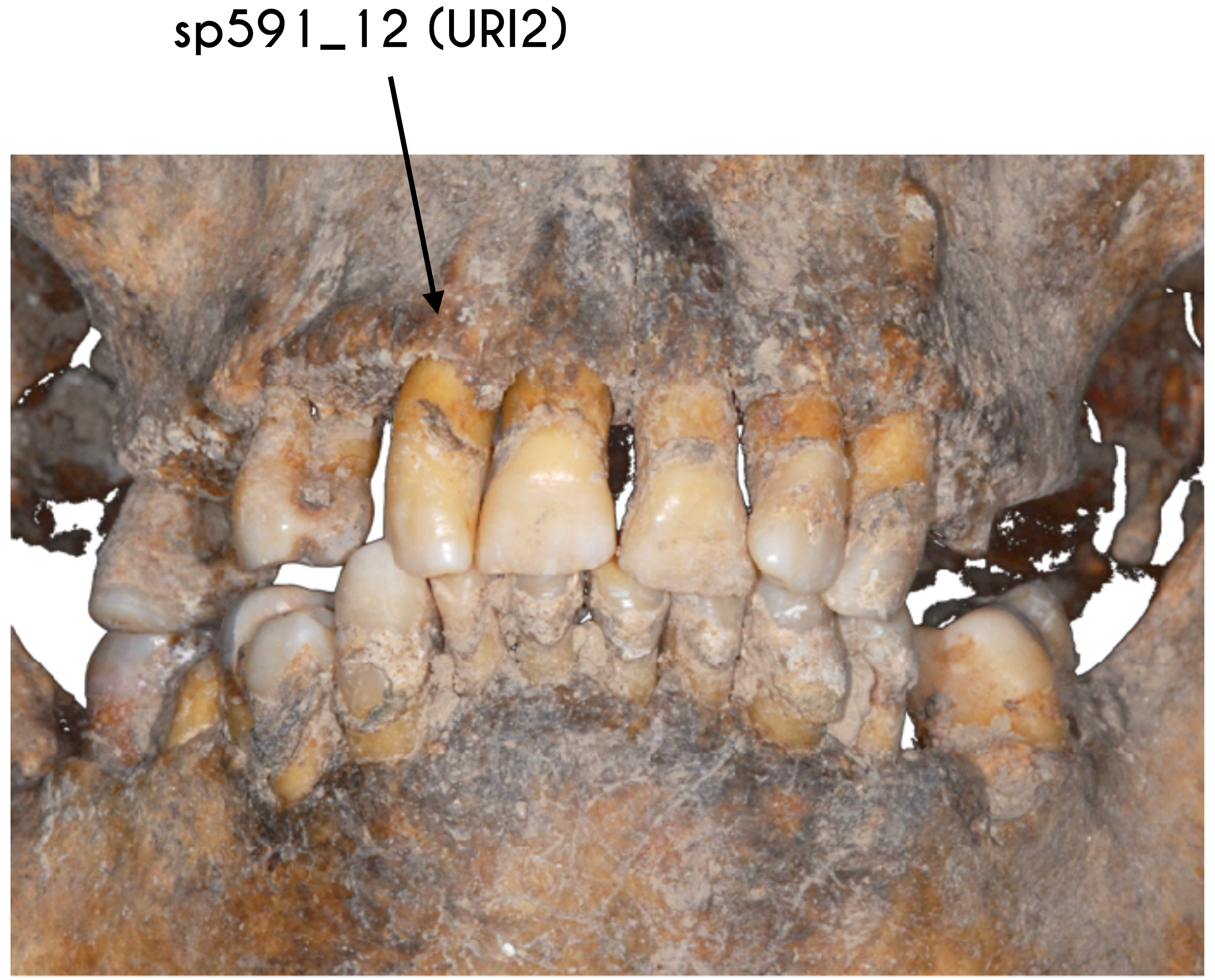

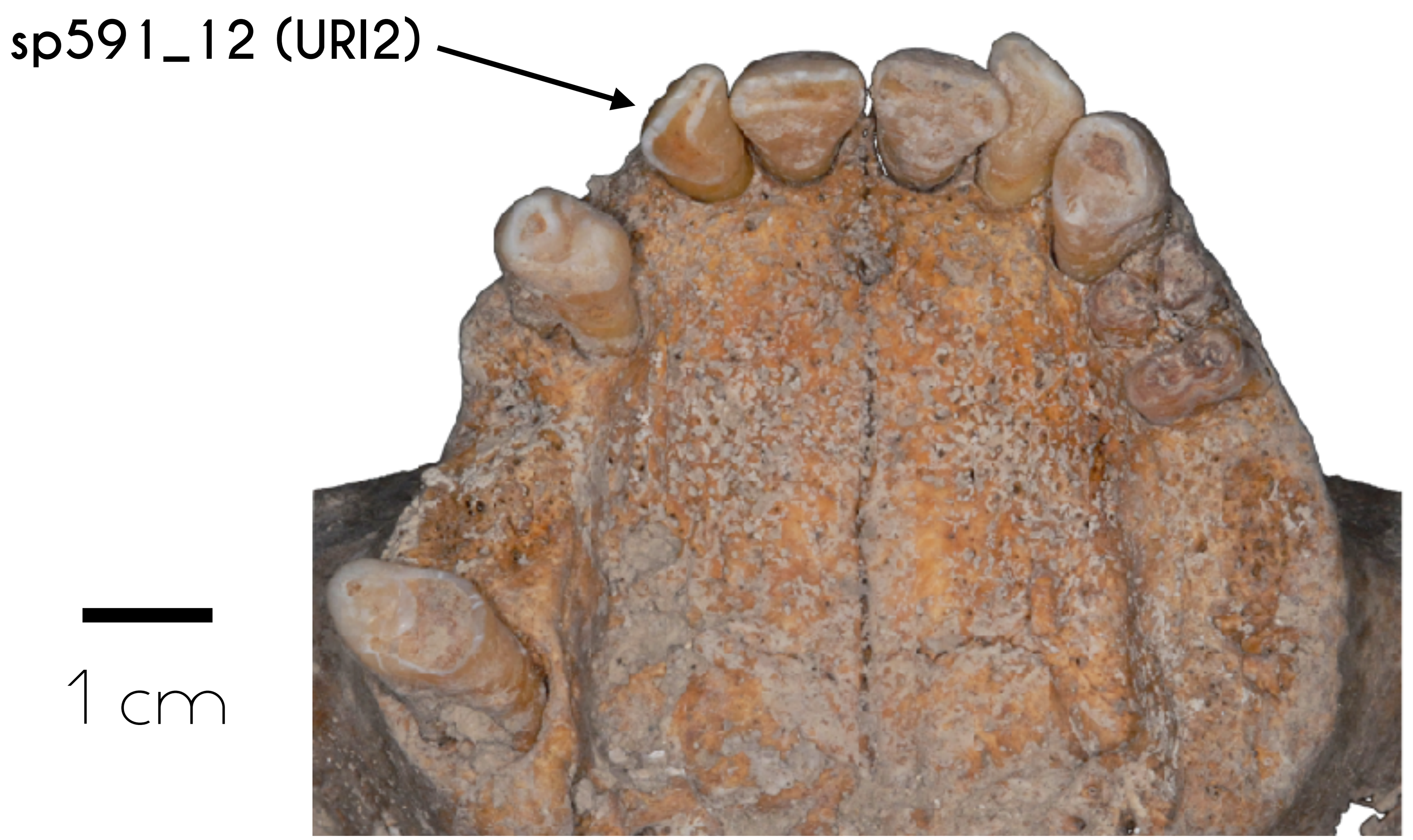

DEG: degree; **DIR:** direction; **FOR:** form - **CAR**: carious lesion - **Pulp EXP:** pulp exposure - **IMP**: impaction - **FEN:** fenestration; **CAL:** calculus; **NT:** tooth loss in neighbouring teeth; **ANTA:** antagonist

Sp591_12

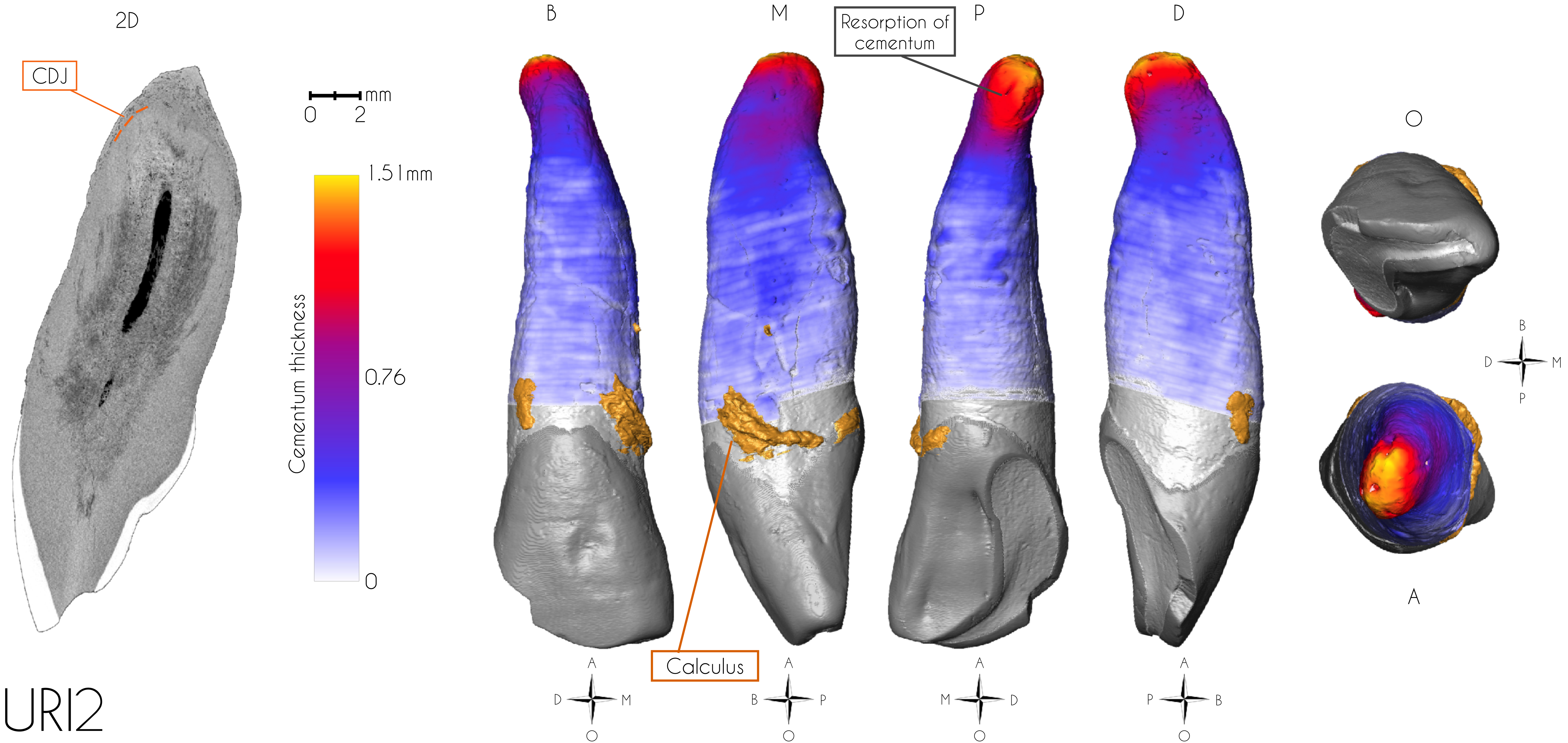

Microtomography - Thickness 3D maps				
MAX THI (μm)	LOC MAX	LOC MIN	PREF LOC	Note
1510	1>	<	Yes	FR (1>)

MAX THI: maximum thickness of cementum;
LOC MAX: location of maximum thickness;
LOC MIN: location of minimum thickness;
PREF LOC: preferential location;
FR: fracture of cementum;
(1: apical root third; 2: middle root third; 3: cervical root third; m: mesial; d: distal; <: buccal; >: lingual)

Hyperfunctional

URI2

Face	Range	Light	Width	Height	Columns/Rows	Factor	Led Color	Gain	Gamma	Overlapping
PD	804	6.02%	1.67mm	1.25mm	2/2	2	Blue	1.47	1.61	10 %

Sp591_12

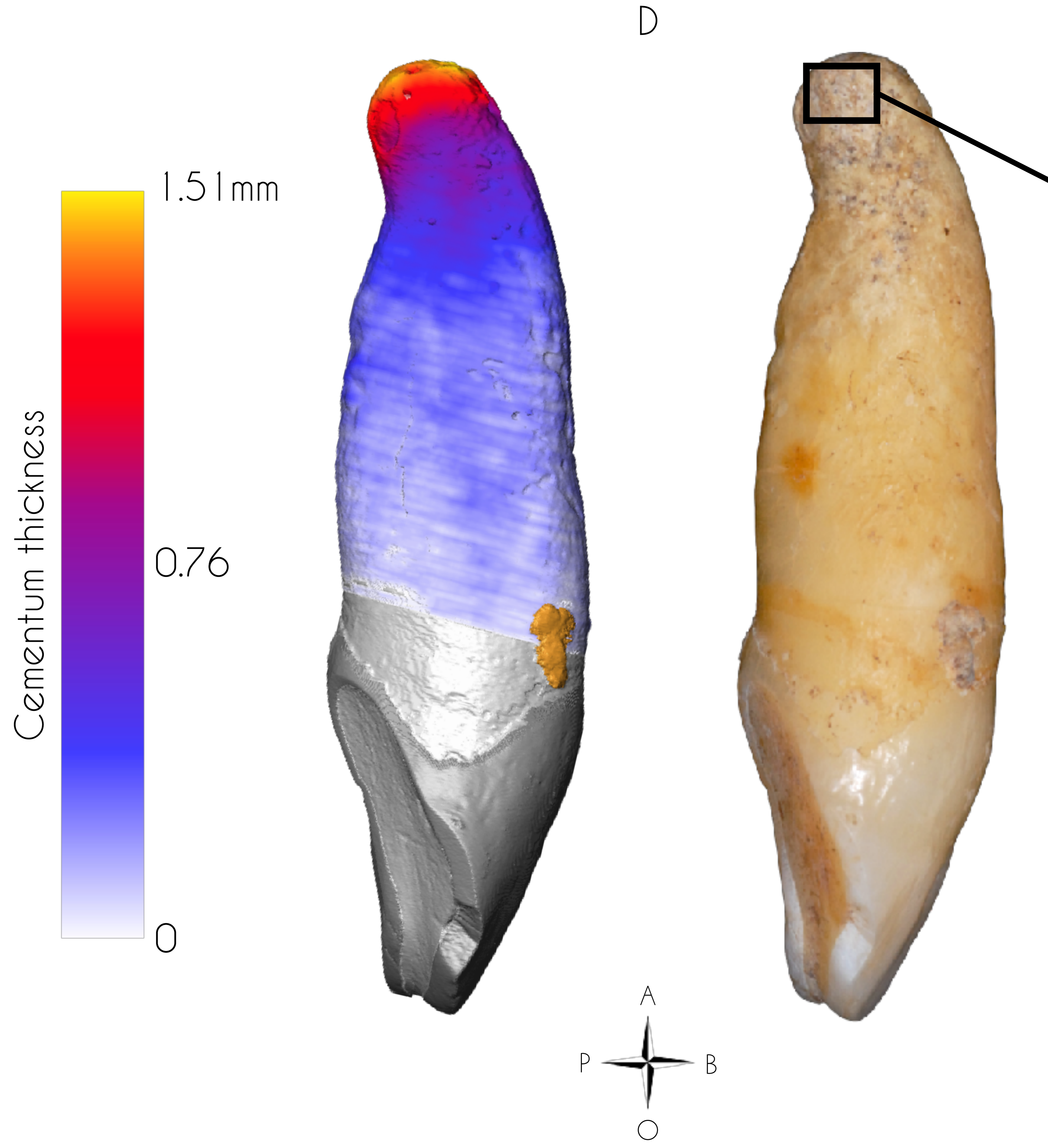

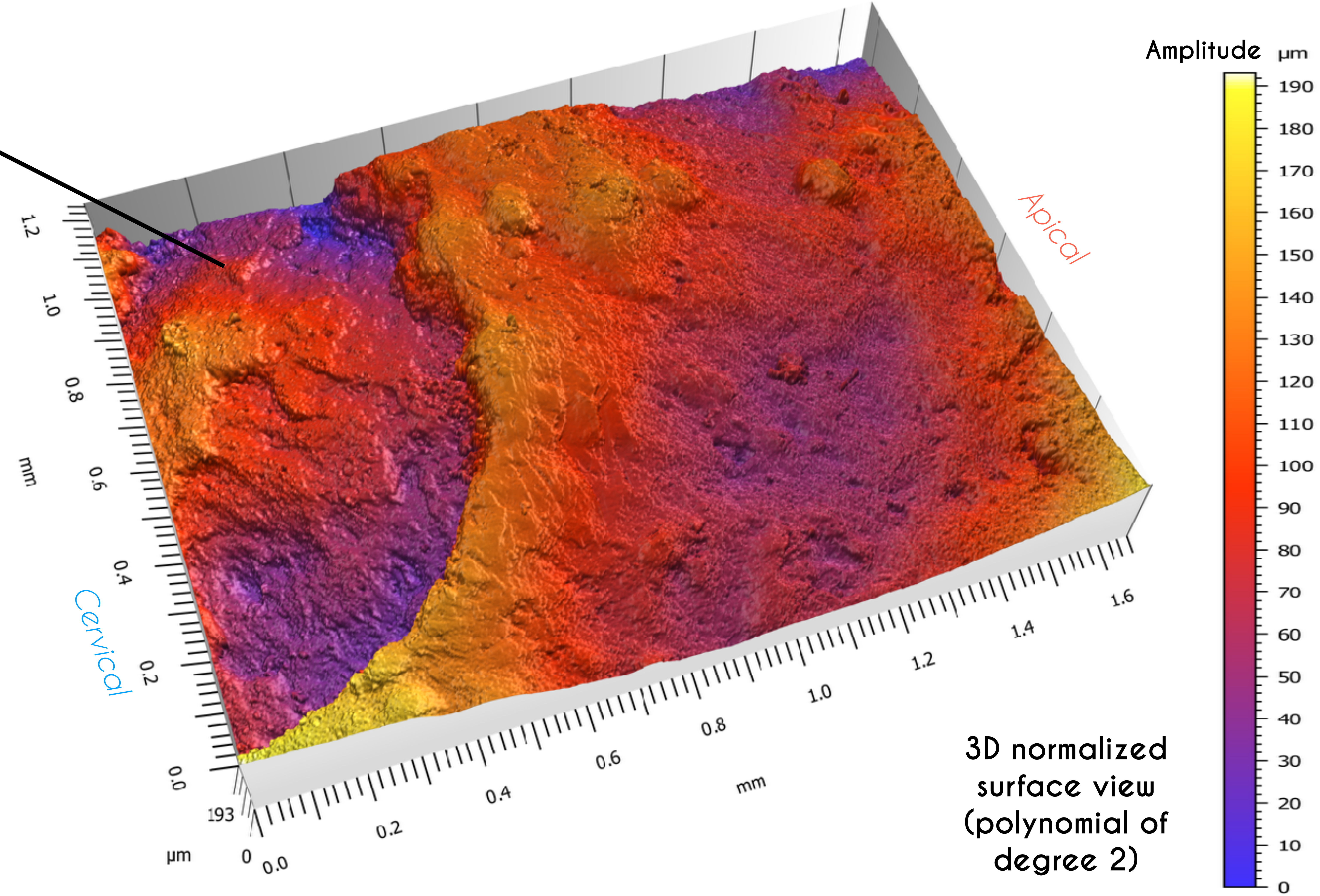

Confocal microscopy - Topography 3D maps			
LOC MAX	MAX VE (µm)	ST	Note
1d	192	-R2	FR

Atypical SA: fracture of cementum

LOC MAX: location of maximum thickness (1: apical root third; 2: middle root third; 3: cervical root third; m: mesial; d: distal; <: buccal; >: lingual);
MAX VE: maximum vertical elevation;
ST: surface texture; : scored (**+**) if it is high, (**-**) if it is low; **S:** smooth or **R:** rough; the frequency of occurrence of the elevations scored (**1**) if the frequency is high, (**2**) if the frequency is low.
FR: fracture of cementum

Hyperfunctional

Sp810_13

Tooth	Occlusal Wear			CAR Si/Sta	Pulp EXP	IMP	Hypercementosis	Bone Context			
	DEG	DIR	FOR					FEN	CAL	NT	ANTA
**URC**	**3**	**2**	**2**	**-**	**0**	**0**	**1.3.m**	**0**	**0**	**0**	**NA**

URC

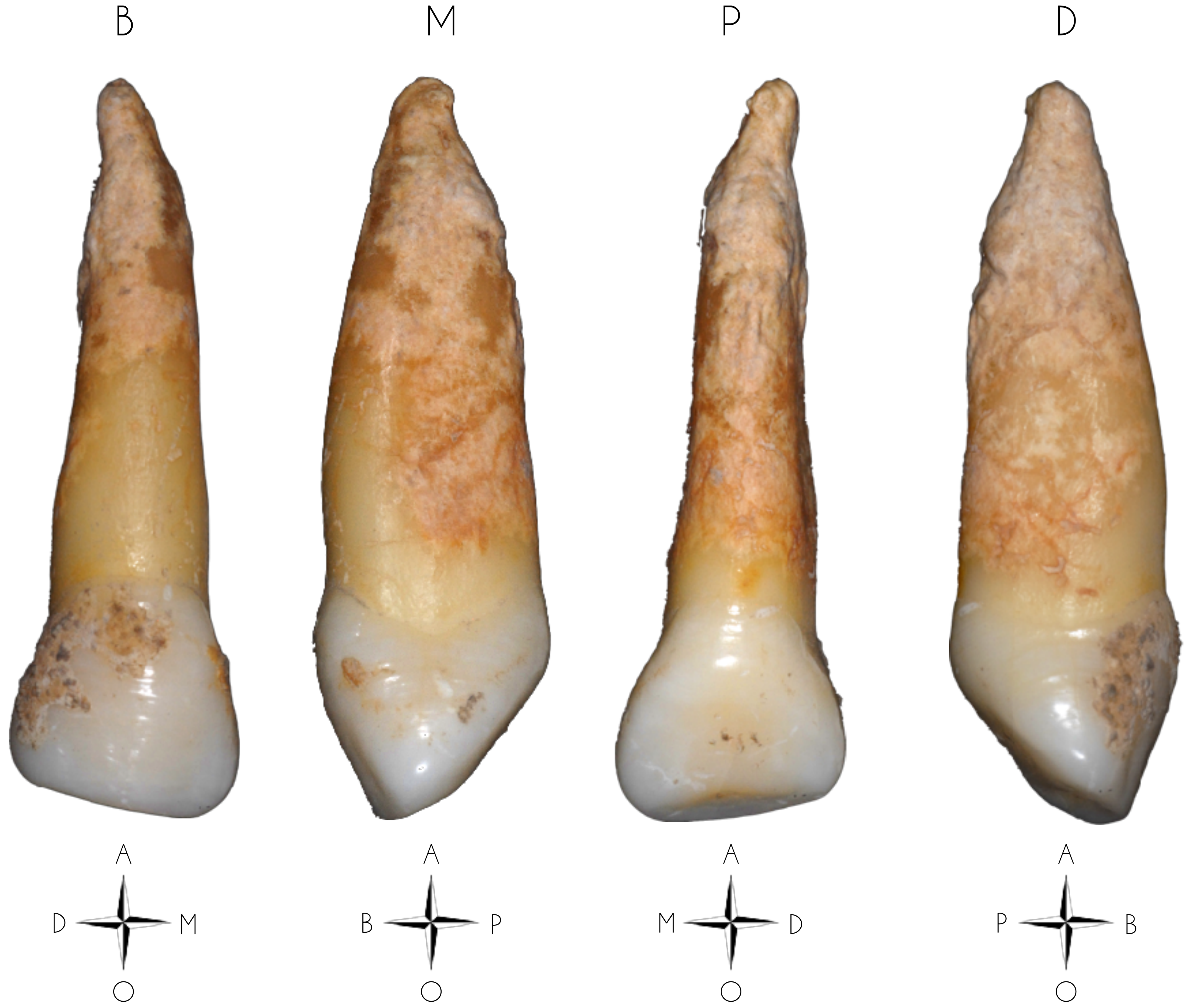

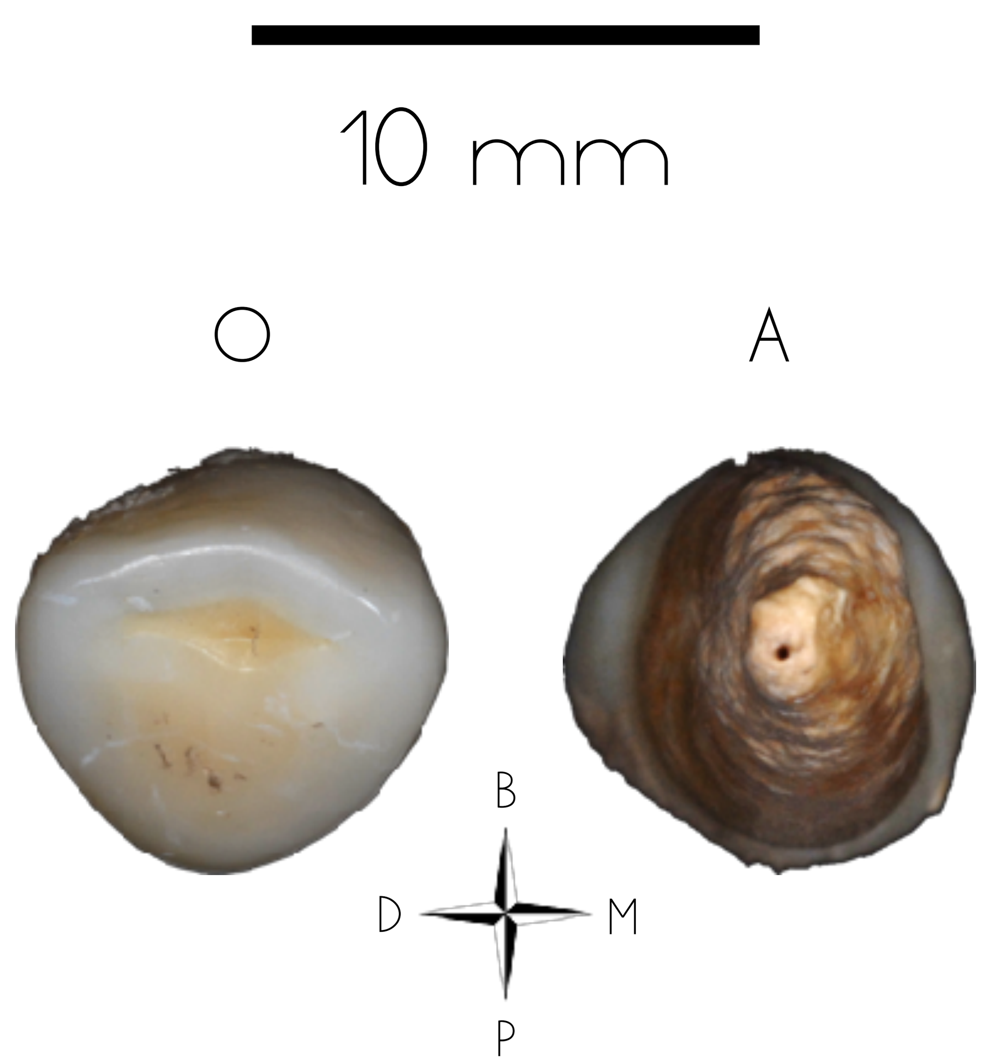

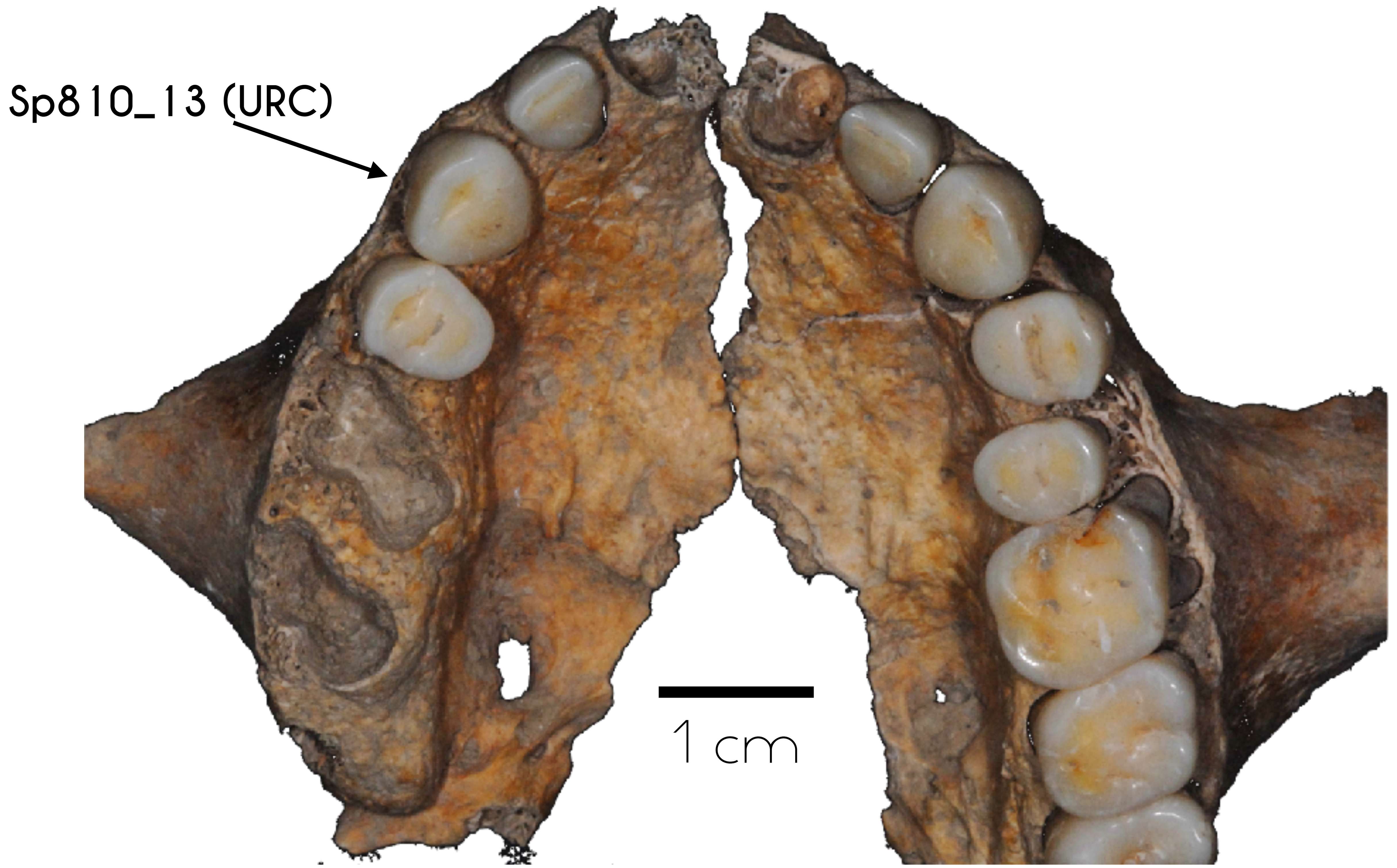

DEG: degree; **DIR:** direction; **FOR:** form - **CAR**: carious lesion - **Pulp EXP:** pulp exposure - **IMP**: impaction - **FEN:** fenestration; **CAL:** calculus; **NT:** tooth loss in neighbouring teeth; **ANTA:** antagonist

Sp810_13

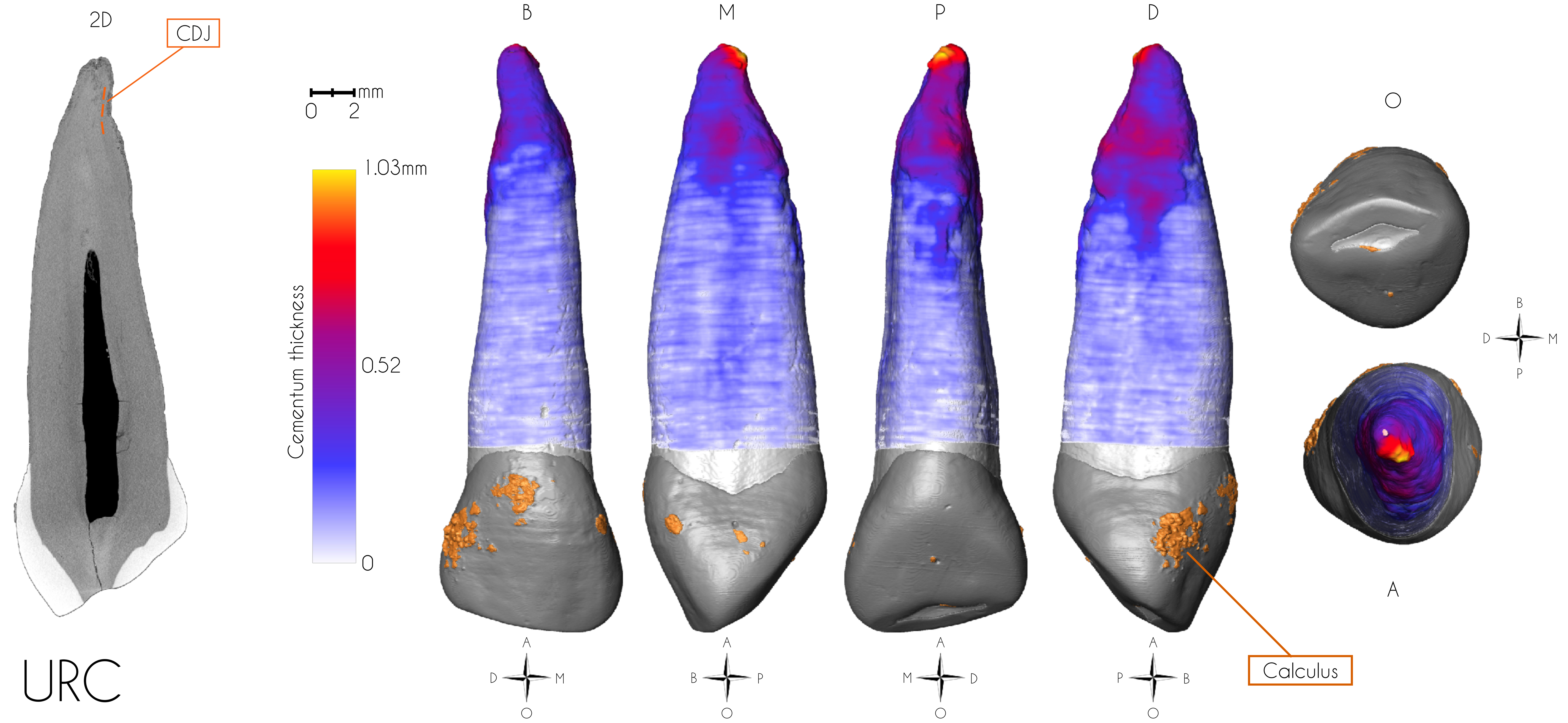

Microtomography - Thickness 3D maps				
MAX THI (μm)	LOC MAX	LOC MIN	PREF LOC	Note
1030	1>	<	Yes	-

MAX THI: maximum thickness of cementum;
LOC MAX: location of maximum thickness;
LOC MIN: location of minimum thickness;
(1: apical root third; 2: middle root third; 3: cervical root third; m: mesial; d: distal; <: buccal; >: lingual)
PREF LOC: preferential location;

Hyperfunctional

URC

Face	Range	Light	Width	Height	Columns/Rows	Factor	Led Color	Gain	Gamma	Overlapping
P	721	6.02%	0.88mm	1.25mm	1/2	2	Blue	1.5	1.8	10 %

Sp810_13

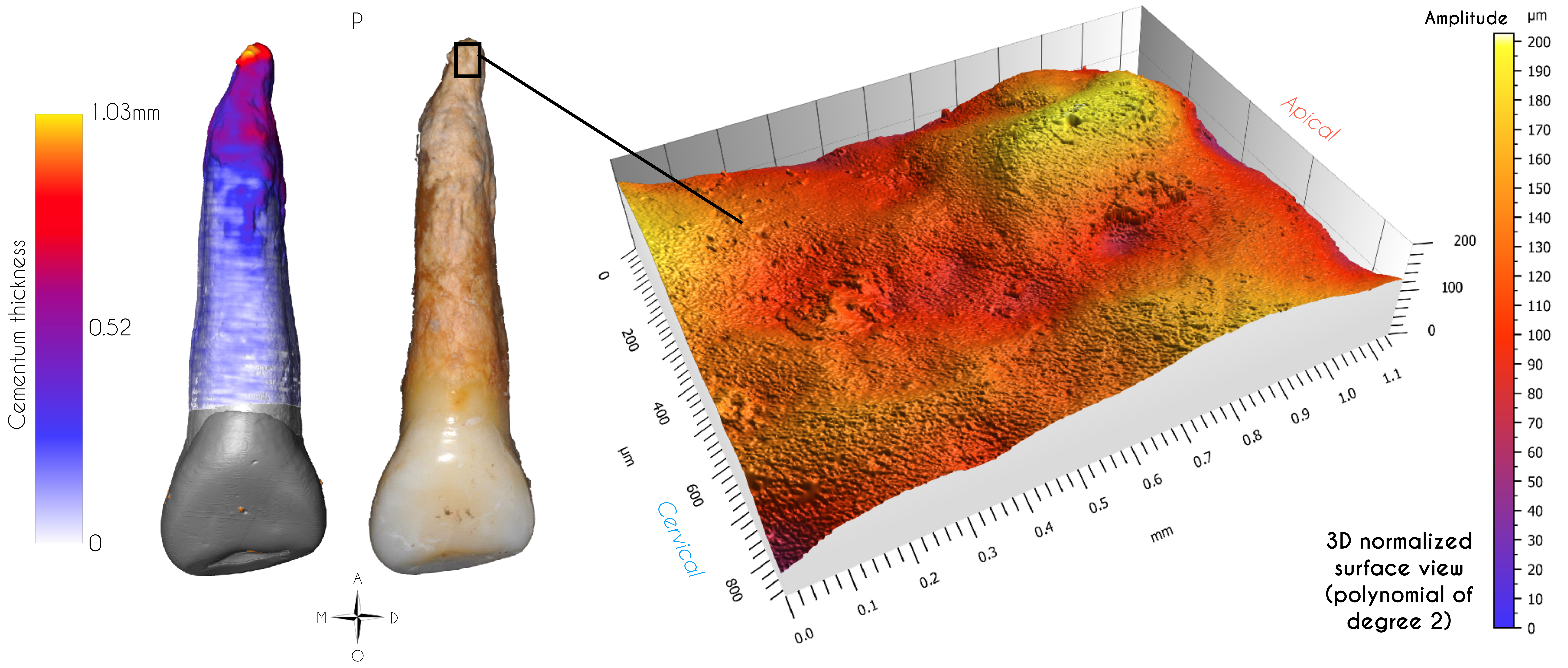

Confocal microscopy - Topography 3D maps			
LOC MAX	MAX VE (µm)	ST	Note
1>	200	-R1	-

LOC MAX: location of maximum thickness (1: apical root third; 2: middle root third; 3: cervical root third; m: mesial; d: distal; <: buccal; >: lingual);
MAX VE: maximum vertical elevation;
ST: surface texture; : scored **(+)** if it is high, **(-)** if it is low; **S:** smooth or **R:** rough; the frequency of occurrence of the elevations scored **(1)** if the frequency is high, **(2)** if the frequency is low.

Hyperfunctional

Sp810_23

Tooth	Occlusal Wear			CAR Si/Sta	Pulp EXP	IMP	Hypercementosis	Bone Context			
	DEG	DIR	FOR					FEN	CAL	NT	ANTA
**ULC**	3	2	2	-	0	0	3.2.m	0	0	0	NA

ULC

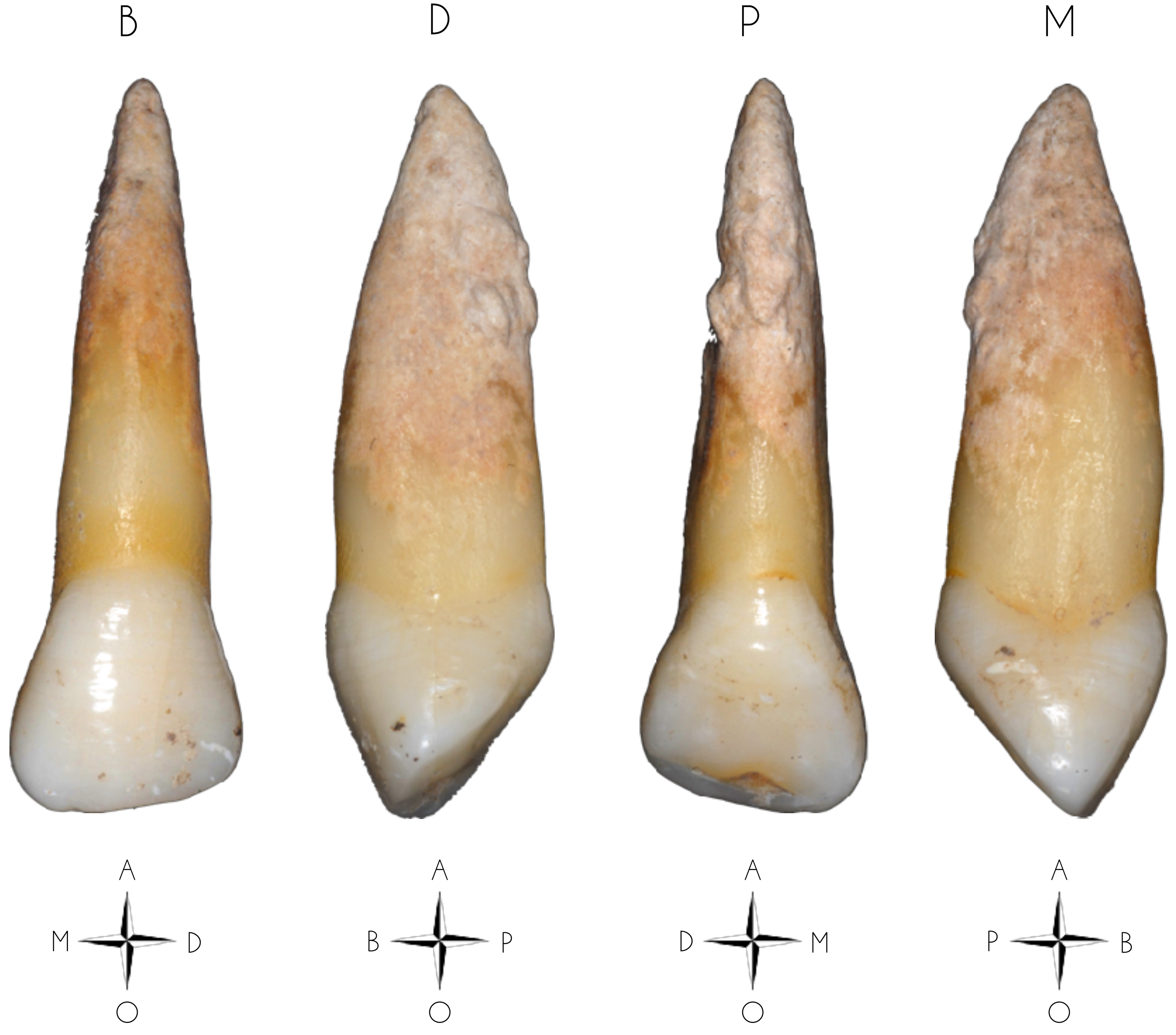

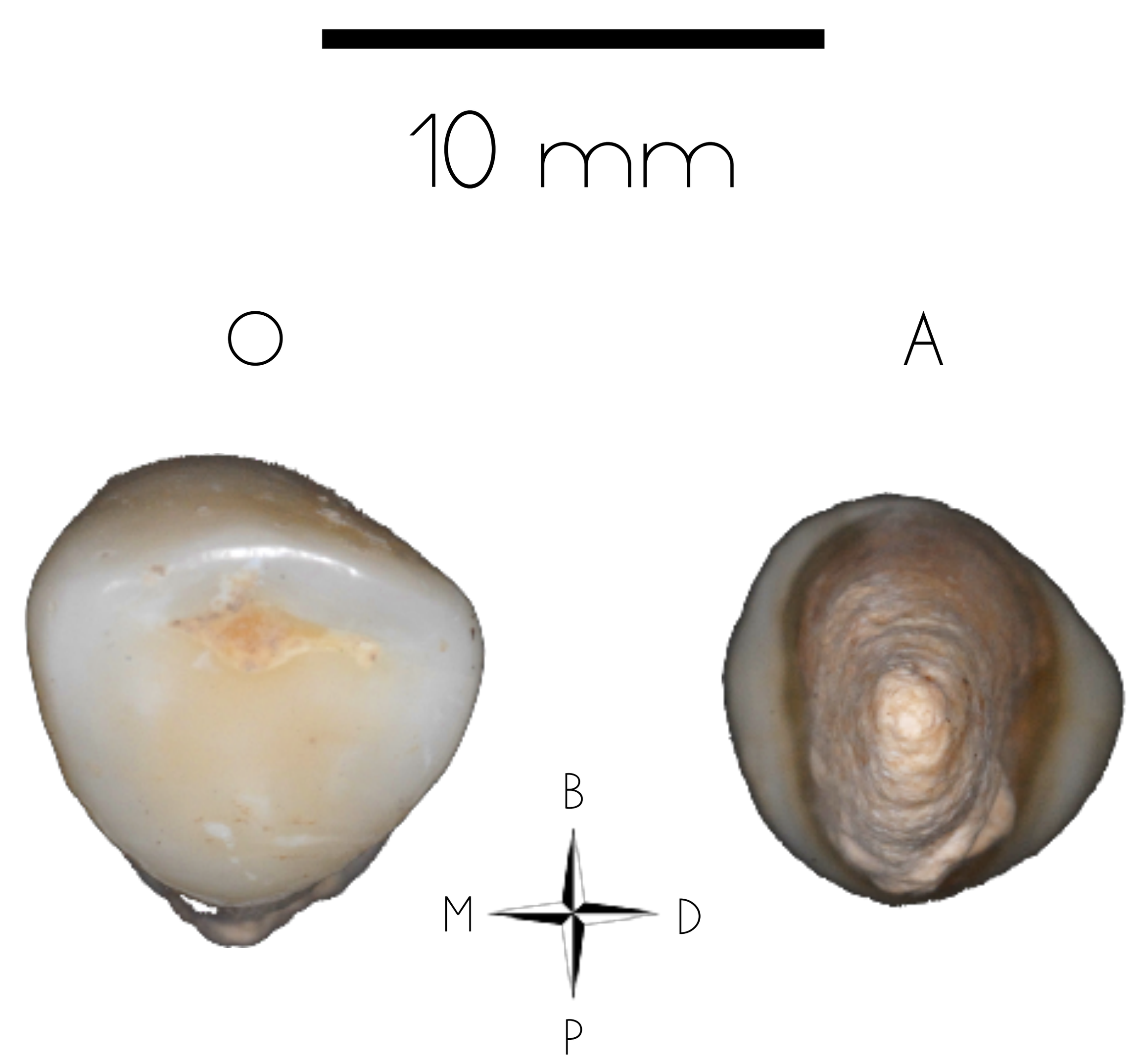

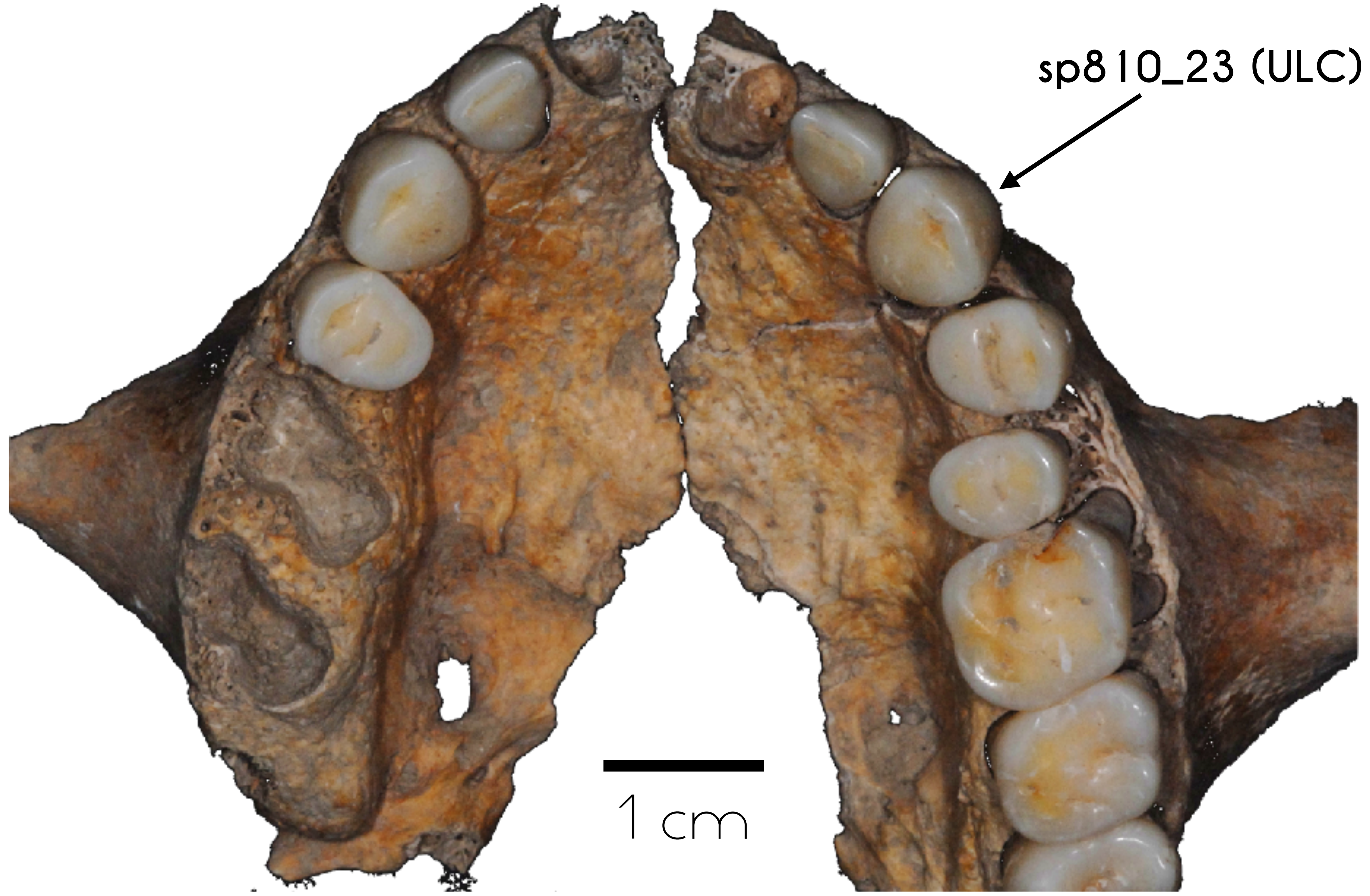

DEG: degree; **DIR:** direction; **FOR:** form - **CAR**: carious lesion - **Pulp EXP:** pulp exposure - **IMP**: impaction - **FEN:** fenestration; **CAL:** calculus; **NT:** tooth loss in neighbouring teeth; **ANTA:** antagonist

Sp810_23

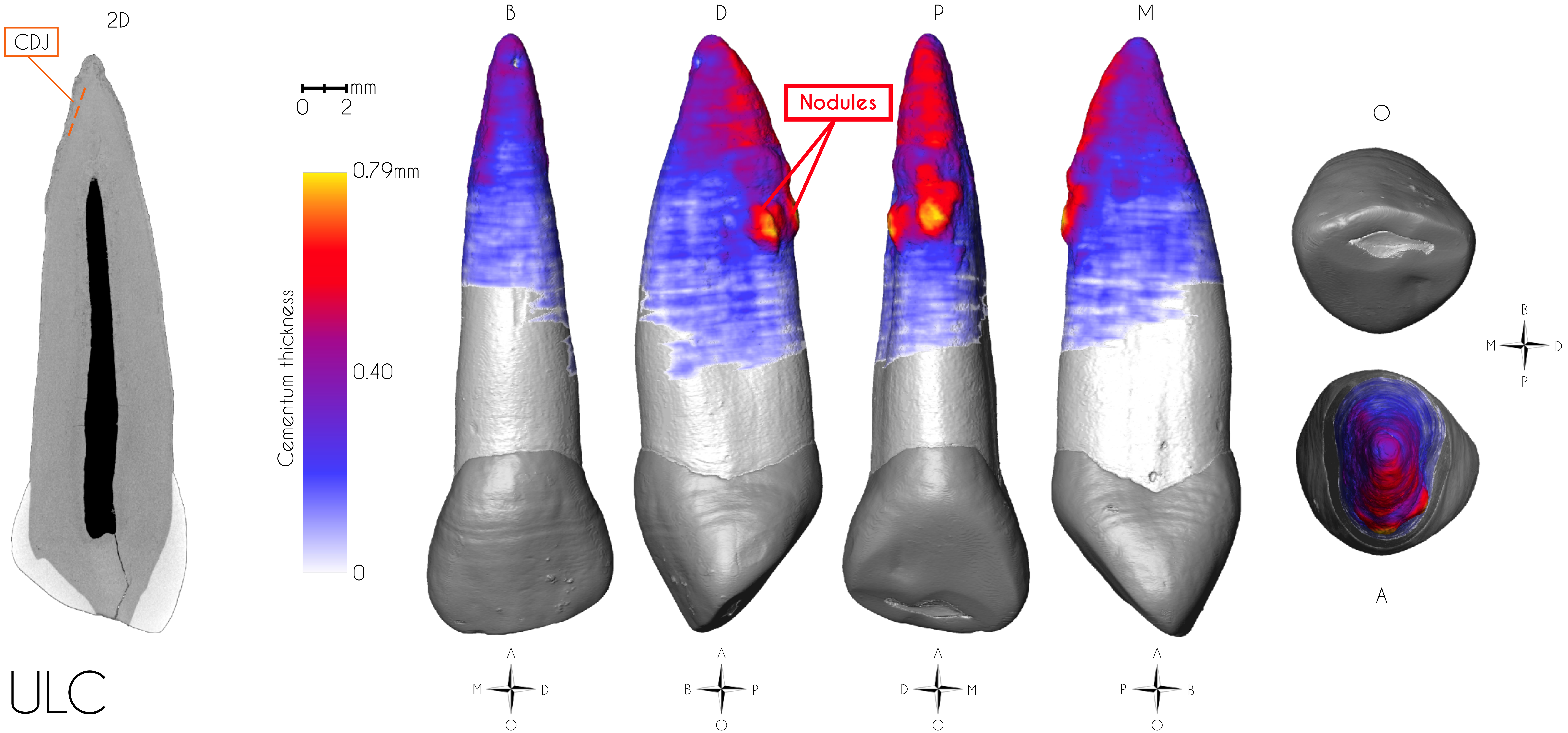

Microtomography - Thickness 3D maps				
MAX THI (µm)	LOC MAX	LOC MIN	PREF LOC	Note
790	2>	<	Yes	Nds (>)

MAX THI: maximum thickness of cementum;
LOC MAX: location of maximum thickness;
LOC MIN: location of minimum thickness;
PREF LOC: preferential location;
Nds: nodules;
(1: apical root third; 2: middle root third; 3: cervical root third; m: mesial; d: distal; <: buccal; >: lingual)

<u>Hyperfunctional</u>

ULC

Face	Range	Light	Width	Height	Columns/Rows	Factor	Led Color	Gain	Gamma	Overlapping
P	1788	5.50%	0.88mm	8.98mm	1/15	2	Blue	1.5	1.8	10 %

Sp810_23

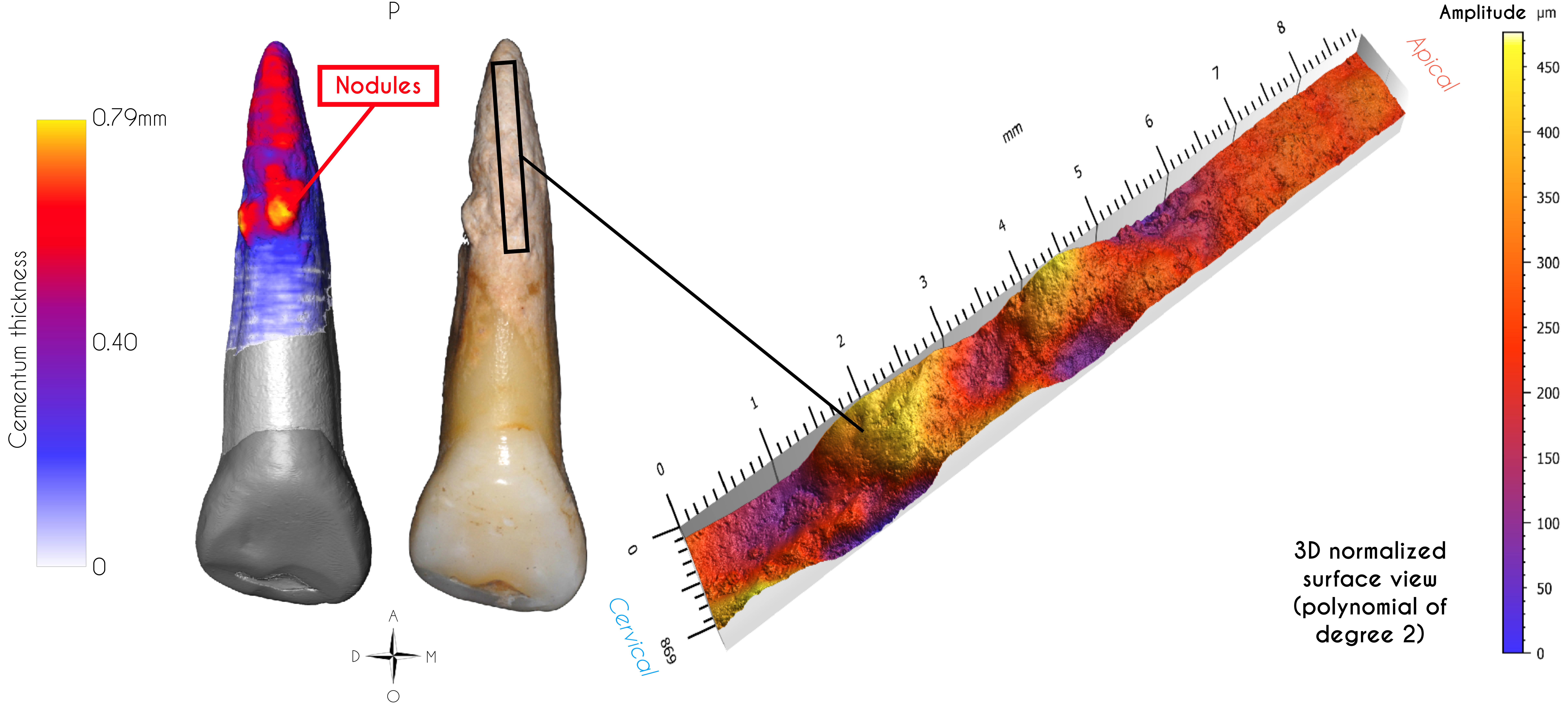

Confocal microscopy - Topography 3D maps			
LOC MAX	MAX VE (µm)	ST	Note
2>	475	+S2	Nds

LOC MAX: location of maximum thickness (1: apical root third; 2: middle root third; 3: cervical root third; m: mesial; d: distal; <: buccal; >: lingual);
MAX VE: maximum vertical elevation;
ST: surface texture; : scored (**+**) if it is high, (**-**) if it is low; **S:** smooth or **R:** rough; the frequency of occurrence of the elevations scored (**1**) if the frequency is high, (**2**) if the frequency is low.
Nds: nodules

Hyperfunctional

Sp876_35

Tooth	Occlusal Wear			CAR Si/Sta	Pulp EXP	IMP	Hypercementosis	Bone Context			
	DEG	DIR	FOR					FEN	CAL	NT	ANTA
**LLP4**	6	2	6	-	0	0	1.2.m	0	1	2	2

LLP4

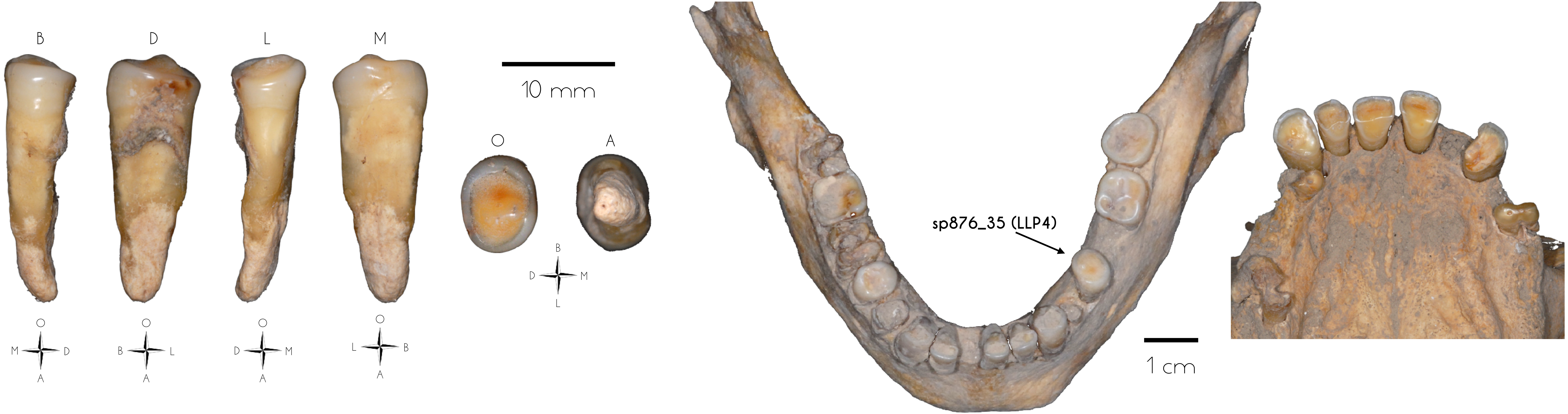

DEG: degree; **DIR:** direction; **FOR:** form - **CAR**: carious lesion - **Pulp EXP:** pulp exposure - **IMP**: impaction - **FEN:** fenestration; **CAL:** calculus; **NT:** tooth loss in neighbouring teeth; **ANTA:** antagonist

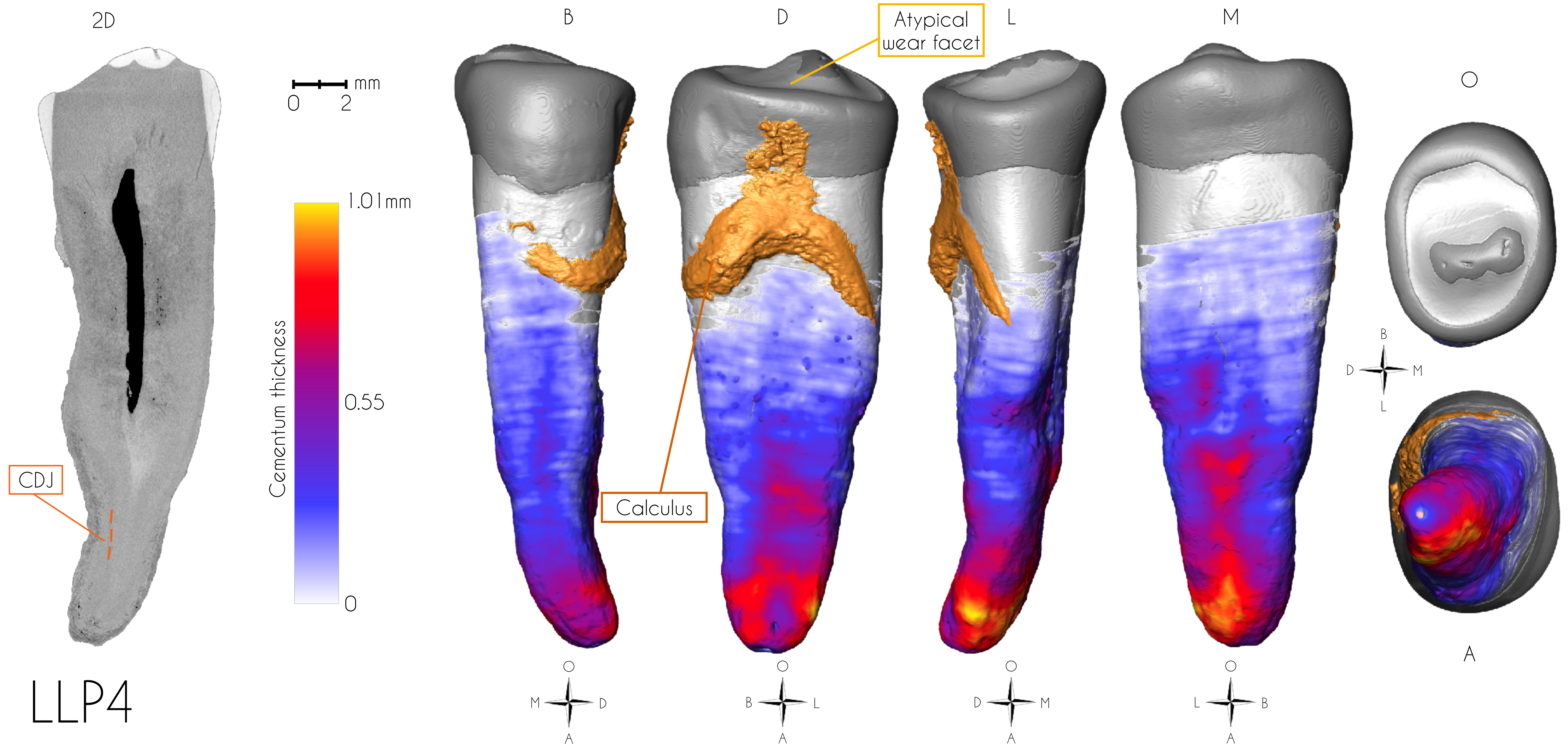

Sp876_35

Microtomography - Thickness 3D maps				
MAX THI (µm)	LOC MAX	LOC MIN	PREF LOC	Note
1010	1>	<	Yes	-

MAX THI: maximum thickness of cementum;
LOC MAX: location of maximum thickness;
LOC MIN: location of minimum thickness;
(1: apical root third; 2: middle root third; 3: cervical root third; m: mesial; d: distal; <: buccal; >: lingual)
PREF LOC: preferential location;

Hyperfunctional

LLP4

Face	Range	Light	Width	Height	Columns/Rows	Factor	Led Color	Gain	Gamma	Overlapping
LM	1304	5.76%	1.67mm	1.85mm	2/3	2	Blue	1.47	1.61	10 %

Sp876_35

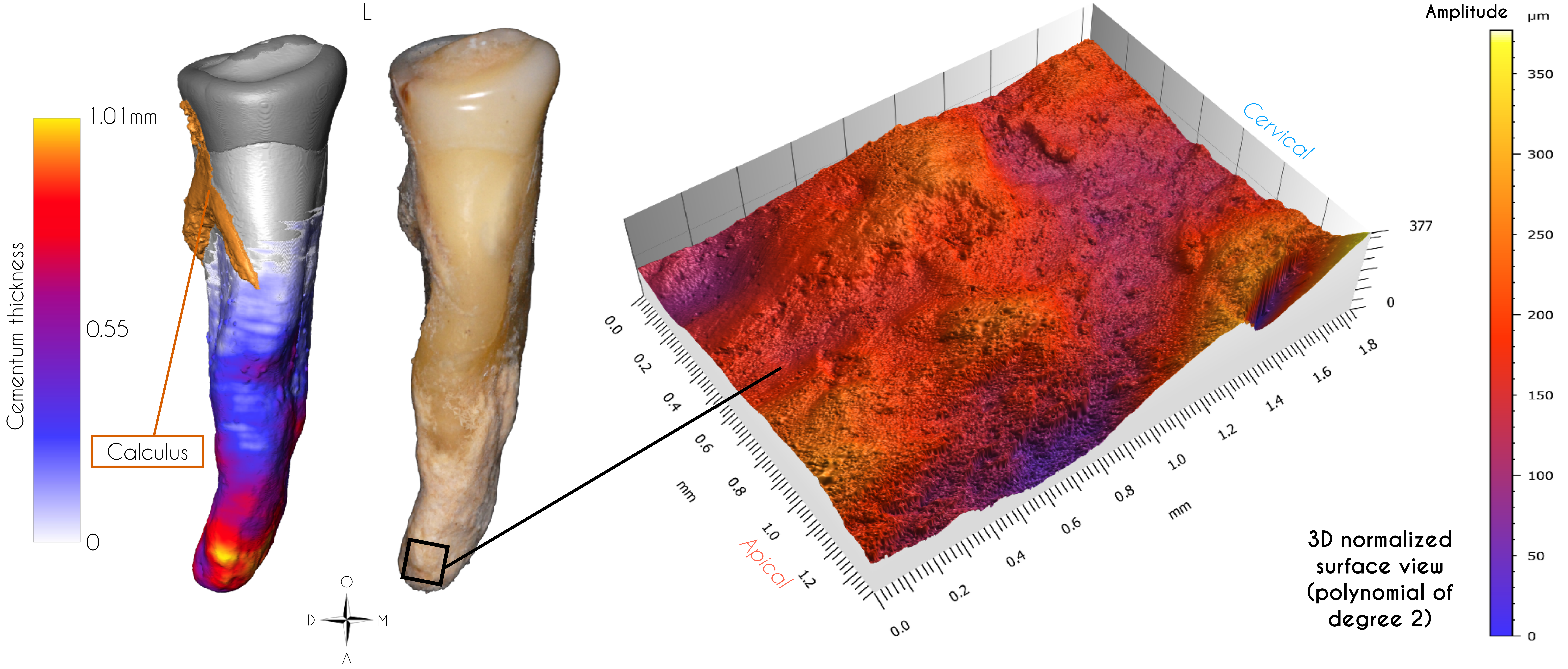

Confocal microscopy - Topography 3D maps			
LOC MAX	MAX VE (µm)	ST	Note
1>	250	+R2	-

LOC MAX: location of maximum thickness (1: apical root third; 2: middle root third; 3: cervical root third; m: mesial; d: distal; <: buccal; >: lingual);
MAX VE: maximum vertical elevation;
ST: surface texture; : scored **(+)** if it is high, **(-)** if it is low; **S:** smooth or **R:** rough; the frequency of occurrence of the elevations scored **(1)** if the frequency is high, **(2)** if the frequency is low.

Hyperfunctional

Sp914_33

Tooth	Occlusal Wear			CAR Si/Sta	Pulp EXP	IMP	Hypercementosis	Bone Context			
	DEG	DIR	FOR					FEN	CAL	NT	ANTA
**LLC**	**4**	**4**	**4**	**-**	**0**	**0**	**1.2.m**	**0**	**0**	**0**	**NA**

LLC

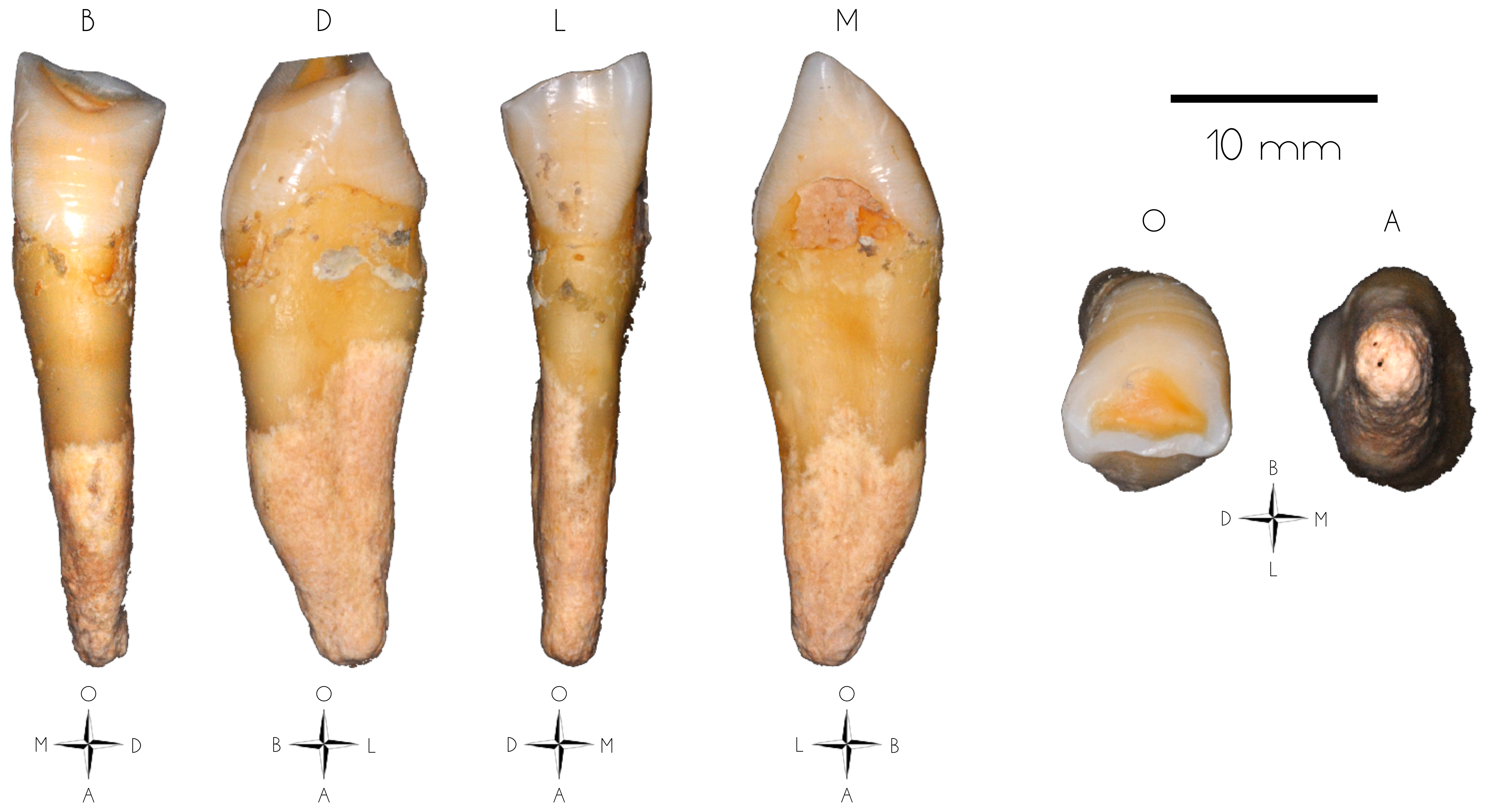

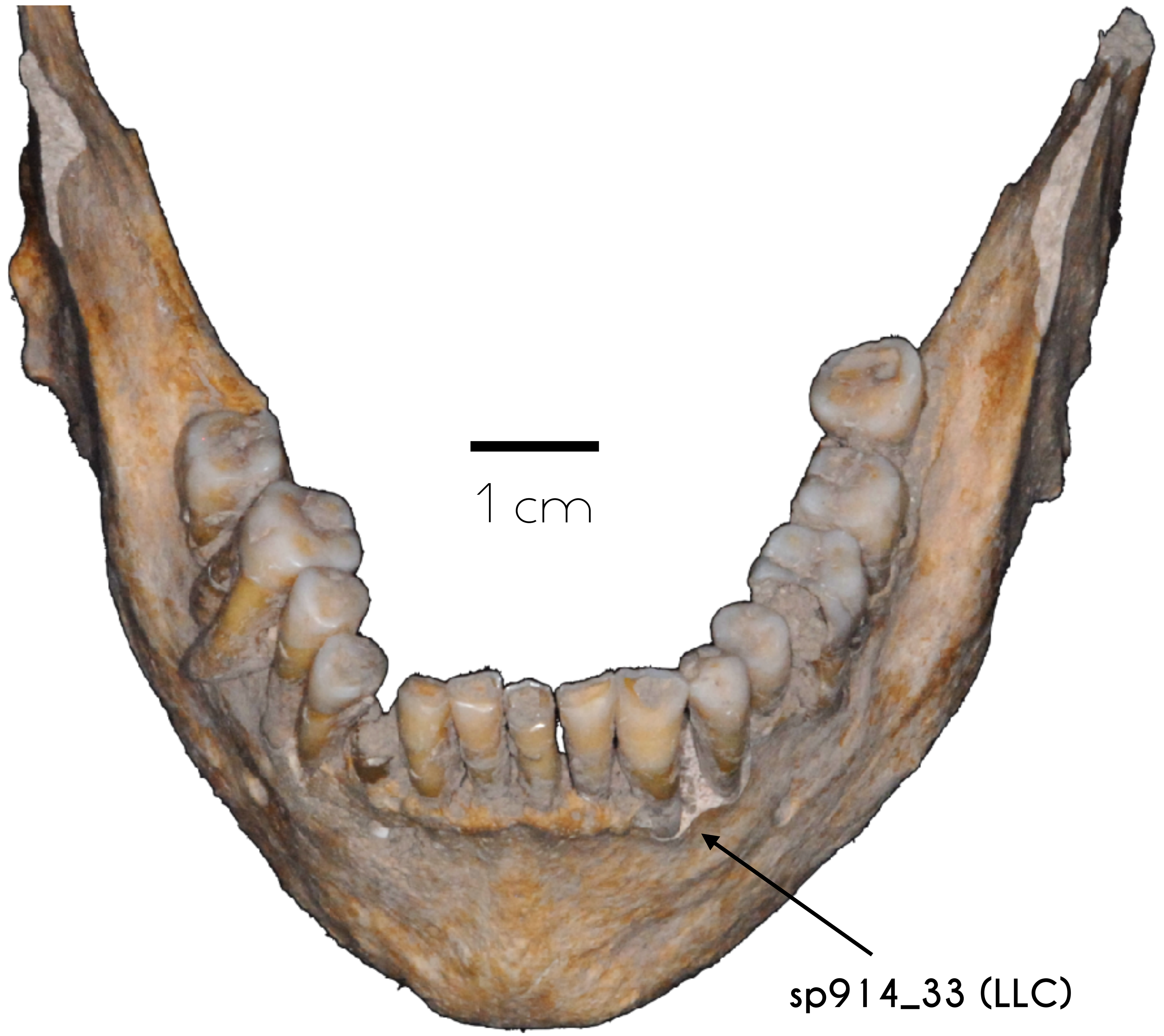

DEG: degree; **DIR:** direction; **FOR:** form - **CAR**: carious lesion - **Pulp EXP:** pulp exposure - **IMP**: impaction - **FEN:** fenestration; **CAL:** calculus; **NT:** tooth loss in neighbouring teeth; **ANTA:** antagonist

Sp914_33

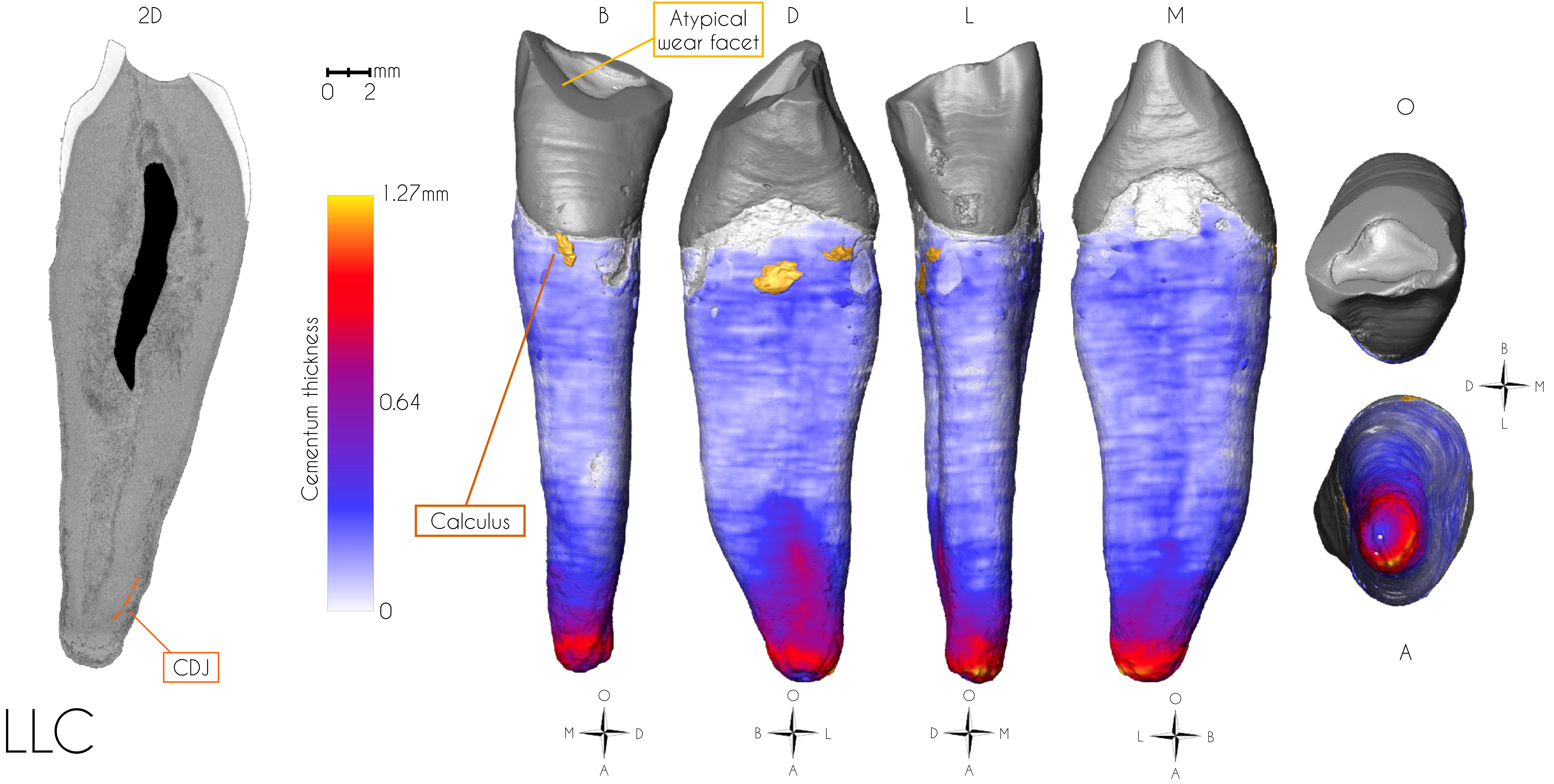

Microtomography - Thickness 3D maps				
MAX THI (µm)	LOC MAX	LOC MIN	PREF LOC	Note
1270	1>	No	Yes	-

MAX THI: maximum thickness of cementum;
LOC MAX: location of maximum thickness;
LOC MIN: location of minimum thickness;
(1: apical root third; 2: middle root third; 3: cervical root third; m: mesial; d: distal; <: buccal; >: lingual)
PREF LOC: preferential location;

Hyperfunctional

LLC

Face	Range	Light	Width	Height	Columns/Rows	Factor	Led Color	Gain	Gamma	Overlapping
LM	241	4.91%	1.67mm	1.85mm	2/3	2	Blue	1.47	1.61	10 %

Sp914_33

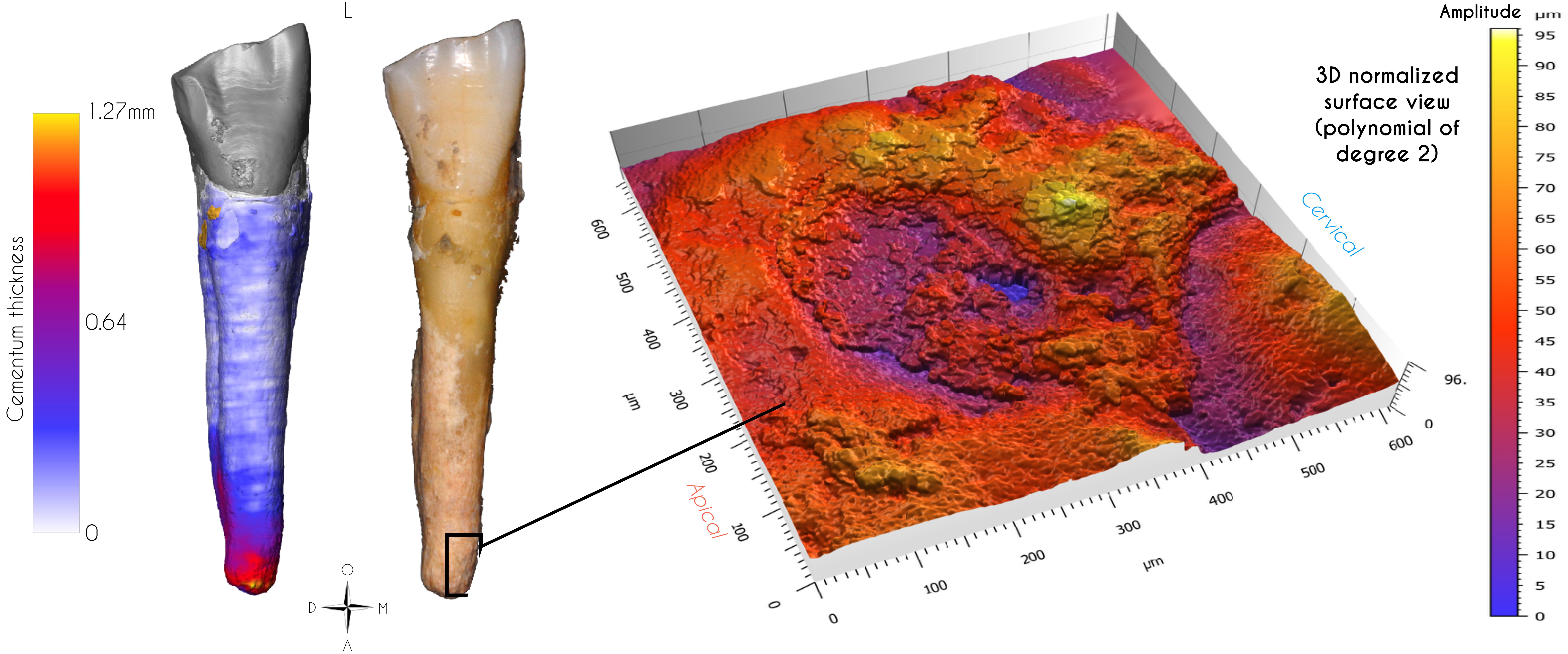

Confocal microscopy - Topography 3D maps			
LOC MAX	MAX VE (µm)	ST	Note
1>	95	+R1	-

LOC MAX: location of maximum thickness (1: apical root third; 2: middle root third; 3: cervical root third; m: mesial; d: distal; <: buccal; >: lingual);
MAX VE: maximum vertical elevation;
ST: surface texture; : scored (**+**) if it is high, (**-**) if it is low; **S:** smooth or **R:** rough; the frequency of occurrence of the elevations scored (**1**) if the frequency is high, (**2**) if the frequency is low.

Hyperfunctional

Sp1010_44

Tooth	Occlusal Wear			CAR Si/Sta	Pulp EXP	IMP	Hypercementosis	Bone Context			
	DEG	DIR	FOR					FEN	CAL	NT	ANTA
LRP3	5	4	6	-	0	0	3.2.M	0	0	1d	NA

LRP3

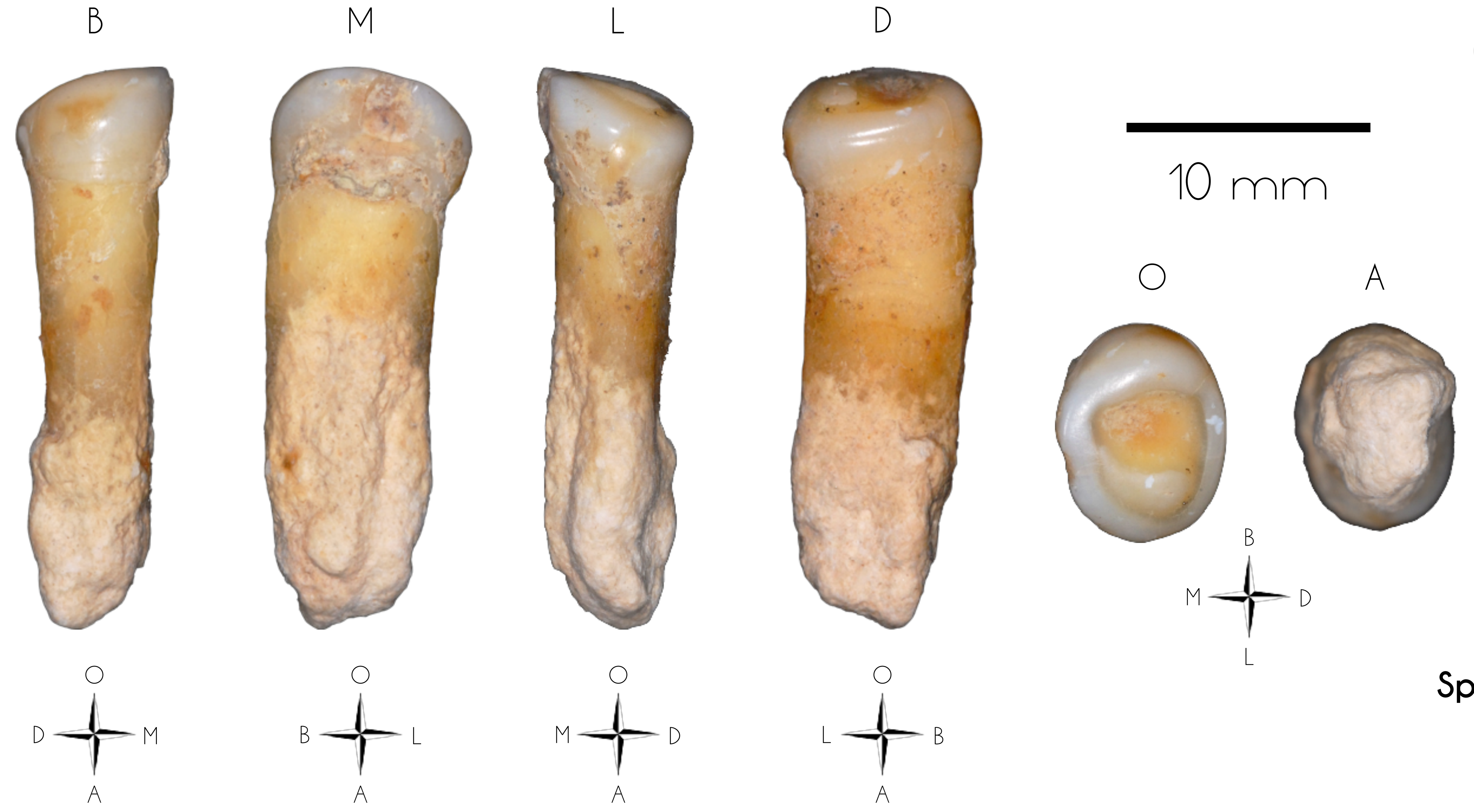

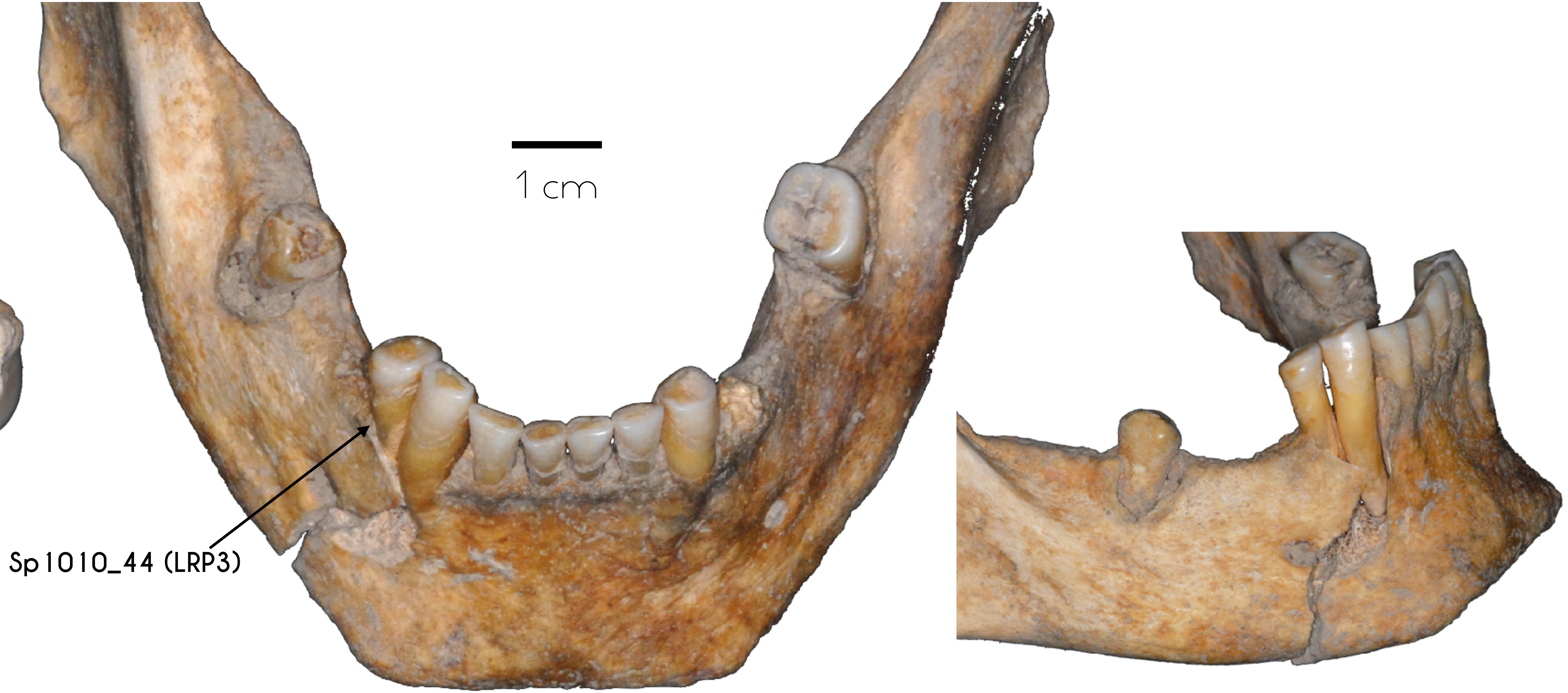

DEG: degree; **DIR:** direction; **FOR:** form - **CAR**: carious lesion - **Pulp EXP:** pulp exposure - **IMP**: impaction - **FEN:** fenestration; **CAL:** calculus; **NT:** tooth loss in neighbouring teeth; **ANTA:** antagonist

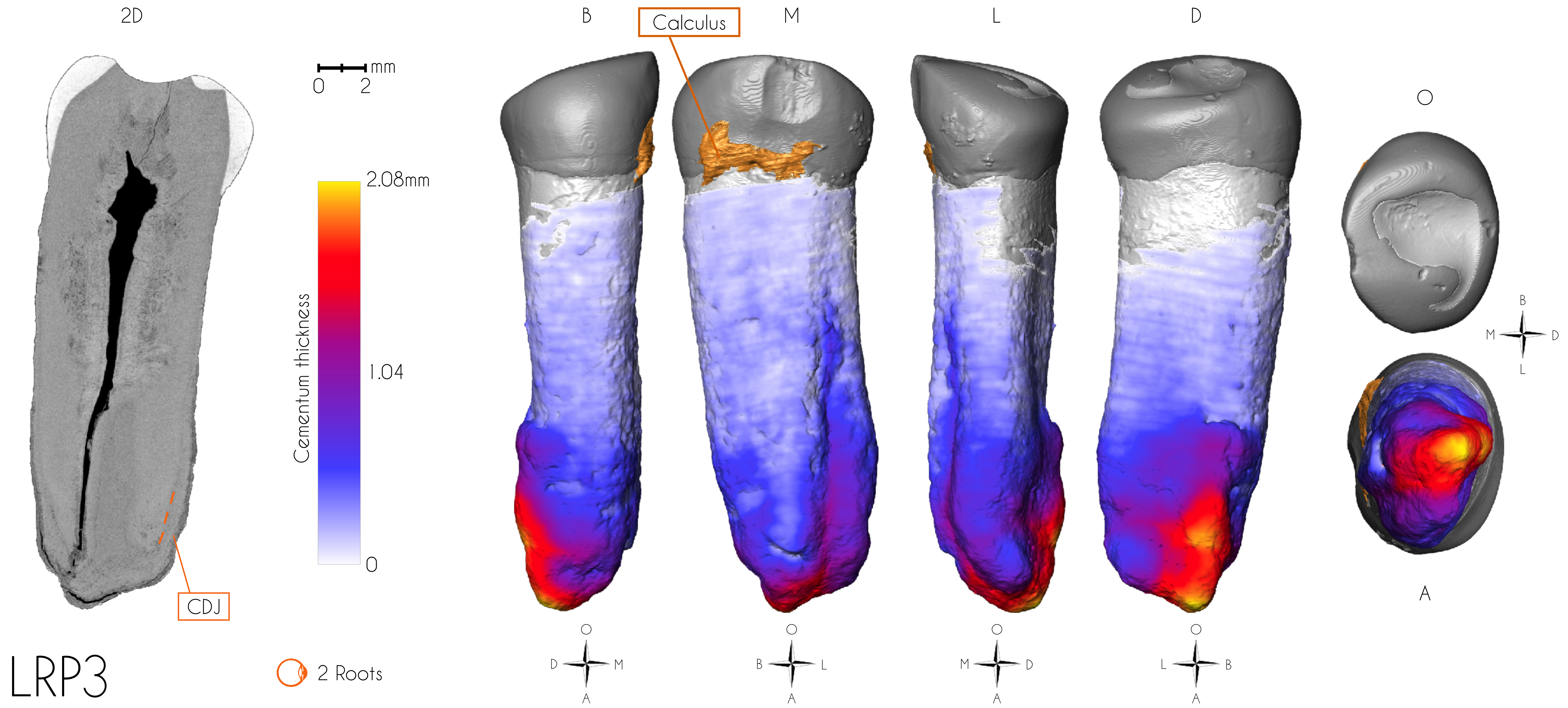

Sp1010_44

Microtomography - Thickness 3D maps				
MAX THI (μm)	LOC MAX	LOC MIN	PREF LOC	Note
2080	1d	m	Yes	2 roots

MAX THI: maximum thickness of cementum;
LOC MAX: location of maximum thickness;
LOC MIN: location of minimum thickness;
(1: apical root third; 2: middle root third; 3: cervical root third; m: mesial; d: distal; <: buccal; >: lingual)
PREF LOC: preferential location;

Excluded

Sp1172_24

Tooth	Occlusal Wear			CAR Si/Sta	Pulp EXP	IMP	Hypercementosis	Bone Context			
	DEG	DIR	FOR					FEN	CAL	NT	ANTA
ULP3	6	2	4	-	0	0	3.2.M	0	0	1d	1

ULP3

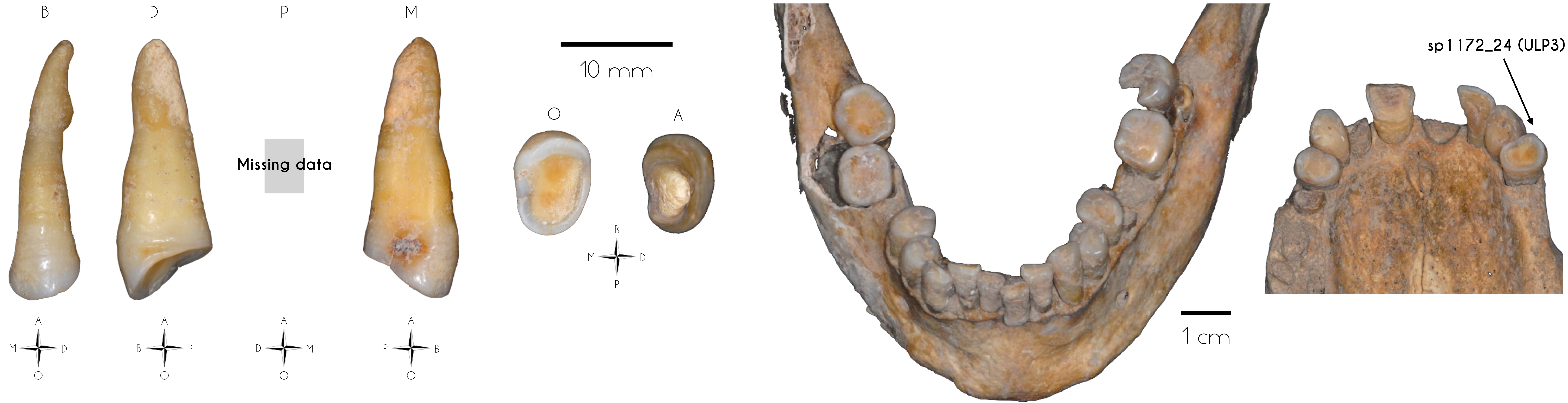

DEG: degree; DIR: direction; FOR: form - CAR: carious lesion - Pulp EXP: pulp exposure - IMP: impaction - FEN: fenestration; CAL: calculus; NT: tooth loss in neighbouring teeth; ANTA: antagonist

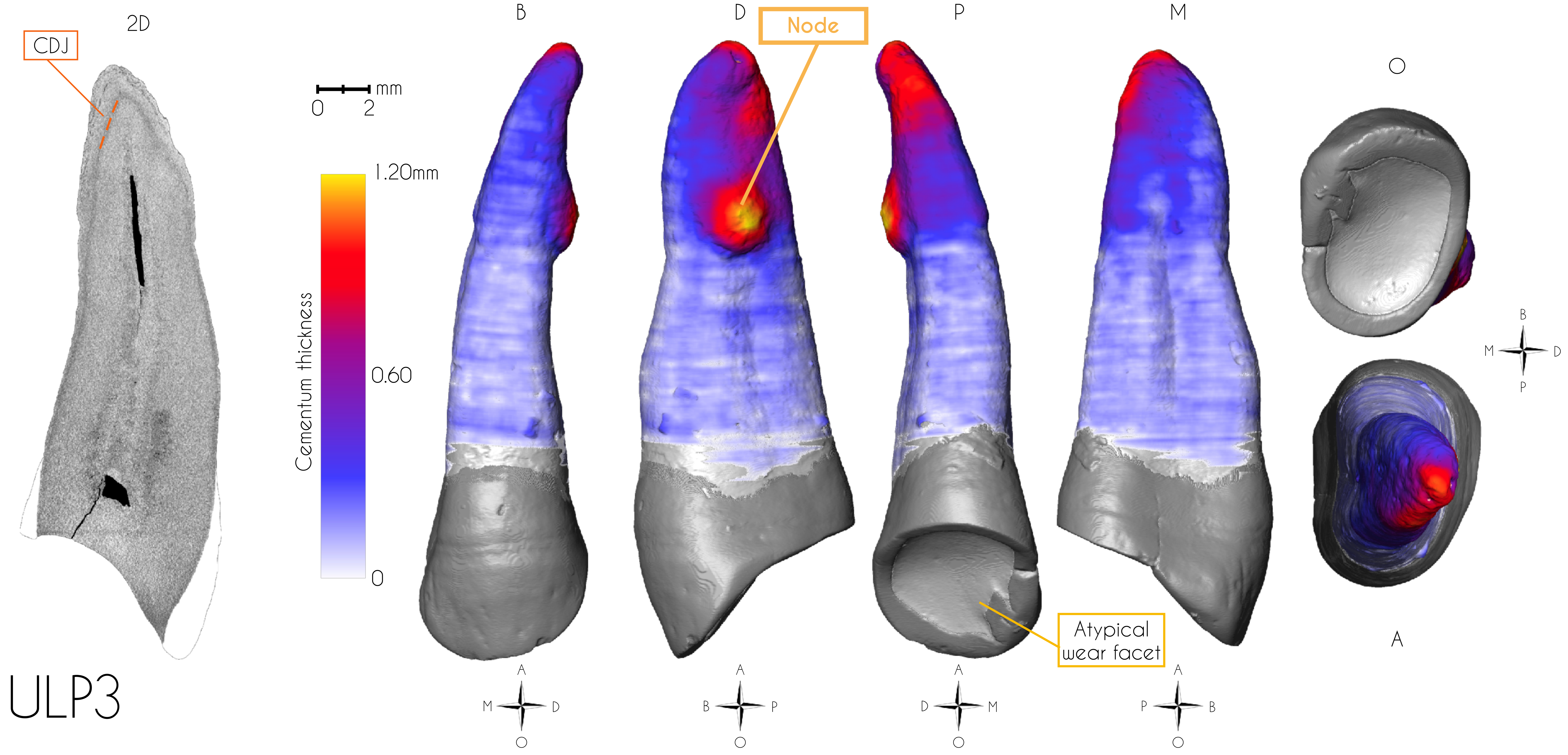

Sp1172_24

Microtomography - Thickness 3D maps				
MAX THI (μm)	LOC MAX	LOC MIN	PREF LOC	Note
1200	2d	<	Yes	NOD (d)

MAX THI: maximum thickness of cementum;
LOC MAX: location of maximum thickness;
LOC MIN: location of minimum thickness;
PREF LOC: preferential location;
NOD: node;
(1: apical root third; 2: middle root third; 3: cervical root third; m: mesial; d: distal; <: buccal; >: lingual)

<u>Hyperfunctional</u>

Sp1135_11

URI1

Tooth	Occlusal Wear			CAR Si/Sta	Pulp EXP	IMP	Hypercementosis	Bone Context			
	DEG	DIR	FOR					FEN	CAL	NT	ANTA
**URI1**	**6**	**4**	**5**	**-**	**0**	**0**	**1.3.m**	**0**	**0**	**0**	**1**

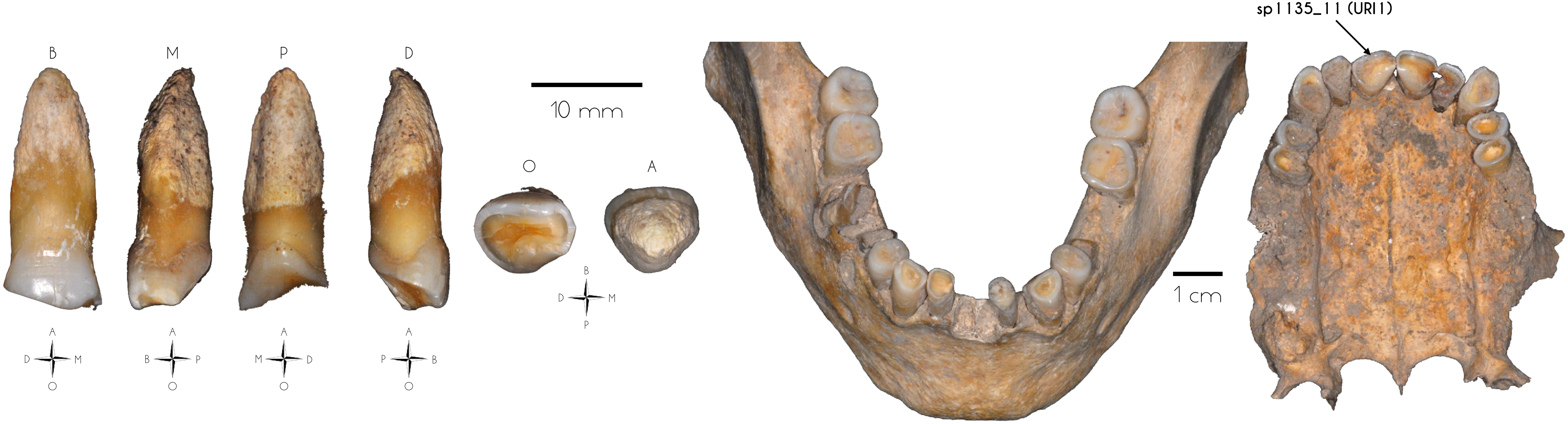

DEG: degree; **DIR:** direction; **FOR:** form - **CAR**: carious lesion - **Pulp EXP:** pulp exposure - **IMP**: impaction - **FEN:** fenestration; **CAL:** calculus; **NT:** tooth loss in neighbouring teeth; **ANTA:** antagonist

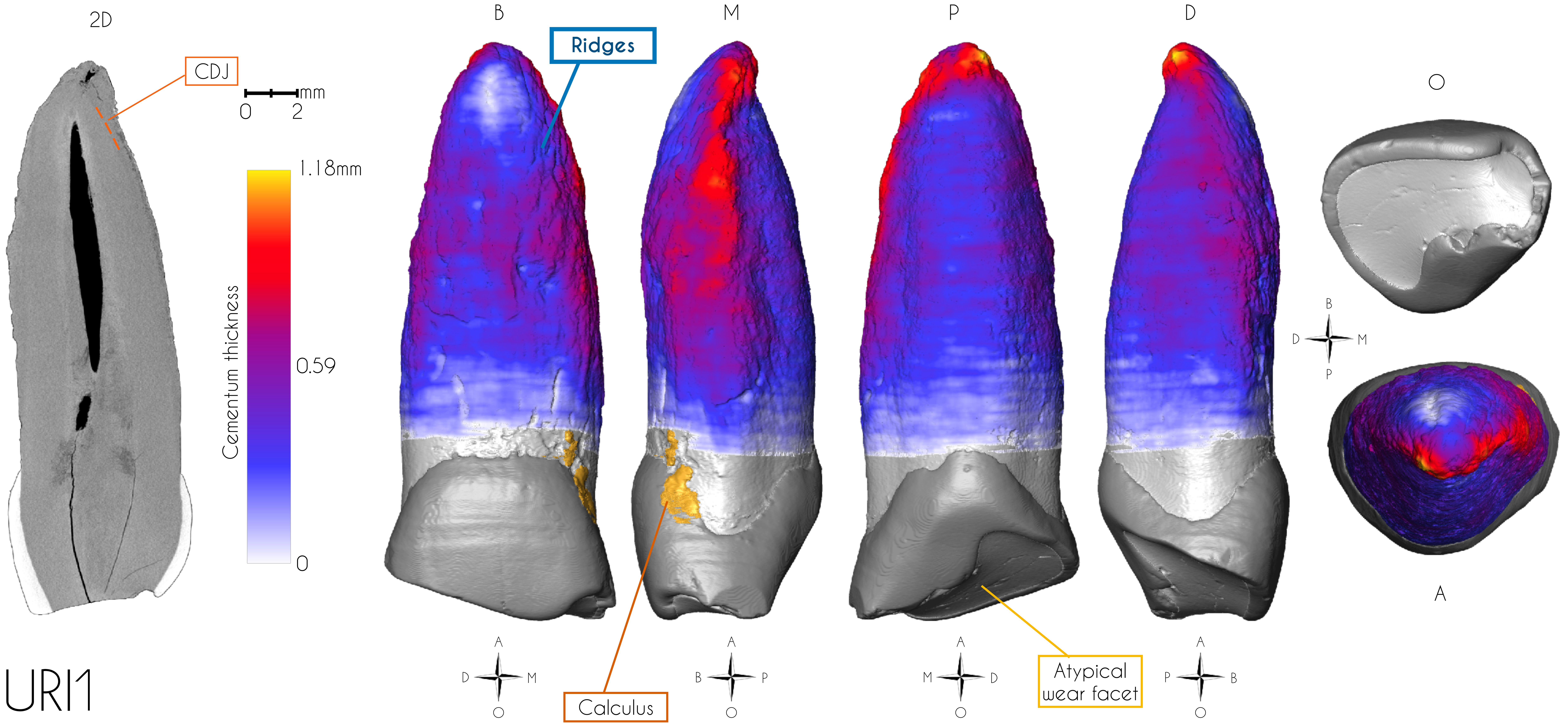

Sp1135_11

Microtomography - Thickness 3D maps				
MAX THI (μm)	LOC MAX	LOC MIN	PREF LOC	Note
1180	1>d +2m	1<	Yes	RID (<)

MAX THI: maximum thickness of cementum;
LOC MAX: location of maximum thickness;
LOC MIN: location of minimum thickness;
PREF LOC: preferential location;
RID: ridges;
(1: apical root third; 2: middle root third; 3: cervical root third; m: mesial; d: distal; <: buccal; >: lingual)

<u>Hyperfunctional</u>

URI1

Face	Range	Light	Width	Height	Columns/Rows	Factor	Led Color	Gain	Gamma	Overlapping
M	1143	5.90%	0.88mm	4.23mm	1/7	2	Blue	1.49	1.79	10 %

Sp1135_11

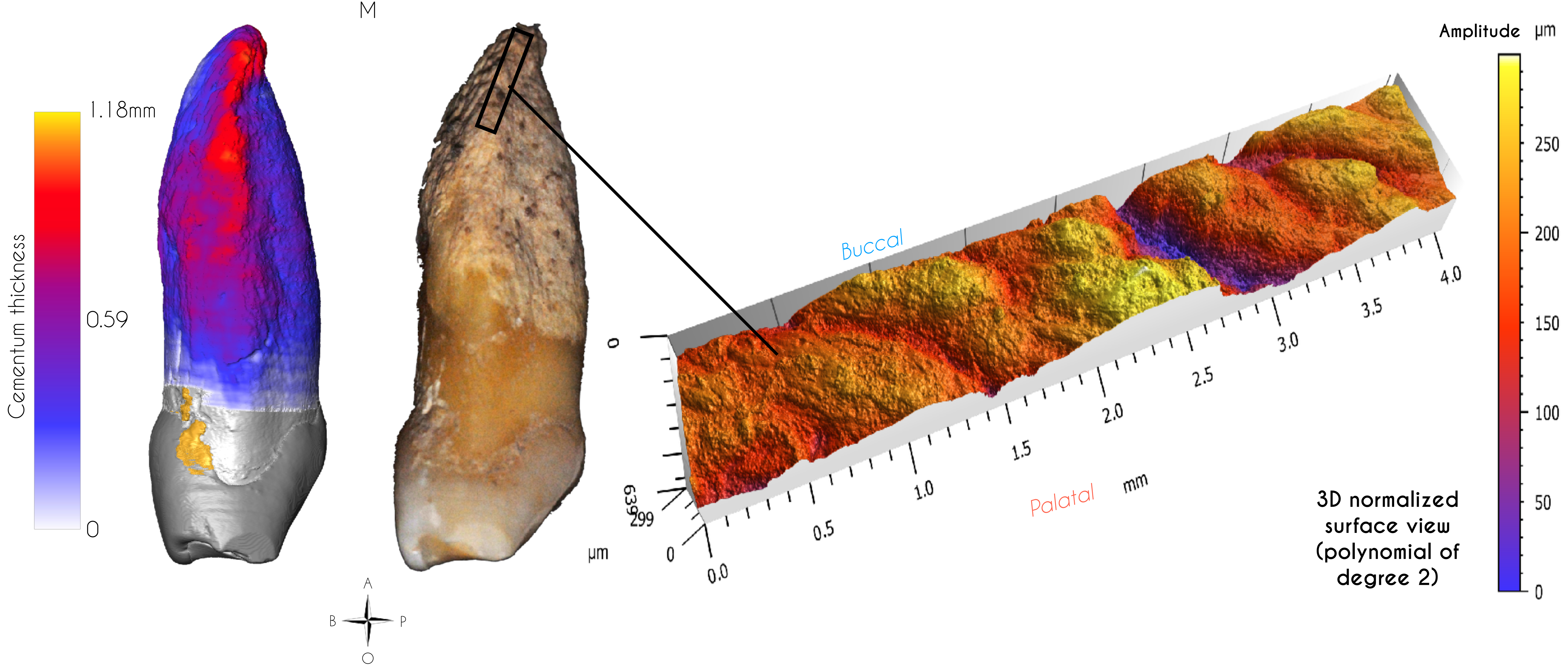

3D normalized surface view (polynomial of degree 2)

Confocal microscopy - Topography 3D maps			
LOC MAX	MAX VE (µm)	ST	Note
1m	300	+R1	-

LOC MAX: location of maximum thickness (1: apical root third; 2: middle root third; 3: cervical root third; m: mesial; d: distal; <: buccal; >: lingual);
MAX VE: maximum vertical elevation;
ST: surface texture; : scored (**+**) if it is high, (**-**) if it is low; **S:** smooth or **R:** rough; the frequency of occurrence of the elevations scored (**1**) if the frequency is high, (**2**) if the frequency is low.

Hyperfunctional

URI1

Face	Range	Light	Width	Height	Columns/Rows	Factor	Led Color	Gain	Gamma	Overlapping
BD	759	5.22%	0.88mm	4.82mm	1/8	2	Blue	1.5	1.8	10 %

Sp1135_11

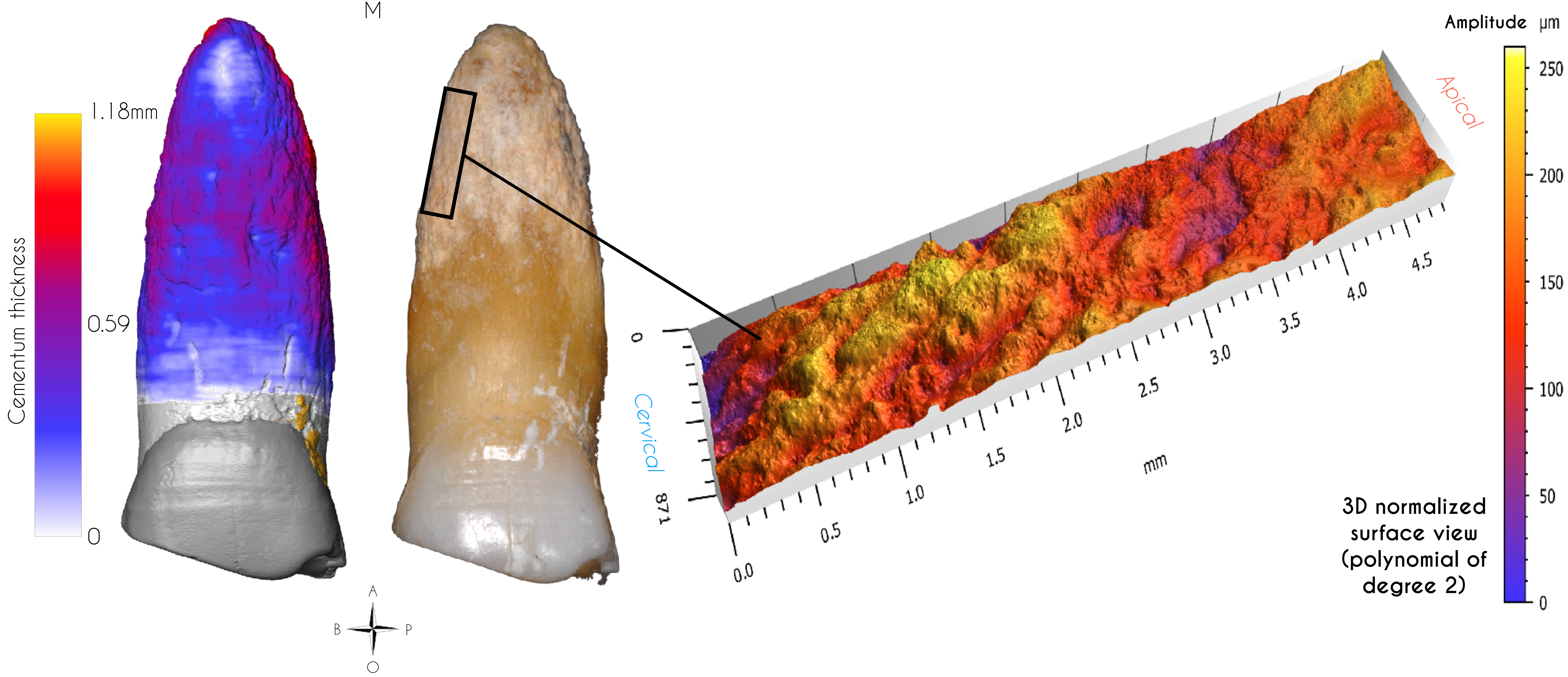

3D normalized surface view (polynomial of degree 2)

Confocal microscopy - Topography 3D maps			
OF	MAX VE (µm)	SA	Note
2<d	260	+R1	RID

OF: other face (1: apical root third; 2: middle root third; 3: cervical root third; m: mesial; d: distal; <: buccal; >: lingual);
MAX VE: maximum vertical elevation;
ST: surface texture; : scored **(+)** if it is high, **(-)** if it is low; **S:** smooth or **R:** rough; the frequency of occurrence of the elevations scored **(1)** if the frequency is high, **(2)** if the frequency is low.
RID: ridges

Hyperfunctional

Sp583_45

Tooth	Occlusal Wear			CAR Si/Sta	Pulp EXP	IMP	Hypercementosis	Bone Context			
	DEG	DIR	FOR					FEN	CAL	NT	ANTA
LRP4	6	4	2	-	0	0	3.2.M	0	1	1d	2

LRP4

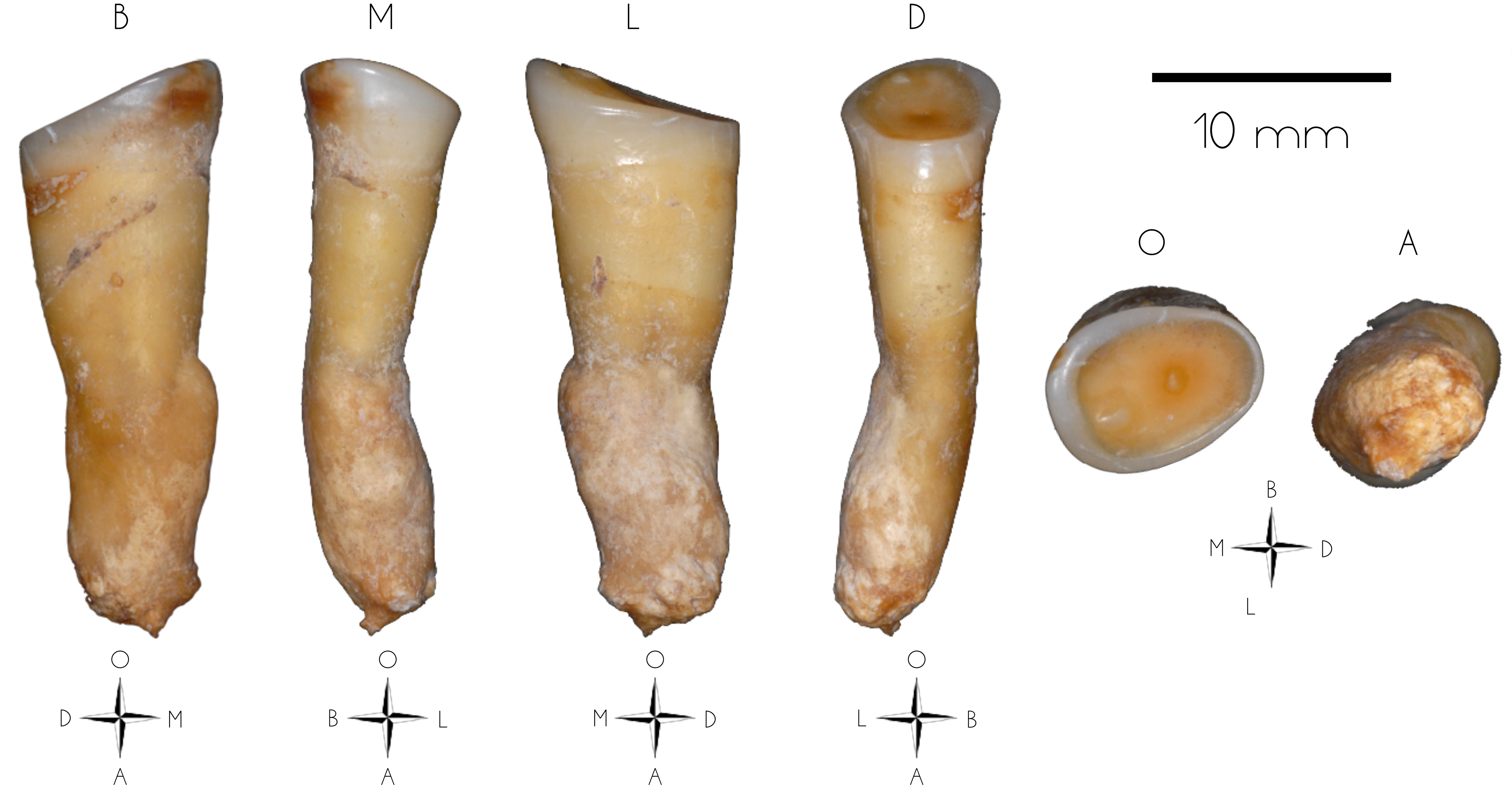

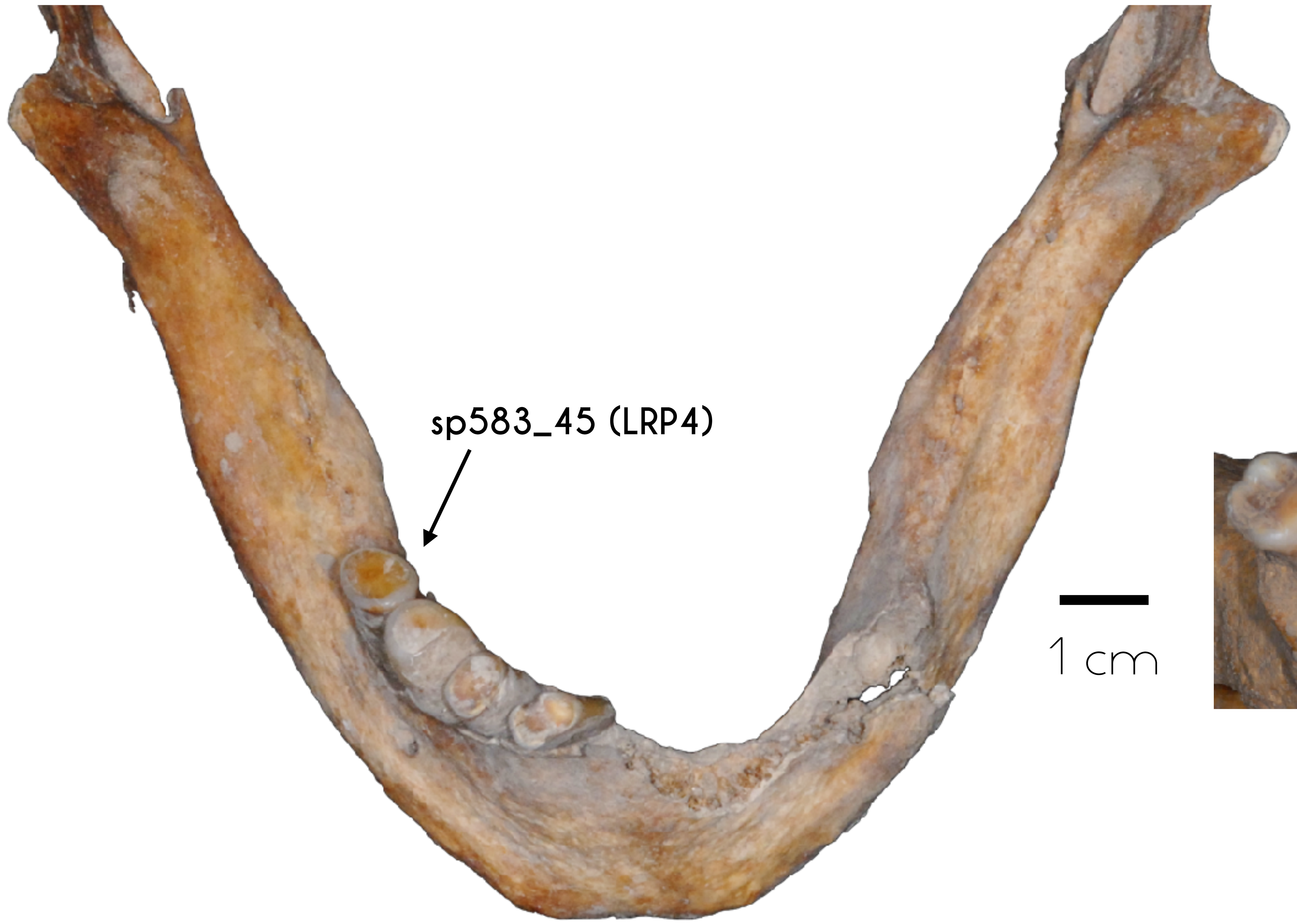

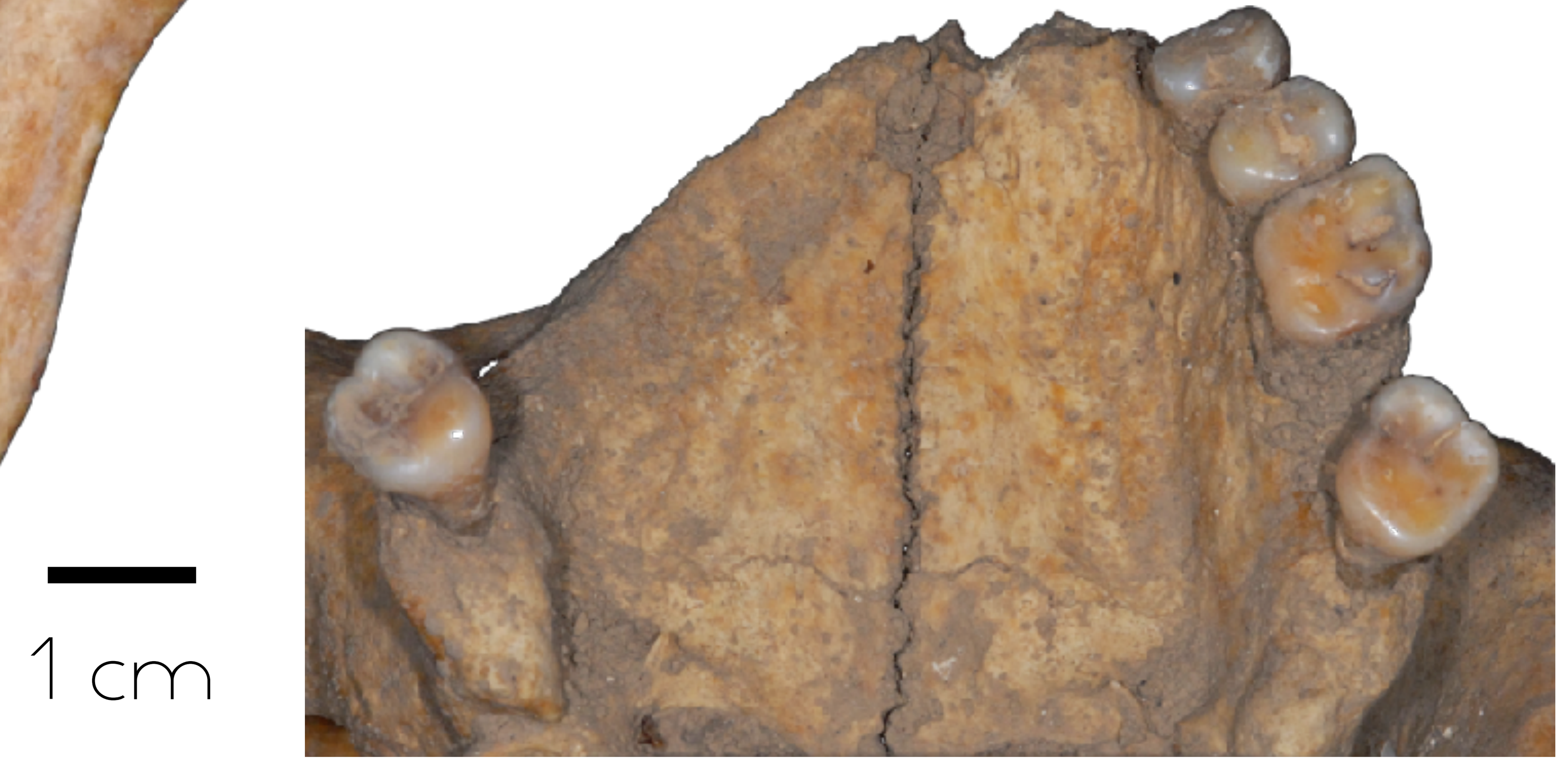

DEG: degree; **DIR:** direction; **FOR:** form - **CAR**: carious lesion - **Pulp EXP:** pulp exposure - **IMP**: impaction - **FEN:** fenestration; **CAL:** calculus; **NT:** tooth loss in neighbouring teeth; **ANTA:** antagonist

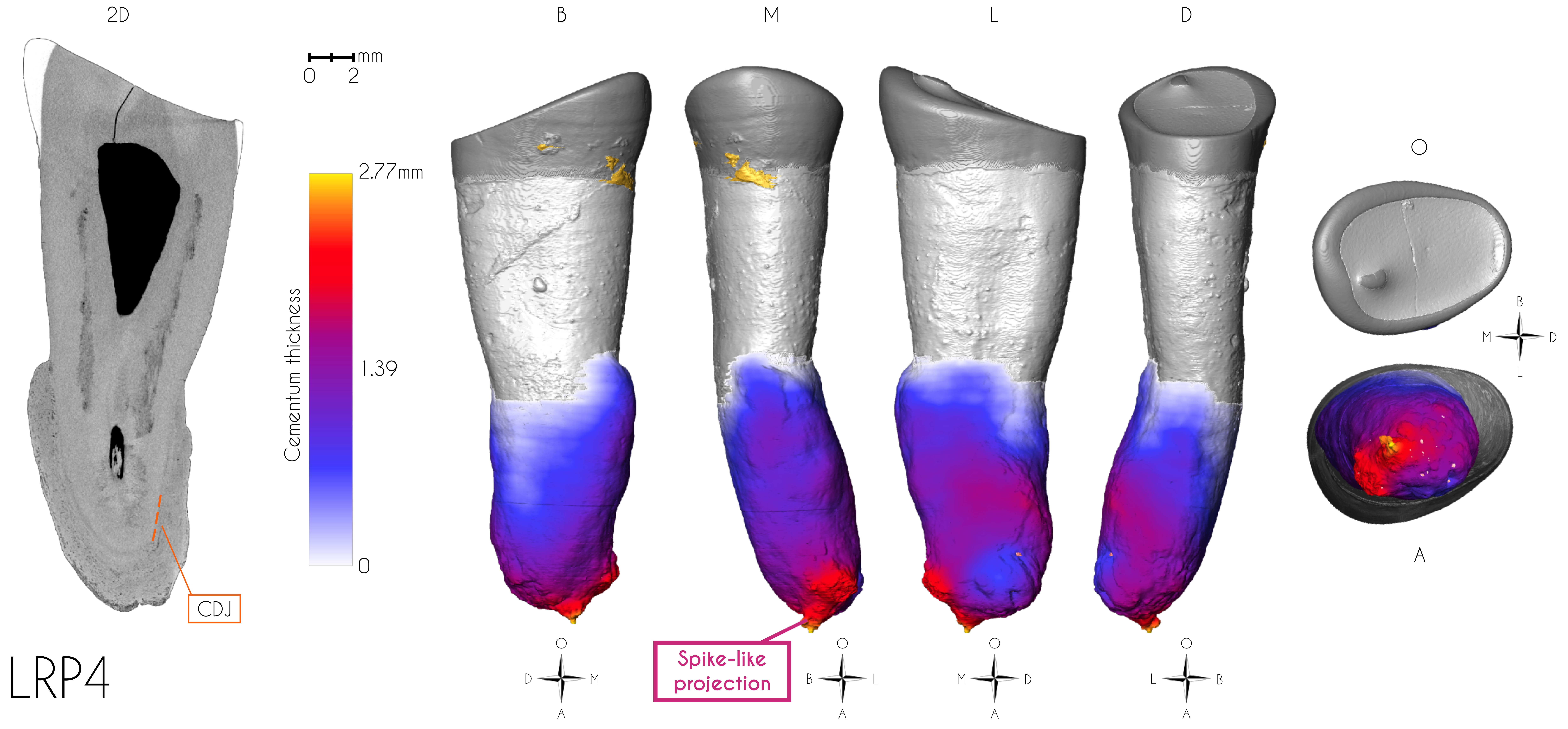

Sp583_45

Microtomography - Thickness 3D maps				
MAX THI (µm)	LOC MAX	LOC MIN	PREF LOC	Note
2770	1	<	Yes	LSP (1)

MAX THI: maximum thickness of cementum;
LOC MAX: location of maximum thickness;
LOC MIN: location of minimum thickness;
PREF LOC: preferential location;
LSP: localised spike-like projection;
(1: apical root third; 2: middle root third; 3: cervical root third; m: mesial; d: distal; <: buccal; >: lingual)

Mixed

LRP4

Face	Range	Light	Width	Height	Columns/Rows	Factor	Led Color	Gain	Gamma	Overlapping
L	417	4.97%	2.46mm	1.85mm	3/3	2	Blue	1.47	1.61	10 %

Sp583_45

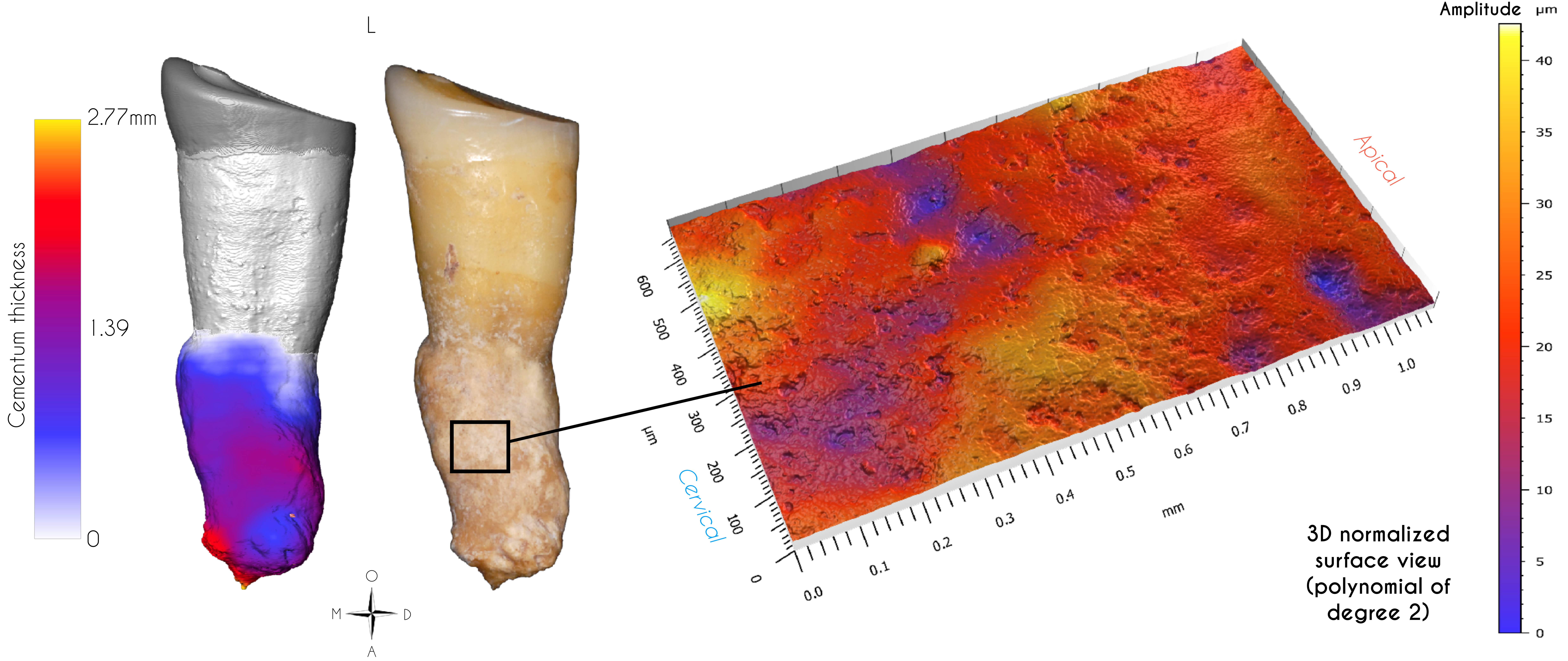

Confocal microscopy - Topography 3D maps			
OF	MAX VE (µm)	ST	Note
2>	43	-S2	-

OF: other face (1: apical root third; 2: middle root third; 3: cervical root third; m: mesial; d: distal; <: buccal; >: lingual);
MAX VE: maximum vertical elevation;
ST: surface texture; : scored (**+**) if it is high, (**-**) if it is low; **S:** smooth or **R:** rough; the frequency of occurrence of the elevations scored (**1**) if the frequency is high, (**2**) if the frequency is low.

Mixed

Sp666_33

Tooth	Occlusal Wear			CAR	Pulp EXP	IMP	Hypercementosis	Bone Context			
	DEG	DIR	FOR	Si/Sta				FEN	CAL	NT	ANTA
**LLC**	**7**	**5**	**6**	**2/2**	**0**	**0**	**3.2.M**	**0**	**1**	**1m**	**NA**

LLC

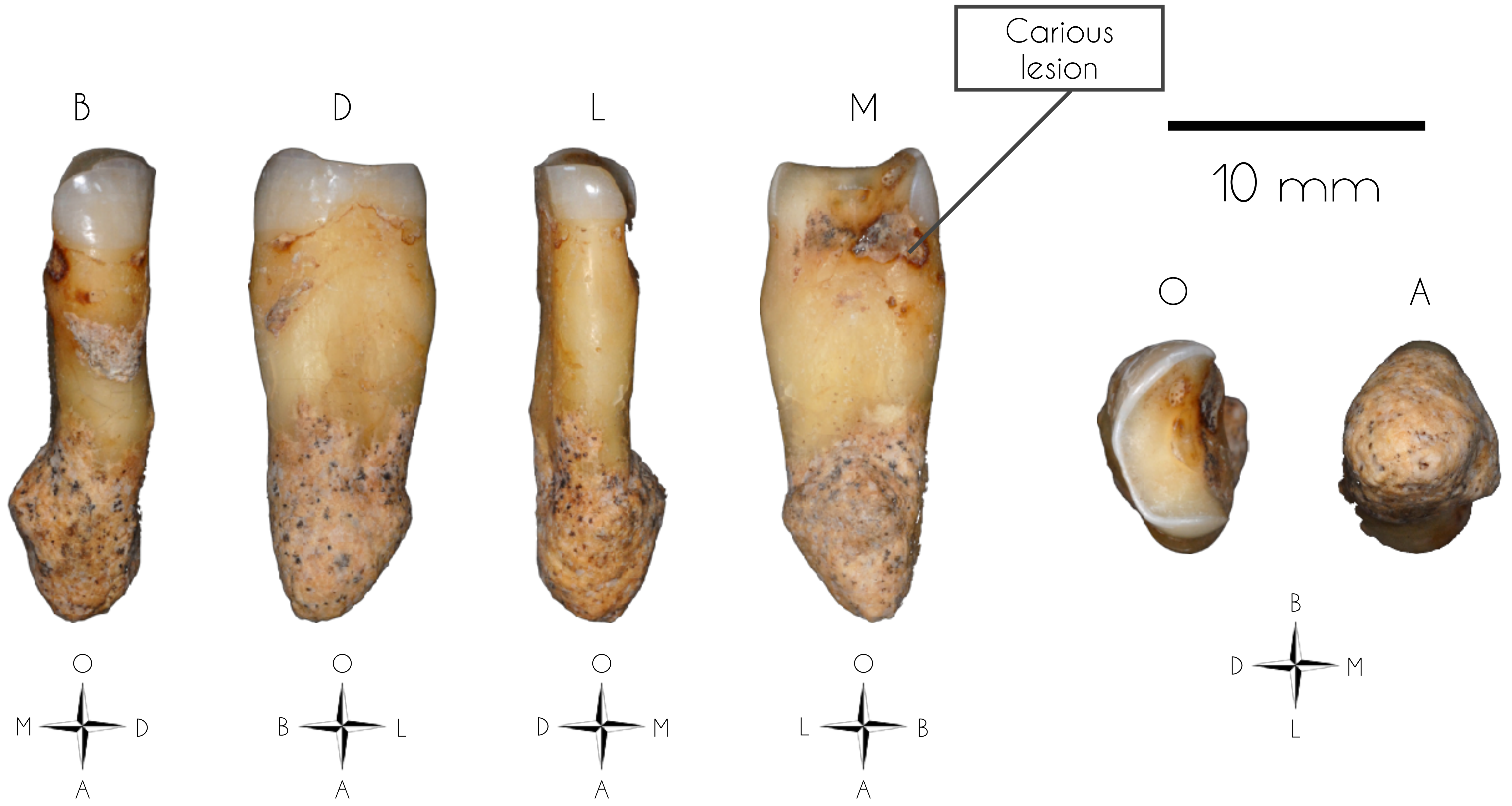

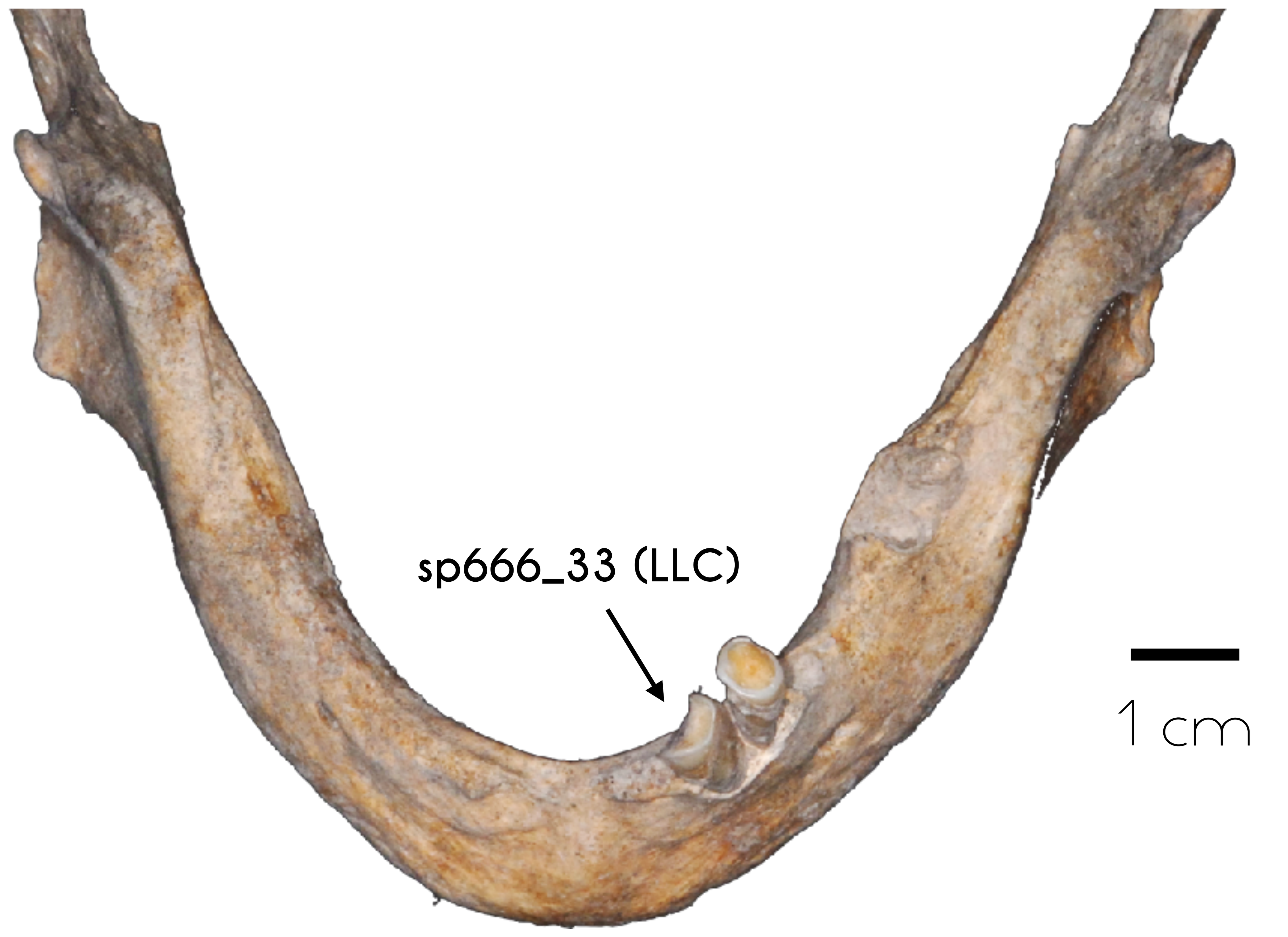

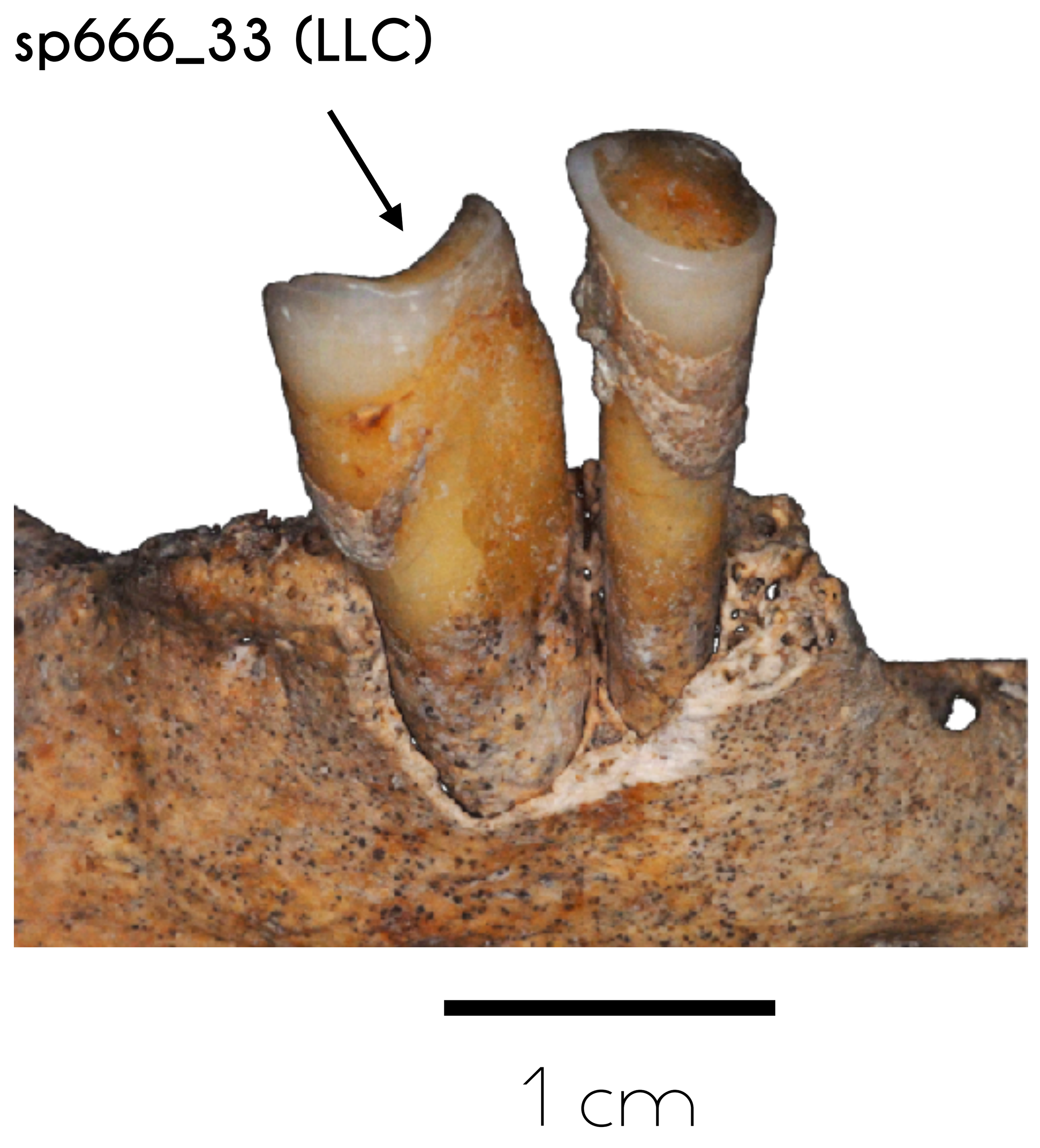

DEG: degree; **DIR:** direction; **FOR:** form - **CAR**: carious lesion - **Pulp EXP:** pulp exposure - **IMP**: impaction - **FEN:** fenestration; **CAL:** calculus; **NT:** tooth loss in neighbouring teeth; **ANTA:** antagonist

Sp666_33

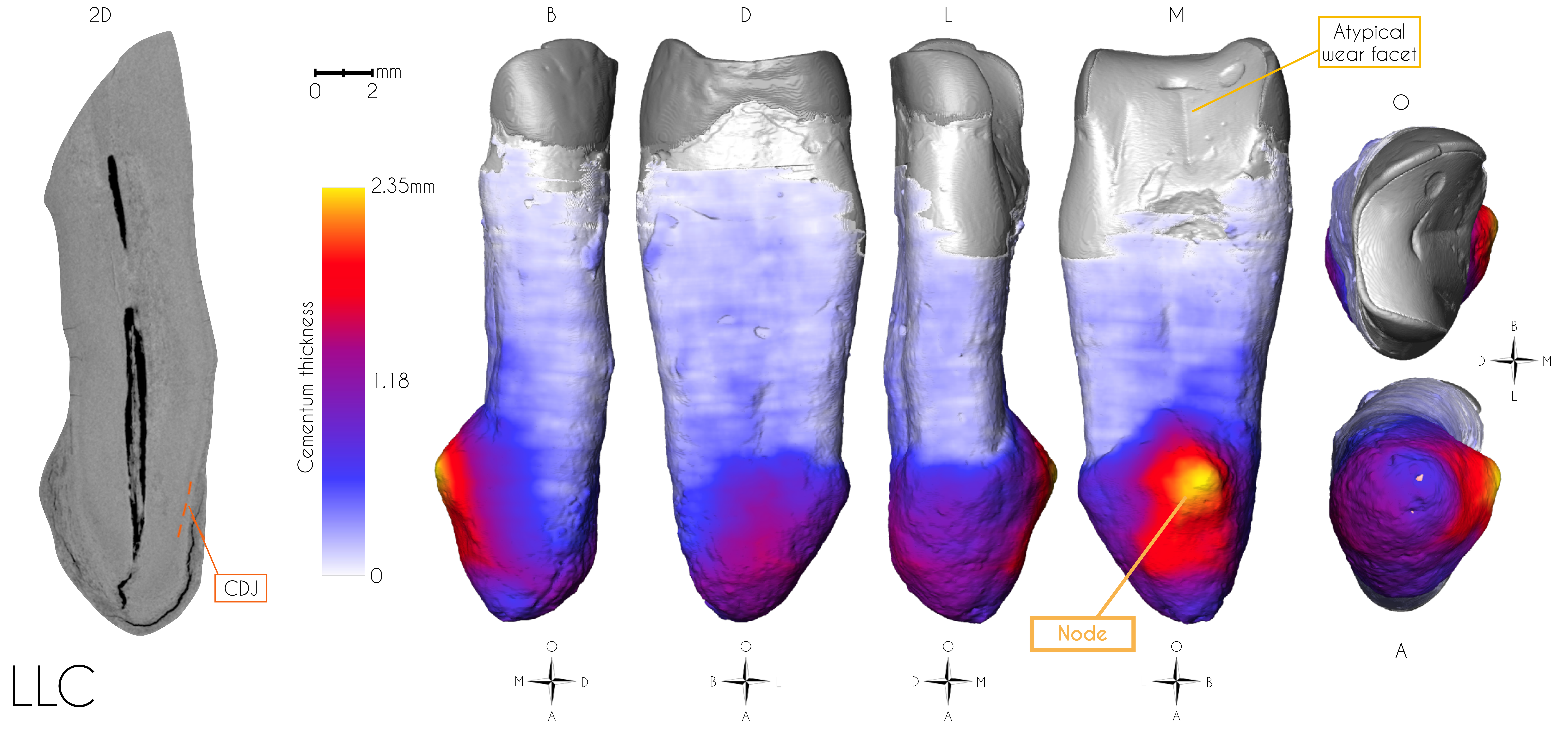

Microtomography - Thickness 3D maps				
MAX THI (µm)	LOC MAX	LOC MIN	PREF LOC	Note
2350	1m	<	Yes	NOD + CAR (m)

MAX THI: maximum thickness of cementum;
LOC MAX: location of maximum thickness;
LOC MIN: location of minimum thickness;
PREF LOC: preferential location;
NOD: node;
CAR: carious lesion
(1: apical root third; 2: middle root third; 3: cervical root third; m: mesial; d: distal; <: buccal; >: lingual)

Mixed

LLC

Face	Range	Light	Width	Height	Columns/Rows	Factor	Led Color	Gain	Gamma	Overlapping
M	1731	6.02%	0.88mm	4.23	1/7	2	Blue	1.5	1.8	10 %

Sp666_33

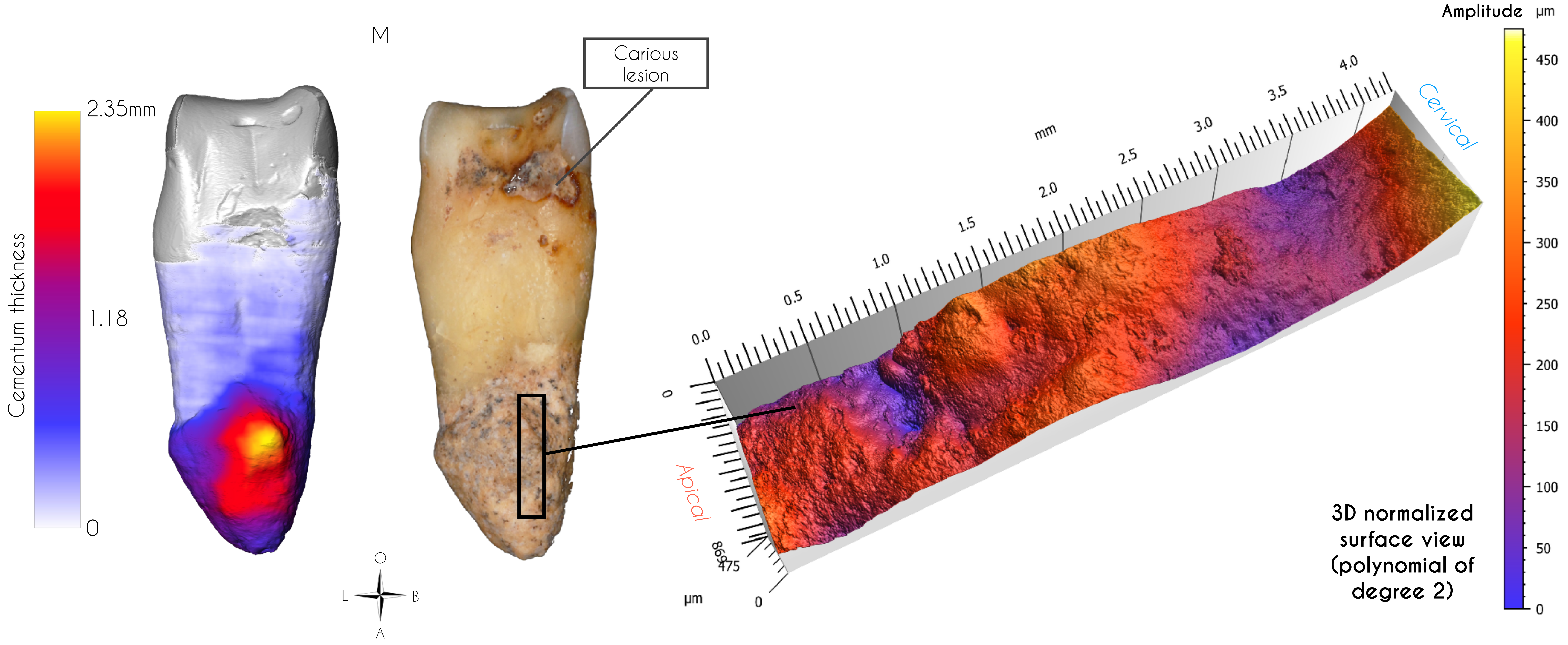

Confocal microscopy - Topography 3D maps			
LOC MAX	MAX VE (µm)	ST	Note
1m	475	+S2	NOD

LOC MAX: location of maximum thickness (1: apical root third; 2: middle root third; 3: cervical root third; m: mesial; d: distal; <: buccal; >: lingual);
MAX VE: maximum vertical elevation;
ST: surface texture; : scored (**+**) if it is high, (**-**) if it is low; **S:** smooth or **R:** rough; the frequency of occurrence of the elevations scored (**1**) if the frequency is high, (**2**) if the frequency is low.
NOD: nodes

Mixed

Sp709_31

Tooth	Occlusal Wear			CAR Si/Sta	Pulp EXP	IMP	Hypercementosis	Bone Context			
	DEG	DIR	FOR					FEN	CAL	NT	ANTA
LLI1	6	6	4	-	0	0	3.1.m	0	1	0	NA

LLI1

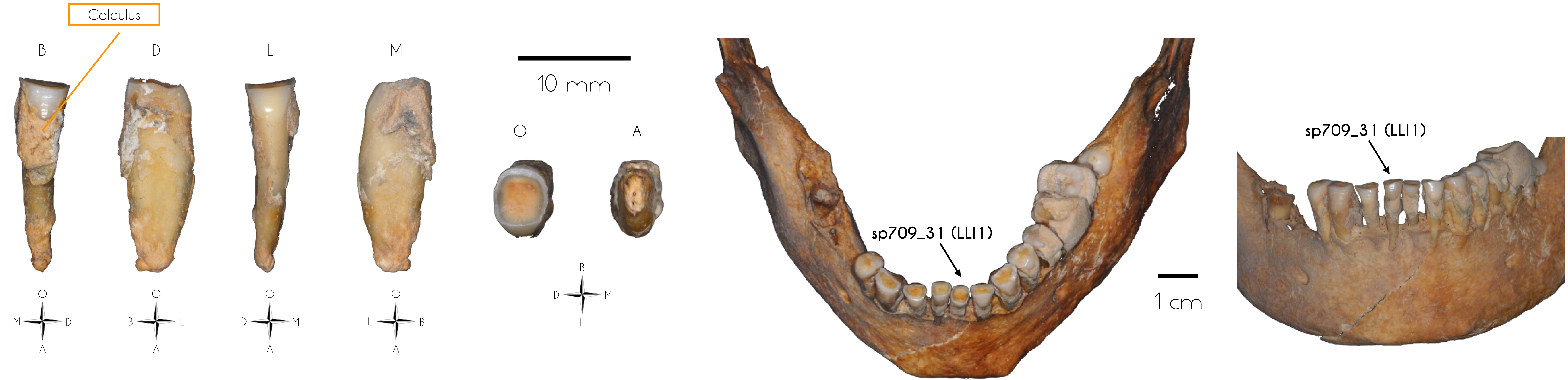

DEG: degree; **DIR:** direction; **FOR:** form - **CAR**: carious lesion - **Pulp EXP:** pulp exposure - **IMP**: impaction - **FEN:** fenestration; **CAL:** calculus; **NT:** tooth loss in neighbouring teeth; **ANTA:** antagonist

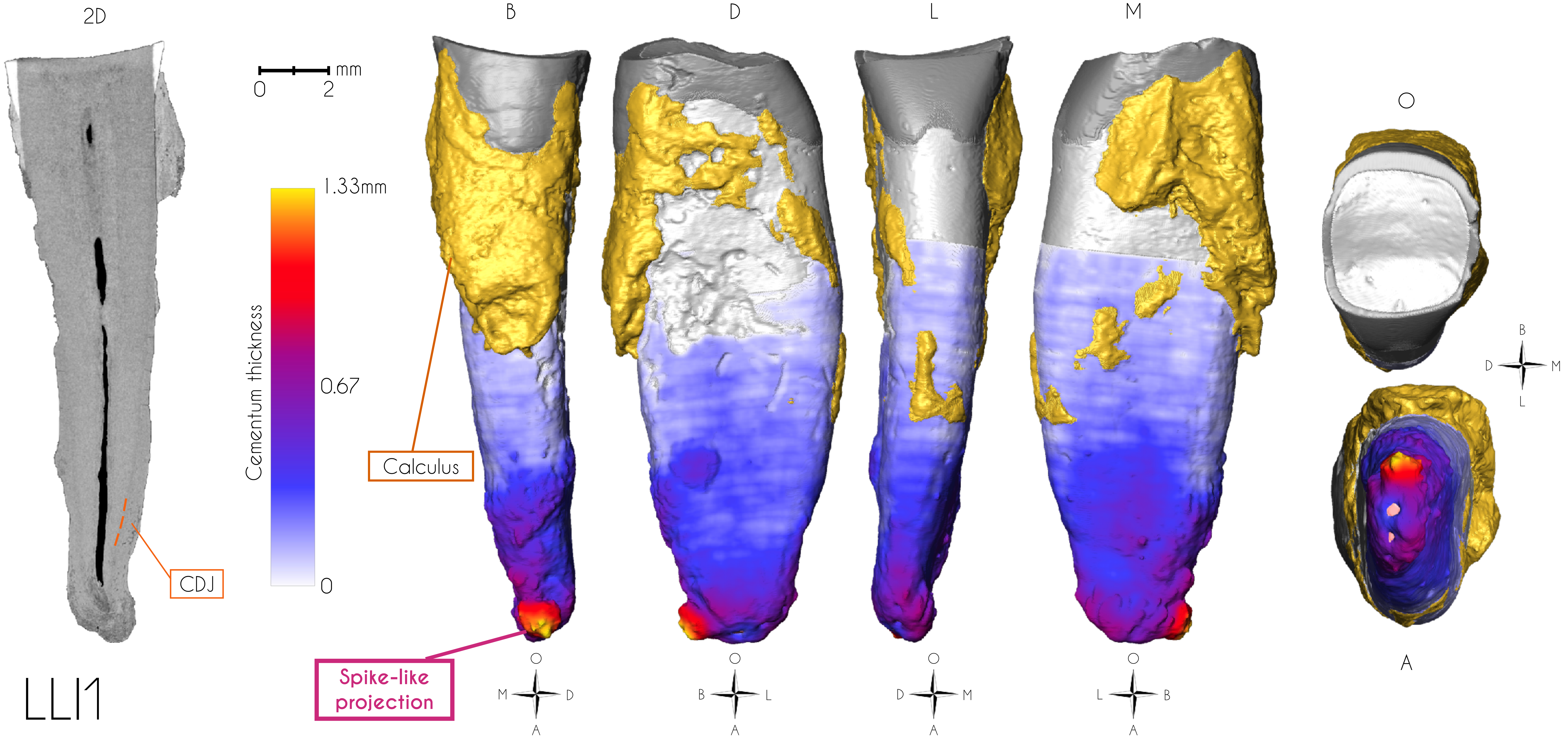

Sp709_31

Microtomography - Thickness 3D maps				
MAX THI (µm)	LOC MAX	LOC MIN	PREF LOC	Note
1330	1<	No	Yes	LSP (<)

MAX THI: maximum thickness of cementum;
LOC MAX: location of maximum thickness;
LOC MIN: location of minimum thickness;
PREF LOC: preferential location;
LSP: localised spike-like projection;
(1: apical root third; 2: middle root third; 3: cervical root third; m: mesial; d: distal; <: buccal; >: lingual)

Mixed

LLI1

Face	Range	Light	Width	Height	Columns/Rows	Factor	Led Color	Gain	Gamma	Overlapping
B	808	7.07%	0.88mm	2.44mm	1/4	2	Blue	1.5	1.8	10 %

Sp709_31

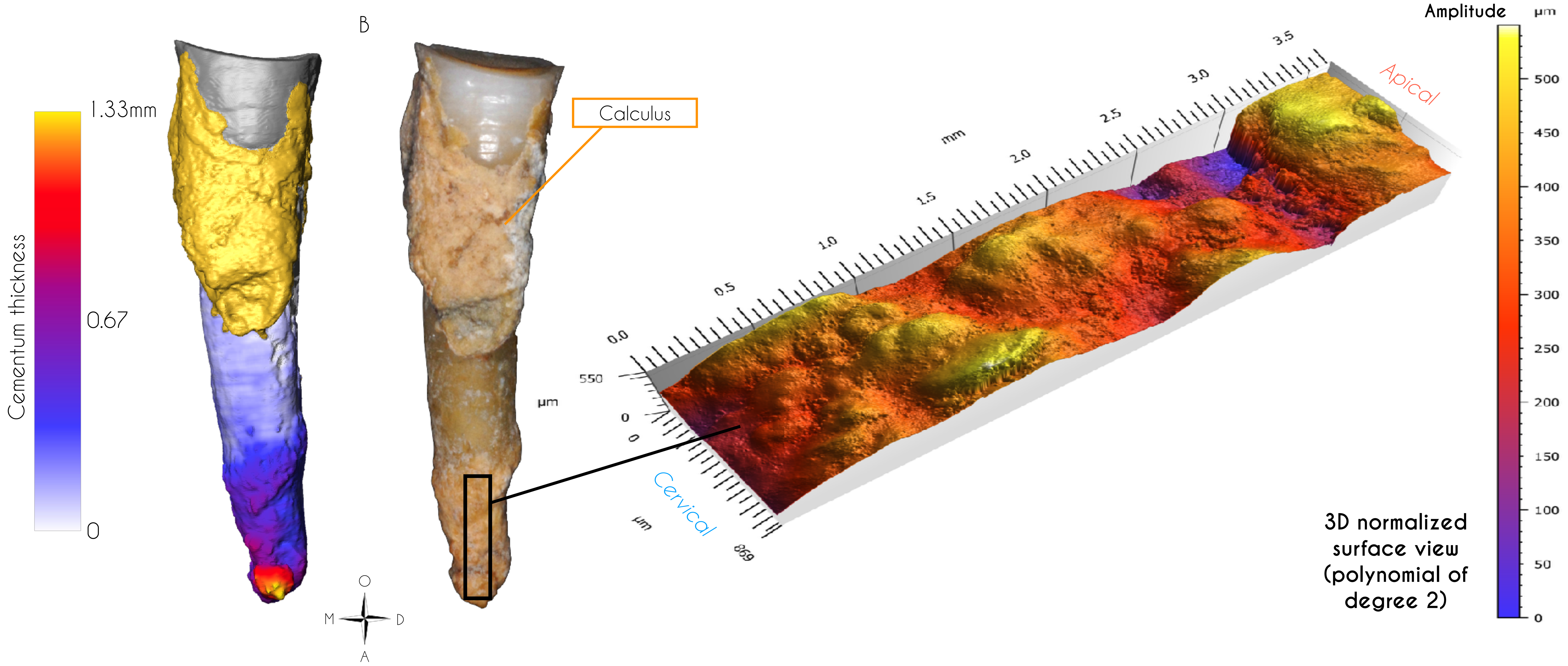

Confocal microscopy - Topography 3D maps			
LOC MAX	MAX VE (µm)	ST	Note
<	550	+R1	LSP

LOC MAX: location of maximum thickness (1: apical root third; 2: middle root third; 3: cervical root third; m: mesial; d: distal; <: buccal; >: lingual);
MAX VE: maximum vertical elevation;
ST: surface texture; : scored **(+)** if it is high, **(-)** if it is low; **S:** smooth or **R:** rough; the frequency of occurrence of the elevations scored **(1)** if the frequency is high, **(2)** if the frequency is low.
LSP: localised spike-like projection

Mixed

Sp735_45

Tooth	Occlusal Wear			CAR Si/Sta	Pulp EXP	IMP	Hypercementosis	Bone Context			
	DEG	DIR	FOR					FEN	CAL	NT	ANTA
**LRP4**	**4**	**6**	**3**	**2/4**	**C**	**0**	**1.2.M**	**0**	**0**	**1d**	**2**

LRP4

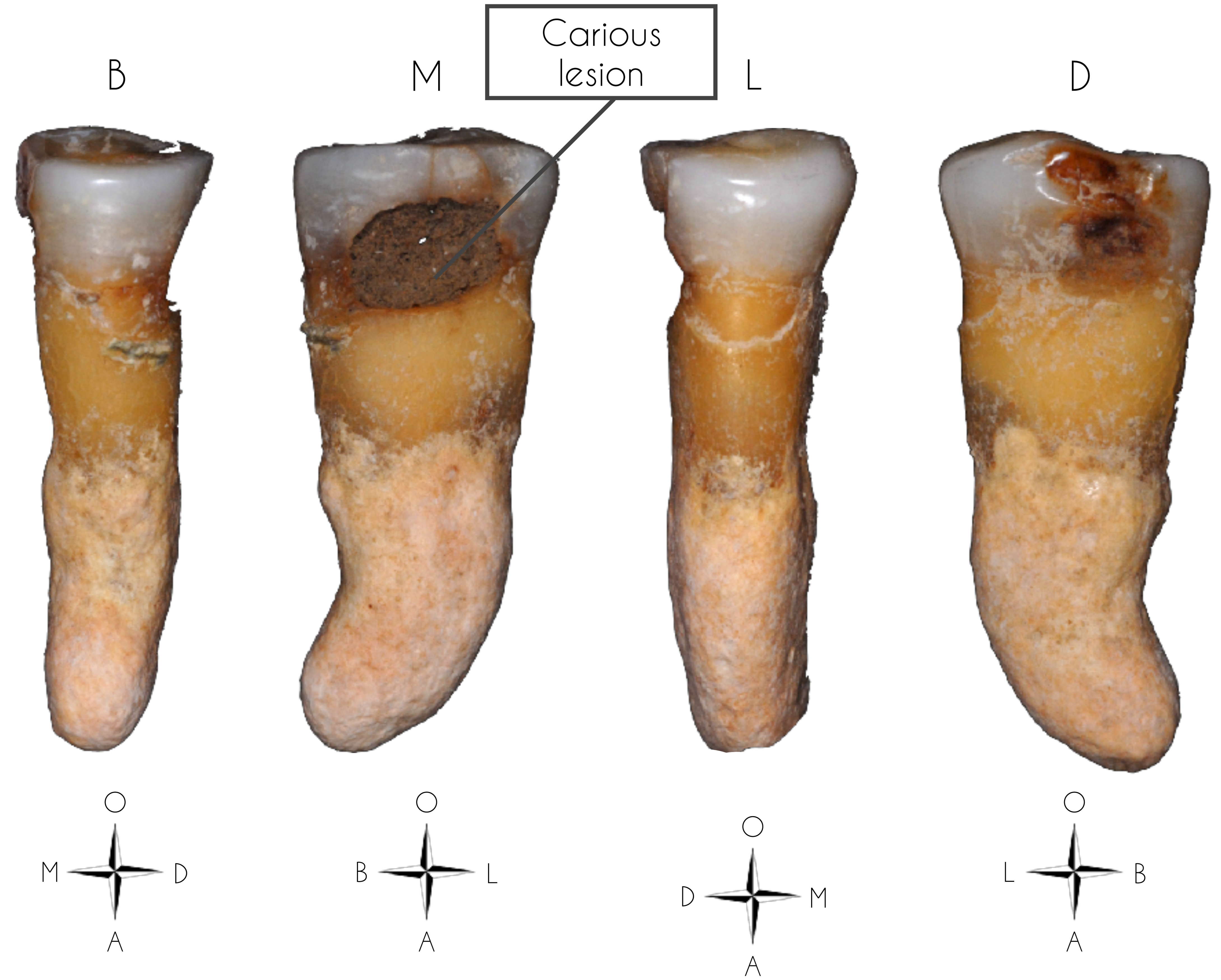

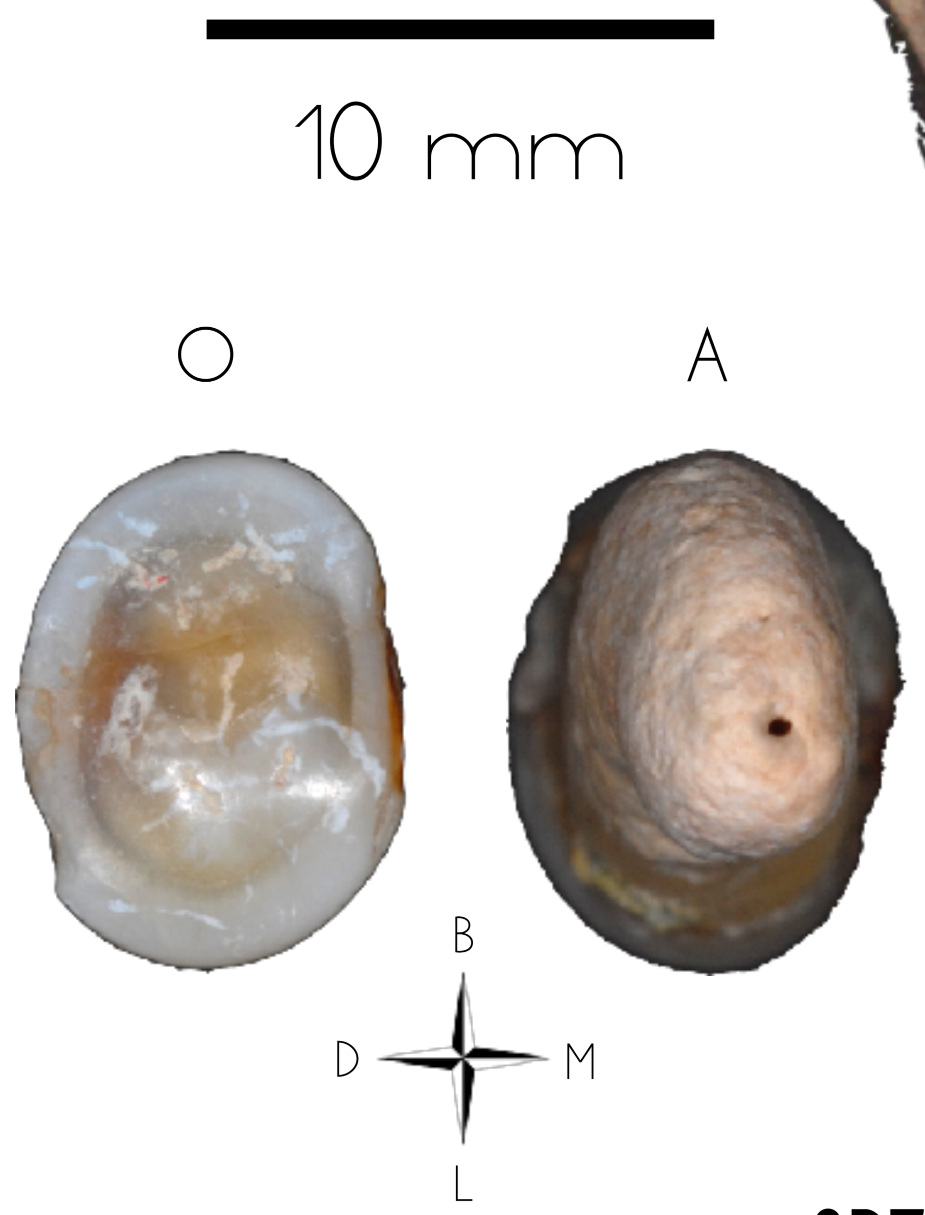

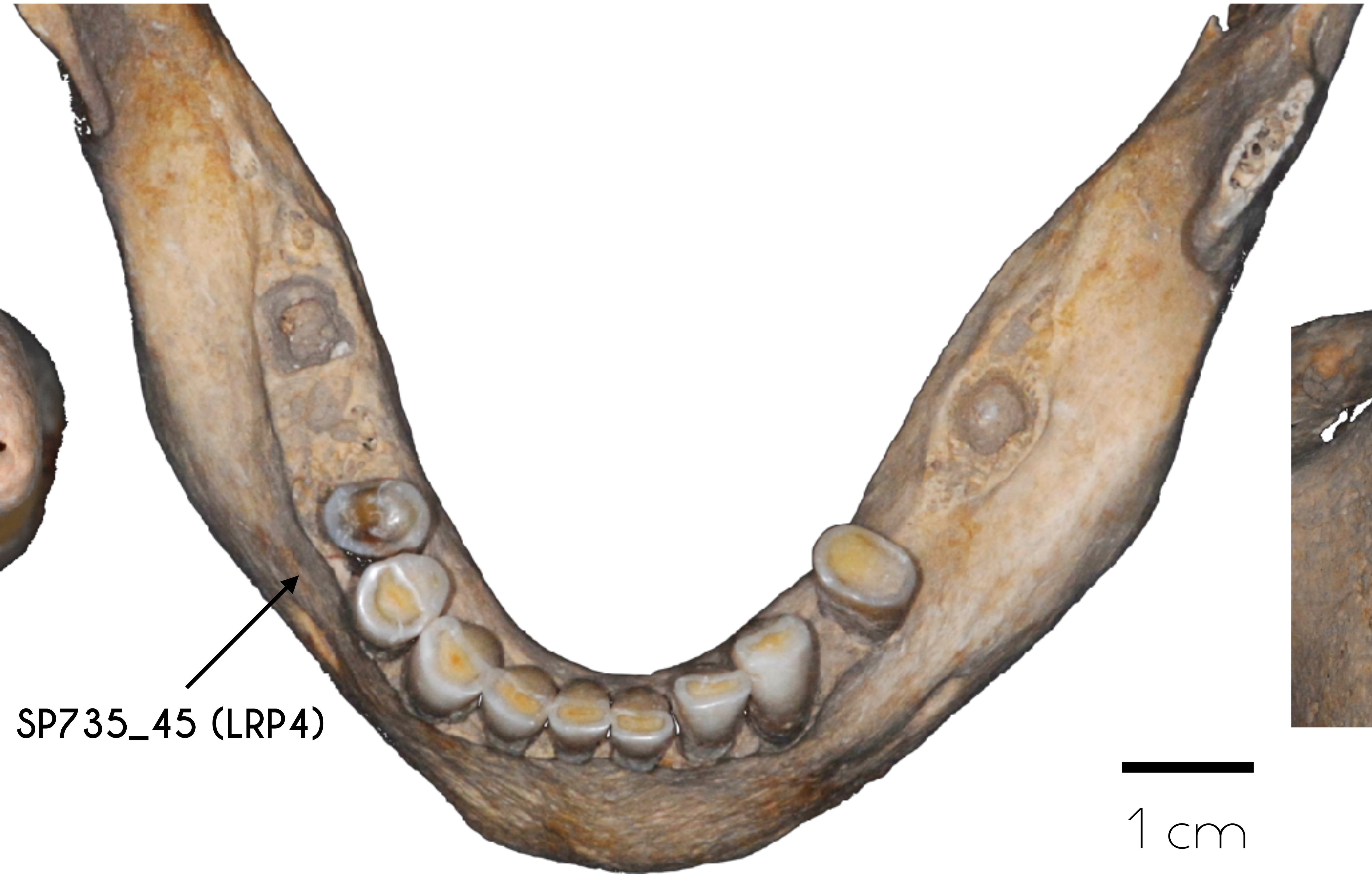

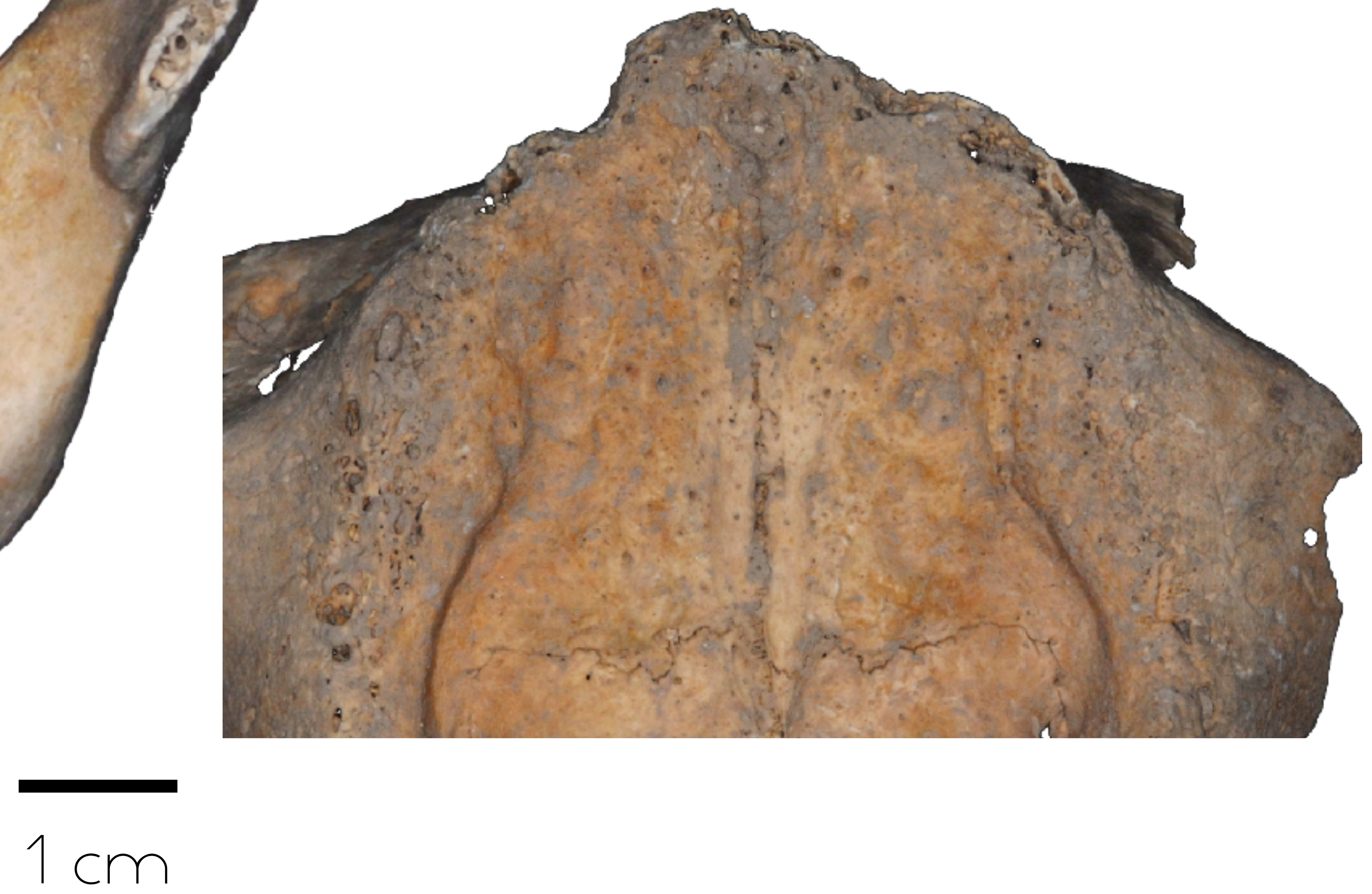

DEG: degree; **DIR:** direction; **FOR:** form - **CAR**: carious lesion - **Pulp EXP:** pulp exposure - **IMP**: impaction - **FEN:** fenestration; **CAL:** calculus; **NT:** tooth loss in neighbouring teeth; **ANTA:** antagonist

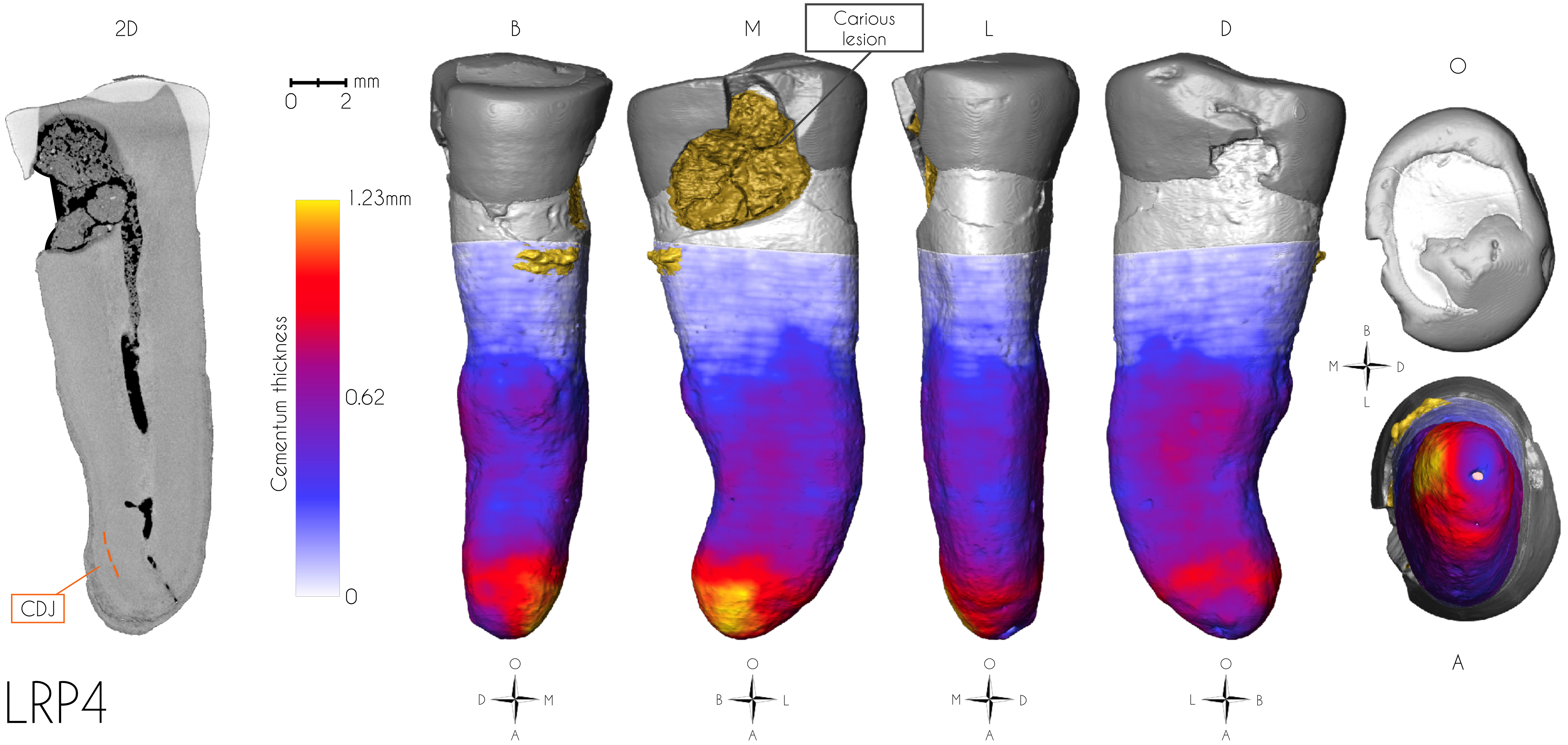

Sp735_45

Microtomography - Thickness 3D maps				
MAX THI (μm)	LOC MAX	LOC MIN	PREF LOC	Note
1230	1m	No	Yes	CAR (m)

MAX THI: maximum thickness of cementum;
LOC MAX: location of maximum thickness;
LOC MIN: location of minimum thickness;
PREF LOC: preferential location;
CAR: carious lesion;
(1: apical root third; 2: middle root third; 3: cervical root third; m: mesial; d: distal; <: buccal; >: lingual)

Mixed

LRP4

Face	Range	Light	Width	Height	Columns/Rows	Factor	Led Color	Gain	Gamma	Overlapping
M	1038	5.15%	1.67mm	1.85mm	2/3	2	Blue	1.5	1.8	10 %

Sp735_45

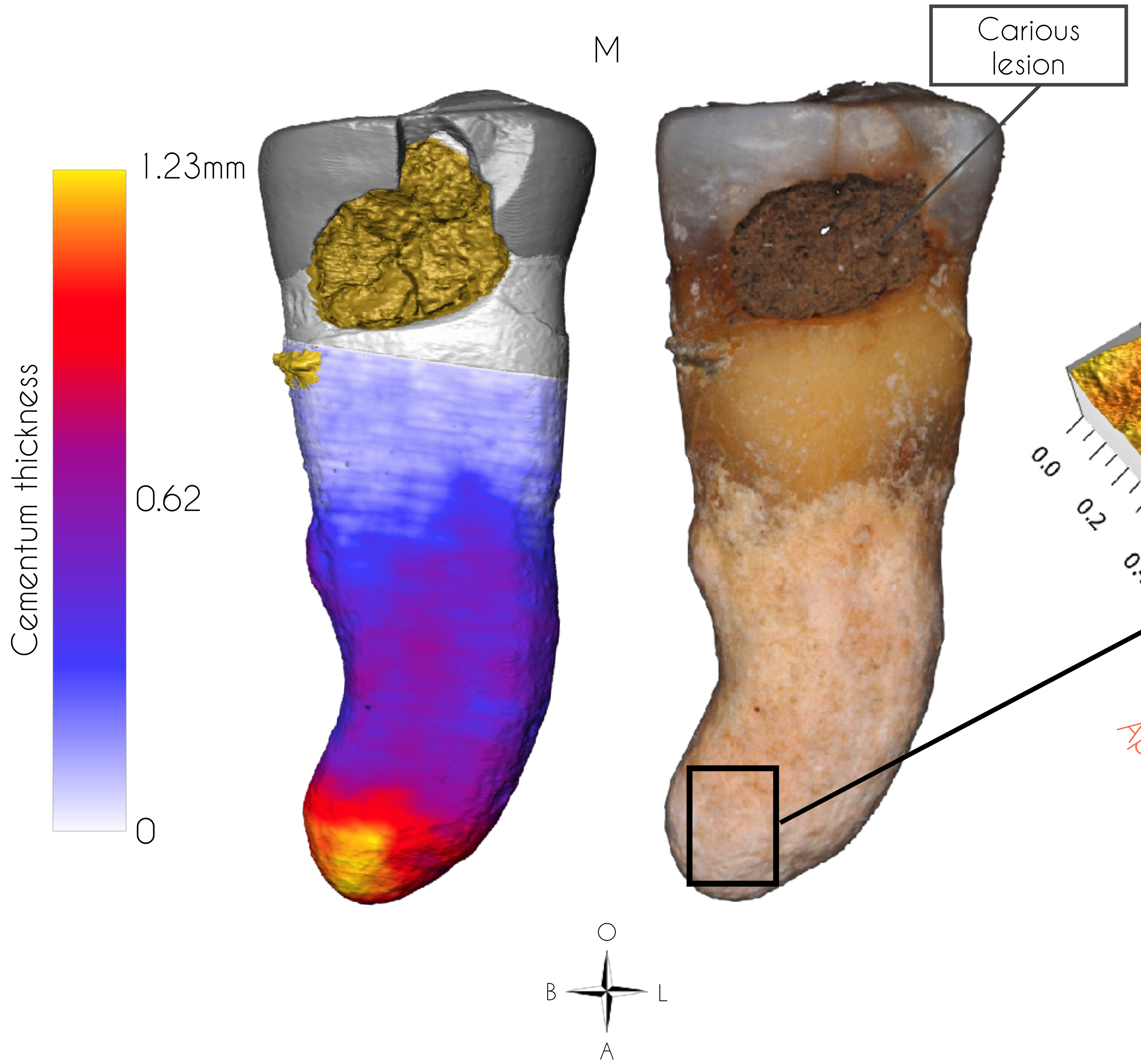

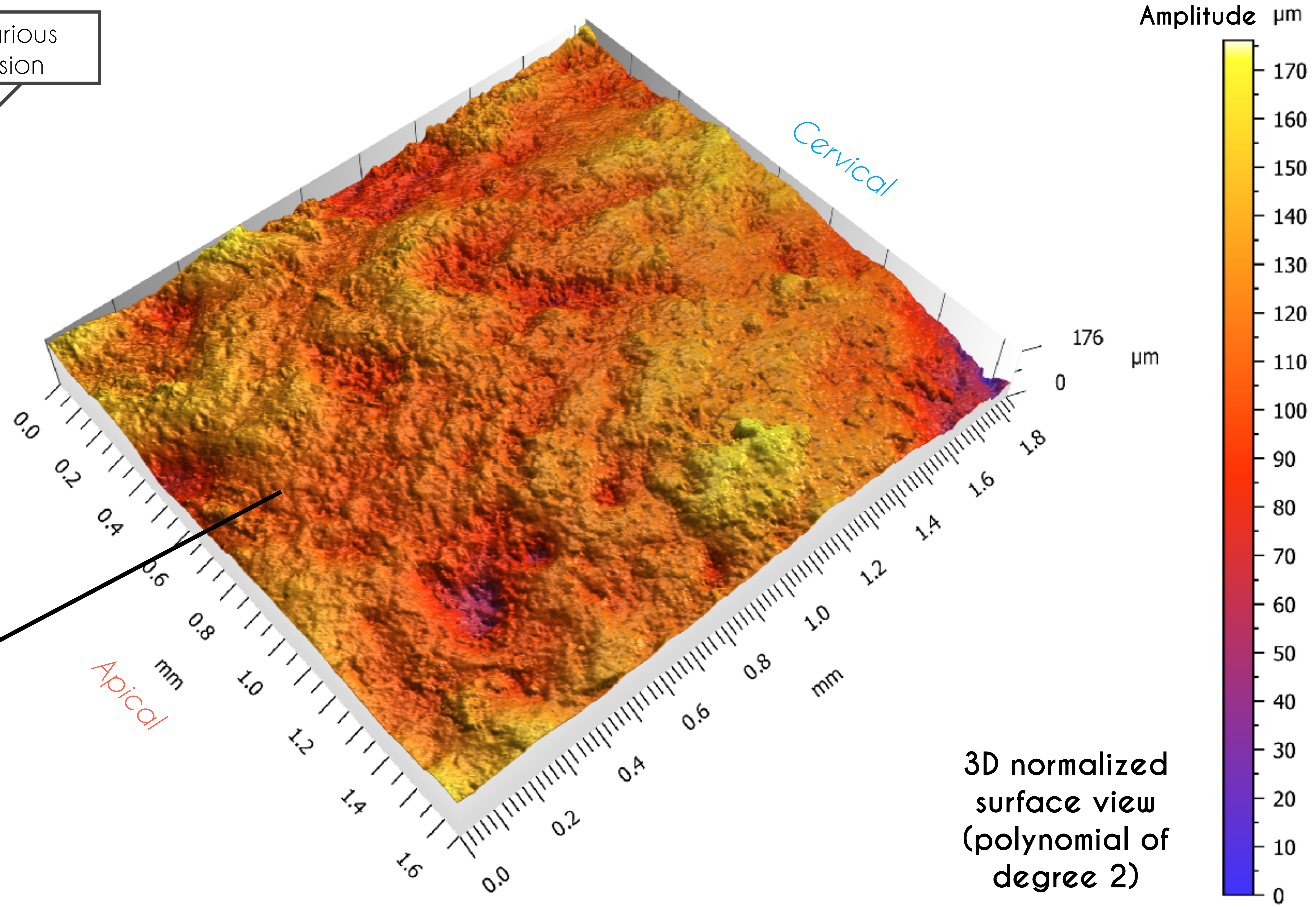

3D normalized surface view (polynomial of degree 2)

Confocal microscopy - Topography 3D maps			
LOC MAX	MAX VE (µm)	ST	Note
1m	175	-R1	-

LOC MAX: location of maximum thickness (1: apical root third; 2: middle root third; 3: cervical root third; m: mesial; d: distal; <: buccal; >: lingual);
MAX VE: maximum vertical elevation;
ST: surface texture; : scored (**+**) if it is high, (**-**) if it is low; **S:** smooth or **R:** rough; the frequency of occurrence of the elevations scored (**1**) if the frequency is high, (**2**) if the frequency is low.

Mixed

LRP4

Face	Range	Light	Width	Height	Columns/Rows	Factor	Led Color	Gain	Gamma	Overlapping
D	715	4.19%	1.67mm	1.25mm	2/2	2	Blue	1.5	1.8	10 %

Sp735_45

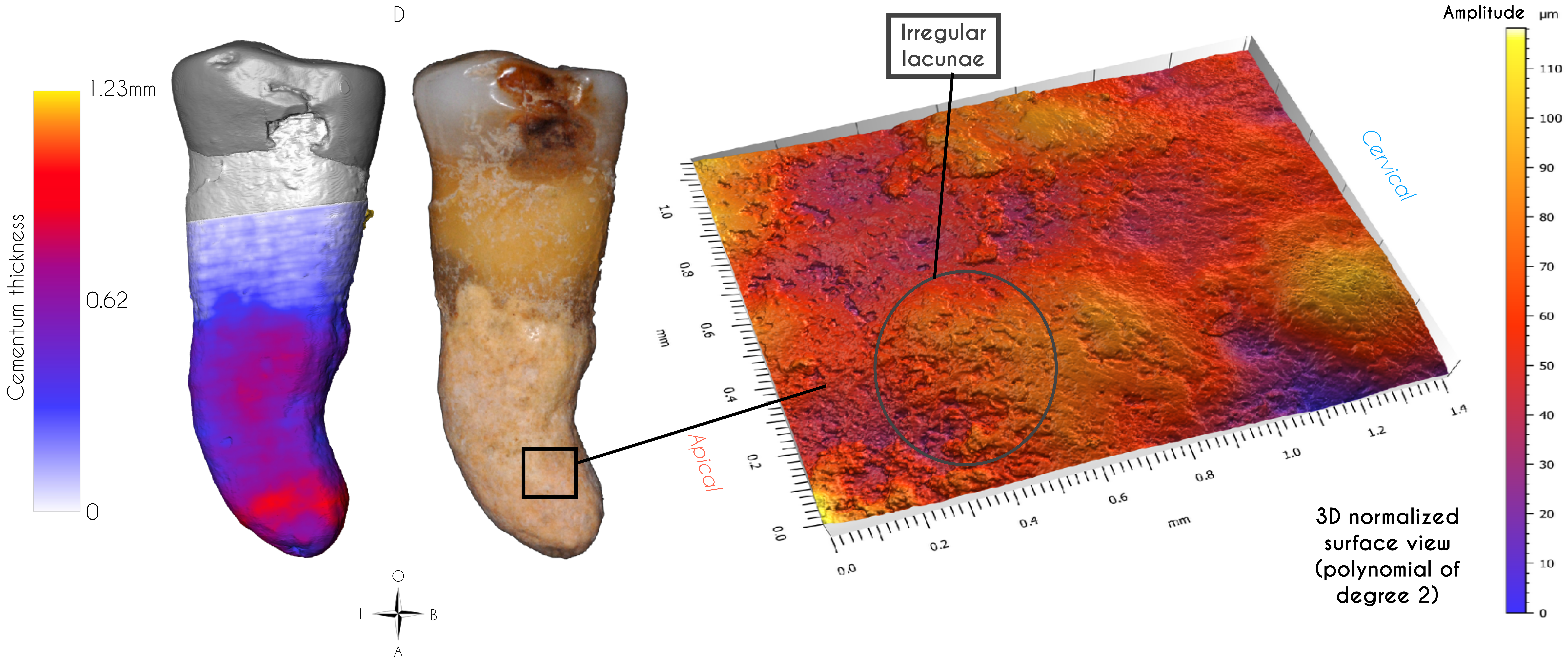

Confocal microscopy - Topography 3D maps			
OF	MAX VE (µm)	ST	Note
D	118	-S2	IL

OF: other face (1: apical root third; 2: middle root third; 3: cervical root third; m: mesial; d: distal; <: buccal; >: lingual);
MAX VE: maximum vertical elevation;
ST: surface texture; : scored (**+**) if it is high, (**-**) if it is low; **S:** smooth or **R:** rough; the frequency of occurrence of the elevations scored (**1**) if the frequency is high, (**2**) if the frequency is low.
IL: irregular lacunae

Mixed

Sp876_44

Tooth	Occlusal Wear			CAR Si/Sta	Pulp EXP	IMP	Hypercementosis	Bone Context			
	DEG	DIR	FOR					FEN	CAL	NT	ANTA
**LRP3**	**NA**	**NA**	**NA**	**1/4**	**M**	**0**	**1.4.m**	**0**	**1**	**0**	**2**

LRP3

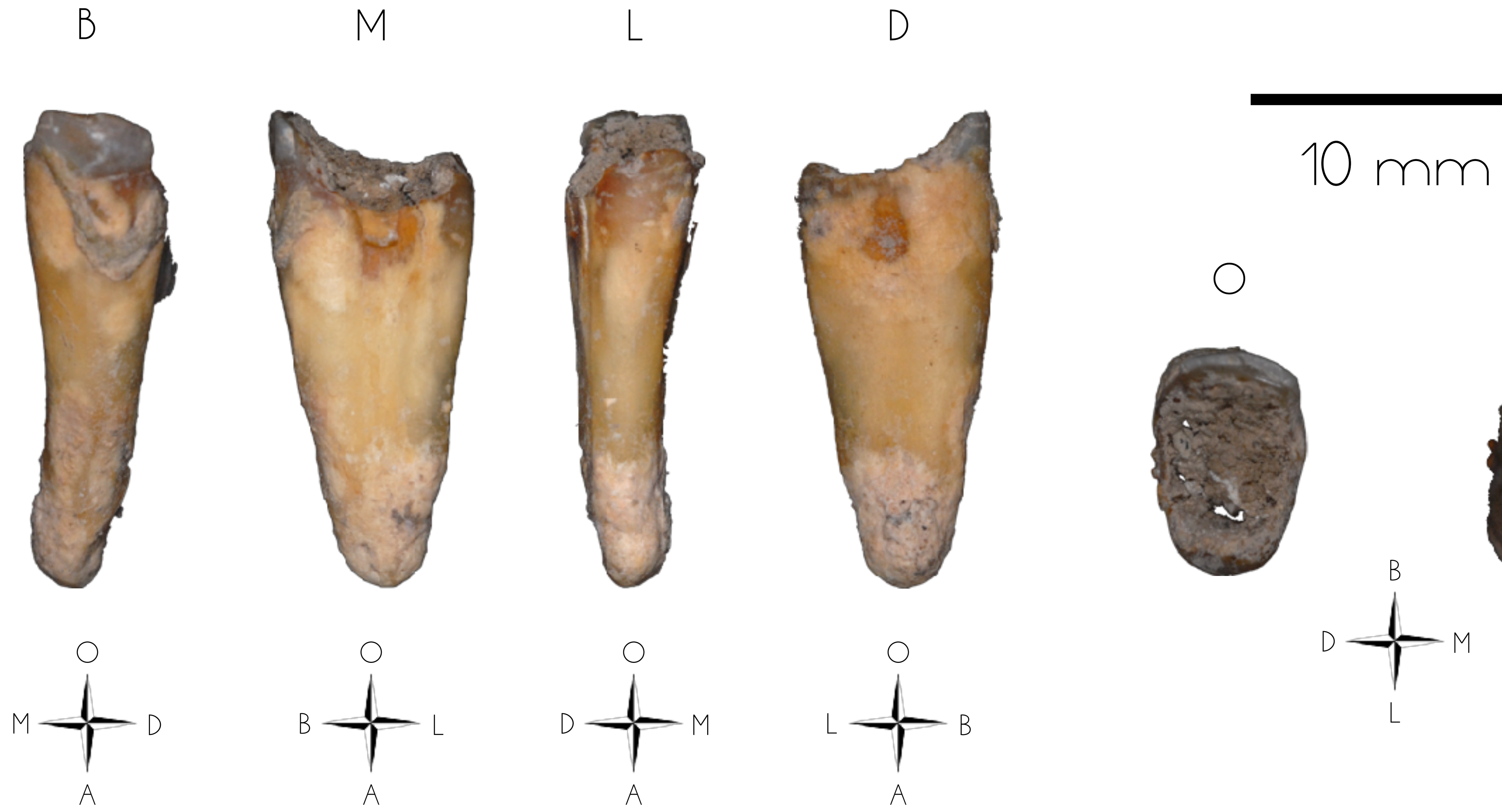

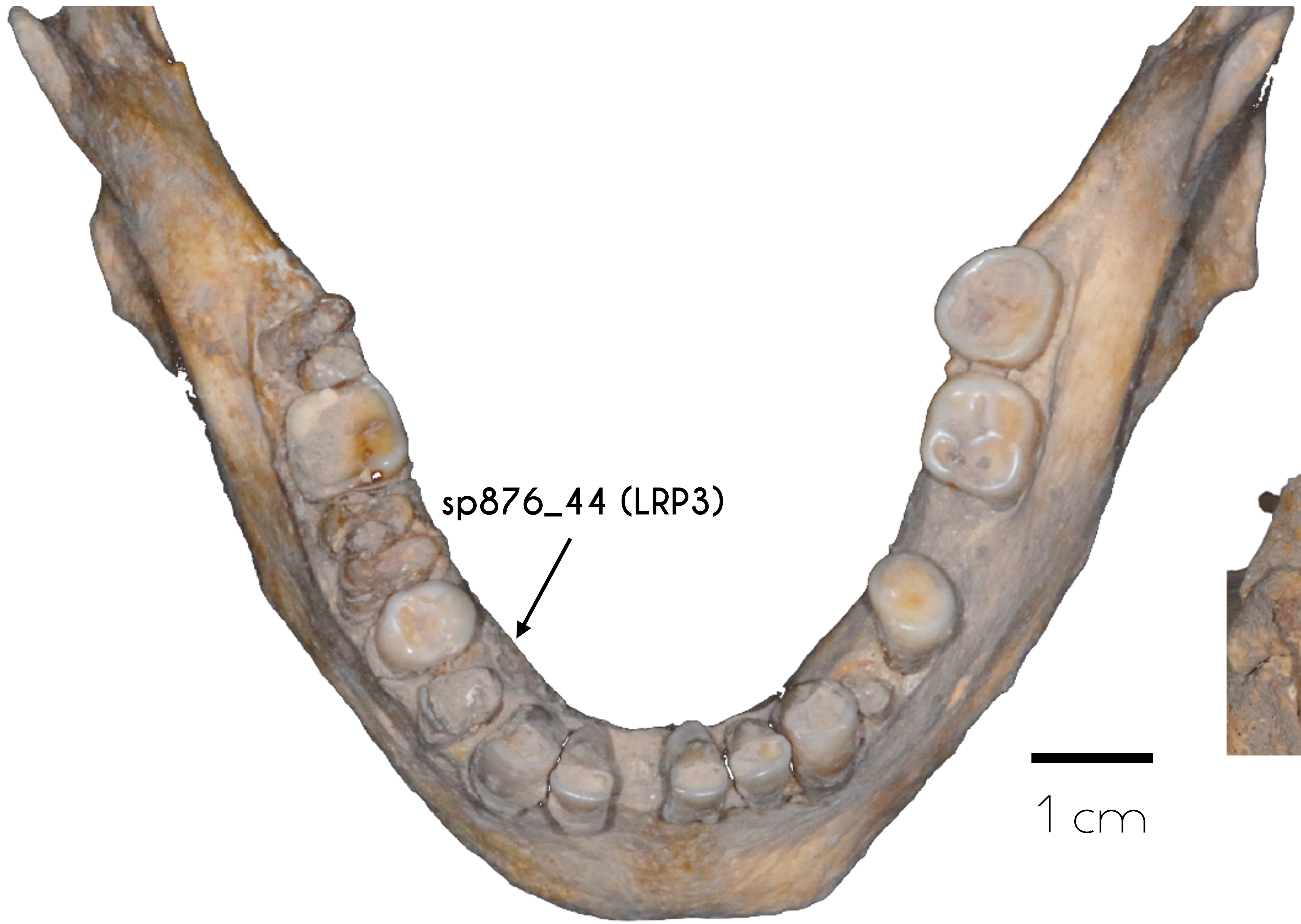

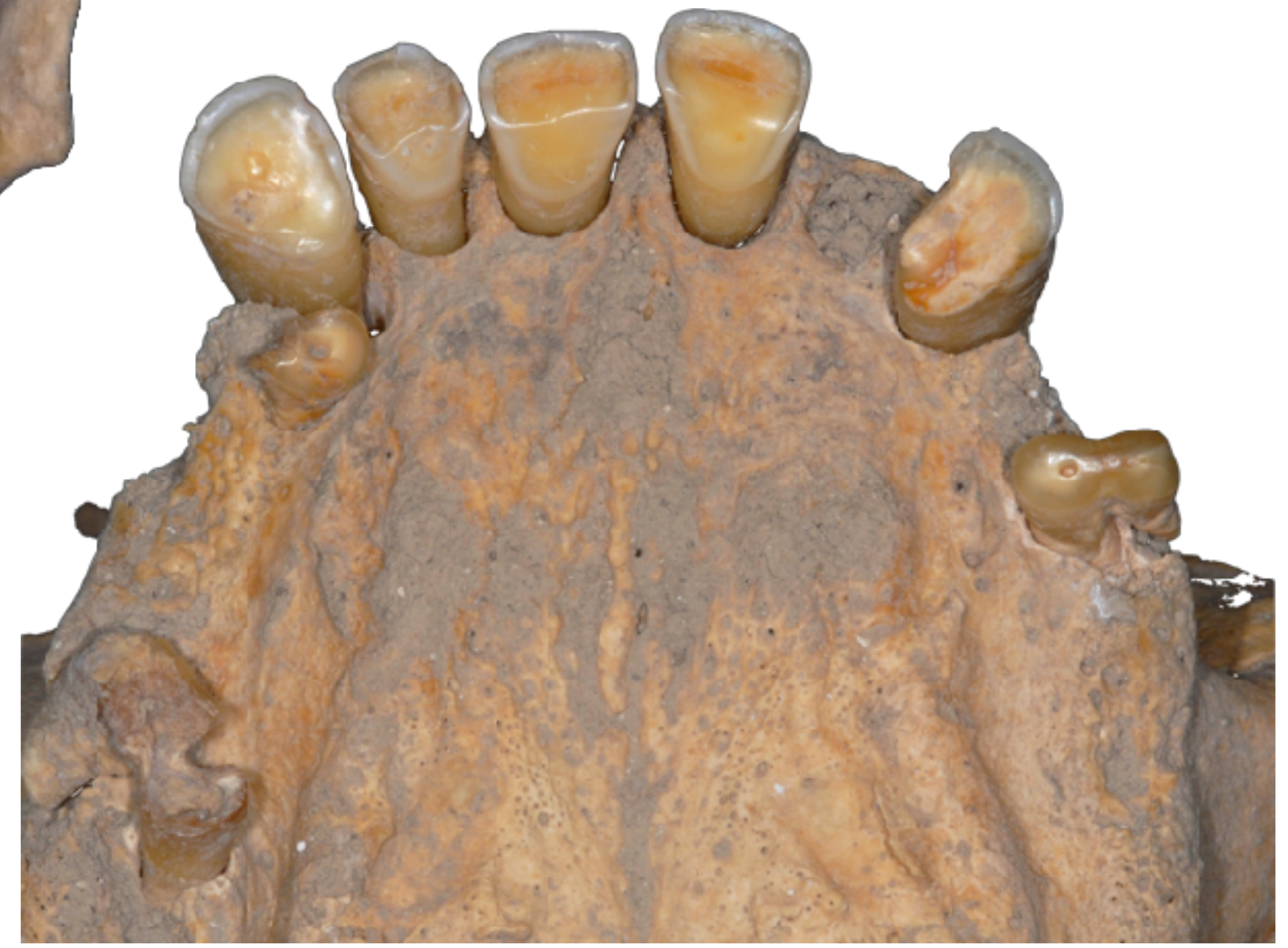

DEG: degree; **DIR:** direction; **FOR:** form - **CAR**: carious lesion - **Pulp EXP:** pulp exposure - **IMP**: impaction - **FEN:** fenestration; **CAL:** calculus; **NT:** tooth loss in neighbouring teeth; **ANTA:** antagonist

Sp876_44

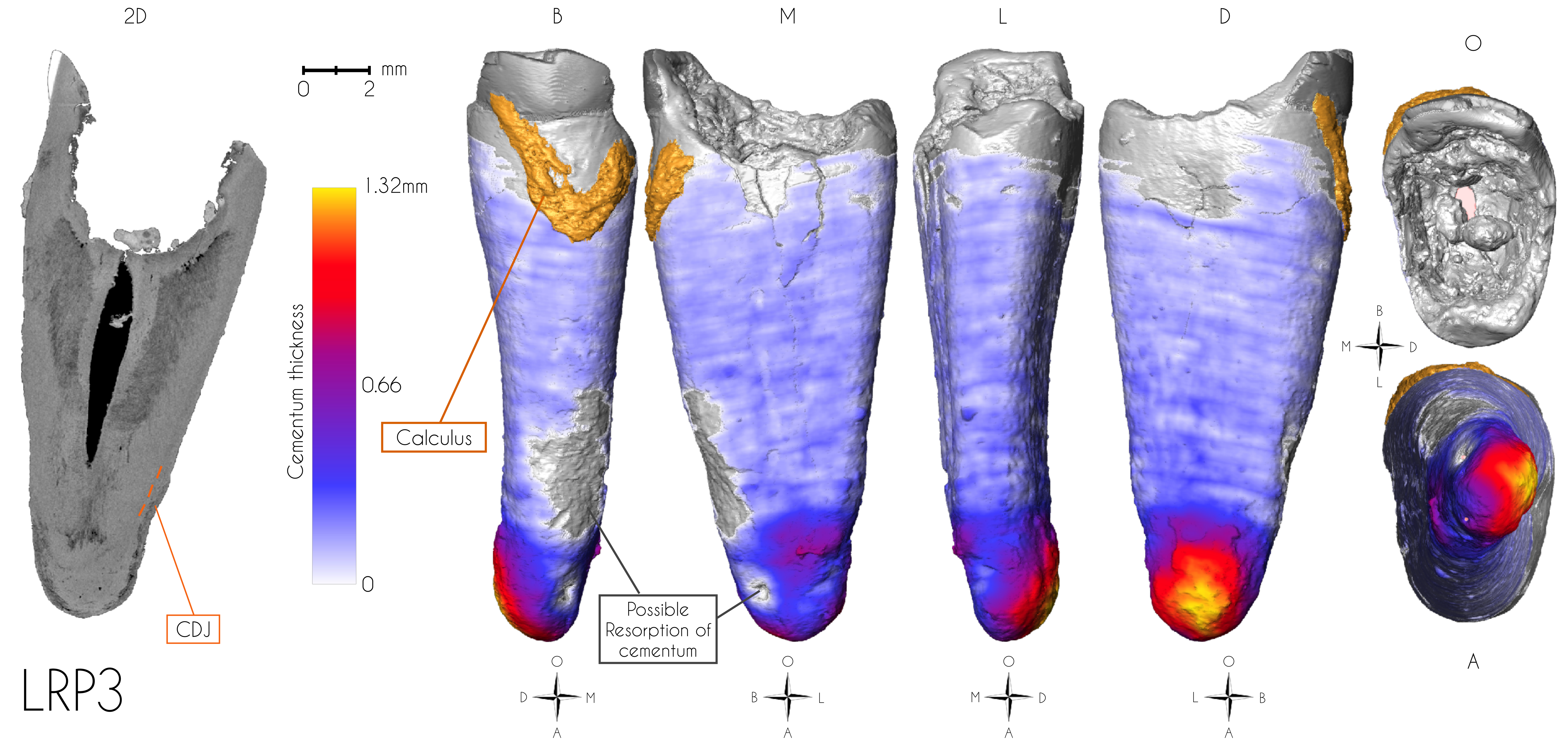

Microtomography - Thickness 3D maps				
MAX THI (μm)	LOC MAX	LOC MIN	PREF LOC	Note
1320	1d	No	Yes	RES (m<)

MAX THI: maximum thickness of cementum;
LOC MAX: location of maximum thickness;
LOC MIN: location of minimum thickness;
PREF LOC: preferential location;
RES: resorption of cementum;
(1: apical root third; 2: middle root third; 3: cervical root third; m: mesial; d: distal; <: buccal; >: lingual)

Mixed

LRP3

Face	Range	Light	Width	Height	Columns/Rows	Factor	Led Color	Gain	Gamma	Overlapping
D	945	5.48%	1.67mm	1.85mm	2/3	2	Blue	1.47	1.61	10 %

Sp876_44

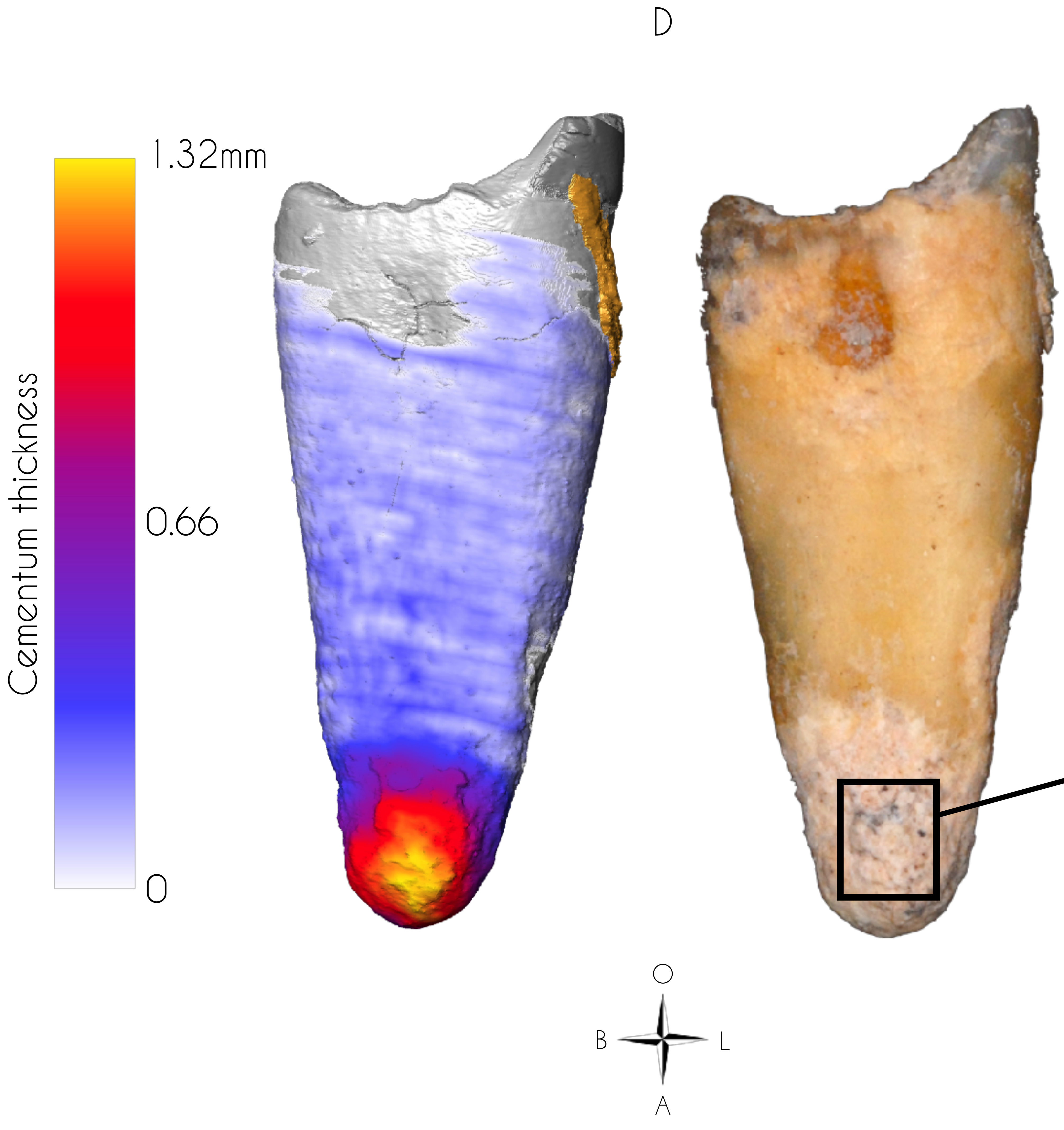

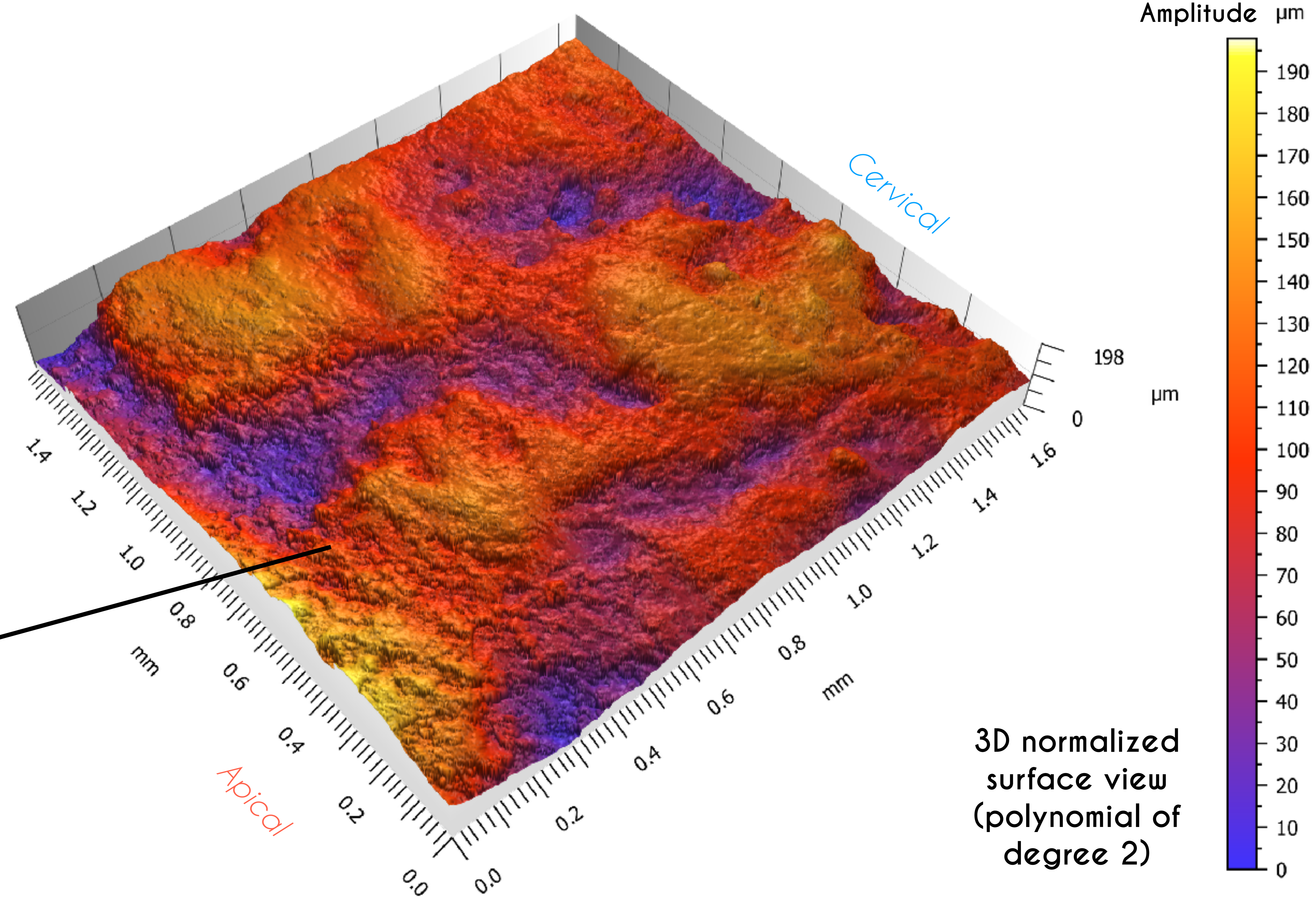

3D normalized surface view (polynomial of degree 2)

Confocal microscopy - Topography 3D maps			
LOC MAX	MAX VE (µm)	ST	Note
1d	200	+R2	-

LOC MAX: location of maximum thickness (1: apical root third; 2: middle root third; 3: cervical root third; m: mesial; d: distal; <: buccal; >: lingual);
MAX VE: maximum vertical elevation;
ST: surface texture; : scored (**+**) if it is high, (**-**) if it is low; **S:** smooth or **R:** rough; the frequency of occurrence of the elevations scored (**1**) if the frequency is high, (**2**) if the frequency is low.

Mixed

Sp876_45

Tooth	Occlusal Wear			CAR Si/Sta	Pulp EXP	IMP	Hypercementosis	Bone Context			
	DEG	DIR	FOR					FEN	CAL	NT	ANTA
4	6	3	2/2	0	0	0	1.2.M	0	1	0	2

LRP4

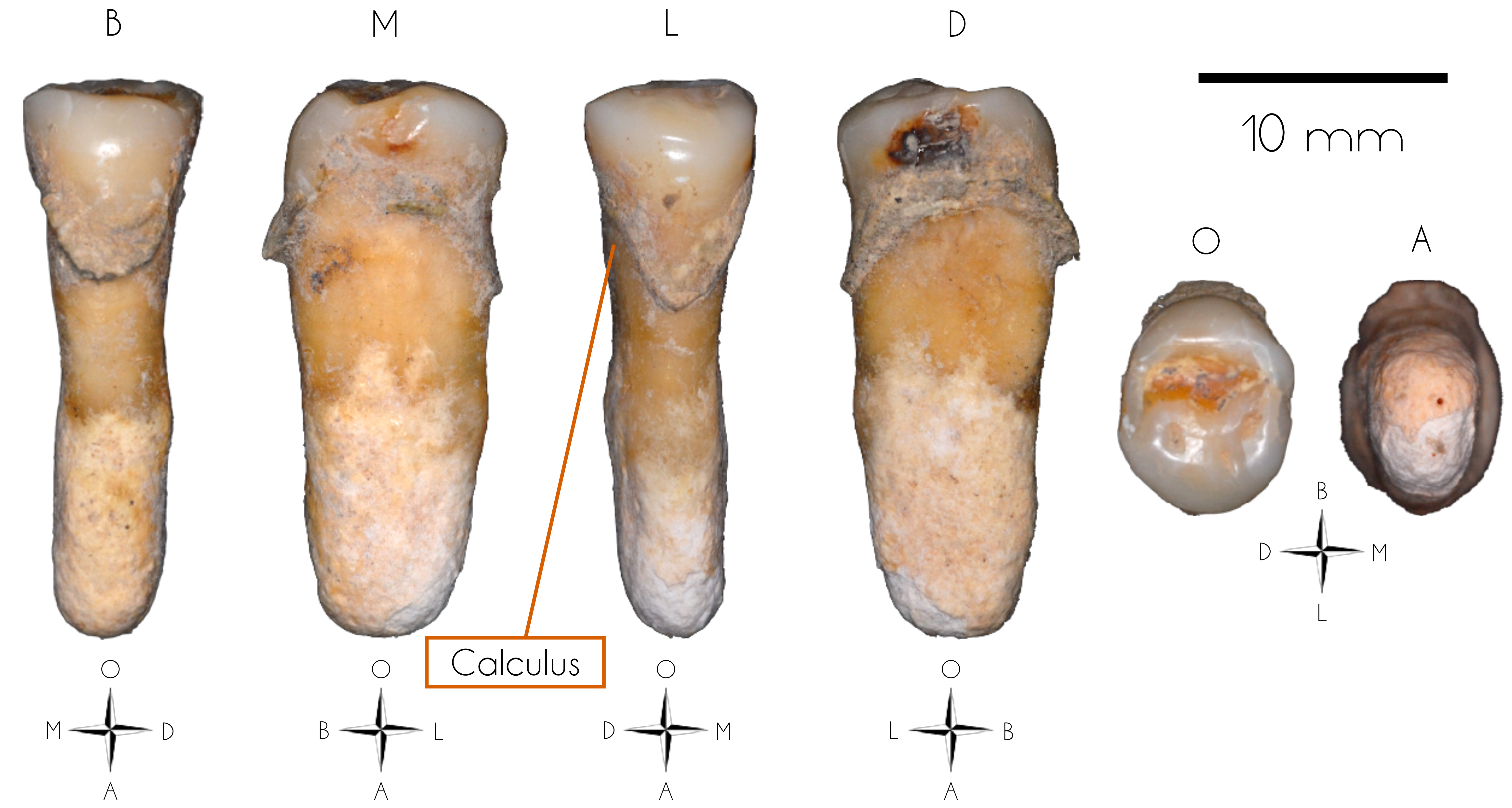

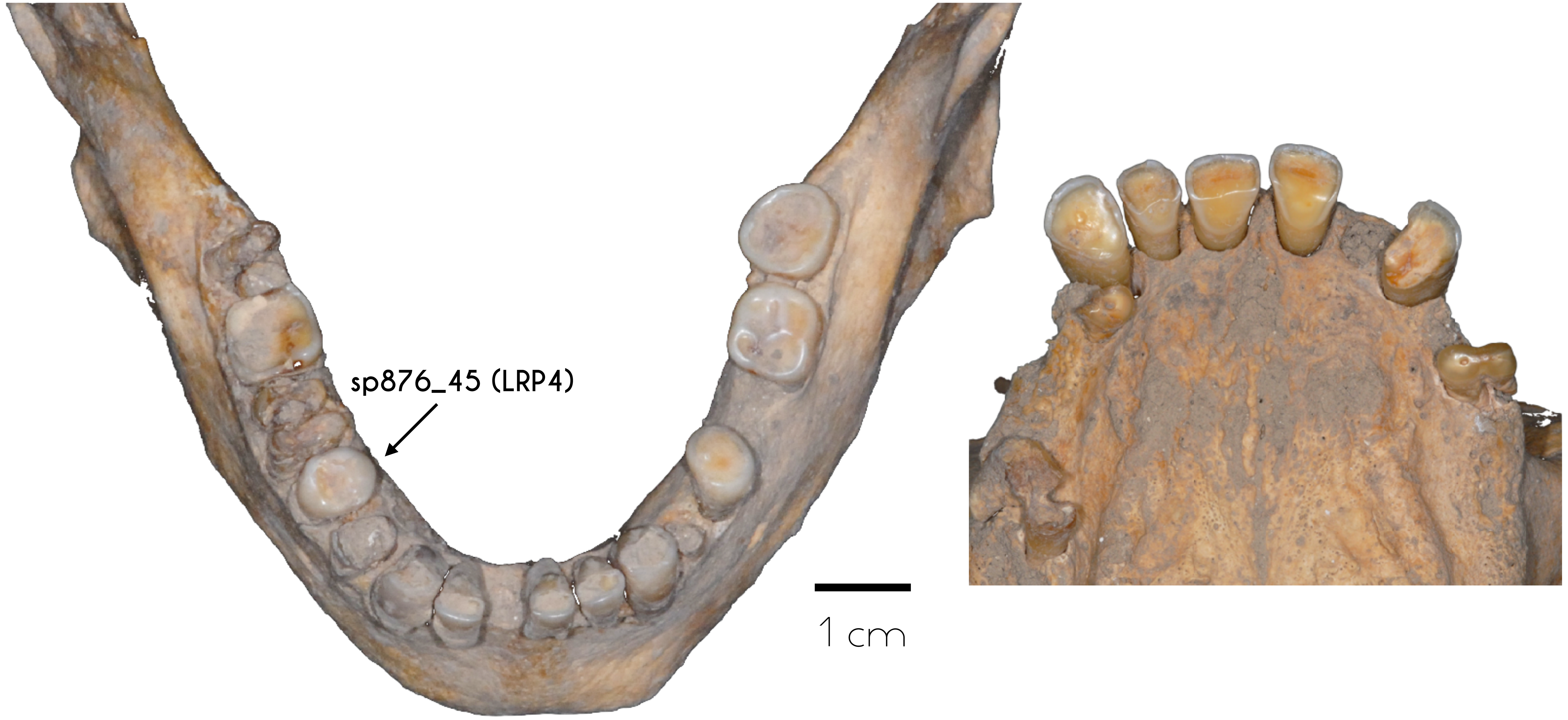

DEG: degree; **DIR:** direction; **FOR:** form - **CAR**: carious lesion - **Pulp EXP:** pulp exposure - **IMP**: impaction - **FEN:** fenestration; **CAL:** calculus; **NT:** tooth loss in neighbouring teeth; **ANTA:** antagonist

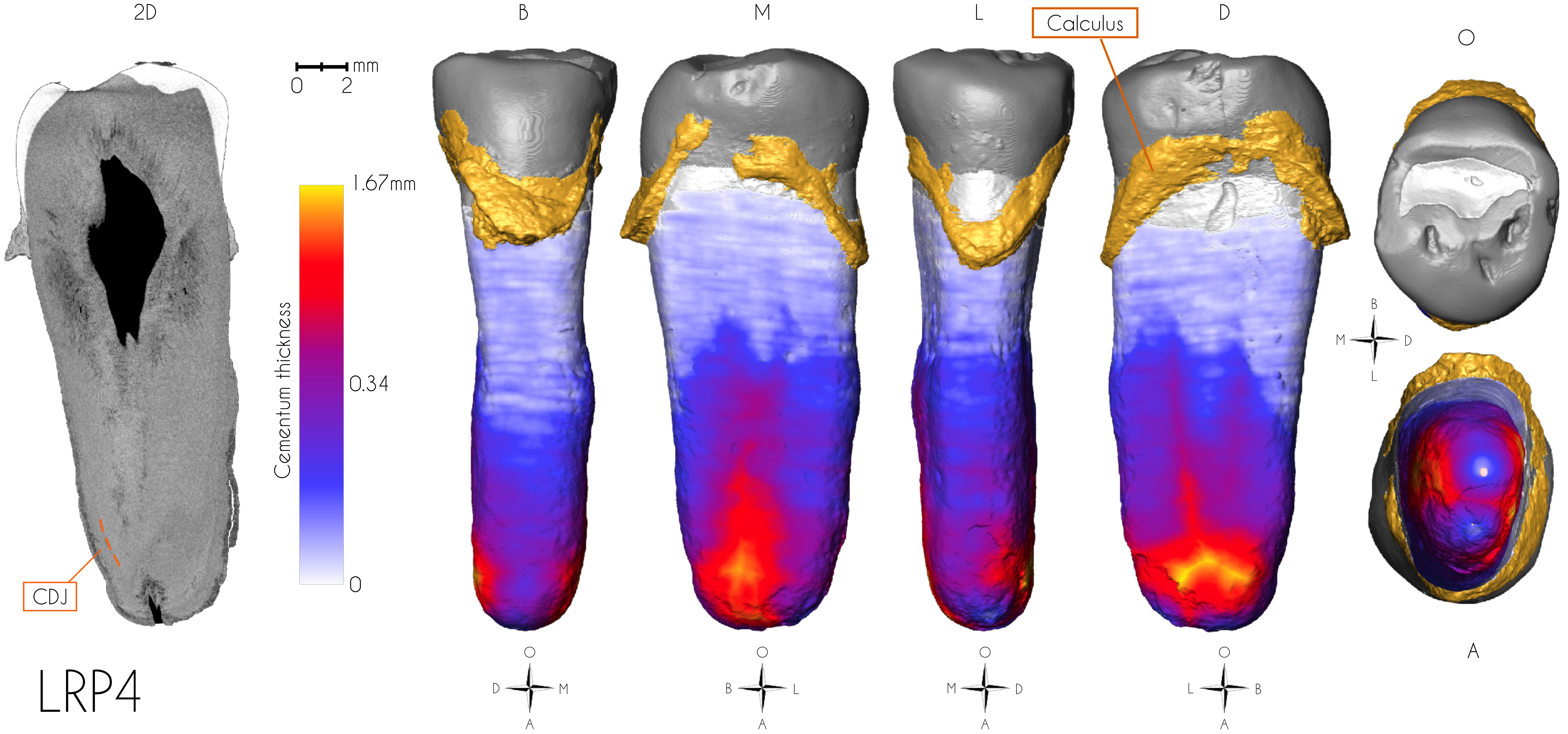

Sp876_45

Microtomography - Thickness 3D maps				
MAX THI (µm)	LOC MAX	LOC MIN	PREF LOC	Note
1670	1d	<	No	FR (1>)

MAX THI: maximum thickness of cementum;
LOC MAX: location of maximum thickness;
LOC MIN: location of minimum thickness;
PREF LOC: preferential location;
FR: fracture of cementum;
(1: apical root third; 2: middle root third; 3: cervical root third; m: mesial; d: distal; <: buccal; >: lingual)

<u>Mixed</u>

LRP4

Face	Range	Light	Width	Height	Columns/Rows	Factor	Led Color	Gain	Gamma	Overlapping
D	511	4.9%	0.88mm	4.82mm	1/8	2	Blue	1.5	1.8	10 %

Sp876_45

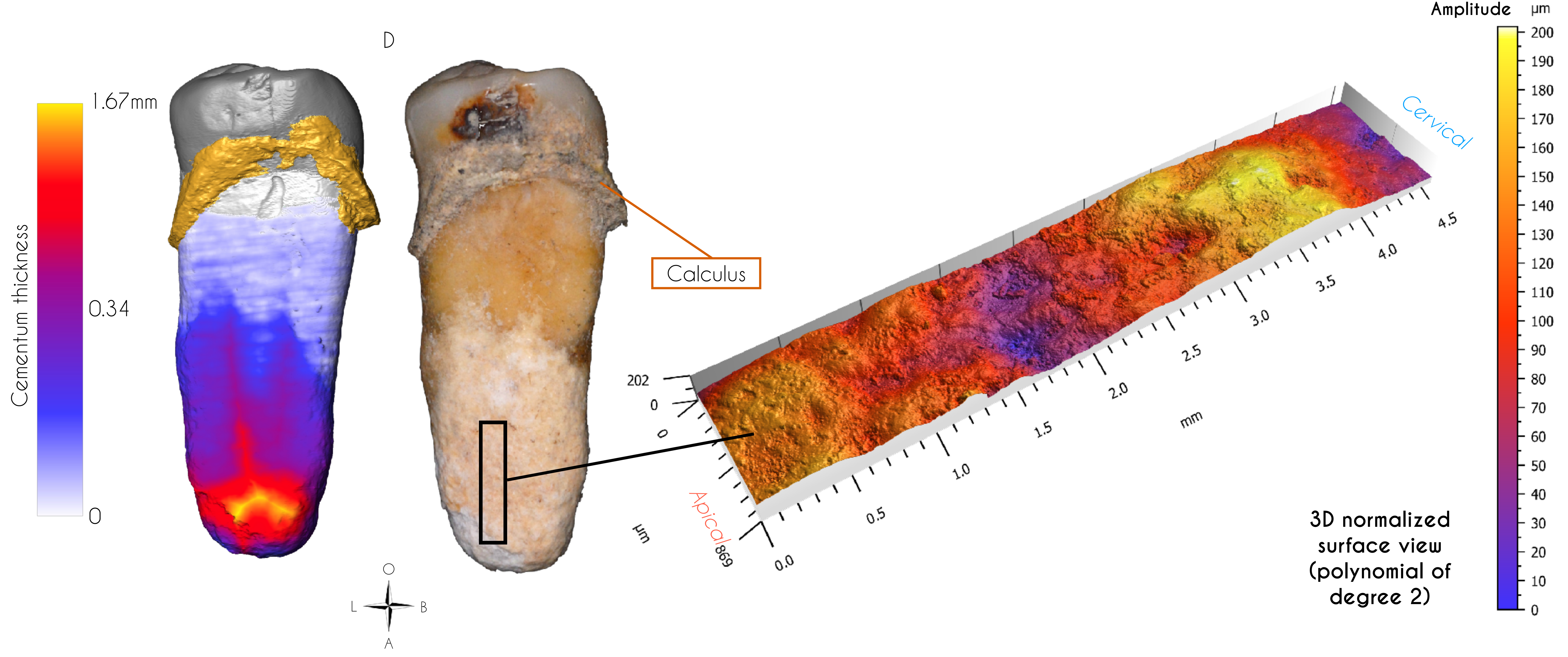

3D normalized surface view (polynomial of degree 2)

Confocal microscopy - Topography 3D maps			
LOC MAX	MAX VE (µm)	ST	Note
2d	200	+R2	-

LOC MAX: location of maximum thickness (1: apical root third; 2: middle root third; 3: cervical root third; m: mesial; d: distal; <: buccal; >: lingual);
MAX VE: maximum vertical elevation;
ST: surface texture; : scored (**+**) if it is high, (**-**) if it is low; **S:** smooth or **R:** rough; the frequency of occurrence of the elevations scored (**1**) if the frequency is high, (**2**) if the frequency is low.

Mixed

Sp884_34

Tooth	Occlusal Wear			CAR Si/Sta	Pulp EXP	IMP	Hypercementosis	Bone Context			
	DEG	DIR	FOR					FEN	CAL	NT	ANTA
**LLP3**	**8**	**3**	**6**	**-**	**0**	**0**	**3.4.M**	**0**	**0**	**1m**	**1**

LLP3

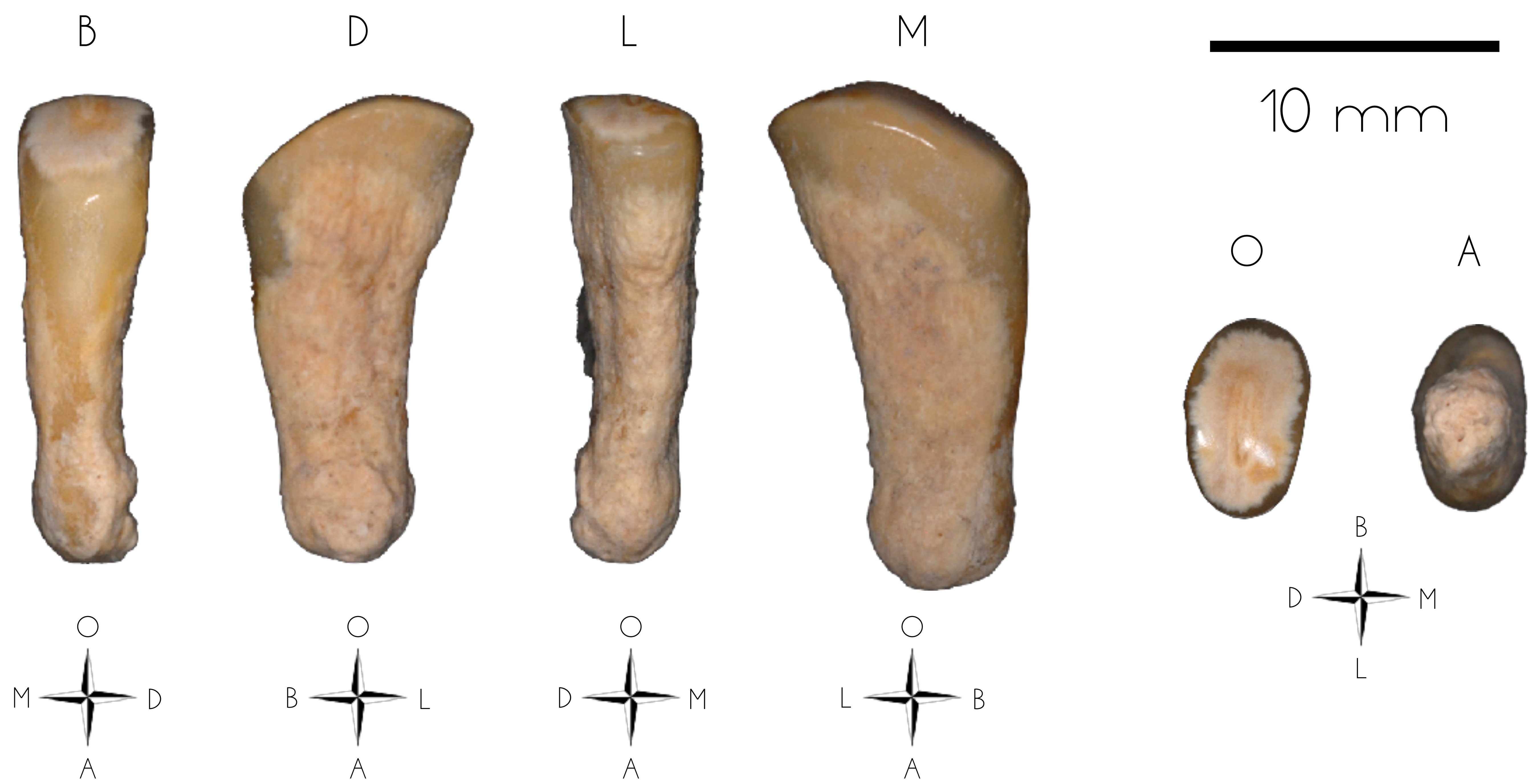

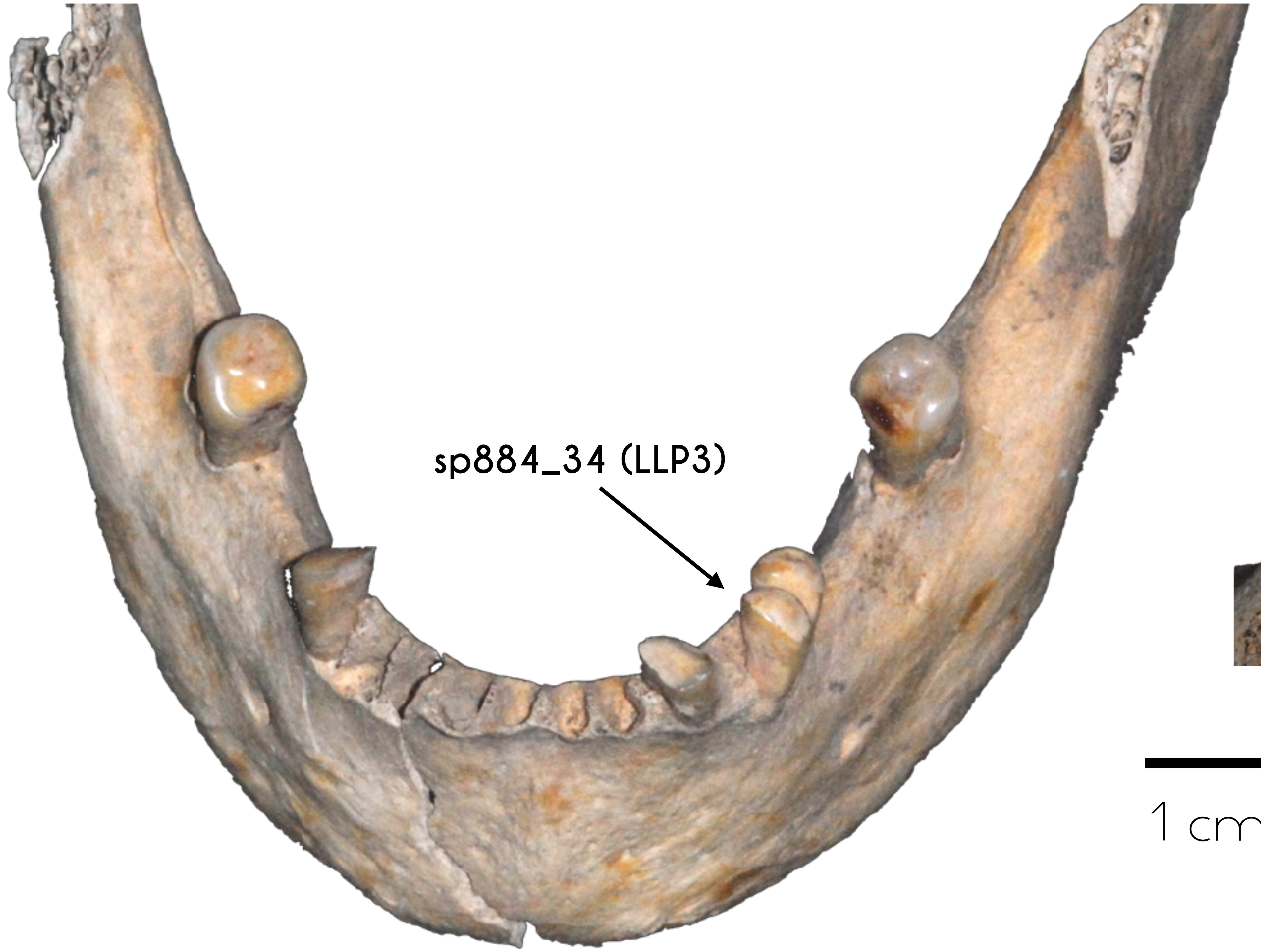

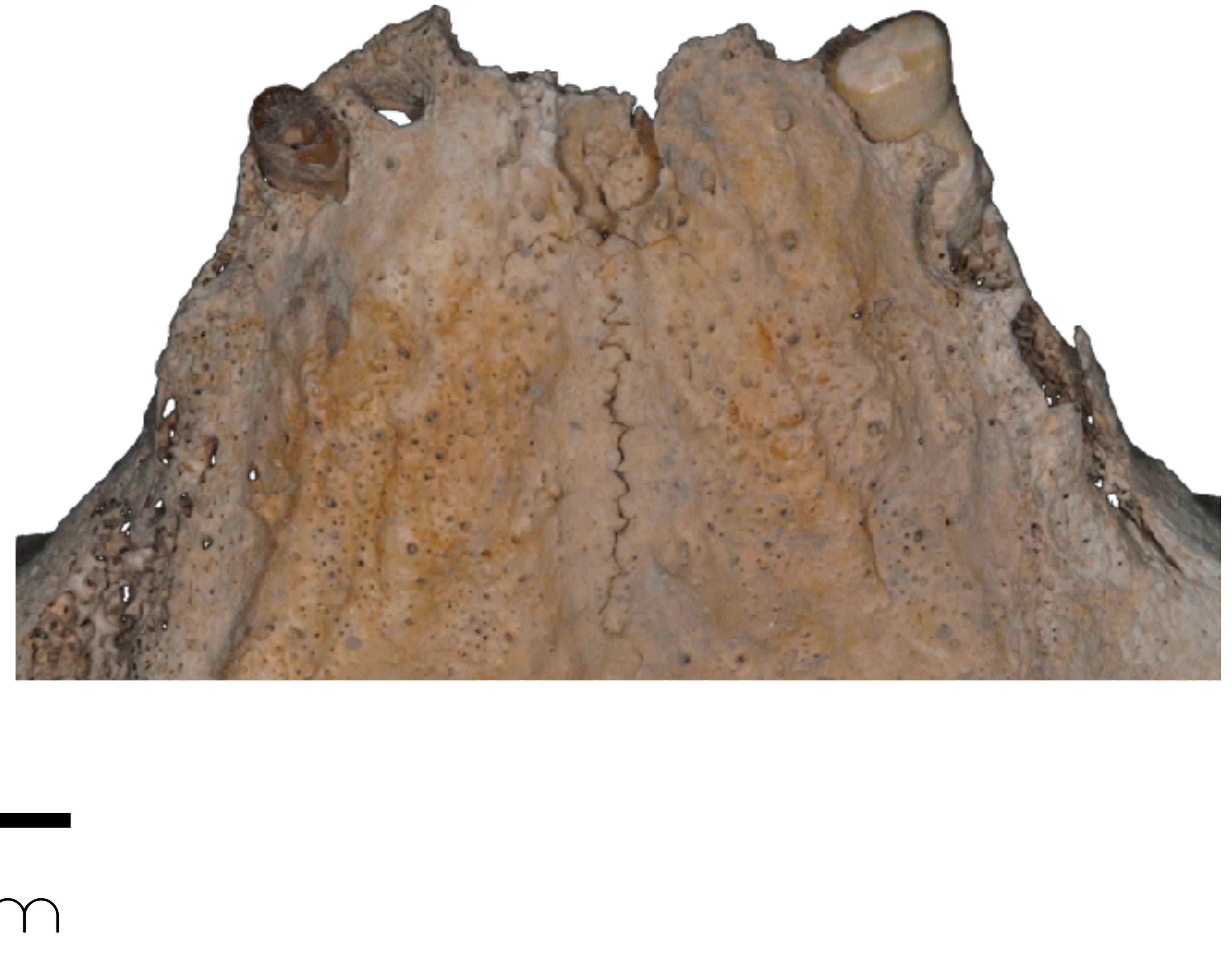

DEG: degree; **DIR:** direction; **FOR:** form - **CAR**: carious lesion - **Pulp EXP:** pulp exposure - **IMP**: impaction - **FEN:** fenestration; **CAL:** calculus; **NT:** tooth loss in neighbouring teeth; **ANTA:** antagonist

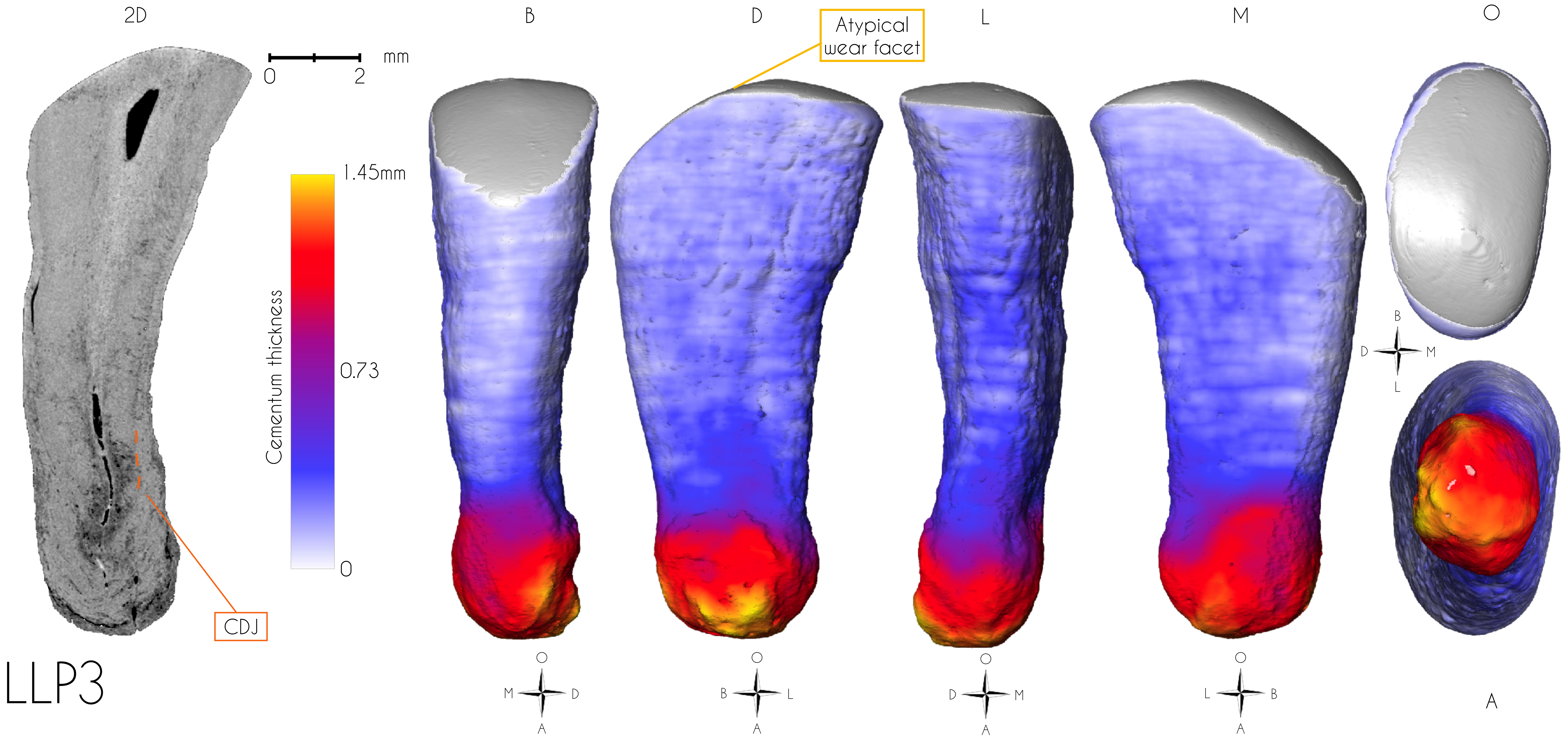

Sp884_34

Microtomography - Thickness 3D maps				
MAX THI (µm)	LOC MAX	LOC MIN	PREF LOC	Note
1450	d	No	No	W

MAX THI: maximum thickness of cementum;
LOC MAX: location of maximum thickness;
LOC MIN: location of minimum thickness;
PREF LOC: preferential location;
W: atypical wear facet;
(m: mesial; d: distal)

Mixed

LLP3

Face	Range	Light	Width	Height	Columns/Rows	Factor	Led Color	Gain	Gamma	Overlapping
D	1188	5.50%	1.67mm	1.85mm	2/3	2	Blue	1.47	1.61	10 %

Sp884_34

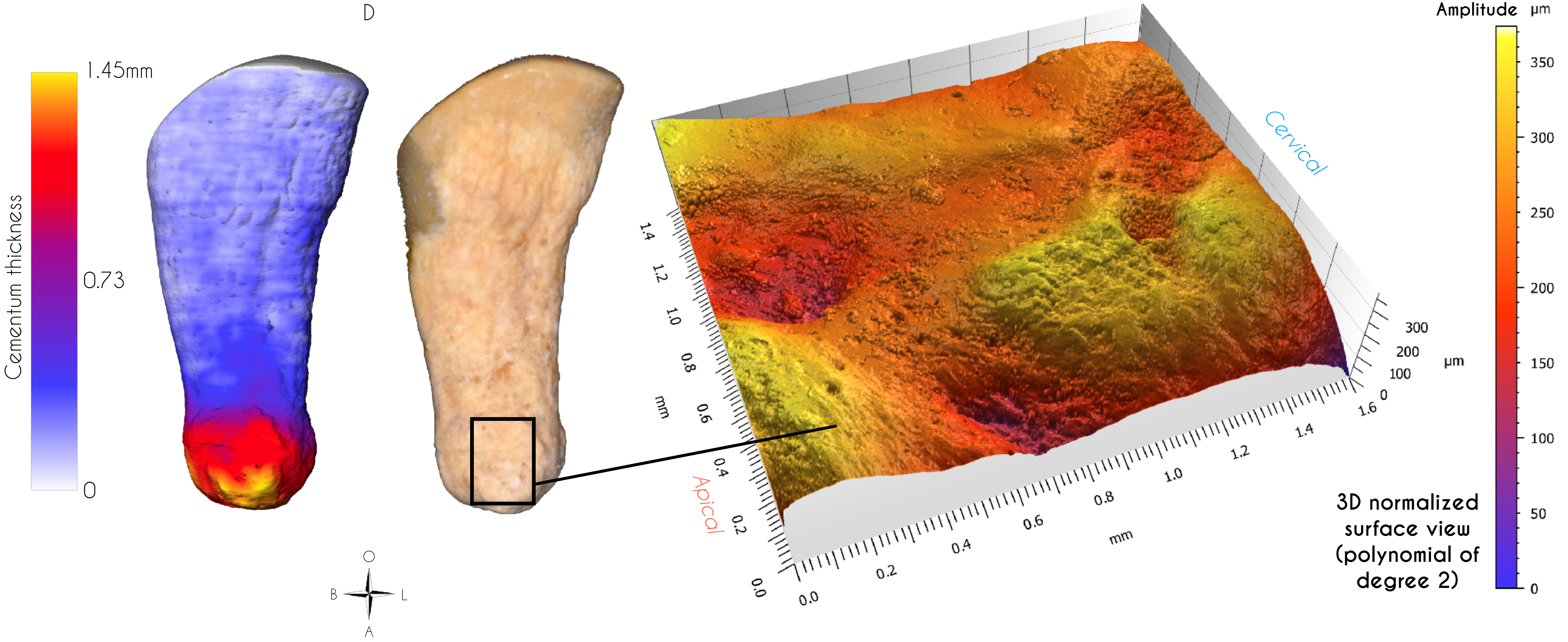

Confocal microscopy - Topography 3D maps			
LOC MAX	MAX VE (µm)	ST	Note
1d	370	+R2	-

LOC MAX: location of maximum thickness (1: apical root third; 2: middle root third; 3: cervical root third; m: mesial; d: distal; <: buccal; >: lingual);
MAX VE: maximum vertical elevation;
ST: surface texture; : scored (**+**) if it is high, (**-**) if it is low; **S:** smooth or **R:** rough; the frequency of occurrence of the elevations scored (**1**) if the frequency is high, (**2**) if the frequency is low.

Mixed

Sp884_35

Tooth	Occlusal Wear			CAR Si/Sta	Pulp EXP	IMP	Hypercementosis	Bone Context			
	DEG	DIR	FOR					FEN	CAL	NT	ANTA
**LLP4**	**8**	**7**	**6**	**-**	**W**	**0**	**3.4.M**	**0**	**0**	**1d**	**2**

LLP4

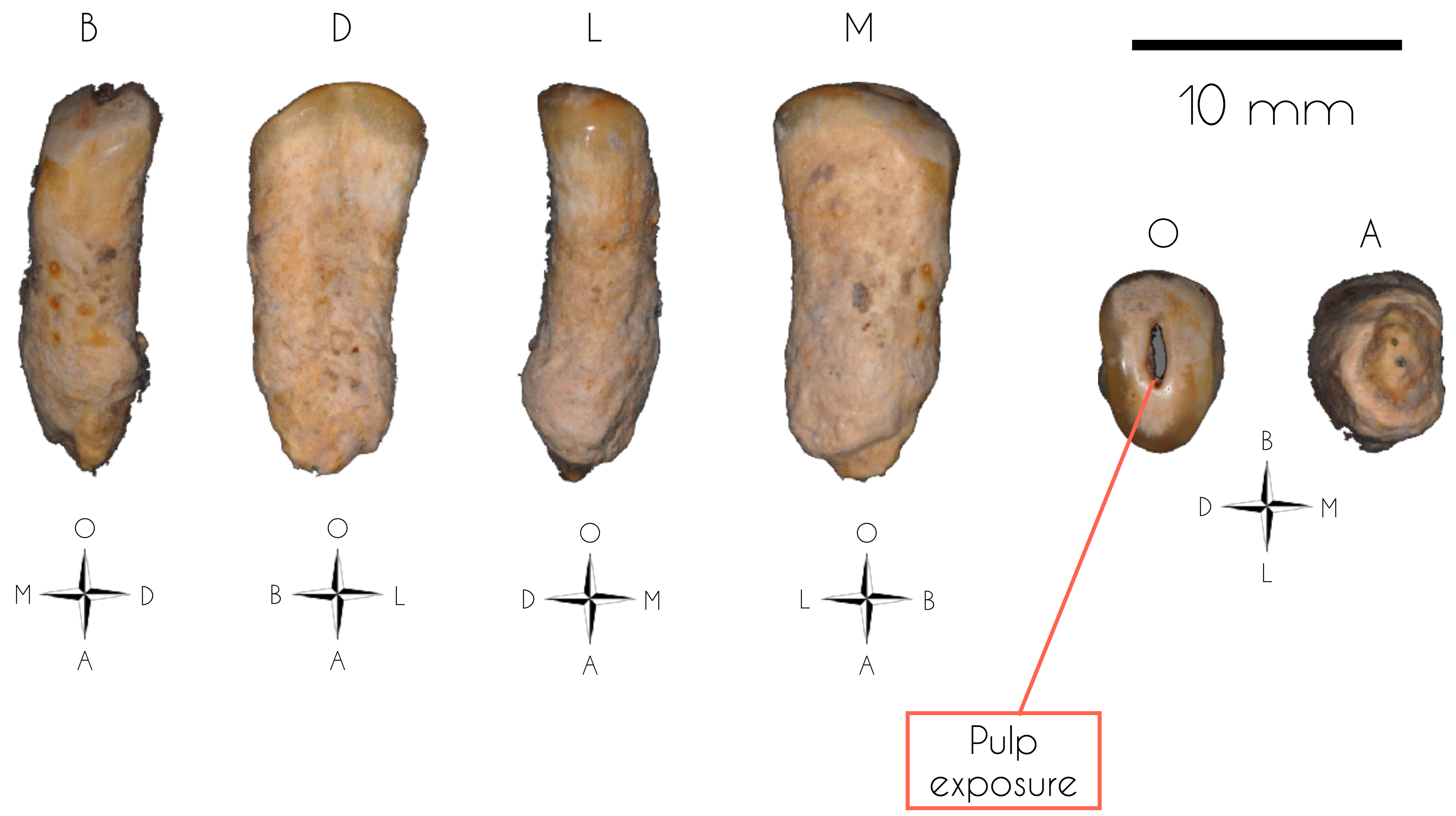

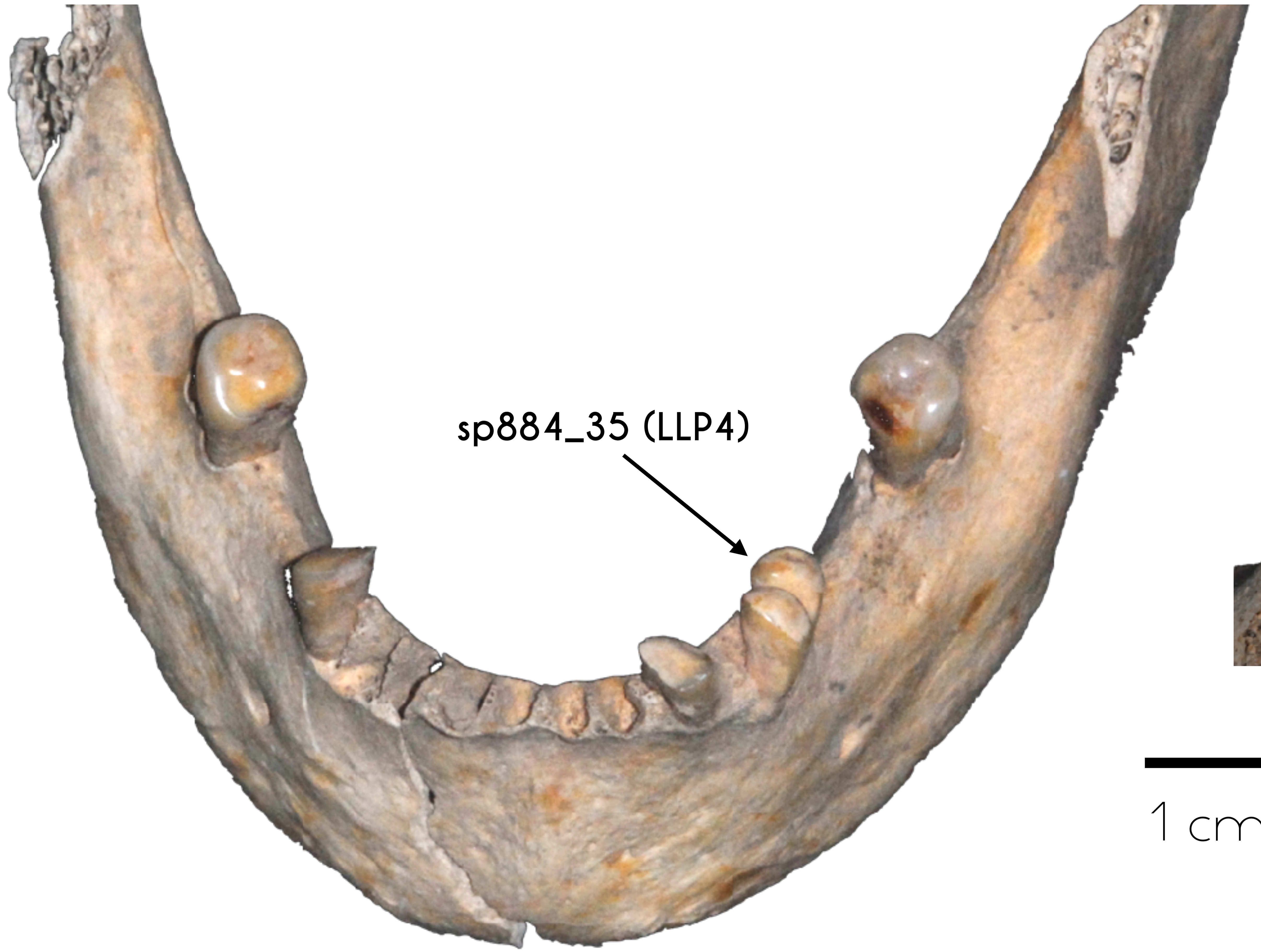

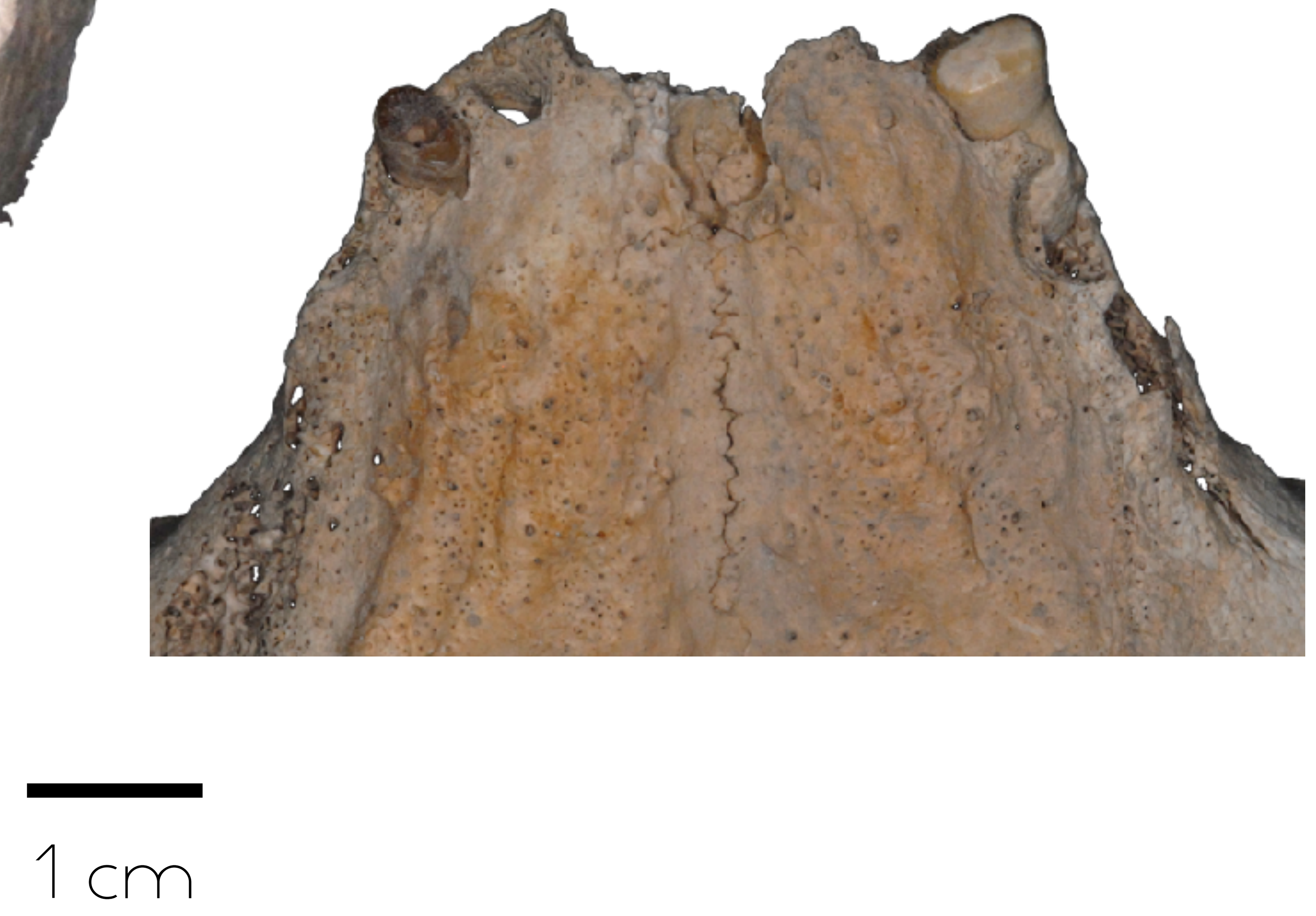

DEG: degree; **DIR:** direction; **FOR:** form - **CAR**: carious lesion - **Pulp EXP:** pulp exposure (**W:** wear) - **IMP**: impaction - **FEN:** fenestration; **CAL:** calculus; **NT:** tooth loss in neighbouring teeth; **ANTA:** antagonist

Sp884_35

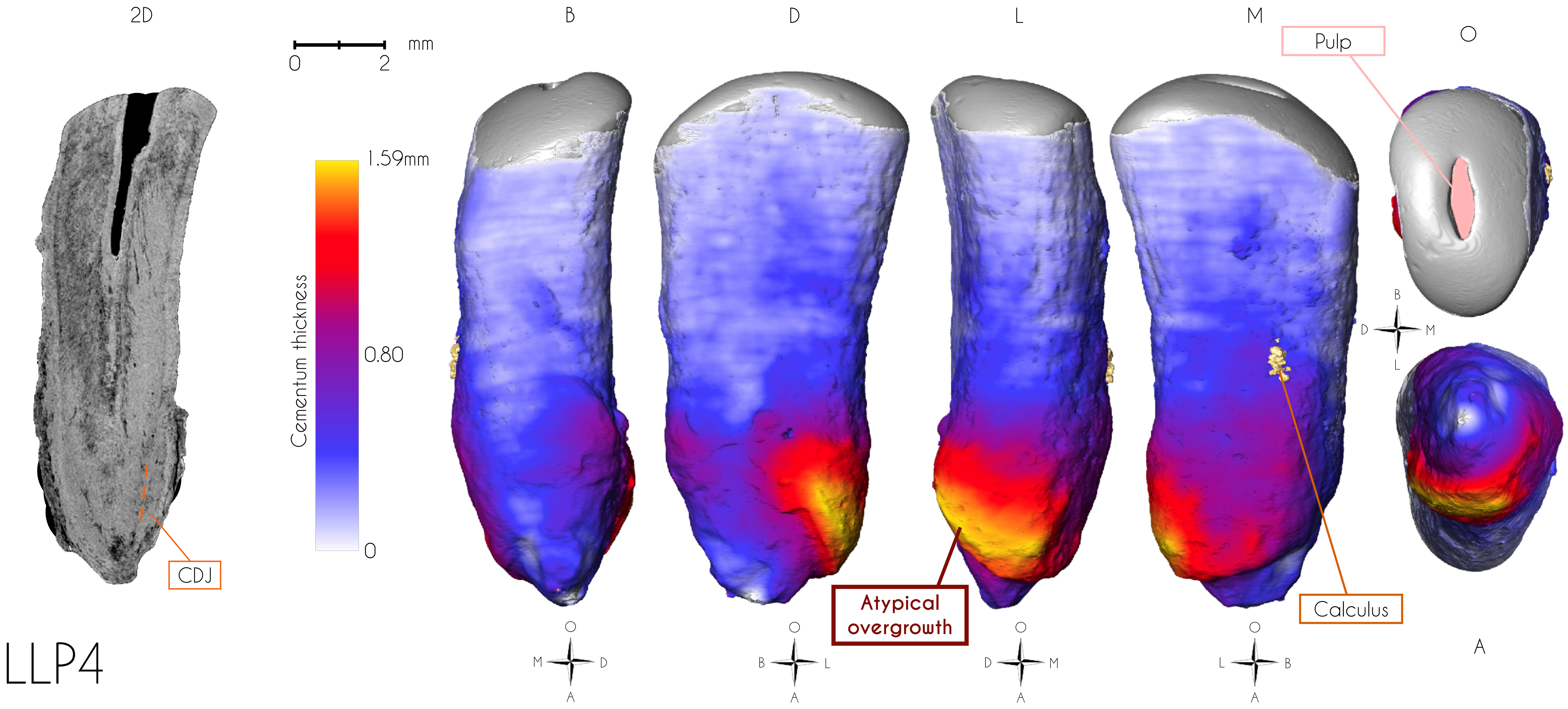

Microtomography – Thickness 3D maps				
MAX THI (µm)	LOC MAX	LOC MIN	PREF LOC	Note
1590	d>	<	Yes	OG (>)

MAX THI: maximum thickness of cementum;
LOC MAX: location of maximum thickness;
LOC MIN: location of minimum thickness;
PREF LOC: preferential location;
OG: atypical overgrowth;
(m: mesial; d: distal; <: buccal; >: lingual)

Mixed

LLP4

Face	Range	Light	Width	Height	Columns/Rows	Factor	Led Color	Gain	Gamma	Overlapping
L	1201	6.02%	2.45mm	1.85mm	3/3	2	Blue	1.47	1.61	10 %

Sp884_35

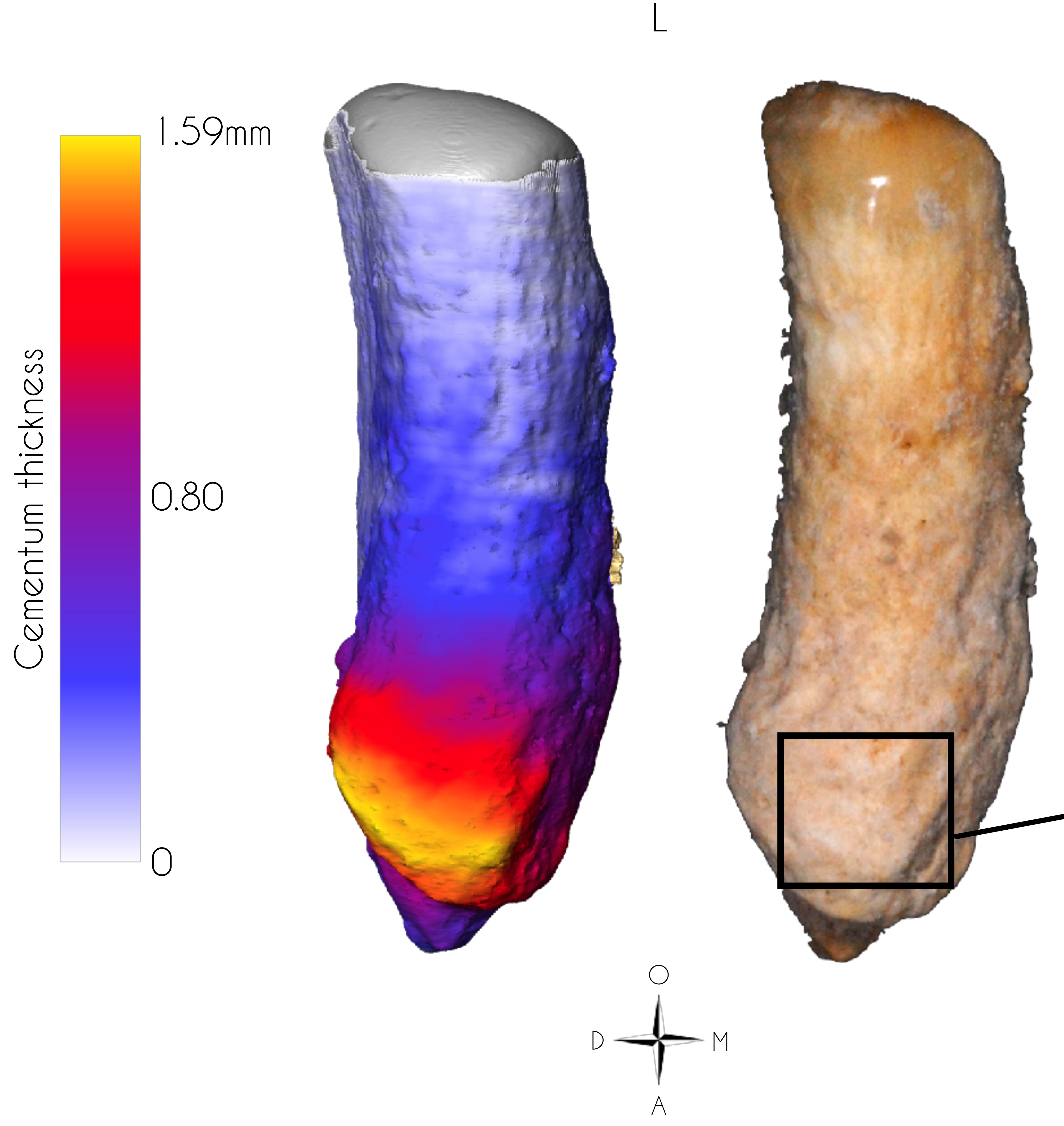

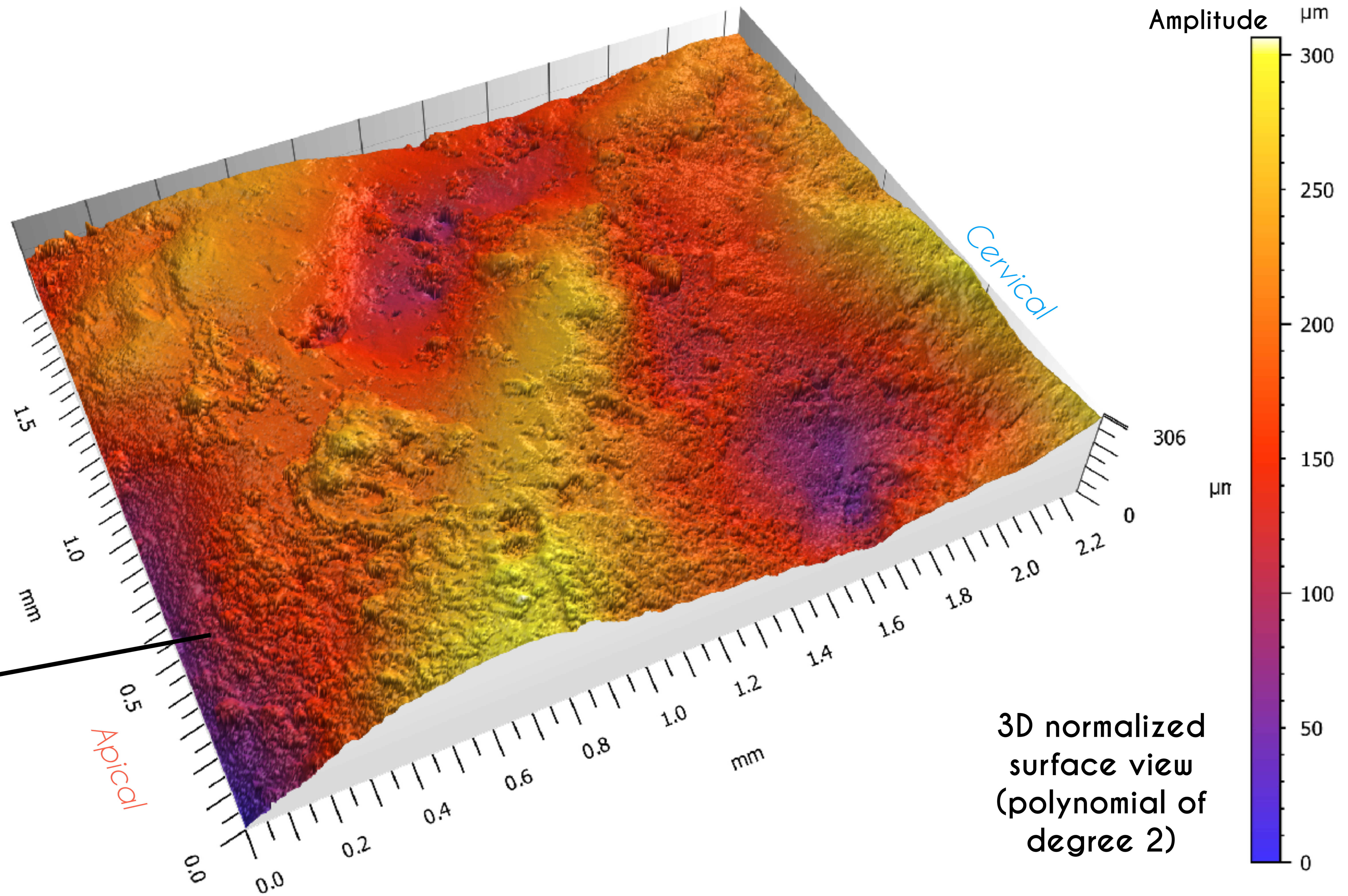

3D normalized surface view (polynomial of degree 2)

Confocal microscopy - Topography 3D maps			
LOC MAX	MAX VE (µm)	ST	Note
>	310	-R2	-

LOC MAX: location of maximum thickness (1: apical root third; 2: middle root third; 3: cervical root third; m: mesial; d: distal; <: buccal; >: lingual);
MAX VE: maximum vertical elevation;
ST: surface texture; : scored **(+)** if it is high, **(-)** if it is low; **S:** smooth or **R:** rough; the frequency of occurrence of the elevations scored (**1**) if the frequency is high, (**2**) if the frequency is low.

Mixed

Sp914_35

Tooth	Occlusal Wear			CAR Si/Sta	Pulp EXP	IMP	Hypercementosis	Bone Context			
	DEG	DIR	FOR					FEN	CAL	NT	ANTA
**LLP4**	**3**	**3**	**3**	**2/3**	**0**	**0**	**1.2.m**	**0**	**0**	**0**	**NA**

LLP4

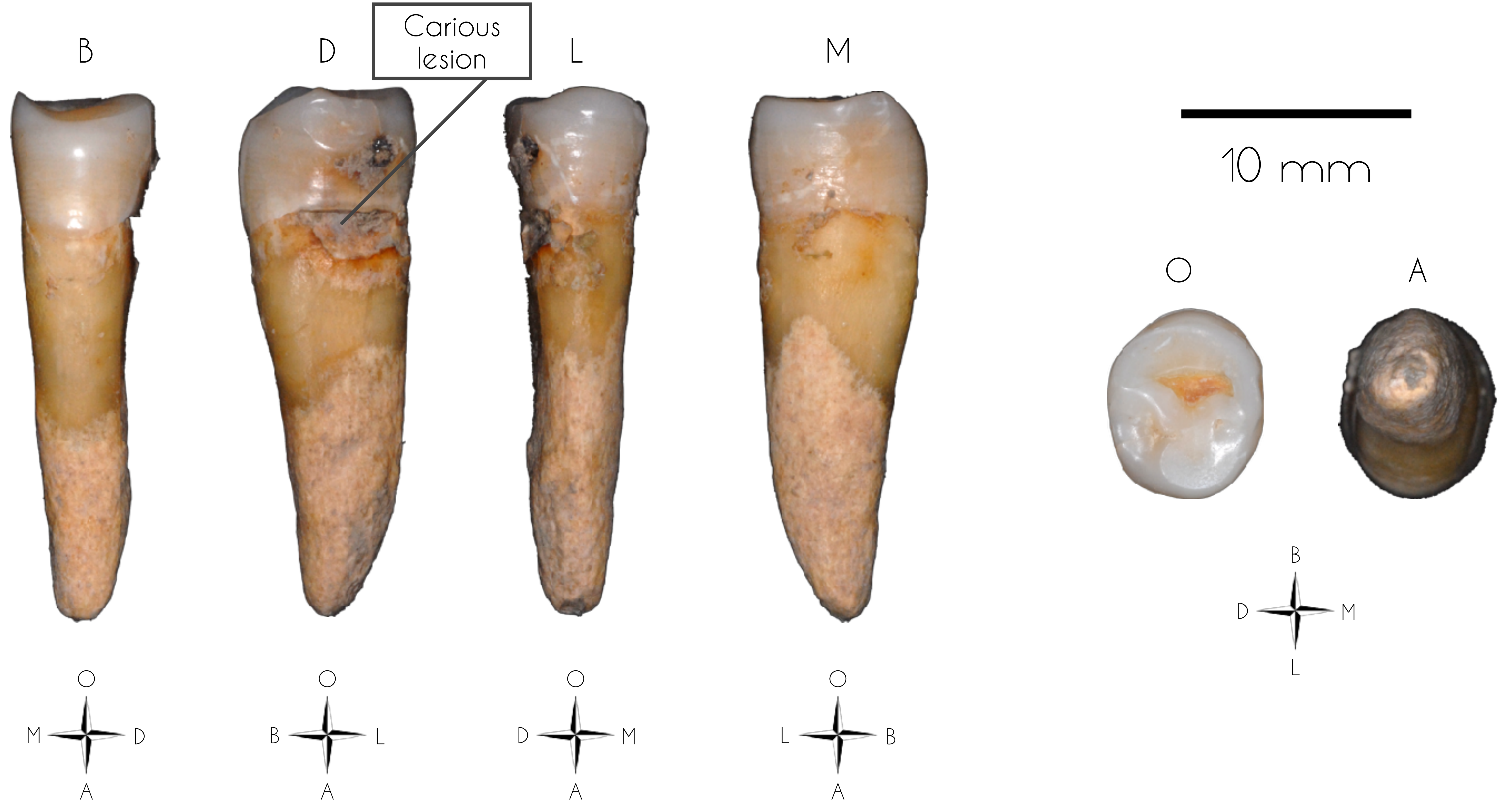

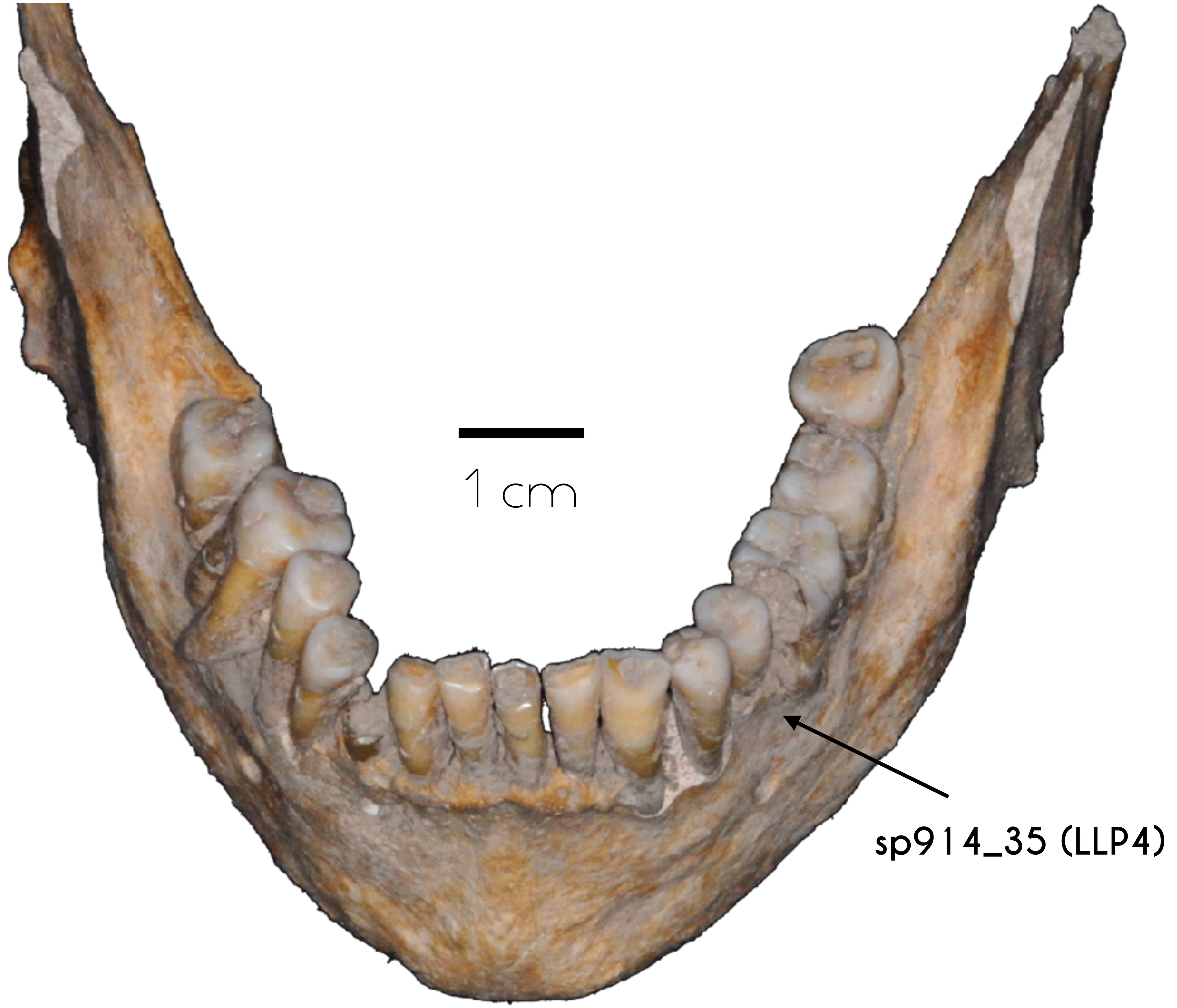

DEG: degree; **DIR:** direction; **FOR:** form - **CAR**: carious lesion - **Pulp EXP:** pulp exposure - **IMP**: impaction - **FEN:** fenestration; **CAL:** calculus; **NT:** tooth loss in neighbouring teeth; **ANTA:** antagonist

Sp914_35

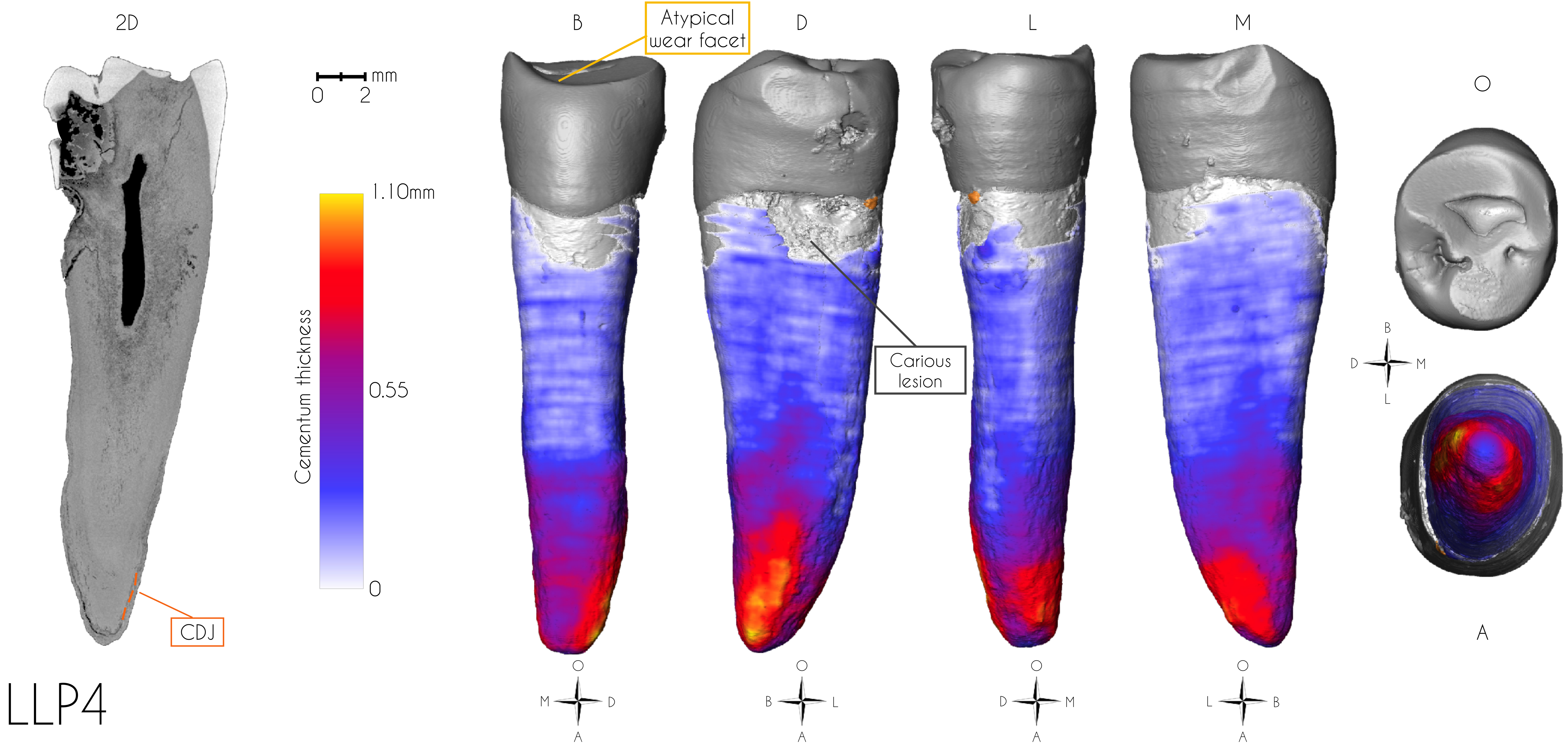

Microtomography - Thickness 3D maps				
MAX THI (μm)	LOC MAX	LOC MIN	PREF LOC	Note
1100	1d	No	No	CAR (d)

MAX THI: maximum thickness of cementum;
LOC MAX: location of maximum thickness;
LOC MIN: location of minimum thickness;
PREF LOC: preferential location;
CAR: carious lesion;
(m: mesial; d: distal)

Mixed

LLP4

Face	Range	Light	Width	Height	Columns/Rows	Factor	Led Color	Gain	Gamma	Overlapping
D	1788	5.42%	1.67mm	4.82mm	2/8	2	Blue	1.47	1.61	10 %

Sp914_35

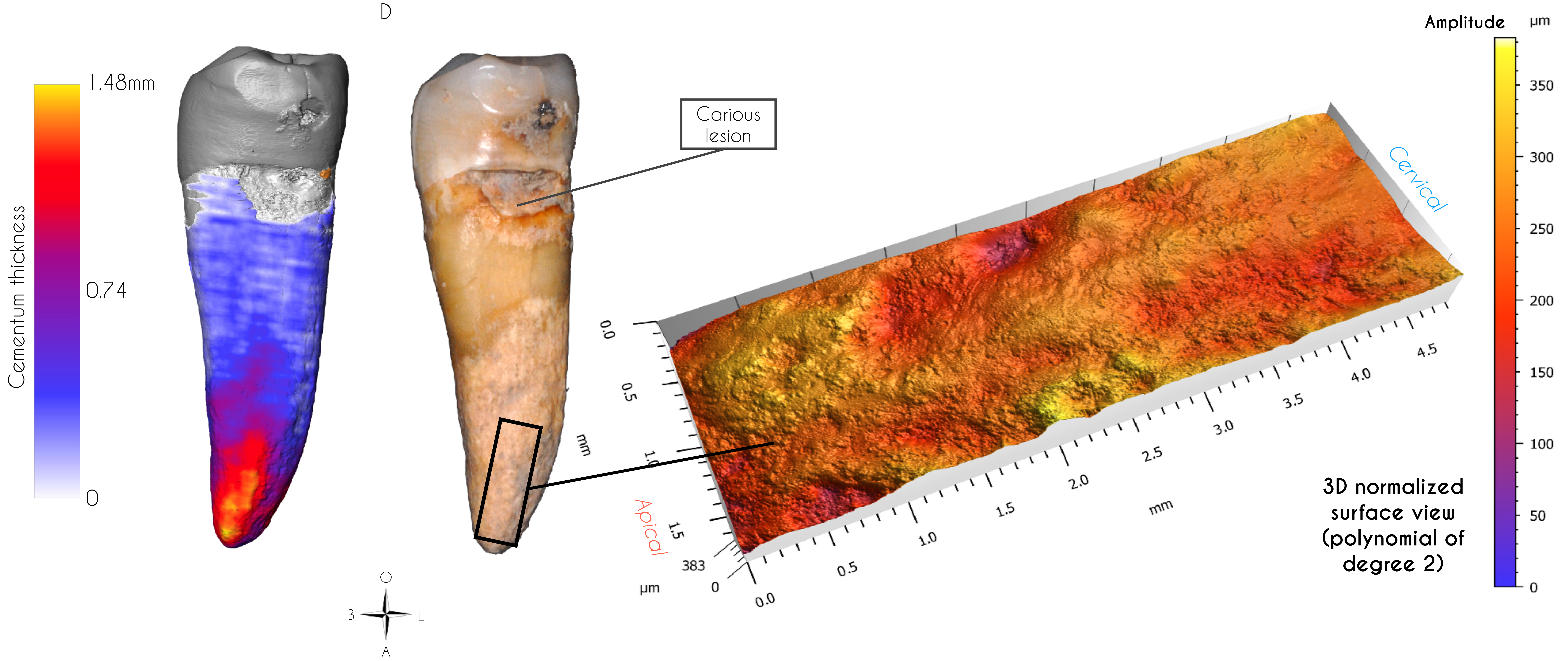

Confocal microscopy - Topography 3D maps			
LOC MAX	MAX VE (µm)	ST	Note
1d	375	-R2	-

LOC MAX: location of maximum thickness (1: apical root third; 2: middle root third; 3: cervical root third; m: mesial; d: distal; <: buccal; >: lingual);
MAX VE: maximum vertical elevation;
ST: surface texture; : scored (**+**) if it is high, (**-**) if it is low; **S:** smooth or **R:** rough; the frequency of occurrence of the elevations scored (**1**) if the frequency is high, (**2**) if the frequency is low.

Mixed

Sp1300_14

Tooth	Occlusal Wear			CAR Si/Sta	Pulp EXP	IMP	Hypercementosis	Bone Context			
	DEG	DIR	FOR					FEN	CAL	NT	ANTA
**URP3**	**7**	**4**	**4**	**2/2**	**0**	**0**	**1.2.M**	**0**	**0**	**NA**	**2**

URP3

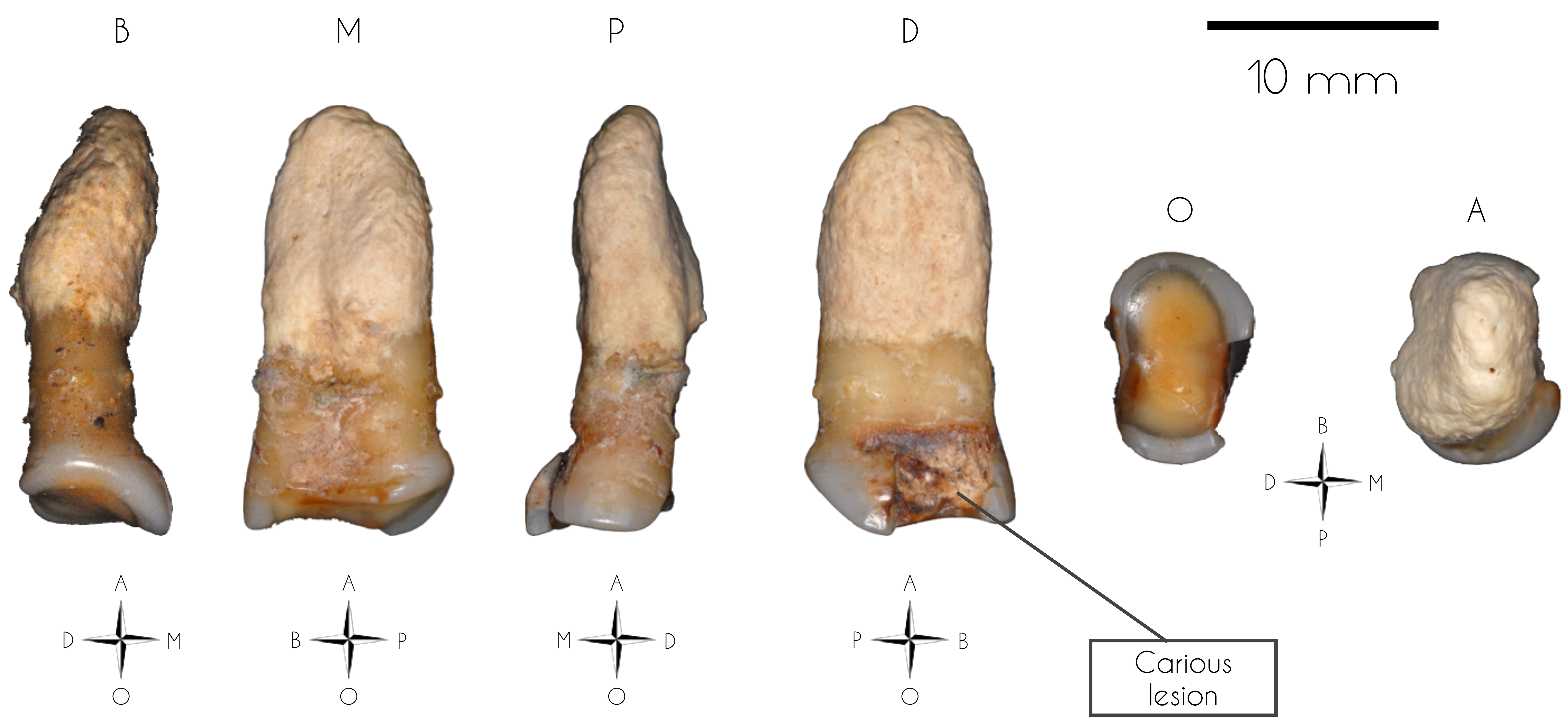

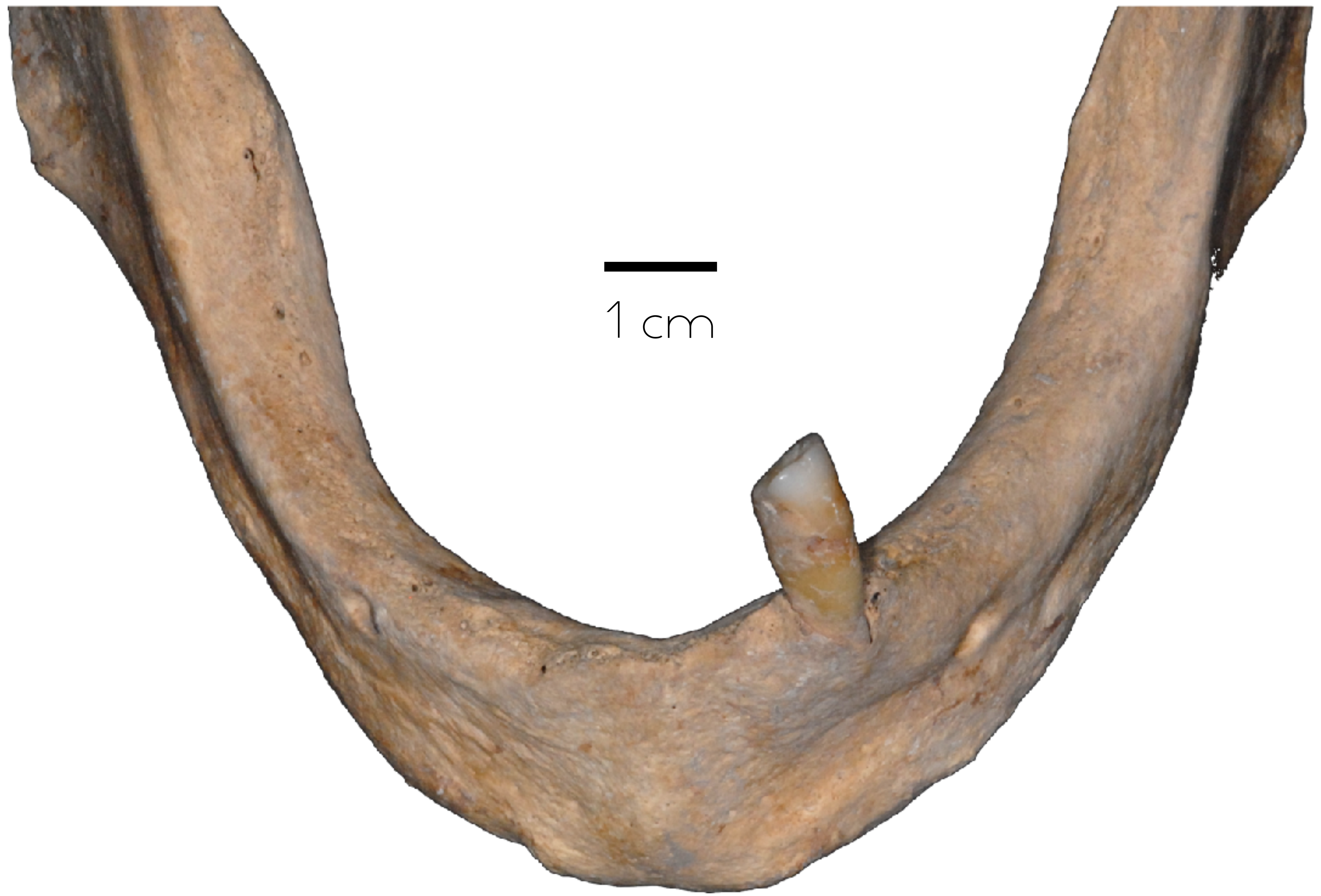

DEG: degree; **DIR:** direction; **FOR:** form - **CAR**: carious lesion - **Pulp EXP:** pulp exposure - **IMP**: impaction - **FEN:** fenestration; **CAL:** calculus; **NT:** tooth loss in neighbouring teeth; **ANTA:** antagonist

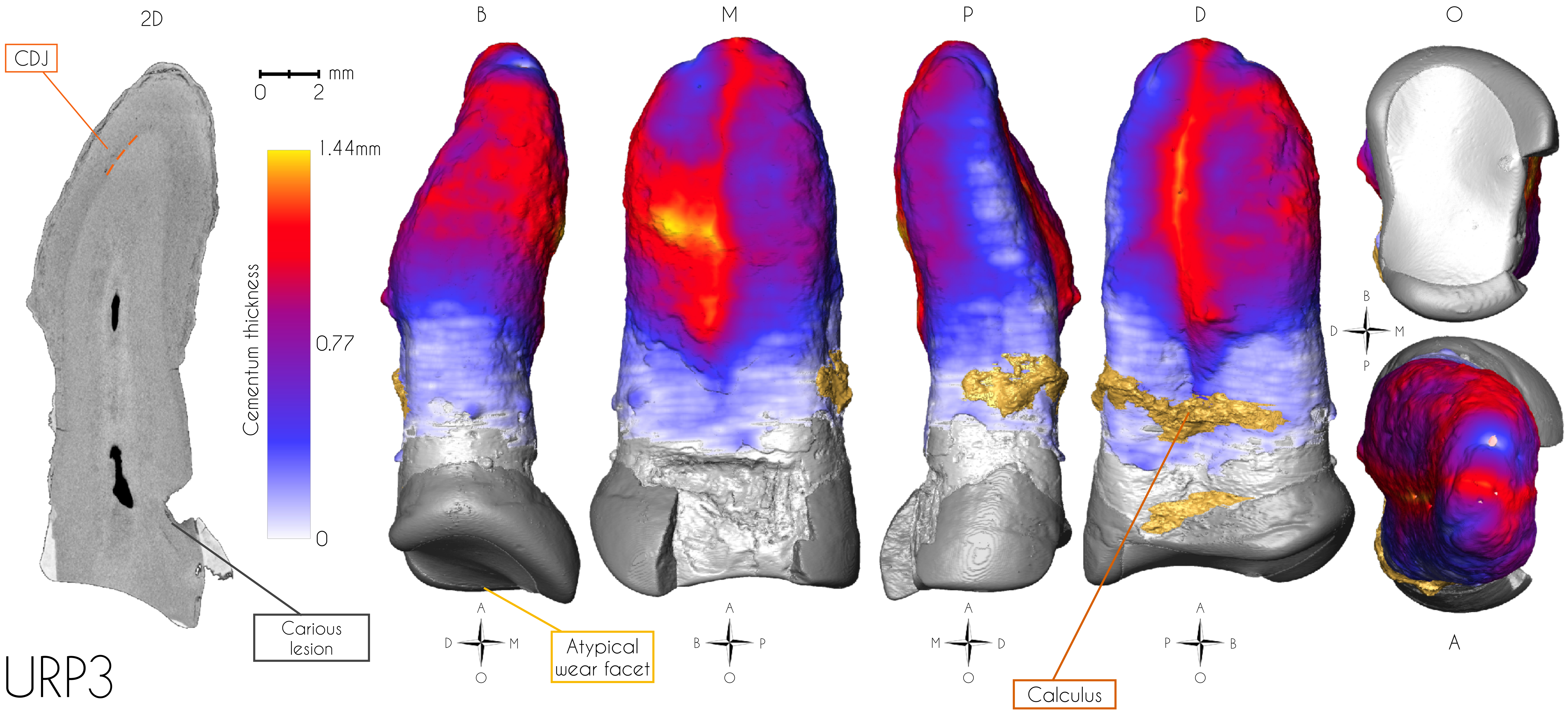

Sp1300_14

Microtomography - Thickness 3D maps				
MAX THI (μm)	LOC MAX	LOC MIN	PREF LOC	Note
1440	1m	>	No	CAR (m)

MAX THI: maximum thickness of cementum;
LOC MAX: location of maximum thickness;
LOC MIN: location of minimum thickness;
PREF LOC: preferential location;
CAR: carious lesion;
(1: apical root third; 2: middle root third; 3: cervical root third; m: mesial; d: distal; <: buccal; >: lingual)

Mixed

URP3

Face	Range	Light	Width	Height	Columns/Rows	Factor	Led Color	Gain	Gamma	Overlapping
M	1158	6.42%	1.67mm	4.23mm	2/7	2	Blue	1.47	1.61	10 %

Sp1300_14

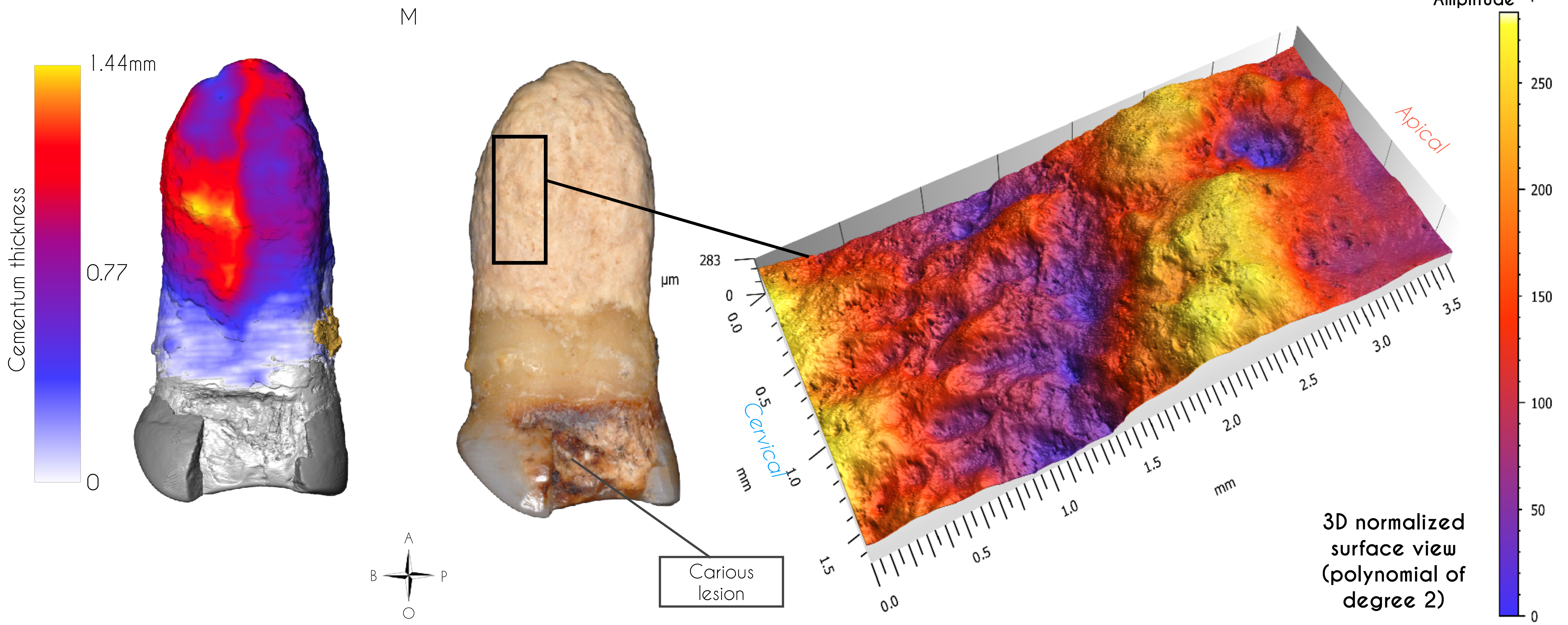

3D normalized surface view (polynomial of degree 2)

Confocal microscopy - Topography 3D maps			
LOC MAX	MAX VE (µm)	SA	Note
2m	280	+R1	-

LOC MAX: location of maximum thickness (1: apical root third; 2: middle root third; 3: cervical root third; m: mesial; d: distal; <: buccal; >: lingual);
MAX VE: maximum vertical elevation;
ST: surface texture; : scored (**+**) if it is high, (**-**) if it is low; **S:** smooth or **R:** rough; the frequency of occurrence of the elevations scored (**1**) if the frequency is high, (**2**) if the frequency is low.

Mixed
