# Supplementary material for: Unraveling the Life History of Past Populations through Hypercementosis: Insights into Cementum Apposition Patterns and Possible Etiologies Using Micro-CT and Confocal Microscopy
Source: Biology (Basel). 2024 Jan 13;13(1):43. doi: 10.3390/biology13010043 (PMC10813066; doi:10.3390/biology13010043)
Supplement: Supplementary file 1 [file biology-13-00043-s001.zip › Supplementary Information S2.pdf]

Unraveling the Life History of Past Populations through Hypercementosis: Insights into Cementum Apposition Patterns and Possible Etiologies using Micro-CT and Confocal Microscopy

Supporting Information S2: statistical analyses and R code

Léa Massé, Emmanuel d'Incau, Antoine Souron, Nicolas Vanderesse, Frédéric Santos, Bruno Maureille, Adeline Le Cabec

December 12, 2023

Contents

1 Configuration **2**

2 Load and summarize data **3**

3 Link between each variable and etiology **6**
3.1 Continuous variables 6
3.2 Ordinal traits (dental wear) 7
3.3 Qualitative traits 9

4 Multivariate analyses **11**
4.1 Factor Analysis of Mixed Data (FAMD) 11
4.2 Decision tree 16

1. Configuration

This document provides additional results that are not presented in the main text of our article, and presents the R code written for this study. All the analyses were performed using R 4.3.2 (R Core Team, 2023), and this document has been built with Org mode 9.6.13 for GNU Emacs 29.1 (Schulte, Davison, Dye, & Dominik, 2012).

To improve the reproducibility of our results, the following R packages are loaded using their version available on CRAN at a fixed date (2023-12-05), thanks to the R package {groundhog} (Simonsohn & Gruson, 2021):

```
date <- "2023-12-05"
library(groundhog)

groundhog.library("cowplot", date = date)
groundhog.library("factoextra", date = date)
groundhog.library("FactoMineR", date = date)
groundhog.library("ggpubr", date = date)
groundhog.library("ggrepel", date = date)
groundhog.library("missMDA", date = date)
groundhog.library("rattle", date = date)
groundhog.library("rpart", date = date)
groundhog.library("tidyverse", date = date)
```

More details about the R session and the versions of the R packages can be found below:

```
print(sessionInfo(), locale = FALSE)
```

```
R version 4.3.2 (2023-10-31)
Platform: x86_64-pc-linux-gnu (64-bit)
Running under: Manjaro Linux

Matrix products: default
BLAS:   /usr/lib/libblas.so.3.12.0
LAPACK: /usr/lib/liblapack.so.3.12.0

attached base packages:
[1] stats     graphics  grDevices utils     datasets  methods   base

other attached packages:
 [1] lubridate_1.9.3  forcats_1.0.0    stringr_1.5.1    dplyr_1.1.4
 [5] purrr_1.0.2      readr_2.1.4      tidyr_1.3.0      tidyverse_2.0.0
 [9] rpart_4.1.21     rattle_5.5.1     bitops_1.0-7     tibble_3.2.1
[13] missMDA_1.19     ggrepel_0.9.4    ggpubr_0.6.0     FactoMineR_2.9
[17] factoextra_1.0.7 ggplot2_3.4.4    cowplot_1.1.1    groundhog_3.1.2
```

2. Load and summarize data

The data file, available on Zenodo (Massé et al., 2023), is loaded and then summarized in R:

```r
## Load data sheet from CSV file, and apply various conversions
## of types for the variables:
dat <- read.csv2(
    file = "https://page.hn/dvqtfy", # data file on Zenodo
    na.strings = "NA",
    row.names = 1,
    stringsAsFactors = TRUE
) |>
    mutate(across(Wear_DEG:Wear_FOR, as.ordered)) |>
    mutate(across(c(CAR:ANT, MAX_TOMO_1:PREF, ST_pm:ST_12), as.factor))
## Summary of the dataframe:
summary(dat, maxsum = 9)
```

```
Wear_DEG Wear_DIR Wear_FOR CAR    PULP_EXP IMP    HYP_type HYP_stage HYP_Form
0:5      0:5      0:5      0:23   0:26     0:31   1:22     1: 2      m:21
1:2      1:4      1:4      1:10   1: 7     1: 2   3:11     2:19      M:12
2:2      2:6      2:8                                      3: 5
3:6      3:2      3:4                                      4: 7
4:4      4:8      4:6
5:1      5:1      5:1
6:7      6:6      6:5
7:3      7:1
8:3
FEN    CAL        NT     ANT        MAX_THI      MAX_TOMO_1 MAX_TOMO_2 MAX_TOMO_3
0:31   0:26   0    :17   0: 2   Min.   : 740    0:11       0:27       0:33
1: 2   1: 7   1    : 9   1:15   1st Qu.:1180    1:22       1: 6
              2    : 4   2: 8   Median :1380
              NA's: 3    3: 8   Mean   :1418
                                3rd Qu.:1510
                                Max.   :2770



MAX_TOMO_inf MAX_TOMO_sup MAX_TOMO_d MAX_TOMO_m MIN_TOMO_1 MIN_TOMO_2
0:31         0:23         0:19       0:24       0:32       0:33
1: 2         1:10         1:14       1: 9       1: 1







MIN_TOMO_3 MIN_TOMO_inf MIN_TOMO_sup MIN_TOMO_d MIN_TOMO_m PREF
0:33       0:13         0:31         0:33       0:32       0:13
           1:20         1: 2                    1: 1       1:20
```

```
  MAX_MICRO        ST_pm      ST_SR     ST_12        ETIO
Min.   : 43.0   minus:11   R   :20   1   :10   HYPER:12
1st Qu.:173.8   plus :21   S   :12   2   :22   HYPO : 5
Median :250.0   NA's : 1   NA's: 1   NA's: 1   IMP  : 2
Mean   :295.8                                  INF  : 4
3rd Qu.:412.5                                  MIX  :10
Max.   :690.0
NA's   :1
```

The following abbreviations are used for the variables names:

- `Wear_DEG`: degree, `DIR`: direction, `FOR`: form (according to a classification by Molnar (1971)). A score of 0 was assigned to teeth for which it was impossible to assess wear due to the absence of the dental crown;
- `CAR`: carious lesion (0: absence, 1: presence);
- `PULP_EXP`: pulp exposure (0: absence, 1: presence);
- `IMP`: impacted teeth (0: absence, 1: presence);
- `HYP_type`: type of hypercementosis; 1: diffuse apposition (cellular cementum apposition covering on a variously broad height and circumference of the root); 2: focal or local apposition (cementum apposition restricted to a precise point of the root); 3: combination of 1 and 2;
- `HYP_stage`: describing the apical root third covered by hypercementosis; 1: apical third, 2: up to the middle third, 3: up to the cervical third. Stage 4: partially or fully damaged cemento-enamel junction;
- `HYP_form`: form of hypercementosis, defined by direct visual observation of the extent of cementum thickness in regards to the natural shape of the root; m: moderate (apposition of small to medium thickness), M: marked (apposition of significant thickness);
- `FEN`: bone fenestration (0: absence, 1: presence);
- `CAL`: calculus (0: absence, 1: presence);
- `NT`: *ante-mortem* tooth loss in neighboring teeth (0: tooth loss, 1: loss of one neighboring tooth, 2: loss of both left and right neighboring teeth, NA: not applicable because the scored tooth is impacted);
- `ANT`: antagonist tooth (0: not applicable because the scored tooth is impacted; 1: presence; 2: *ante-mortem* loss; 3: not available because of missing data (incomplete associated osteological context);
- `MAX_THI`: maximum thickness of cementum (µm);

- MAX_TOMO: location of maximum cementum thickness. This variable is sub-divided into several sub-variables, which are scored as absence (0) or presence (1). The number corresponds to the location in terms of root thirds: MAX_TOMO_1 (apical), MAX_TOMO_2 (middle), MAX_TOMO_3 (cervical). When the cemento-enamel junction was not visible, the number was omitted. The symbol corresponds to the location on the root divided into sides, MAX_TOMO_m (mesial), MAX_TOMO_d (distal), MAX_TOMO_inf (buccal), MAX_TOMO_sup (lingual);

- MIN_TOMO: location of minimum cementum thickness. The naming and scoring of the sub-variables are designed the same way as for MAX_TOMO;

- PREF: preferential location of cementum apposition (0: no, 1: yes);

- MAX_MICRO: maximum vertical elevation of cementum apposition (µm);

- ST: surface texture. This variable is sub-divided into several sub-variables. ST_pm corresponds to the vertical elevation, plus: $\geqslant$ 200 µm, minus: < 200 µm. ST_SR corresponds to the surface texture, S: smooth, R: rough. ST_12 corresponds to the frequency of occurrence of the elevations (i.e., if the positive reliefs are close to each other), 1: high frequency, 2: low frequency;

- ETIO: supposed etiologies. IMP: impacted teeth ($n = 2$), INF: infected teeth ($n = 4$), HYPO: hypofunctional teeth ($n = 5$), HYPER: hyperfunctional teeth ($n = 12$), MIX: mixed condition ($n = 10$);

3. Link between each variable and etiology

In this section, we simply explore and represent graphically the link between the five etiology groups, and each continuous variable or qualitative trait in the dataset.

3.1. Continuous variables

See Figures 1 et 2.

```
## Set a color palette:
my.colors <- c("#ff9966", "#3366cc", "#cc3300", "#009933", "#cc0099")

## Variable MAX_THI :
ggplot(dat, aes(x = ETIO, y = MAX_THI, color = ETIO)) +
    geom_violin() +
    stat_summary(fun.y = mean, geom = "point", pch = 5, size = 4) +
    geom_jitter(width = 0.1) +
    theme_bw(base_size = 16) +
    theme(legend.position = "none") +
    labs(x = "Etiology", y = "Maximum thickness of cementum (µm)") +
    scale_colour_manual(values = my.colors)
```

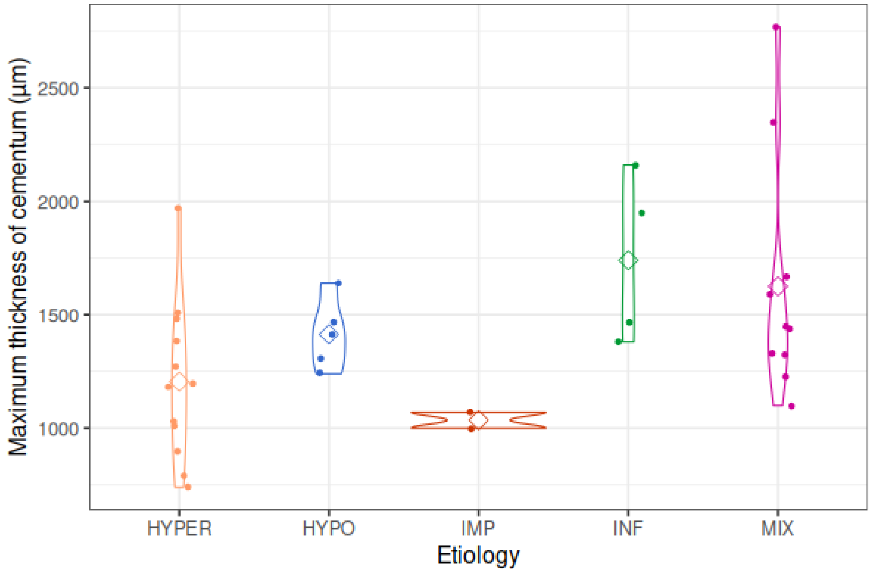

Figure S1: Individual values and mean value of `MAX_THI` by etiology.

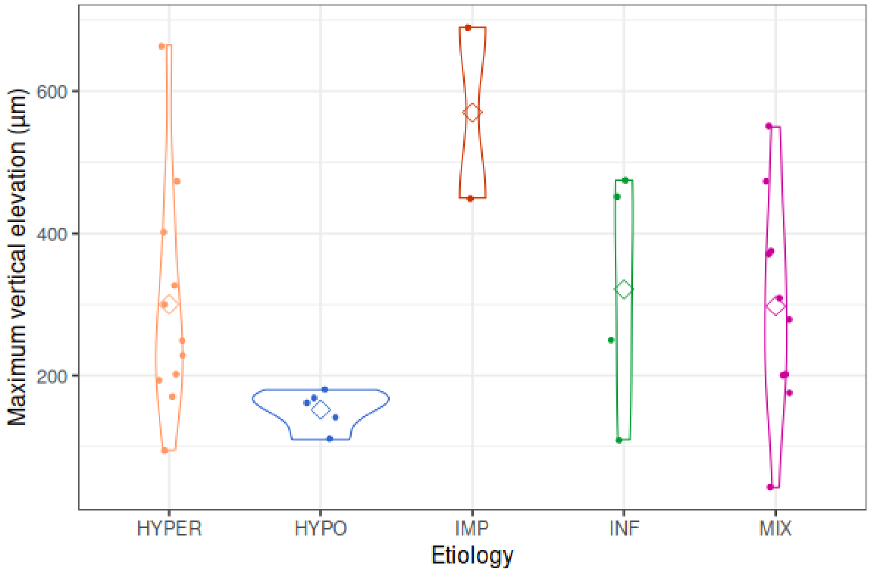

Figure S2: Individual values and mean value of `MAX_MICRO` by etiology.

3.2. Ordinal traits (dental wear)

See Figure 3.

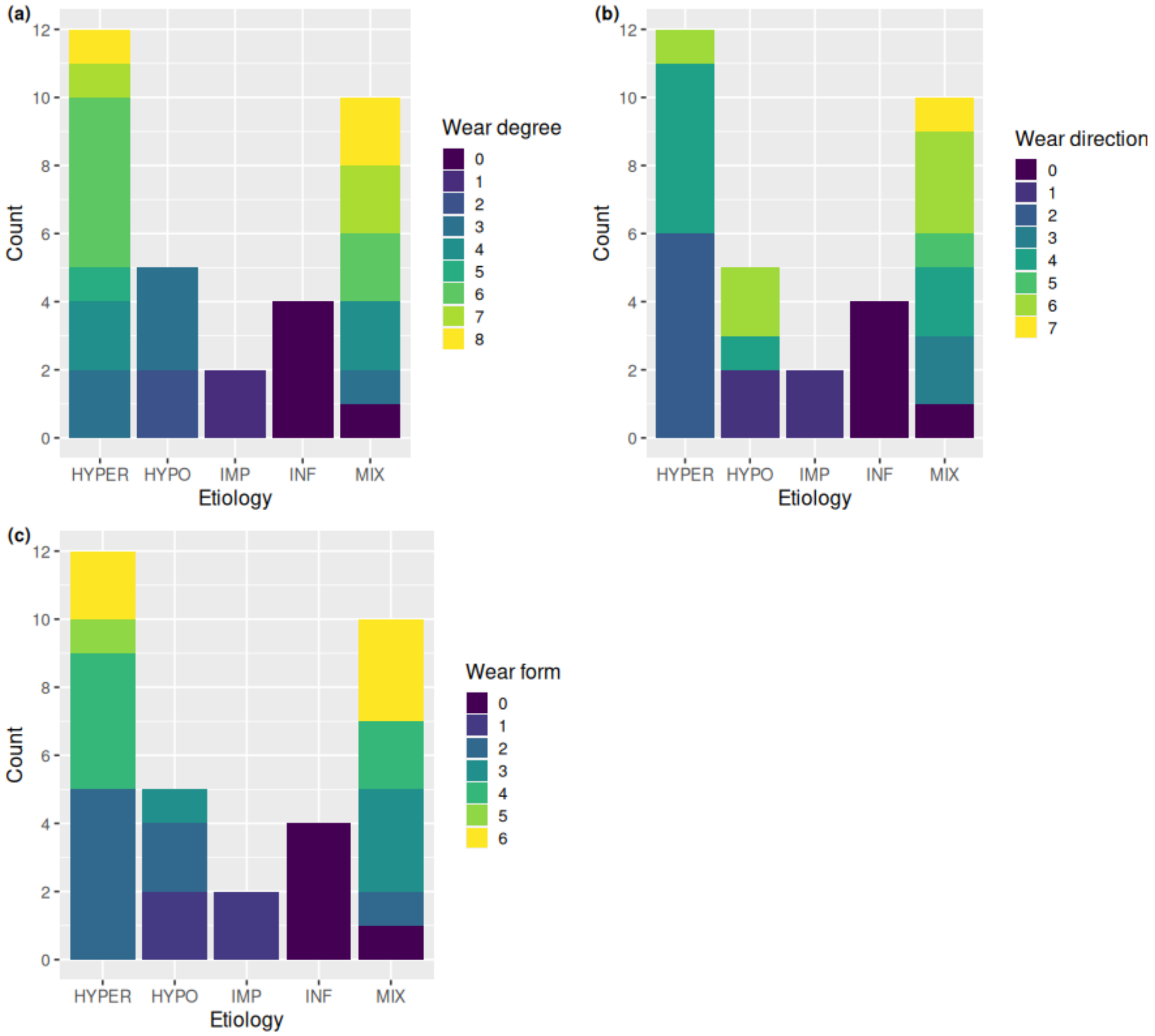

Figure S3: Barplots for wear degree (a), direction (b), and form (c) by etiology following Mol-nar (1971). Molnar's classification was used to evaluate occlusal wear using three criteria:(i) degree of wear: from 1 (no wear) to 8 (major wear, the tooth crown is totally worn away, and the chewing surface is on the root itself); (ii) direction of the worn surface: natural (1), oblique (2 to 5), horizontal (6) or rounded (7 and 8); (iii) form of the worn surface: natural (1), flat (2), half or fully concave (3 and 4), notched (5) or rounded (6). A score of 0 was assigned to teeth for which it was impossible to assess wear due to the absence of the dental crown.

3.3. Qualitative traits

We first define a helper function to draw more easily various barplots for the qualitative traits.

```
## Helper function for drawing barplots:
my.barplot <- function(data, y, titre) {
    dtf <- data.frame(ETIO = data$ETIO, Y = data[, y])
    p <- ggplot(dtf, aes(x = ETIO, fill = Y)) +
        geom_bar(position = position_stack(reverse = TRUE), stat = "count") +
        theme_gray(base_size = 17) +
        scale_y_continuous(breaks = c(0, 2, 4, 6, 8, 10, 12)) +
        labs(x = "Etiology", y = "Count", fill = titre) +
        theme(legend.position = "top") +
        theme(legend.title = element_text(face = "bold"))
    p
}
```

Figure 4 presents various barplots, for a subset of traits that play an important role in subsequent analyses (see Section 4).

```
## Variable 'Preferential location' (PREF):
bar.pref <- my.barplot(data = dat, y = "PREF",
                       titre = "Preferential location")
## Variable 'Antagonist tooth' (ANT):
bar.ant <- my.barplot(data = dat, y = "ANT",
                      titre = "Antagonist tooth")
## Variable 'Pulp exposure' (PULP_EXP):
bar.pulpexp <- my.barplot(data = dat, y = "PULP_EXP",
                          titre = "Pulp exposure")
## Variable 'Tooth loss' (NT):
bar.nt <- my.barplot(data = dat, y = "NT",
                     titre = "Tooth loss in neighbouring teeth") +
    scale_fill_manual(values = c("lightskyblue", "cornflowerblue", "blue"))
## Composition of Figure:
cowplot::plot_grid(bar.pref, bar.ant, bar.pulpexp, bar.nt,
                   ncol = 2,
                   labels = c("(a)", "(b)", "(c)", "(d)"),
                   label_size = 18)
```

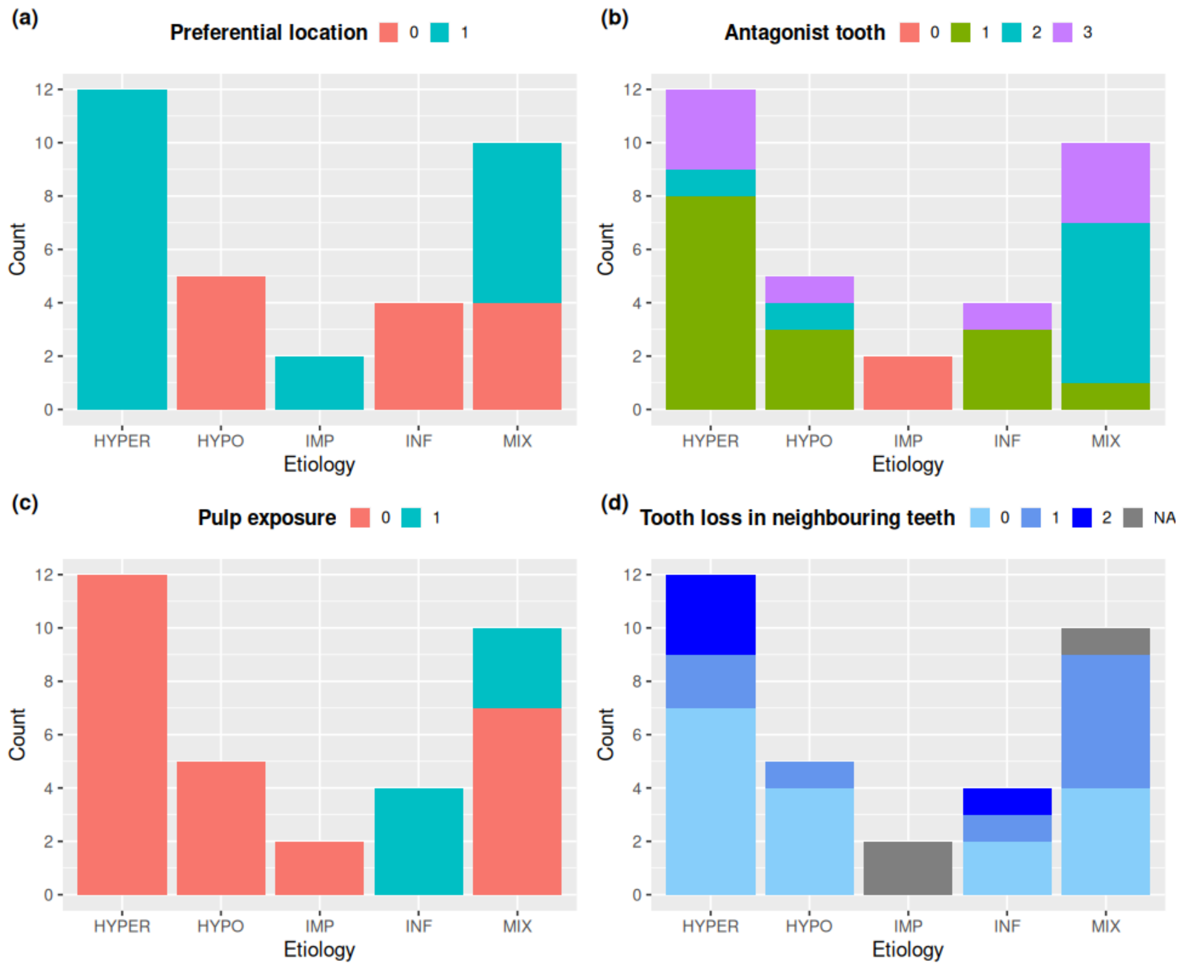

Figure S4: Barplots for various qualitative traits depending on etiology. (a) Preferential lo-cation and (c) Pulp exposure which are scored as 0: absence or 1: presence; (b) Antagonist tooth which are scored as, 0: not applicable if the scored tooth is impacted, 1: presence, 2: *ante-mortem* loss, 3: not applicable because of missing data (incomplete associated osteolog-ical context); (d) *Ante-mortem* tooth loss in neighboring teeth which are scored as 0: no tooth loss, 1: loss of one neighboring tooth, 2: loss of both left and right neighboring teeth, NA: not applicable because the scored tooth is impacted.

4. Multivariate analyses

4.1. Factor Analysis of Mixed Data (FAMD)

To perform a Factor Analysis of Mixed Data (Pagès, 2004), we considered the two continuous variables, the three ordinal traits (related to dental wear), and a subset of qualitative traits. We simply discarded the traits that were non-polymorphic (i.e., always equal to `0` or `1` in the whole sample), and a trait that was observed on one individual only. Thus, the following variables were included in the analysis:

```
 [1] "ETIO"         "Wear_DEG"     "Wear_DIR"     "Wear_FOR"     "CAR"
 [6] "PULP_EXP"     "HYP_type"     "HYP_stage"    "HYP_Form"     "FEN"
[11] "CAL"          "NT"           "ANT"          "MAX_THI"      "MAX_TOMO_1"
[16] "MAX_TOMO_2"   "MAX_TOMO_inf" "MAX_TOMO_sup" "MAX_TOMO_d"   "MAX_TOMO_m"
[21] "MIN_TOMO_inf" "MIN_TOMO_sup" "PREF"         "MAX_MICRO"    "ST_pm"
[26] "ST_SR"        "ST_12"
```

Then, we imputed the missing values using an iterative algorithm implemented in the R package `{missMDA}`:

```
## Imputation of NAs:
dtf.imp <- imputeFAMD(X = dtf)$completeObs
```

Finally, we computed the FAMD; the results for the first three components are represented in Figure 5.

```
## Compute the FAMD:
famd <- FAMD(dtf.imp, sup.var = 1, graph = FALSE)
## Extract the coordinates of individuals and factor levels:
indc <- data.frame(famd$ind$coord[, 1:3], ETIO = dtf$ETIO)
varc <- as.data.frame(famd$quali.var$coord[, 1:3])
## Extract the cos-squared for the factor levels:
cos2.12 <- apply(famd$quali.var$cos2[, 1:2], 1, sum)
cos2.23 <- apply(famd$quali.var$cos2[, 2:3], 1, sum)

## Plot axes (1,2):
famd.ind12 <- fviz_famd(X = famd, habillage = 1, geom = c("point"),
          select.var = list(cos2 = 0.56),
          pointsize = 2.5,
          axes = 1:2,
          invisible = "quali",
          col.quali.var = "gray50") +
    geom_text_repel(data = varc[cos2.12 >= 0.56, ],
                    mapping = aes(x = Dim.1, y = Dim.2),
                    label = rownames(varc[cos2.12 >= 0.56, ]),
                    nudge_y = 0.3, color = "gray50") +
    stat_chull(data = indc,
               aes(x = Dim.1, y = Dim.2, color = ETIO, fill = ETIO),
               geom = "polygon", alpha = 0.1) +
```

```
    theme_minimal(base_size = 15) +
    labs(color = "Etiology") +
    guides(fill = "none") +
    ggtitle("FAMD - Individuals (Axes 1-2)") +
    scale_color_manual(values = my.colors) +
    scale_fill_manual(values = my.colors)

famd.var12 <- plot(famd, choix = "quanti", axes = 1:2, title = "")

## Plot axes (2,3):
famd.ind23 <- fviz_famd(X = famd, habillage = 1, geom = c("point"),
          select.var = list(cos2 = 0.45),
          pointsize = 2.5,
          axes = 2:3,
          invisible = "quali",
          col.quali.var = "gray50") +
    geom_text_repel(data = varc[cos2.23 >= 0.45, ],
                    mapping = aes(x = Dim.2, y = Dim.3),
                    label = rownames(varc[cos2.23 >= 0.45, ]),
                    nudge_y = 0.3, color = "gray50") +
    stat_chull(data = indc,
               aes(x = Dim.2, y = Dim.3, color = ETIO, fill = ETIO),
               geom = "polygon", alpha = 0.1) +
    theme_minimal(base_size = 15) +
    labs(color = "Etiology") +
    guides(fill = "none") +
    ggtitle("FAMD - Individuals (Axes 2-3)") +
    scale_color_manual(values = my.colors) +
    scale_fill_manual(values = my.colors)

famd.var23 <- plot(famd, choix = "quanti", axes = 2:3, title = "")

## Compose a final figure:
cowplot::plot_grid(famd.ind12, famd.ind23, famd.var12, famd.var23,
                   ncol = 2,
                   labels = c("(a)", "(c)", "(b)", "(d)"))
```

The listing presented in pages 14–15 makes easier the description of the etiologies, by giving the categories (“Mod”) most associated to each etiology (or class, “Cla”). In the listing below, “Cla/Mod” and "Mod/Cla“ are respectively the percentages of the etiology within the category, and of the category within the etiology.

Thus, for instance, within the class HYPER of hyperfunctional teeth, 100% of the teeth have a preferential location of cementum apposition (i.e., are such that PREF=1); and conversely, among those teeth that have a preferential location of cementum apposition, 60% are in the class of hyperfunctional teeth.

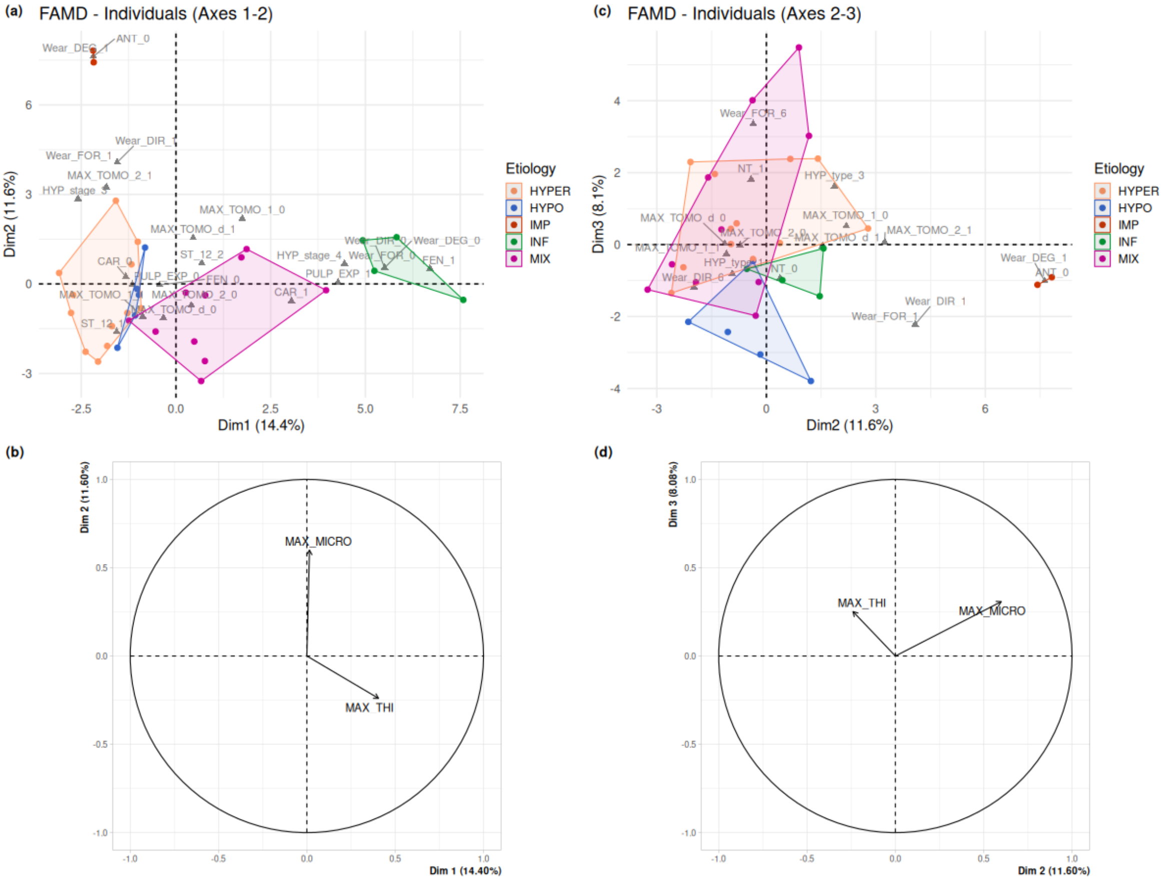

Figure S5: Factor Analysis of Mixed Data of the dataset. (a) and (b): Results for the first two principal axes; only those factor levels reaching a quality of representation (cos^2) greater than 0.56 are represented on (a). (c) and (d): Results for the principal axes 2 and 3; only those factor levels reaching a quality of representation (cos^2) greater than 0.45 are represented on (c).

```
$HYPER
                Cla/Mod   Mod/Cla   Global      p.value    v.test
PREF=1          60.00000 100.00000 60.60606 0.0003550278  3.571437
Wear_DIR=2     100.00000  50.00000 18.18182 0.0008342603  3.341170
MAX_TOMO_sup=1  80.00000  66.66667 30.30303 0.0012242948  3.233159
CAR=0           52.17391 100.00000 69.69697 0.0038106313  2.893427
PULP_EXP=0      46.15385 100.00000 78.78788 0.0272187953  2.208366
Wear_DEG=6      71.42857  41.66667 21.21212 0.0483870968  1.973953
ST_12=2         22.72727  41.66667 66.66667 0.0317873321 -2.147075
HYP_stage=4      0.00000   0.00000 21.21212 0.0272187953 -2.208366
PULP_EXP=1       0.00000   0.00000 21.21212 0.0272187953 -2.208366
CAR=1            0.00000   0.00000 30.30303 0.0038106313 -2.893427
MAX_TOMO_sup=0  17.39130  33.33333 69.69697 0.0012242948 -3.233159
PREF=0           0.00000   0.00000 39.39394 0.0003550278 -3.571437

$HYPO
             Cla/Mod Mod/Cla    Global     p.value    v.test
ST_pm=minus  45.45455     100 33.333333 0.001946607  3.098260
PREF=0       38.46154     100 39.393939 0.005422692  2.780789
Wear_DEG=2  100.00000      40  6.060606 0.018939394  2.346722
Wear_DEG=3   50.00000      60 18.181818 0.033041764  2.131575
HYP_stage=2  26.31579     100 57.575758 0.048993832  1.968645
PREF=1        0.00000       0 60.606061 0.005422692 -2.780789
ST_pm=plus    0.00000       0 63.636364 0.003337041 -2.934855

$IMP
             Cla/Mod Mod/Cla    Global     p.value    v.test
ANT=0       100.00000     100  6.060606 0.001893939  3.106379
Wear_DEG=1  100.00000     100  6.060606 0.001893939  3.106379
NT=NA        66.66667     100  9.090909 0.005681818  2.765600
Wear_FOR=1   50.00000     100 12.121212 0.011363636  2.531313
Wear_DIR=1   50.00000     100 12.121212 0.011363636  2.531313
HYP_stage=3  40.00000     100 15.151515 0.018939394  2.346722
MAX_TOMO_2=1 33.33333     100 18.181818 0.028409091  2.191590
MAX_TOMO_2=0  0.00000       0 81.818182 0.028409091 -2.191590

$INF
              Cla/Mod Mod/Cla    Global      p.value    v.test
Wear_FOR=0    80.000000     100 15.151515 0.0001221896  3.841691
Wear_DIR=0    80.000000     100 15.151515 0.0001221896  3.841691
Wear_DEG=0    80.000000     100 15.151515 0.0001221896  3.841691
HYP_stage=4   57.142857     100 21.212121 0.0008553275  3.334240
PULP_EXP=1    57.142857     100 21.212121 0.0008553275  3.334240
CAR=1         40.000000     100 30.303030 0.0051319648  2.798632
MAX_TOMO_1=0  36.363636     100 33.333333 0.0080645161  2.649357
FEN=1        100.000000      50  6.060606 0.0113636364  2.531313
PREF=0        30.769231     100 39.393939 0.0174731183  2.376598
HYP_stage=2    0.000000       0 57.575758 0.0244623656 -2.249789
PREF=1         0.000000       0 60.606061 0.0174731183 -2.376598
FEN=0          6.451613      50 93.939394 0.0113636364 -2.531313
MAX_TOMO_1=1   0.000000       0 66.666667 0.0080645161 -2.649357
CAR=0          0.000000       0 69.696970 0.0051319648 -2.798632
PULP_EXP=0     0.000000       0 78.787879 0.0008553275 -3.334240
```

```
$MIX
             Cla/Mod Mod/Cla   Global     p.value    v.test
ANT=2      75.000000      60 24.24242 0.004230722  2.860426
HYP_Form=M 58.333333      70 36.36364 0.013727503  2.464316
CAL=1      71.428571      50 21.21212 0.017241379  2.381519
CAR=1      60.000000      60 30.30303 0.024932974  2.242440
CAR=0      17.391304      40 69.69697 0.024932974 -2.242440
CAL=0      19.230769      50 78.78788 0.017241379 -2.381519
HYP_Form=m 14.285714      30 63.63636 0.013727503 -2.464316
ANT=1       6.666667      10 45.45455 0.008824620 -2.618775
```

4.2. Decision tree

Figure 6 presents a classification tree (Breiman, Friedman, Stone, & Olshen, 1984) for explaining the etiology using all covariates. This tree was not grown from a predictive point of view (i.e., no cross-validation was performed for pruning the tree at an optimal size that maximizes the accuracy rate when predicting new data points); but from an explanatory point of view instead. The tree was allowed to grow until one of the following stopping criteria was reached:

- the minimum number of observations that must exist in a node in order for a split to be attempted was set to 7 (argument `minsplit=7` in the code block below);
- the minimum number of observations in any terminal leaf was set to 2 (argument `minbucket=2` in the code block below). The rationale for this very low value is that the etiology "impacted" (IMP) has only two individuals, and we wanted to be able to characterize this etiology as well.

```
## Decision tree:
par(mar = c(1, 0.5, 0, 1))
arbre <- rpart(ETIO ~ ., data = dat,
               control = list(minsplit = 7, minbucket = 2))
plot(arbre, branch = 0.8, compress = TRUE, uniform = TRUE, margin = 0.1)
text(arbre, all = TRUE, pretty = 0, fancy = TRUE, use.n = TRUE)
```

References

Breiman, L., Friedman, J., Stone, C. J., & Olshen, R. A. (1984). *Classification and Regression Trees*. Taylor & Francis.

Massé, L., d'Incau, E., Souron, A., Vanderesse, N., Santos, F., Maureille, B., & Le Cabec, A. (2023). *Data file for Massé et al.'s article, "Unraveling the Life History of Past Populations through Hypercementosis: Insights into Cementum Apposition Patterns and Possible Etiologies using Micro-CT and Confocal Microscopy"*. Zenodo. doi:10.5281/zenodo.10357391

Molnar, S. (1971). Human tooth wear, tooth function and cultural variability. *American Journal of Physical Anthropology*, *34*(2), 175–189. doi:10.1002/ajpa.1330340204

Pagès, J. (2004). Analyse factorielle de données mixtes. *Revue de Statistique Appliquée*, *52*(4), 93–111.

R Core Team. (2023). *R: A language and environment for statistical computing. R Foundation for Statistical Computing, Vienna, Austria.*

Schulte, E., Davison, D., Dye, T., & Dominik, C. (2012). A Multi-Language Computing Environment for Literate Programming and Reproducible Research. *Journal of Statistical Software*, *46*(1), 1–24. doi:10.18637/jss.v046.i03

Simonsohn, U., & Gruson, H. (2021). *Groundhog: Reproducible Scripts via Version-Specific Package Loading.*

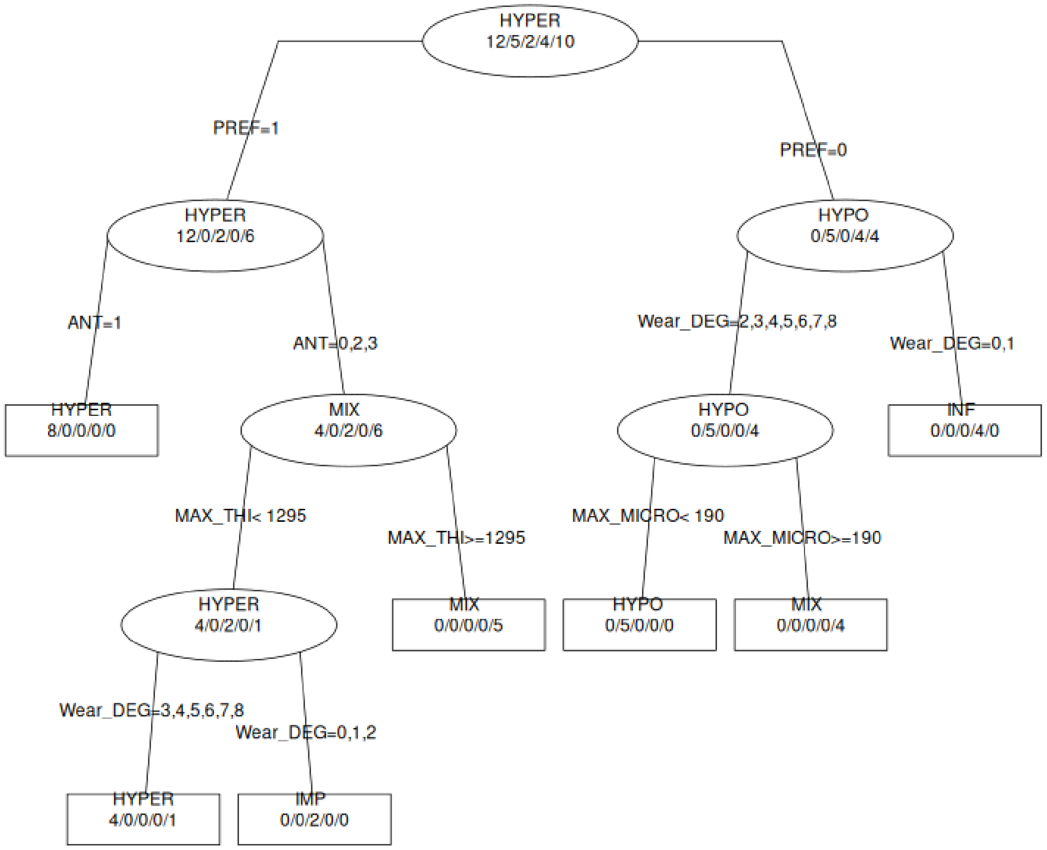

Figure S6: Classification tree for explaining the etiology using all covariates. Terminal leaves are represented as rectangles, while intermediate nodes are represented as ellipses. In each node, the majority class is displayed, along with the number of individuals in the classes `HYPER / HYPO / IMP / INF / MIXED` respectively.
